# Topologies of synthetic gene circuit for optimal fold change activation

**DOI:** 10.1093/nar/gkab253

**Published:** 2021-05-01

**Authors:** Phyana Litovco, Natalia Barger, Ximing Li, Ramez Daniel

**Affiliations:** Department of Biomedical Engineering, Technion - Israel Institute of Technology, Haifa 3200003, Israel; Department of Biomedical Engineering, Technion - Israel Institute of Technology, Haifa 3200003, Israel; Department of Biomedical Engineering, Technion - Israel Institute of Technology, Haifa 3200003, Israel; Department of Biomedical Engineering, Technion - Israel Institute of Technology, Haifa 3200003, Israel

## Abstract

Computations widely exist in biological systems for functional regulations. Recently, incoherent feedforward loop and integral feedback controller have been implemented into *Escherichia coli* to achieve a robust adaptation. Here, we demonstrate that an indirect coherent feedforward loop and mutual inhibition designs can experimentally improve the fold change of promoters, by reducing the basal level while keeping the maximum activity high. We applied both designs to six different promoters in *E. coli*, starting with synthetic inducible promoters as a proof-of-principle. Then, we examined native promoters that are either functionally specific or systemically involved in complex pathways such as oxidative stress and SOS response. Both designs include a cascade having a repressor and a construct of either transcriptional interference or antisense transcription. In all six promoters, an improvement of up to ten times in the fold change activation was observed. Theoretically, our unitless models show that when regulation strength matches promoter basal level, an optimal fold change can be achieved. We expect that this methodology can be applied in various biological systems for biotechnology and therapeutic applications.

## INTRODUCTION

**Figure 1.**
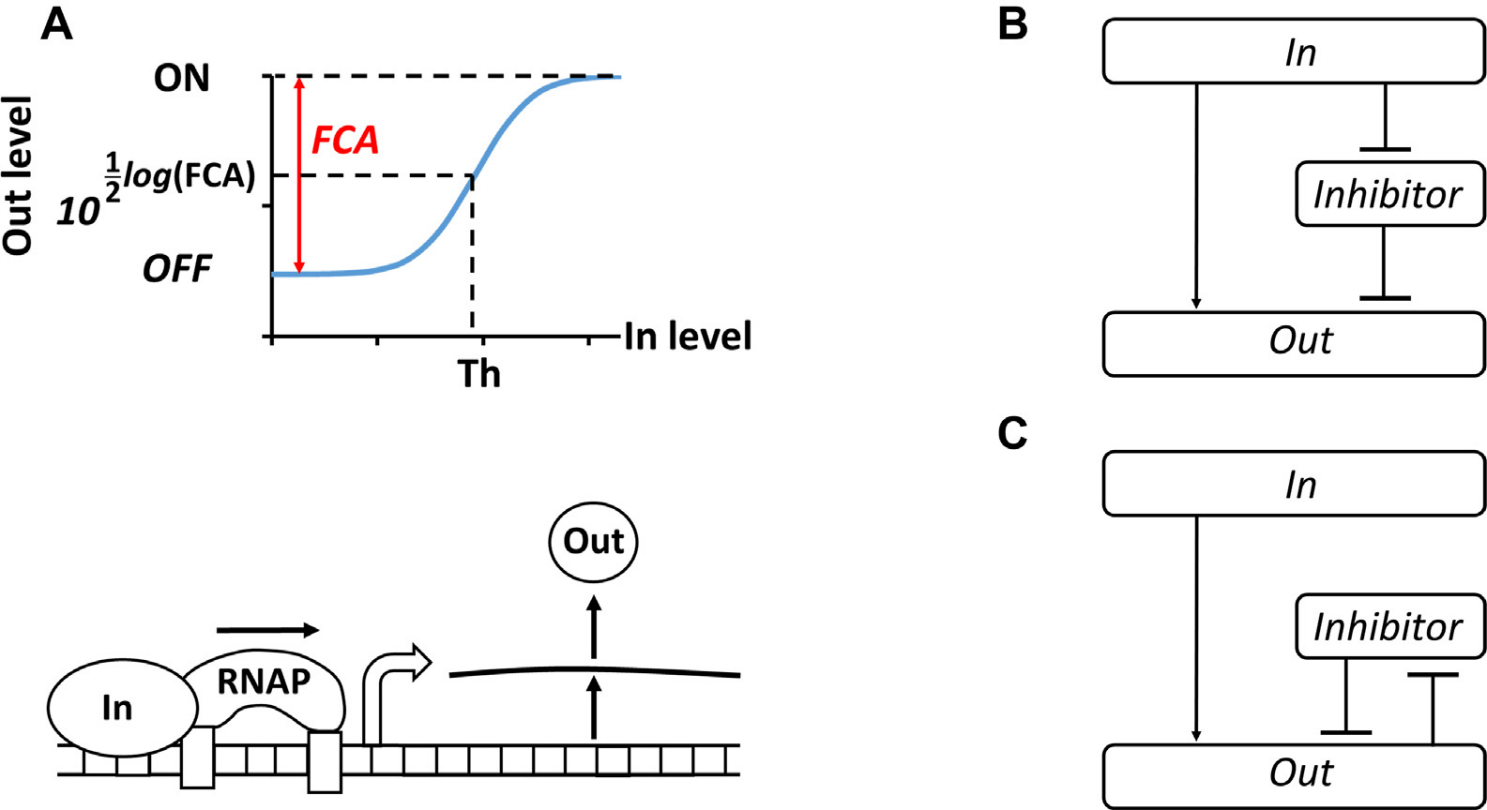
Fold change activation (FCA) and block diagrams for ICF and DNF designs. (**A**) Describes the transfer function of promoter activity (Supplementary Figure S1A). FCA is the ratio between ON and OFF states of the promoter. The OFF state is the minimum activity of the promoter and is achieved when no input molecules are present and there is only leaky gene expression (basal level) due to the unspecific binding of RNA polymerases (RNAPs). The ON state is the maximum activity of the promoter. ‘Th’ is the input threshold of genetic switches and equals to half of FCA on the logarithmic scale. (**B**) Schematic diagram of the indirect coherent feedforward (ICF) circuit. Input molecules regulate both the inhibitor and the output level. (**C**) Schematic diagram of the mutual inhibition through double negative feedback (DNF) circuit. A positive feedback between the inhibitor and the output is coupled through mutual repression.

Gene regulatory networks in cells are often sensitive to the fold-change response of bio-molecular signals and not to their absolute change, e.g. bacterial chemotaxis sensory systems, signaling pathways, and human perception of sound intensity, light intensity, smell and weight (1–4). Such a property is governed by Weber's law, which states that the ratio between the perceptual change in a signal divided by its background level is a constant (4). Fold-change activation (FCA) in gene regulatory networks is defined as the ratio between the ON state, when an activated promoter has maximum activity, and the OFF state (background level or basal level), when RNA polymerases bind to the promoter in the absence of stimulus (e.g. transcription factor) (Figure 1A). Genetically engineered systems require designs, such as feedforward and feedback loops, able to optimize FCA level by achieving a balance between the low basal level (leakiness) and the maximum gene expression. However, the implementation of such designs in living cells is often challenging (5).

So far, genetic circuits have been implemented in living cells using two major computational paradigms (6). The first is the digital paradigm, which uses two discrete binary-coded levels for computation (7), including logic gates (8–10), memory elements (11–13), a counter (14), state machines (15), an oscillator (16), a toggle switch (17) and a digitizer (18). The second is the analog paradigm (19) which takes advantage of mathematically based functions that are naturally present in cells, as well as feedforward and feedback loops over a continuous input range, to perform processing and complex temporal dynamics computations (20,21). Control theory principles have been implemented in both paradigms (20,22).

The basal levels of promoter activity and their FCA play a significant role in determining the behavior of gene circuits in both digital and analog designs. In a digital design, the FCA can be used to tune the threshold of the response function (Figure 1A). Circuits with high FCA exhibit distinct ON and OFF states, which can be directly used for screening or noise filtering. Analog gene circuits with high FCA also have high output dynamic ranges and can properly operate with high computational precision. In contrast, the performance of digital and analog circuits with low FCA are sensitive to environmental changes, having a very narrow noise margin and making them challenging to program (23,24). In metabolic engineering, using inducible promoters with a high basal level of enzyme expression leads to toxicity in cells (25–28). Expressing proteins in high copy numbers can cause inadvertent interactions between molecules involved in essential pathways and can lead to interference with pathway functions due to competitive binding. Furthermore, increasing the FCA can improve the performance of bacterial biosensors by enhancing the signal-to-noise ratio (29), or increase the detection sensitivity of target molecular signals that are at very low concentrations of bacterial biosensors with high basal levels (5).

Several methods have been applied to manage the basal level of promoter activity, such as using antisense transcription (26,30–33), fusing ssrA degradation tags to transcription factors to control their levels in cells (29), altering ribosome-binding sequences (19,34) and mutual inhibition (35). These methods reduce the basal level as well as the maximum level of promoter activity resulting in a decreased FCA level. Thus, it is necessary to reduce the basal level of promoter without changing its maximal level.

To this end, we have developed two designs: (i) an indirect coherent feedforward (ICF) circuit (Figure 1B), and (ii) a mutual inhibition through double negative feedback (DNF) circuit (Figure 1C). In this study, we applied these two designs to six different promoters, showing that the strategy improves their FCA levels.

A feedforward loop is ‘coherent’ when an input signal is split into two downstream pathways, both of which positively regulate the output (36). In an ICF circuit, one pathway is formed by cascading two inhibitors. Such a network is known as coherent-feedforward type 4 (36). For a low level of input, the inhibitor is highly active and strongly reduces the leakage of the output. For a high level of input, the inhibitor level is low and therefore, the maximum output level is kept high. A positive feedback loop is mutual when the two nodes in the feedback loop negatively regulate each other (17). In our case (Figure 1C), the input activates the output, whereas the inhibitor and the output repress each other. Such a mutual repression design using DNF is often used in bistable switches (37,38) and can lead to ultrasensitivity (39). The ICF and DNF designs reduce only the basal output without impacting the maximum output, leading to an increased FCA level. Here, the implementation of ICF and DNF designs in *Escherichia coli* was achieved by using repressor and antisense promoters based on two regulatory mechanisms. The first mechanism involves antisense RNA that can bind to the mRNA preventing its translation (40,41). The second mechanism is based on transcriptional interference, where the forward (sense) promoter is positioned in a clockwise (5′ to 3′) direction and fused to a reverse (antisense) promoter that is positioned in a counterclockwise (3′ to 5′) direction. The RNA polymerases of the forward and reverse promoters interact directly via collisions that lead to downregulation of the gene (42).

### Linear models

**Figure 2.**
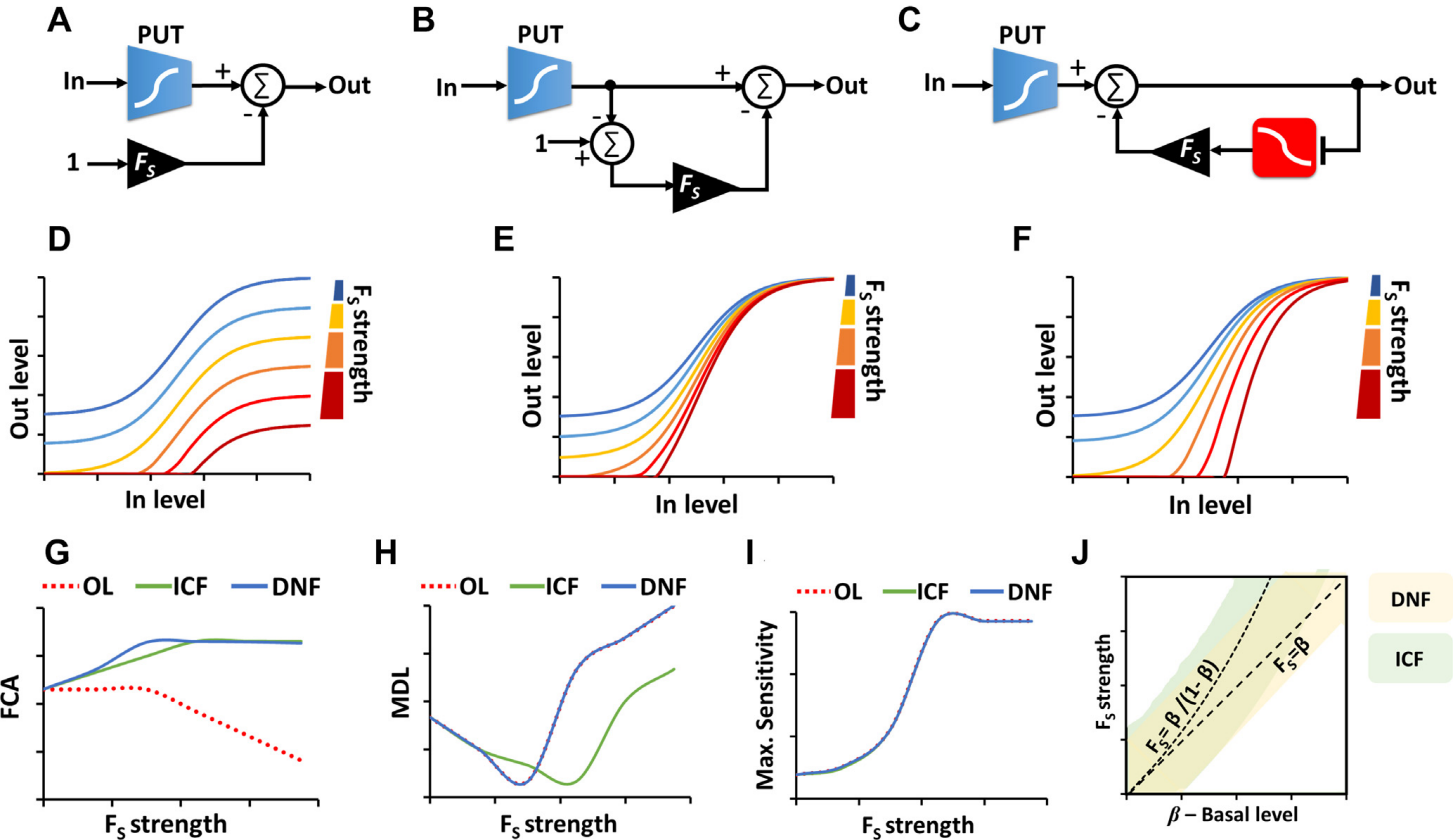
Linear models for ICF and DNF designs. (**A**) Block diagram for the open loop (OL) circuit. The part under test (PUT) has a non-linear monotonic function with two distinct levels: (i) normalized minimum level- β (e.g. basal level of promoter activity) and (ii) normalized maximum level ‘1’. The output of the OL circuit is obtained by subtracting *F*_s_ from the output of the PUT. The connecting node has two inputs. One with a positive sign that is connected directly to the output of the PUT target circuit, and the second is with a negative sign that is connected to a constant ‘1’ through a gain of *F*_s_. (**B**) Block diagram for the indirect coherent feedforward (ICF) circuit. The output of the PUT is split into two branches that both positively regulate the circuit output. The difference between the two branches determines the circuit output. The first branch is directly connected to circuit output and the second branch includes a two-stage subtraction with a gain of *F*_s_. (**C**) Block diagram for the mutual inhibition through double negative feedback (DNF) circuit. The output of the PUT is regulated through a negative feedback loop formed by an inverter (e.g. repressor) with gain of *F*_s_. (**D**) Simulation results of the OL circuit. (**E**) Simulation results of the ICF circuit. (**F**) Simulation results of the DNF circuit. (**G**) FCA level versus *F*_s_ strength for OL, ICF and DNF circuits. (**H**) Minimum detection level (MDL) versus *F*_s_ strength for OL, ICF and DNF circuits. MDL is defined as the input level when the sensitivity is maximum. (**I**) Maximum sensitivity versus *F*_s_ strength for OL, ICF and DNF circuits. The sensitivity is calculated at every input point, based on $S\ = \frac{{dOut/\langle Out\rangle }}{{dIn/\langle In\rangle }}$. (**J**) Qualitative *β – F_S_* diagrams for ICF and DNF designs. The area marked in light green corresponds to maximal FCA and the area marked in light yellow corresponds to minimal MDL. Outside of these areas, the FCA and MDL are not optimal.

We chose to base our approach on coherent feedforward and mutual inhibition designs, which are commonly found in natural transcriptional networks (36,43) and have been used in a number of engineered biological systems (17). We expected that a coherent feedforward design would be simpler than mutual inhibition, because it can function in a wide range of biological contexts. To gain deeper insights into these designs, for example, to understand how the strength of feedforward/feedback can affect FCA level, circuit sensitivity and minimum detection level (MDL), we built three simple linear models: open loop design -OL (Figure 2A and Supplementary Figure S1B), ICF (Figure 2B and Supplementary Figure S1C), and DNF in (Figure 2C and Supplementary Figure S1D). In these models, we assume that the part (circuit/promoter/device) under test has a non-linear monotonic function with two distinct levels, a minimum normalized level ‘*β*’ and a normalized maximum level ‘1’. All the other operations are linear (e.g. subtraction and inhibition, Supplementary Information, Section 1). The OL circuit includes a part under test (PUT) that is directly connected to a subtraction with a strength of *F*_s_ (OUT_OL_ = OUT_PUT_ – *F*_s_). Evidently, enhancing the *F*_s_ level decreases the basal and maximum levels of OL circuit by the same amount. According to this simple analysis, we do not expect that an increase in the *F*_s_ strength can enhance the FCA in the OL circuit. Furthermore, since in reality signals cannot fall below zero, thereby the FCA level decreases as *F*_s_ levels increase (Figure 2D and Supplementary Figure S2A). Conversely, in the ICF (Figure 2E and Supplementary Figure S2B) and DNF (Figure 2F and Supplementary Figure S2C) designs, the FCA levels start to increase with rising *F*_s_ until reaching a plateau (Figure 2G and Supplementary Figure S3A). The sensitivity, which is evaluated by calculating the ratio between the fold change in the output relative to the fold change in the input (Supplementary Equation Eq. S3), and it's maximum is the same for the three circuit designs (Figure 2I, Supplementary Figure S2D–F, and Supplementary Figure S3C). The MDL, which is defined as the input level when the sensitivity is maximum, is another important parameter that should be considered when designing gene circuits, because in many cases the target molecules are present at very low concentrations. The linear models show that there is a tradeoff between FCA and MDL (Figure 2H and Supplementary Figure S3B), meaning that there is a specific *F*_s_ value that yields minimum MDL and maximum FCA (*F*_s_*= β*/(1 – *β)* for ICF, and *F*_s_*= β* for DNF design). Our qualitative analysis of the *β – F*_s_ relations for the ICF and DNF circuits shows areas where the MDL and FCA are optimal (Figure 2J and Supplementary Figure S4). Further analysis of the linear models for OL, ICF and DNF designs is provided in Supplementary Information, Section 1.

### Molecular models with nonlinearities

**Figure 3.**
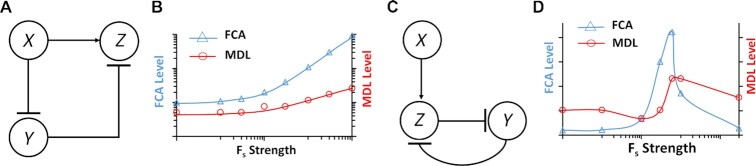
Models for ICF and DNF circuits based on biochemical reactions. (**A**) Schematic diagram for molecular ICF network. Molecule *Z* is activated by molecule *X* and repressed by molecule *Y*, which is activated by *X*. (**B**) Simulation results of FCA and MDL for molecular ICF circuit. (**C**) Schematic diagram for molecular DNF circuit. Similar to the ICF, but here the molecule *Z* also represses *Y*. (**D**) Simulation results of FCA and MDL for the molecular DNF circuit. Simulation parameters: *β = 0.1, α = 10, n = 1.5, m = 1, h = 1*.

To capture the behavior of biochemical reactions, we replaced the linear operations (e.g. subtraction) in the ICF and DNF designs with non-linear operations (e.g. Hill-function) (Figure 3A and Supplementary Figure S11 for ICF and Figure 3C and Supplementary Figure S14 for DNF, Supplementary Information, Sections 2.2.1 and 2.3.1). In these models, the molecule *Z* is activated by molecule *X* and is repressed by molecule *Y* as described in Equation (1). Molecule *Y* is repressed by molecule *X* in the ICF circuit (Figure 3A) and is described by Equation (2), whereas in the DNF circuit (Figure 3C), molecule *Y* is also repressed by molecule *Z* and is described by Equation (3):

(1) $$\begin{equation*}z = \alpha \frac{{{x^n} + \beta }}{{1 + {x^n}}}\frac{1}{{1 + {y^m}}}\end{equation*}$$

(2) $$\begin{equation*}y = \frac{{{F_S}}}{{1 + {x^n}}}\end{equation*}$$

(3) $$\begin{equation*}y = \frac{{{F_S}}}{{1 + {z^h}}}\end{equation*}$$

where *x, y, z* are the dimensionless concentrations of molecules *X*, *Y* and *Z*, respectively (x *= X/K*_dx_*, y = Y/K*_dy_*, z = Z/K_dz_*, where *K*_dx_*, K*_dy_ and *K*_dz_ are proportional to dissociation constants; we assumed that *X* has the same binding affinity to *Y* and *Z*). *n*, *m* and *h* are Hill coefficients, *F*_s_ is the strength of the feedforward/feedback loops, *β* is basal level and *α* is a parameter representing the activation strength of *X* on *Z*. The simulation results of FCA for the nonlinear molecular ICF network is shown in Figure 3B. The FCA exhibits similar behavior to the linear model of the ICF circuit. In both cases, FCA increases monotonically with rising *F*_s_ strength. The MDL of the molecular ICF circuit also rises when the *F_s_* increases, compared to the MDL of the linear model which has an optimum. Interestingly, simulation of FCA and MDL for the molecular DNF network (Figure 3C) show an optimum for a narrow range of *F*_s_. These simulation results suggest that ICF behaves more desirable, however, we decided to build the DNF versions for further investigation. Detailed analysis of the molecular OL, ICF and DNF circuits is provided in Supplementary Information Section 2.

## MATERIALS AND METHODS

### Chemicals

Nalidixic acid (NA), hydrogen peroxide (H_2_O_2_), sodium (meta)arsenite (AsNaO_2_), hemin, arabinose, isopropyl-β-D-1-thiogalactopyranoside (IPTG), anhydrotetracyclin hydrochloride (aTc) and acyl homoserine lactone 3OC6HSL (AHL) were used as inducers and were obtained from Sigma-Aldrich. For a list of parameters, see Supplementary Information, Table S4.

### Bacterial strains, plasmids and gene origins

Standard molecular cloning techniques were used for plasmids construction. New England Biolab's (Beverly, MA) restriction endonucleases, Thermo Scientific FastDigest Restriction Enzymes, T4 DNA Ligase were used for plasmid construction. All plasmids in this work were built and transformed to NEB 10-beta *Escherichia coli* (*araD139 D(ara-leu)7697 fhuA lacX74 galK (W80 D(lacZ)M15) mcrA galU recA1 endA1 nupG rpsL (StrR) D(mrr-hsdRMS-mcrBC*). The P_katG_, P_recA_ and P_arsR_ promoters were obtained by PCR amplification (Phusion High-Fidelity PCR Kit – New England Biolabs) from the genome of MG1655 *E. coli* (*F− λ− ilvG− rfb-50 rph-1*), with primers listed in Supplementary Information, Table S3. For part amplification from the genome, 5 ml of MG1655 strain *E. coli* were inoculated from frozen glycerol stocks and were grown for 16 h. The next morning, 5 μl from the overgrown culture was mixed with 15 μl of DNase and RNase free water, heated at 96°C for 6 min and incubated at –80°C for 10 min. 2 μl from this solution was added into PCR mixture with total volume of 50 μl. The primers were synthesized by Integrated DNA Technologies (Leuven, Belgium). Plasmids for cloning were transformed into chemically competent *E. coli* 10-beta with a standard heat shock protocol (44). Bacterial cultures were consistently cultured at 37°C in Luria-Bertani (LB) Broth, Miller (Difco). The overnight grown cells were grown from glycerol stocks in 5 ml at 37°C or inoculated from colonies on agar plate with appropriate antibiotics for plasmid preparation in the next morning. The growth media was supplemented with appropriate concentration of antibiotics: carbenicillin (50 μg/ml), kanamycin (30 μg/ml) or/and chloramphenicol (34 μg/ml). Plasmids were extracted from the bacterial cells with QIAprep Spin Miniprep Kit (Qiagen, Hilden, Germany) according to the manufacturer's manual. Colony screening was carried out by PCR with suitable forward and reverse primers. Sequencing was approved by Macrogen Sequencing Service (Macrogen Europe, the Netherlands). All synthetic parts used in this work are listed in Supplementary Information, Table S2 and the plasmid maps are included in Supplementary Information, Sections 5 and 6.

### Plasmid construction

All plasmids in this work were constructed in a similar manner: Promoter-RBS-gene-terminator-origin-of-replication-antibiotic-resistance, where the origin-of-replication was cut with AvrII and SacI restriction enzymes, the gene was digested with restriction enzymes KpnI and BamHI, and the antibiotic resistance was cut with SacI and AatII restriction enzymes. Different combinations of plasmids forming different synthetic circuits (are summarized in Supplementary Information, Table S1) were transformed into competent *E. coli* 10-beta or MG1655 *E. coli* either through heat-shock protocol (44) or electroporation protocol (45).

### Cytometry measurement and data analysis

Different combinations of plasmids forming different synthetic circuits were transformed into competent NEB 10-beta *E. coli* for cytometry measurements except from P_LhrtO_ and P_recA_, which were transformed into MG1655 *E. coli* wild-type strain. The bacterial cultures were inoculated from colonies on agar plate the previous day and grown in 5 ml of LB with appropriate antibiotics at 37°C and 300 r.p.m. In the morning, the overnight grown bacterial cultures were diluted 1:100 into fresh LB medium (for P_lacO_, P_LhrtO_ and P_arsR_ circuits) or were diluted 1:50 into fresh M9 minimal medium [1× M9 Salts (Sigma-Aldrich, M6030), 2 mM MgSO_4_, 100 μM CaCl_2_, 0.4% glucose, 0.1% casamino acids, 50 mg/l thiamine] (for P_BAD_, for P_BADsyn_, P_katG_ and P_recA_) for the flow cytometry experiment with appropriate concentrations of antibiotics and incubated for specific time for regrowth and adaptation in fresh media, as described in Supplementary Information, Section 3 for each promoter. Bacterial cultures were transformed into 96-well plates with known concentrations of inducers to total volume of 200 μl, incubated in microplate shaker (37°C, 500 r.p.m) for relevant time described in Section 3 for each promoter until they reached optical density of OD_600 nm_ ∼ 0.4–0.7. Then, the fluorescence and scattering of bacterial cultures were analysed through flow cytometry analyzer (CytoFLEX S Flow Cytometer). In all experiments 10000 events have been obtained and the fluorescence and forward and side scattering were taken using CytExpert 2.2 software. The fluorescence distribution data over population data were extracted together with its geometric mean from each well in 96-well plate and plotted using MATLAB. Fluorescence measurement was based on geometric mean of flow cytometry populations from three experiments. The flow cytometry data for one representative experiment for each combination, which was independently repeated for two more times, is provided in Supplementary Information, Section 4. Next, the figures were built in EXCEL, based on geometric mean of flow cytometry populations with error bars representing the standard deviation errors of the geometric mean.

## RESULTS

### ICF in natural biological systems

**Figure 4.**
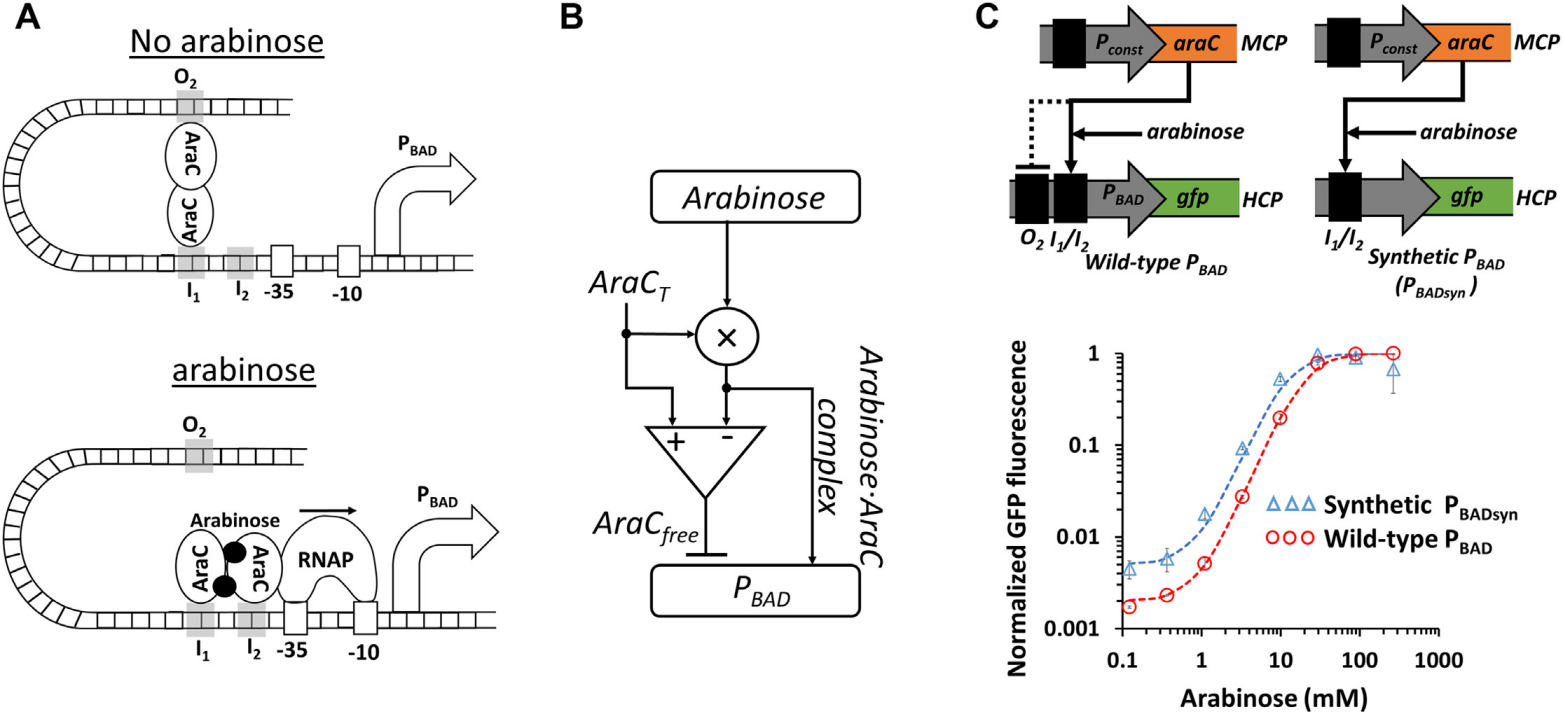
ICF design describes l-arabinose utilization system. (**A**) The structure of P_BAD_ promoter in l-arabinose utilization system. In the absence of arabinose, a loop between O_2_ and I_2_ binding sites is formed through AraC, which prevents RNA polymerase from accessing the promoter. When arabinose is present, the loop is released and AraC binds to I_1_ and I_2_ sites. This leads to RNA polymerase (RNAP) binding to DNA sites (-35, -10) and the initiation of transcription. (**B**) A diagram model for AraC and P_BAD_ promoter showing that the system resembles an ICF network. On the one hand, the arabinose acts as an input to activate the P_BAD_ by forming arabinose–AraC complex. On the other hand, the free AraC represses the P_BAD_ promoter and is equal to the total concentration of AraC (AraC_T_) minus the arabinose-AraC complex concentration. (**C**) The measured transfer function of wild-type P_BAD_ and synthetic P_BAD_ (P_BADsyn_). The synthetic P_BADsyn_ contains only I_1_ and I_2_ binding sites without O_2_ DNA sites. AraC is expressed by a constitutive promoter, encoded on a medium-copy-number plasmid (MCP). The synthetic P_BADsyn_ and wild-type P_BAD_ promoters regulate green fluorescent protein (GFP), encoded on a high-copy-number plasmid (HCP). The dotted lines are Hill function fittings. All experimental data are averaged from three experiments.

We started our study by searching for natural biological systems that contain ICF and DNF designs. We found that the ICF network naturally occurs in the l-arabinose utilization system (Figure 4A) (46,47). In the absence of arabinose, AraC protein binds araI_1_ (I_1_) and araO_2_ (O_2_) DNA binding sites by rigidly holding the DNA binding domains through its N-terminal arm. This conformation creates a loop in the DNA that prevents the RNA polymerase from binding to initiate transcription. When arabinose is present, the physically closed loop is released and AraC moves to bind araI_1_ (I_1_) and araI_2_ (I_2_) DNA binding sites. The opened loop allows RNA polymerase to freely access the promoter, and the positioning of a DNA binding domain of AraC at I_2_ facilitates transcription initiation by RNA polymerase. According to this explanation, a diagram model that describes the P_BAD_ promoter system is shown in Figure 4B. While the arabinose–AraC complex activates the P_BAD_ promoter, the free AraC represses the P_BAD_ promoter. Since the total amount of AraC is equal to the amount of free AraC and that of the arabinose-AraC complex, in our model, the arabinose participates in two circuit branches. The first is driven by the complex (arabinose-AraC) which directly activates the output. The second branch indirectly activates the output through double inhibitions: the free AraC and what resides from the complex. Motivated by this model, we modified the wild-type P_BAD_ that has I_1_/I_2_ and O_2_ binding sites, by removing O_2_ binding sites (Figure 4C and Supplementary Figure S24). The new synthetic P_BADsyn_ promoter has only I_1_/I_2_ binding sites. Thus, in the absence of arabinose, RNA polymerase can bind to the P_BADsyn_ promoter, leading to higher leaky gene expression than the wild-type P_BAD_ promoter (Figure 4C).

### Implementation of ICF and DNF designs

**Figure 5.**
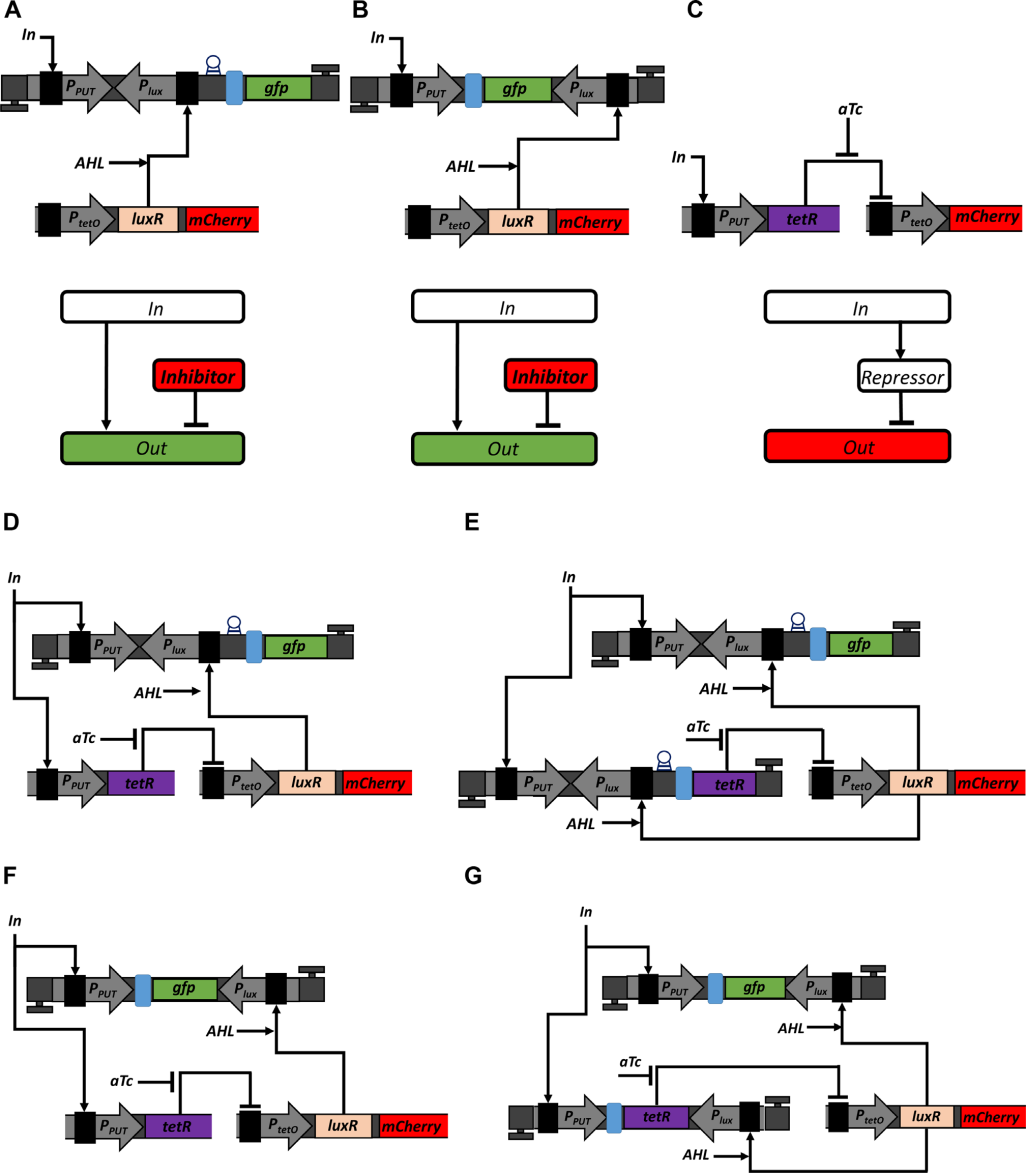
Implementation of ICF and DNF designs in living cells. (**A**) Utilization of transcriptional interference to mimic subtraction. The P_PUT_ activates GFP signal. The P_lux_ reverse promoter is located opposite to P_PUT_ and upstream to *gfp* gene repressing GFP signal. The first unidirectional terminator is in the same orientation as P_PUT_ and downstream to *gfp* gene. The second unidirectional terminator is in the same orientation as P_lux_ and upstream to P_PUT_. The terminator is represented by a highlighted letter T. The RBS is marked by a blue rectangle. The riboj sequence is inserted upstream of the RBS which is marked by a circle (59). The LuxR transcription activator and mCherry are expressed under P_tetO_ promoter, encoded on MCP. When no TetR is expressed, P_tetO_ acts as a constitutive promoter. Both *LuxR* and *mCherry* genes have their own RBS sequences. The unit Terminator__RC_-P_PUT_-P_lux_RC_-GFP-Terminator is encoded on HCP. The block diagram describes the operation of OL circuit, where the output is regulated both by the input and inhibitor. (**B**) Utilizing antisense transcription to mimic subtraction. The P_lux_ promoter is oriented in reverse to P_PUT_ and downstream to *gfp* gene repressing GFP signal. The first unidirectional terminator was placed in the same orientation to P_PUT_ and downstream to *gfp* gene. The second unidirectional terminator was placed in the same orientation to P_lux_ and upstream to P_PUT_. The LuxR activator and mCherry are expressed by P_tetO_ promoter encoded on MCP. Both LuxR and mCherry genes have their own RBS sequences. The unit Terminator__RC_-P_PUT_-GFP-P_lux_RC_-Terminator is encoded on HCP. The block diagram describes the operation of OL, where both input and inhibitor regulate the output level. (**C**) Implementation of an inverting switch using TetR repressor. The P_PUT_ controls the expression of TetR, which represses the activity of P_tetO_. The small molecule aTc binds TetR to release the repression of P_tetO_. The P_tetO_-mCherry-Terminator construct was placed on MCP, while the P_PUT_-TetR-Terminator construct was cloned on LCP in order to match their copy numbers in ICF and DNF circuits. The mCherry gene was further replaced by *LuxR* gene to be integrated in ICF and DNF circuits. The block diagram describes the operation of an inverting switch circuit. (**D**) Implementation of ICF circuit by combining a transcriptional interference unit with TetR inverting switch. Here TetR is controlled only by P_PUT_. (**E**) Implementation of a DNF circuit by combining transcriptional interference unit with TetR inverting switch. Here TetR is controlled by both P_PUT_ and P_lux_ promoters. (**F**) Implementation of ICF circuit by combining an antisense transcription unit with TetR inverting switch. Here TetR is controlled only by P_PUT_. (**G**) Implementation of a DNF circuit by combining an antisense transcription with TetR inverting switch. Here TetR is controlled by both promoters P_PUT_ and P_lux_.

We started to implement the ICF and DNF designs in living cells by mimicking a subtraction using a transcriptional interference system and an antisense transcription system. In both systems, we placed the P_lux_ promoter in opposite orientation to promoter under test (P_PUT_), which inhibits the P_PUT_ activity. We start with the transcriptional interference system (Supplementary Figures S5A and S6) that involves P_PUT_ followed by a downstream transcriptional-regulation component. The interference component, P_lux_ promoter, is oriented in the opposite direction to P_PUT_ and located upstream to the *gfp* gene_._ Thus, in such a system, the output GFP signal is activated by P_PUT_ promoter and is repressed by transcription of the reverse P_lux_ promoter. This special organization allows interference between forward and reverse promoters due to collisions of RNAPs actively transcribing from these promoters. The second system is the antisense transcription (30) (Supplementary Figure S5B) that also involves a P_PUT_ and an interference component from P_lux_ promoter. The P_lux_ promoter is oriented oppositely to P_PUT_ and is located downstream to *gfp* gene. Consequently, the output GFP signal is activated by P_PUT_ promoter and is repressed by the antisense (reverse) P_lux_ promoter, where both DNA strands are fully transcribed in both directions to produce mRNA and antisense RNA. This special organization allows interference between forward and reverse promoters due to direct interaction between mRNA and antisense RNA. Further analysis of transcriptional interference and antisense transcription is provided in Supplementary Information, Section 2.1 and Supplementary Figures S7–S10. In this study, we successfully built a protocol including five steps that guarantees an improvement in the FCA of P_PUT_. In the first two steps, we characterize the behavior of the transcriptional interference unit (Figure 5A) and antisense transcription unit (Figure 5B) by varying acyl homoserine lactone (AHL) concentration. The AHL binds to transcription factor LuxR and forms a complex which activates the transcription of P_lux_ promoter. Thus, by varying AHL concentration, we can control the strength of the feedforward/feedback loop (*F*_s_). In the third step, we implement an inverting switch through TetR repressor (Figure 5C) which is regulated by P_PUT_. We can tune P_tetO_/TetR behavior either by changing TetR level (fusion with different ssrA degradation tags) or by varying anhydrotetracycline (aTc) concentration. After selecting an optimal aTc concentration, which gives the highest ON/OFF ratio of LuxR levels represented by mCherry, we can implement the ICF and DNF gene circuits. Then, we will apply ICF and DNF circuits by combining the inverting switch as described in step three with the transcriptional interference unit (Figure 5D and E, respectively) or antisense transcription unit (Figure 5F and G, respectively). The difference between ICF and DNF implementations is that in the ICF design, TetR is regulated only by P_PUT_ and GFP is regulated by P_PUT_ and P_lux_, whereas in the DNF design, both proteins, GFP and TetR are regulated by P_PUT_ and P_lux_. Based on the genetic circuits shown in Figure 5, we modified the three-nodes molecular models to create genetic four-nodes models. The new models showed that in both ICF and DNF circuits an optimum FCA level is achieved when *F*_s_ increases (Supplementary Information, Sections 2.2.2 and 2.3.2).

**Figure 6.**
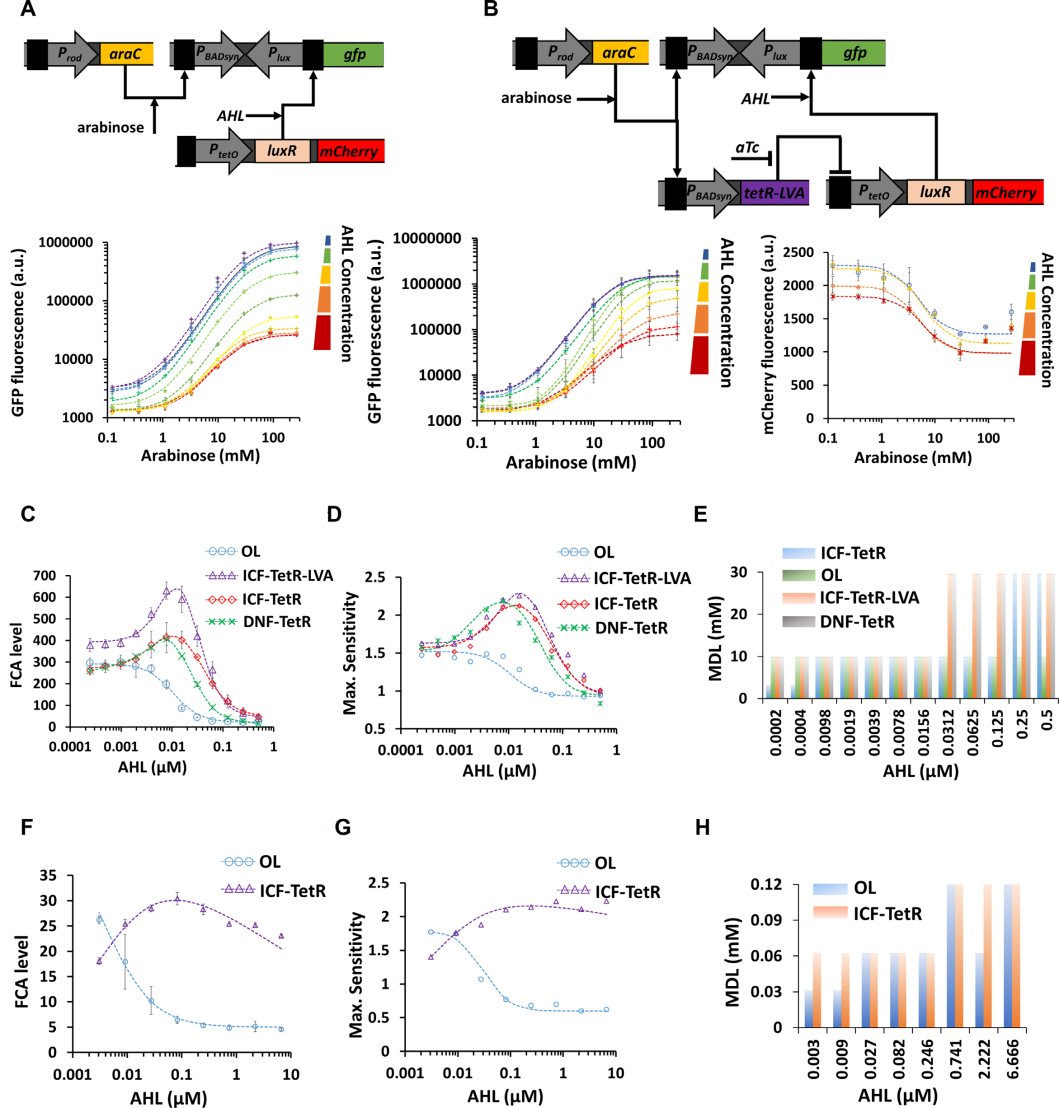
Transcriptional interference- based ICF and DNF topologies for synthetic P_BADsyn_ and P_lacO_ inducible promoters. (**A**) Experimentally measured arabinose-GFP transfer function for the synthetic P_BADsyn_-based OL circuit as a function of AHL concentration. (**B**) Experimentally measured arabinose-GFP and arabinose-mCherry transfer functions for the synthetic P_BADsyn_-based ICF circuit (TetR is fused with a LVA degradation tag) for various AHL concentrations. (**C**–**E**) FCA levels, Maximum sensitivity level and MDL for different synthetic P_BADsyn_-based circuits (OL, ICF, DNF) versus AHL concentrations derived from experimental data. (**F**–**H**) FCA levels, Maximum sensitivity level and MDL for different P_lacO_-based circuits (OL, ICF) versus AHL concentration derived from experimental data provided in Supplementary Information, Section 3.2 (Supplementary Figure S19). The dotted lines are fittings using Hill-functions ($a \cdot \frac{{{{( {AHL/{K_1}} )}^{{n_1}}}}}{{1 + {{( {AHL/{K_1}} )}^{{n_1}}}}} \cdot \frac{1}{{1 + {{( {\frac{{AHL}}{{{K_1}}}} )}^{{n_1}}}}} + b$). All experimental data are averaged from three experiments. The flow cytometry data for this figure is provided in Supplementary Information, Supplementary Figures S25–S38.

According to our simulation results (Figure 5 and Supplementary Figures S12–S16), we tested the l-arabinose regulation system with the new synthetic P_BADsyn_ promoter (without the O_2_ DNA binding site, Figure 4). We constructed the ICF and DNF circuits based on P_BADsyn_ using the transcriptional interference model. In the OL circuit both basal and maximum levels decrease as AHL increases (Figure 6A and Supplementary Figure S17A). In the ICF circuit on the other hand, at a specific value of AHL (7.8 × 10^–3^ μM) the basal level decreases to very low values, while the maximum level only slightly decreases (Figure 6B and Supplementary Figure S17D). Our experimental results also show that TetR acts as an ‘Inverter-logic-gate’ to control the LuxR expression. Other topologies of synthetic gene circuits, such as ICF and DNF containing TetR repressor without the degradation tag, were considered and constructed for optimizing the FCA of P_BADsyn_ (Supplementary Information, Section 3, Supplementary Figure S17). The FCA levels based on the experimental results for the P_BADsyn_ circuit are shown in Figure 6C. All circuits, except for the OL, showed an optimal FCA and maximum sensitivity (Figure 6D) as a function of AHL concentration. We also derived MDL (Figure 6E) from sensitivity values for various circuits (Supplementary Figure S18). At the AHL concentration yielding the highest FCA level, the MDL is very low. In conclusion, for different design topologies an appropriate *F_S_* strength allows FCA level to be improved up to three times (from ON/OFF = 215 to ON/OFF = 630) without compromising the MDL. We also experimentally tested P_lacO_ promoter with an isopropyl β-d-1-thiogalactopyranoside (IPTG) inducer using the transcriptional interference model. The OL and ICF circuits showed consistent behaviors with our mathematical models (Figure 6F–H). Further analysis of the P_lacO_ construct is provided in Supplementary Information, Section 3.2 (Supplementary Figure S19).

**Figure 7.**
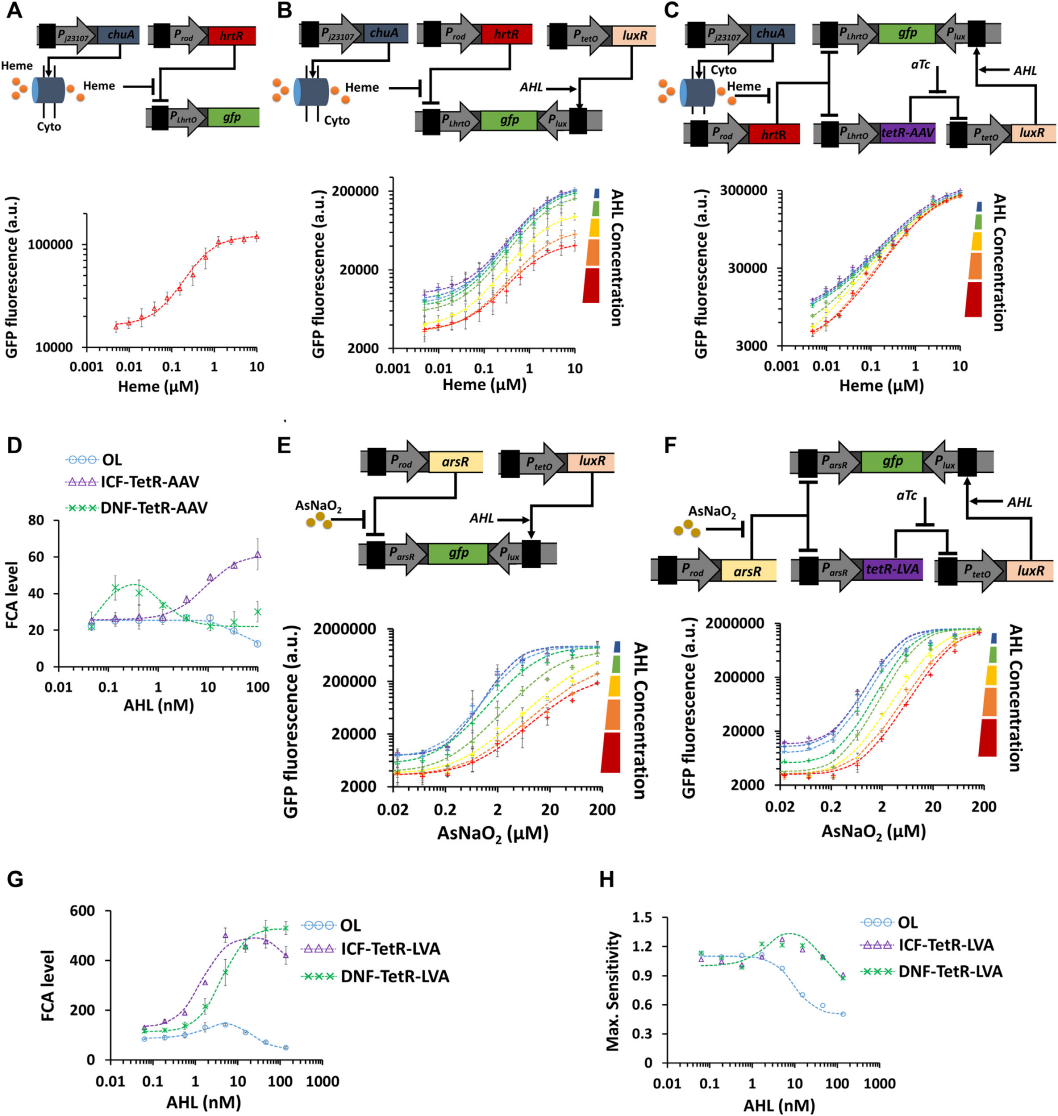
ICF and DNF topologies for specific bacterial biosensors sensitive to heme and arsenic (AsNaO_2_) based on antisense transcription. (**A**) Blood sensor operation. Experimentally measured heme-GFP transfer function of a blood sensing circuit in the simplest (wild-type) design. Transporter proteins are constitutively expressed from *ChuA* gene. HrtR is a repressor and is driven by a constitutive promoter. A heme-group containing molecule enters the bacterial cells through the outer membrane ChuA protein and binds the transcriptional repressor HrtR to form a heme-HrtR complex which is then released from P_LhrtO_ heme-inducible promoter allowing its activation and GFP expression. (**B**) Experimentally measured heme-GFP transfer function of P_LhrtO_-based OL circuit relative to AHL concentration. (**C**) The measured heme-GFP transfer function of P_LhrtO_ based ICF circuit (TetR is fused with a AAV degradation tag) relative to AHL concentration. (**D**) FCA levels derived from experimental results for various blood sensor circuits (OL, ICF, DNF) as a function of AHL concentration. (**E**) Arsenic sensor circuit with inducible antisense transcription. The transcription factor ArsR encoded by *arsR* gene is constitutively expressed to repress P_arsR_ promoter. Arsenic input, AsNaO_2_, can bind with ArsR to release the repression on P_arsR_, to produce a GFP signal. A reverse P_lux_ is located downstream of *gfp* gene to induce antisense transcription. The induction of antisense transcription is controlled by varying AHL concentrations. Experimentally measured arsenic-GFP transfer function of P_arsR_-based OL circuit under various AHL concentrations. (**F**) Experimentally measured arsenic-GFP transfer function of P_arsR_-based ICF circuit (TetR is fused with a LVA degradation tag) under various AHL concentrations. (**G**) and (**H**) FCA levels, Maximum sensitivity derived from experimental results for various arsenic sensor circuits (OL, ICF, DNF) under various AHL concentrations. The dotted lines are fittings using Hill-functions ($a \cdot \frac{{{{( {AHL/{K_1}} )}^{{n_1}}}}}{{1 + {{( {AHL/{K_1}} )}^{{n_1}}}}} \cdot \frac{1}{{1 + {{( {\frac{{AHL}}{{{K_1}}}} )}^{{n_1}}}}} + b$). All experimental data represent the average of three experiments. The flow cytometry data for this figure is provided in Supplementary Information, Supplementary Figures S39-S53.

As an application we used ICF and DNF designs to improve the performance of different types of bacterial biosensors, specifically for detection of heme (48), arsenic (29), hydrogen peroxide (49) and Nalidixic Acid toxins (50). Heme is released from lysed red blood cells, and the presence of this biomolecule in clinical samples is indicative of bleeding (48). The heme biosensor consists of three synthetic parts (Figure 7A and Supplementary Figure S20A); the ChuA protein, the HrtR repressor and the synthetic P_LHrtO_ promoter. ChuA is an outer-membrane transporter from *E. coli* strain O157:H7 that facilitates heme entry across cellular membranes. The HrtR repressor inhibits P_LHrtO_ promoter activity. A Heme-containing molecule binds to HrtR forming the complex Heme-HrtR which is released from P_LHrtO_ promoter allowing its activation with FCA≈7 (Figure 7A and Supplementary Figure S20A). The OL circuit of the heme sensor based on antisense transcription reduced both the basal and maximum levels across the whole AHL range acting as a subtractor (Figure 7B and Supplementary Figure S20B). In contrast, both ICF (Figure 7C and Supplementary Figure S20D) and DNF (Supplementary Figure S20E) circuits designed for heme detection in combination with antisense transcription units reduced the basal level without decreasing the maximum level. At specific AHL concentrations, FCA can be increased to 60 and 40 in ICF and DNF respectively, as shown in Figure 7D. While the DNF circuit reaches an optimal FCA level, the FCA level of the ICF circuit monotonically increases as AHL concentration increases. Further analysis of the heme biosensor is provided in Supplementary Information, Section 3.3 (Supplementary Figure S20).

Arsenic is a heavy metal, which can contaminate drinking water and its long-term exposure can lead to toxicity and health issues including skin diseases and cancer. The Arsenic biosensor has an ArsR repressor and a synthetic promoter (P_arsR_). In the wild-type circuit (Supplementary Figure S21A), ArsR binds Arsenic forming the Arsenic-ArsR complex which is released from P_arsR_ promoter allowing its activation (29). In the OL circuit, increasing AHL concentration decreases both basal and maximum levels (Figure 7E and Supplementary Figure S21B), whereas in ICF (Figure 7F and Supplementary Figure S21D) and DNF (Supplementary Figure S21E) circuits only the basal level is reduced, resulting in maximal FCA≈500 for both circuits. The FCA levels derived from the experimental results for arsenic biosensor are shown in Figure 7G. The ICF circuit shows an optimal FCA. Interestingly, the OL circuit also demonstrates an optimal FCA that is in good agreement with our models (Supplementary Information, Section 2.1, Supplementary Figure S10). Such behavior can be obtained when the reverse promoter P_lux_ affects the switching threshold of the forward promoter. The FCA level of the DNF circuit monotonically increases as a function of AHL concentration. For the ICF, the maximum sensitivity (∼1.3) and maximum FCA level (∼500) were obtained at the same AHL (∼4nM), while for the DNF, the maximum sensitivity and maximum FCA levels were obtained at different AHL concentrations (Figure 7H, AHL∼4nM for maximum sensitivity and AHL∼150nM for maximum FCA). Further analysis for the arsenic biosensor is provided in Supplementary Information, Section 3.4, Supplementary Figure S21. In addition, the behavior of P_arsR_-based ICF circuit remained stable over the course of ∼11 h (Supplementary Figure S21J).

**Figure 8.**
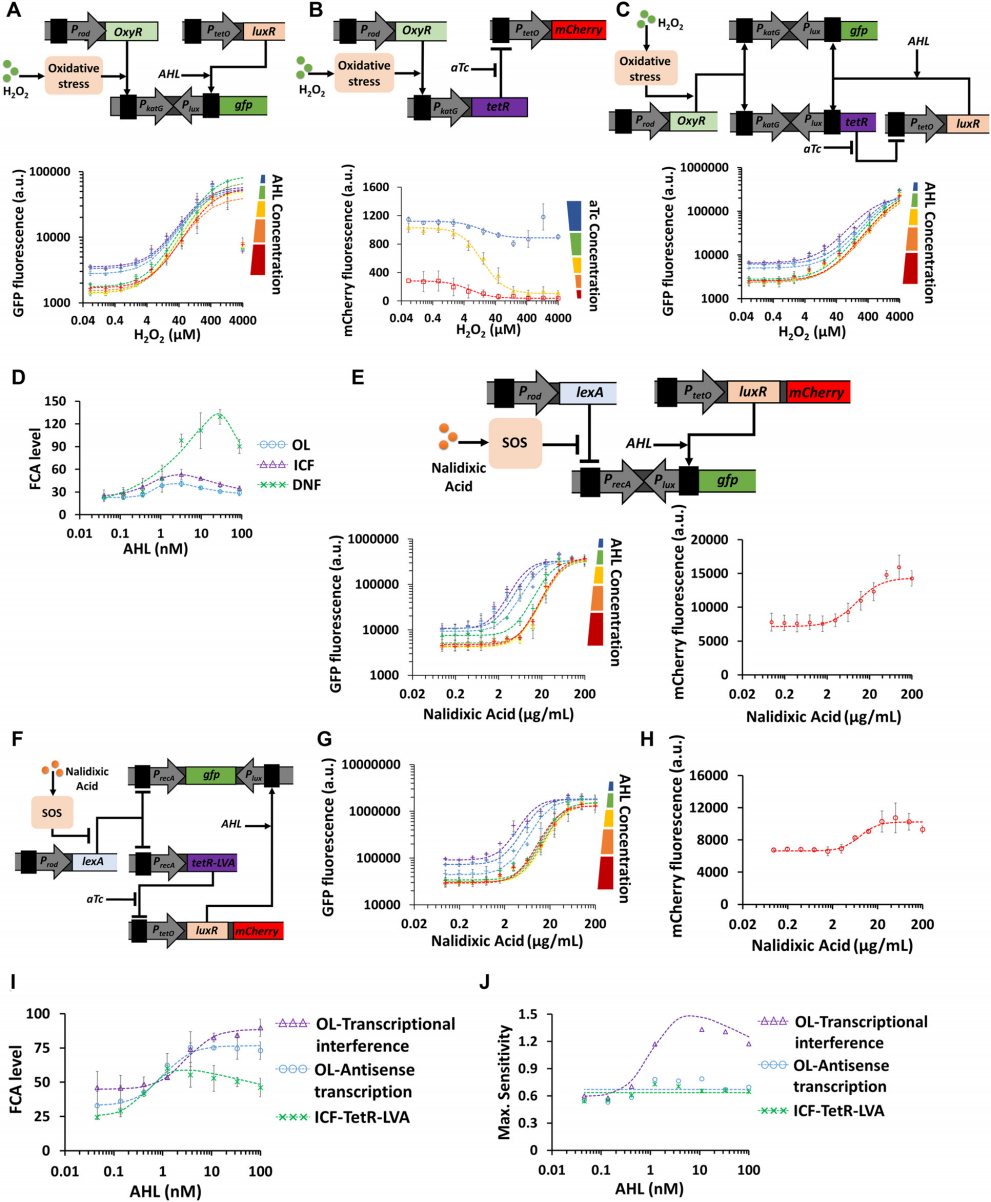
ICF and DNF designs for systemic bacterial biosensors based on oxidative stress response and SOS response. (A) OL circuit based on antisense transcription for the katG biosensor inducible by hydrogen peroxide (H_2_O_2_). H_2_O_2_ interacts with the transcription factor OxyR causing a conformational change to its structure which in turn activates through a series of oxidative stress responses the P_katG_ promoter enabling GFP expression. OxyR is constitutively expressed. A reverse promoter, P_lux_, is placed downstream of P_katG_ as a transcriptional interference component, the strength of which can be programmed using AHL concentration. The experimentally measured H_2_O_2_-GFP transfer function of P_katG_-based OL circuit under various AHL concentrations is also shown. (B) Experimentally measured H_2_O_2_-mCherry transfer function of the inverting switch using TetR repressor. The P_katG_ promoter controls the expression of TetR, which represses the activity of P_tetO_. The small molecule, aTc, inhibits the activity of TetR. (C) Experimentally measured H_2_O_2_-GFP transfer function of the P_katG_-based DNF circuit under various AHL concentrations. (D) FCA levels derived from experimental results for different katG biosensor circuits (OL, ICF, DNF) across AHL concentrations. (E) OL circuit based on transcriptional interference for recA biosensor activated by Nalidixic Acid. The LexA repressor inhibits the activity of P_recA_ promoter, and Nalidixic Acid induces a series of SOS responses that inhibit the LexA activity. P_recA_ drives GFP expression and LexA is constitutively expressed. A reverse promoter, P_lux_, is placed downstream of P_recA_ as a transcriptional interference component, the strength of which can be programmed using AHL concentration. The experimentally measured Nalidixic Acid-GFP transfer function of P_recA_-based OL circuit under various AHL concentrations, as well as the Nalidixic Acid-mCherry transfer function of P_tetO_ in the absence of TetR are also shown. (F) Experimentally measured Nalidixic Acid -GFP transfer function of P_recA_-based ICF circuit (TetR is fused with a LVA degradation tag) using antisense transcription under AHL concentrations. The measured Nalidixic Acid -mCherry transfer function of P_recA_-based ICF circuit is shown. (G) and (H) show FCA levels, Maximum sensitivity derived from experimental results for the OL and ICF circuits of recA biosensor across AHL concentrations. The dotted lines are fittings using Hill-functions ($a \cdot \frac{{{{( {AHL/{K_1}} )}^{{n_1}}}}}{{1 + {{( {AHL/{K_1}} )}^{{n_1}}}}} \cdot \frac{1}{{1 + {{( {\frac{{AHL}}{{{K_1}}}} )}^{{n_1}}}}} + b$). All experimental data represent the average of three experiments. The flow cytometry data for this figure is provided in Supplementary Information, Supplementary Figures S54–S63.

So far, we have applied the proposed designs (OL, ICF and DNF) in biological systems where chemical inputs directly interact with target promoters by binding transcription factors. However, biological systems often activate multiple pathways in response to chemical signals and more specifically in response to toxic chemicals. For example, cells induce repair systems by activating cascades of regulators, e.g. oxidative stress response (49) and SOS response (50). Implementing the ICF and DNF designs in these complex biological systems can be challenging from a circuit design aspect. To further explore the applicability of our designs, we first examined the oxidative stress response that is sensitive to hydrogen peroxide (H_2_O_2_). The transcriptional regulator, OxyR, is activated by oxidation by H_2_O_2_ which in turn activates several genes involved in bacterial defense mechanisms, among them is the *KatG* gene (51). In the wild-type circuit, the transcriptional activator OxyR binds the katG promoter (P_katG_) allowing its activation with FCA level of 10 (Supplementary Figure S22A). We implemented the OL circuit based on transcriptional interference as shown in Figure 8A and Supplementary Figure S22B. The OL circuit reduced the basal and maximum levels across the entire AHL range with an increase in the switching threshold. Our experimental results also show that TetR acts as an ‘Inverter-logic-gate’ to control LuxR expression level (Figure 8B and Supplementary Figure S22C). At a specific AHL concentration, the DNF circuit reduced P_katG_ basal level without affecting the maximum levels (Figure 8C and Supplementary Figure S22D). The FCA levels under various AHL concentrations are derived from the experimental results for katG biosensors and are shown in Figure 8D. For OL, ICF and DNF circuits, FCA reaches optimal values when AHL concentrations fall between 1 and 100 nM, with increasing MDL (Supplementary Figure S22). Further analysis of the katG biosensors is provided in Supplementary Information, Section 3.5.

The SOS response in cells is induced by the DNA damage repair process and involves the activation of more than 40 genes, including *recA* gene and its transcriptional repressor LexA (50,52). To build bacterial biosensors that are sensitive to SOS response, first, a synthetic circuit that includes recA promoter (P_recA_) and LexA repressor was transformed into bacterial cells. The circuit is induced by Nalidixic Acid toxin and demonstrated a FCA level of 50 (Supplementary Figure S23A). In such circuit, there is no direct interaction between Nalidixic Acid and LexA. Then, we applied the OL design with recA promoter using both transcriptional interference (Figure 8E and Supplementary Figure S23C) and antisense transcription (Supplementary Figure S23B). The OL designs reduced the basal and maximum levels of P_recA_ across the entire range of AHL concentration and increased the switching threshold. In the case of P_recA_ promoter, the activity of P_tetO_ promoter in the absence of TetR (P_tetO_ is effectively a constitutive promoter) exhibits a strong dependency on Nalidixic Acid concentrations (mCherry signal in Figure 8E, Supplementary Figure S23C, and in Supplementary Figure S23B). Such a dependency has not been observed with other inducers applied to P_BADsyn_, P_lacO_, P_LhrtO_, P_arsR_ and P_KatG_. The dependency of P_tetO_ activity on Nalidixic Acid also affected the behavior of the ICF circuit (Figure 8F and Supplementary Figure S23E). For example, P_tetO_ is supposed to act as an inverter gate where the mCherry signal decreases as a function of Nalidixic Acid, instead, the mCherry signal increases when the Nalidixic Acid concentration is above 2 ug/ml. This trend in P_tetO_ activity affects the optimal FCA level of the circuits. The FCA and maximum sensitivity levels derived from the experimental results for the recA biosensors are shown in Figure 8G and H, respectively. The OL based on transcriptional interference design performs best among the three designs, showing a doubled FCA level and maximum sensitivity. Further analysis of the recA biosensor is provided in Supplementary Information, Section 3.6 (Supplementary Figure S23).

## DISCUSSION

Here, we demonstrated that synthetic gene regulatory circuits can be engineered to improve the fold change activation (FCA) of a target promoter by reducing its basal level while maintaining its maximum activity at high inducer levels. Two circuit designs were implemented, first is an indirect coherent feedforward (ICF) circuit, which is known as a coherent feedforward type 4 system, and second is a mutual inhibition by double negative feedback loop (DNF). Both designs are common motifs in natural regulatory networks. It has been reported that the ICF design can reject disruptions in indirect pathways or create delays in gene expression (36). The DNF circuit is useful for creating bistable switches coupled with ultrasensitivity (17). Recent study has shown that DNF circuit can significantly reduce the basal level. The circuit also affected the maximum level without gaining an improvement for the FCA (35). In this study, we showed that the ICF network naturally occurs in the L-arabinose regulation system (Figure 4A).

Here, we started with simple linear models, which allowed us to study the behavior of the ICF and DNF circuits when the strength of feedback/feedforward loop is varied. Based on these models, we verified that such designs could improve the FCA level of the circuit/part under test. Next, by replacing the linear operations with non-linear equations such as Hill-function and repression equations, we developed molecular models for the ICF and DNF circuits including three nodes. The molecular models showed that the ICF design has an advantage over the DNF design. While the DNF circuit showed an optimum in the FCA behavior, the FCA of ICF circuit monotonically increases with the loop strength. Finally, we developed genetic models for the ICF and DNF networks using four nodes. These genetic models show that for both designs an optimum can be achieved when the loop strength is varied (Supplementary Figures S12 and S15). These simulation results were used to guide circuit construction of the ICF and DNF gene networks. For example, we chose to implement the subtraction using an inducible biological part (LuxR/P_lux_) which allows us to easily tune the loop strength with AHL concentration. Experimentally, we could not find any advantage of applying the ICF over DNF. However, we prefer using the ICF design, since it has fewer regulatory elements and is predicted to achieve better performance in wide range of biological contexts.

We selected two biological mechanisms to implement subtraction. In both, a reverse promoter is oriented in the opposite direction of promoter under test (also called the forward promoter). The first was transcriptional interference, where the reverse promoter is located directly downstream adjacent to the promoter under test and upstream of the reporter gene. The second is antisense transcription, where the reverse promoter is located downstream of the reporter gene. Although the antisense transcription involves possible interruptions between RNAP of forward promoter and RNAP of reverse promoter, here we assumed that the main mechanism for repression is the binding of antisense RNA to the sense mRNA to block translation. Although experimentally we found that both mechanisms have simple constructs and yield similar results in terms of FCA, sensitivity and cell growth (e.g. toxicity), we suggest using antisense transcription because it has been demonstrated to function in a wide range of biological contexts (e.g. cell types). Other biological mechanisms can also be used for implementing a subtraction, for example, by embedding the LuxR binding site inside the target promoter that drives the *gfp* gene. However, such promoters, known as combinatorial promoters (53), require re-engineering, which can be challenging in some applications, and they also affect the binding affinity of the RNA polymerase.

In this study, we successfully applied the ICF and DNF designs to six different promoters starting with synthetic promoters, such as arabinose/P_BADsyn_ and IPTG/P_lacO_, as a proof-of-principle. Then we examined native promoters that are either functionally specific or systemically involved in complex pathways in living cells such as oxidative stress and the SOS response. Improved FCA levels were achieved in all six promoters, with improvement varying from three to ten times. Furthermore, using the proposed methodology, we increased the sensitivity of the target promoters. The minimum detection limit for some of these promoters was increased. Notably, the open loop design for the P_lacO_ and P_katG_ promoters, where the reverse promoter controls GFP only, showed an optimal behavior. This is because the reverse promoter can both reduce the activities of P_lacO_ and P_katG_ and increase their switching thresholds, in contrast to other promoters where the reverse promoter acts as a pure subtraction and reduce the expression level only. In addition, the highest FCA level of the recA promoter was obtained using the open loop design due to the sensitivity of the P_tetO_ promoter to Nalidixic Acid.

In real-world biosensors, it is challenging to optimize biosensors employing this technique without using additional inducers such as AHL and aTc. To overcome this limitation, we built a new strain that constitutively produced AHL (N_1_) and mixed it with a strain containing an ICF circuit (N_2_) in different ratios (Supplementary Figure S21I). The ratio between the two strains determines the effective AHL concentration that each receiver cell obtains. To optimize the FCA level, we adjusted the ratio N_1_/N_2_ to be 0.033 (Supplementary Figure S21I). The TetR activity can be optimized by selecting an optimal protein degradation system (54) without the need to use an additional inducer as aTc.

Improving the performance of bacterial biosensors (e.g. recA, katG, hrtR and arsR promoters) by increasing the FCA, i.e. ON/OFF ratio (or the output dynamic range), and sensitivity has long remained an important goal of synthetic biology. For example, it has been shown that FCA can be increased by tuning the ribosome-binding sequences (34) or fusing the reporter protein with a ssrA degradation tag driven by an arsR promoter (29). Cascaded signal amplifiers have also been implemented in bacterial biosensors to increase circuit sensitivity and output dynamic range (29,39,55). Such methods can simply be combined with our methodology (ICF, DNF designs) to achieve even higher FCA levels. In this study, we have shown that the ICF and DNF network motifs can be applied to several biological systems. Therefore, we expect these motifs to be general, versatile, and adaptable to other biological systems e.g., cell free genetically encoded sensors (56), and RNA components (57). This flexibility can help address technological challenges for a wide range of industrial, diagnostic, and therapeutic applications such as reducing the leaky expression of toxic proteins, and may improve safety of genetic circuits for cancer immunotherapy (58).

## DATA AVAILABILITY

The Flow Cytometry experiments generated during the current study are available at the FlowRepository with Repository ID: FR-FCM-Z3DB.

## Supplementary Material

gkab253_Supplemental_File
