# Supplementary material for: Topologies of synthetic gene circuit for optimal fold change activation
Source: Nucleic Acids Res. 2021 May 1;49(9):5393–406. doi: 10.1093/nar/gkab253 (PMC8136830; doi:10.1093/nar/gkab253)
Supplement: gkab253_Supplemental_File [file gkab253_supplemental_file.pdf]

Supplementary Information for:

Topologies of synthetic gene circuit for optimal fold change activation

List of Contents

1. Simplified models with linear operations........ 2
2. Models and simulations........ 5
   2.1 Biological models for transcriptional interference and antisense transcription........ 5
   2.2 Models for ICF design........ 12
      2.2.1 Molecular ICF three-nodes model........ 12
      2.2.2 Genetic ICF four-nodes model........ 14
   2.3 Models for DNF design........ 16
      2.3.1 Molecular DNF three-nodes model........ 16
      2.3.2 Genetic DNF four-nodes model........ 18
3. Experimental results for the 6 promoters........ 20
   3.1 The synthetic promoter P_{BADsyn}........ 20
   3.2 The synthetic promoter P_{lacO}........ 23
   3.3 The native specific promoter P_{LhrtO}........ 25
   3.4 The native specific promoter P_{arsR}........ 28
   3.5 The native stress promoter P_{katG}........ 30
   3.6 The native stress promoter P_{recA}........ 33
4. Experimental data from Flow cytometry........ 35
5 Plasmid maps used for this work (Table S1)........ 81
6 Synthetic parts used for this work (Tables S2, S3 and S4)........ 88
References........ 94

1. Simplified models with linear operations:

In this section, we build linear models of three circuit topologies that can control their fold change:

(1) Open loop (OL) circuit
(2) Indirect Coherent feedforward (ICF) circuit.
(3) Mutual inhibition by double negative feedback (DNF) circuit

First, we describe the part under test (PUT) in its prototype topology, as shown in Fig. S1. The circuit performs a non-linear monotonic function with two distinct levels: (1) a minimum normalized level "β" (e.g., basal level of promoter) and (2) a maximum normalized level "1". Such a function can be described by:

$$Out = (1-\beta)\cdot f(In) + \beta \quad \text{(S1.1)}$$

And the fold change detection FCA level is given by:

$$FCA = 1-\beta \quad \text{(S1.2)}$$

Where *f(In)* is also non-linearly monotonic with a basal level equal to zero, and a maximum level equal to one. The *f(In)* satisfies:

$$f_{min}(In = 0) = 0 \quad \text{(S2.1)}$$

$$f_{max}(In = In_{max}) = 1 \quad \text{(S2.2)}$$

$$FCA = 1 \quad \text{(S2.3)}$$

Sensitivity measures the fold change in the output regarding the fold change in the input, and is given by:

$$S = \frac{\Delta Out/Out}{\Delta In/In} \quad \text{(S3)}$$

By substituting Eq. S1.1 into Eq. S3, we get the sensitivity of part under test as:

$$\frac{\Delta Out}{Out} = \frac{(1-\beta)\cdot df(In)}{(1-\beta)\cdot f(In)+\beta} \quad \text{(S4.1)}$$

$$S = \frac{S_f}{1+\frac{\beta}{(1-\beta)\cdot f(In)}} \quad \text{(S4.2)}$$

Where $S_f = \frac{df(In)/f(In)}{dIn/In}$ is the sensitivity of the function *f(In)*. For the open loop circuit (Fig. S1B), the output is reduced by a constitutive value (F_S), and is given by:

$$Out = (1-\beta)\cdot f(In) + \beta - F_S \quad \text{(S5.1)}$$

Since the basal and maximum levels are reduced by the same amount, we do not expect for an improvement in the FCA level:

$$Out_{max} = 1 - F_S \quad \text{(S5.2)}$$

$$Out_{min} = \beta - F_S \quad \text{(S5.3)}$$

$$FCA_f = 1-\beta \quad \text{(S5.4)}$$

$$S = \frac{S_f}{1+\frac{\beta - F_S}{(1-\beta)\cdot f(In)}} \tag{S5.5}$$

Our analysis over the three circuits is presented with normalizations, with $0<\beta<1$, $0<F_S<1$.

The indirect coherent feedforward (ICF) circuit is shown in Fig. S1C. The signal of the part under test splits to two branches, the sum of which positively regulate the final output. While in the first branch the signal directly controls the final output, the second branch involves two inhibition nodes to regulate the final output. The ICF circuit output is given by:

$$Out = (1-\beta)\cdot f(In) + \beta - F_S \cdot [1-(1-\beta)\cdot f(In) - \beta] \tag{S6.1}$$

$$Out_{max} = 1 \tag{S6.2}$$

$$Out_{min} = \beta - F_S \cdot (1-\beta) \tag{S6.3}$$

$$FCA = 1 - \beta + F_S \cdot (1-\beta) \tag{S6.4}$$

$$S = \frac{S_f}{1+\frac{\beta - F_S + F_S\cdot\beta}{(1-\beta)\cdot(1+F_S)\cdot f(In)}} \tag{S6.5}$$

From Eq. S6.4 and Eq. S6.5, we obtain the maximum value of *FCA* and maximum value of sensitivity when:

$$F_S = \frac{\beta}{1-\beta} \quad \Rightarrow \quad FCA_{max} = 1, \;\; S = S_f \tag{S7}$$

By selecting a specific level of F_S (Eq. S7), we can enhance the FCA level using the ICF design.

The double negative feedback loop (DNF) circuit is shown in Fig. S1D. The signal of the part under test is regulated by a cascade of an indirect positive feedback and an inhibition with a negative signal. The circuit output is given:

$$Out = (1-\beta)\cdot f(In) + \beta - F_S \cdot (1-out) \tag{S8.1}$$

$$\Rightarrow \quad Out = \frac{(1-\beta)\cdot f(In) + \beta - F_S}{1-F_S}$$

$$Out_{max} = 1 \tag{S8.2}$$

$$Out_{min} = \frac{\beta - F_S}{1-F_S} \tag{S8.3}$$

$$FCA = \frac{1-\beta}{1-F_S} \tag{S8.4}$$

$$S = \frac{S_f}{1+\frac{\beta - F_S}{(1-\beta)\cdot f(In)}} \tag{S8.5}$$

From Eq. S8.3 and Eq. S8.5, we obtain maximum value of *FCA* and maximum value of sensitivity when:

$$F_S = \beta \quad \Rightarrow \quad FCA_{max} = 1, \;\; S = S_f \tag{S9}$$

By selecting a specific level of F_S (Eq. S9), we can enhance the FCA level using the DNF design.

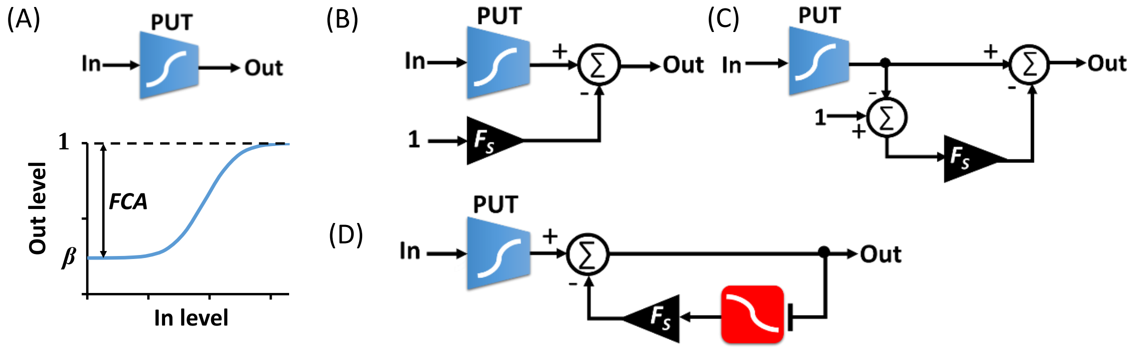

Fig. S1. (A) Transfer function of part under test (PUT) in its prototype design. (B) Open loop (OL) design for basal level reduction. (C) Indirect coherent feedforward (ICF) design for basal level reduction. (D) Double negative feedback (DNF) design for basal level reduction

Our simulation results including the input-output transfer function and sensitivity for the three circuits are shown in Fig. S2. In these simulations, we assumed that $f(I_n)$ is a sigmoid function that is shifted in *x-axis*:

$$f(In) = \frac{e^{(x-x_0)}}{1+e^{(x-x_0)}} \quad \text{(S10)}$$

Since biological signals cannot be negative, only non-negative outputs are allowed for the three designs. All the negative values were set to zero. The sensitivity was calculated using Eq. S3. As shown in our simulation results (Fig. S3A), the open loop design cannot increase the *FCA*, while the DNF loop can reach maximum of FCA=1 and reduce the basal level to zero when $F_S=\beta$. Similar behavior can be obtained for the ICF design, when $F_s=\beta/(1-\beta)$. We define minimum detection level (MDL) as the input value when the sensitivity is maximum (Fig. S3C). Interestingly, the three circuits show the same characteristics of minimum detection level (MDL) as a function of F_s (Fig. S3B). Our result show that MDL has optimum at different F_s value for each circuit. For ICF circuit, minimal MDL is obtained at $F_s=\beta/(1-\beta)$. For DNF circuit, minimal MDL is at $F_S=\beta$. In both cases, the minimum MDL values are the same. The MDL is proportional to the limit of detection corresponding to the lowest concentration of a target input that is detectable.

As shown in simulation results, there is a strong dependency between the basal level and the F_s strength. This dependency can guide us to search for the β and F_S values to reach an optimal performance (Maximum *FCA* and Minimum MDL). Therefore, we graphed β–F_S diagrams to show qualitatively how relations between β and F_S affect FCA and MDL in ICF and DNF circuits (Fig. S4A-4B). For comparison, we also combined the graphs for both circuits (Fig. S4C).

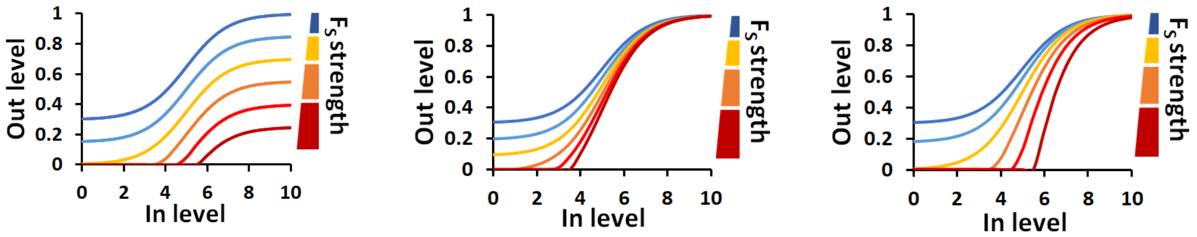

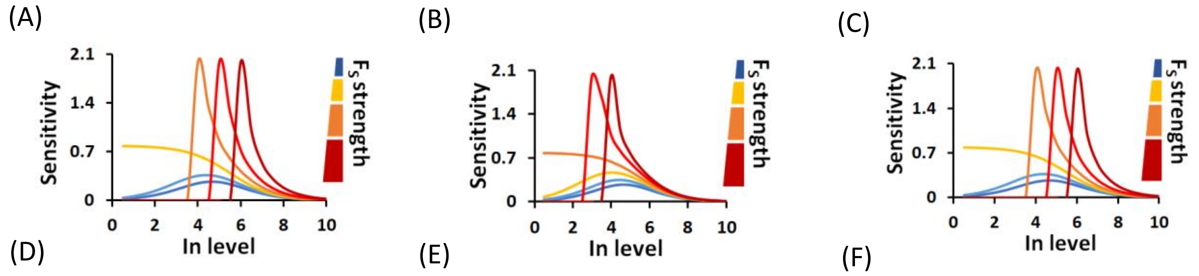

Fig. S2. (A)-(C) simulation results for input-output transfer functions for the open loop (OL), indirect coherent feedforward (ICF) and double negative feedback (DNF) designs. (D)-(F) Sensitivity results for OL, ICF and DNF models based on Eq. S3.

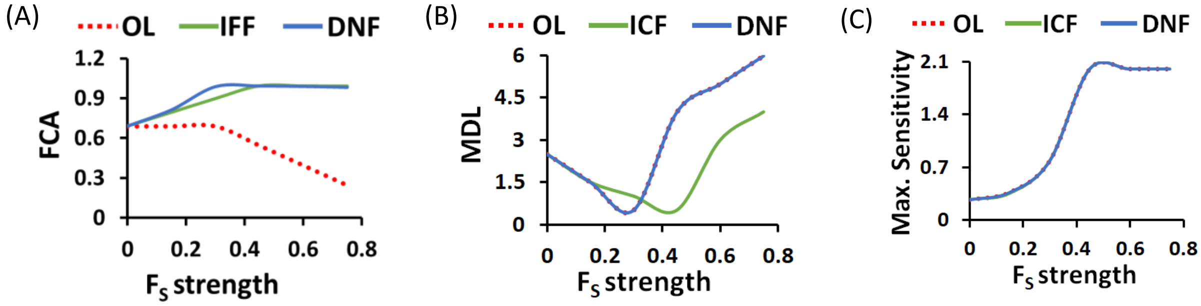

Fig. S3. Simulation results of (A) Fold change activation (*FCA*) versus F_s strength, (B) Minimum detection level versus F_s strength. Minimum detection level or MDL is defined as the input value when the sensitivity is maximum, and (C) Maximum sensitivity versus F_s strength. These results were estimated based on the simulation results of Fig. S2.

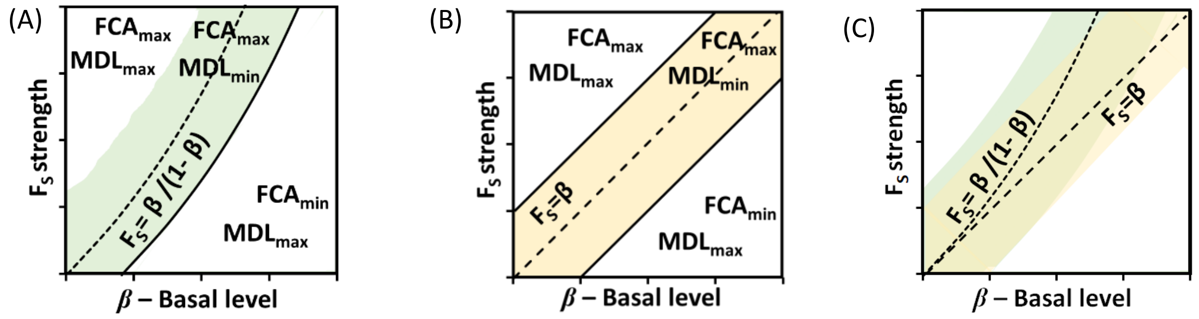

Fig. S4. Qualitative β–F_S diagrams for (A) indirect coherent feedforward circuit (ICF), (B) double negative feedback loop (DNF) and (C) Both circuits.

2. Models and simulations

2.1. Biological models for transcriptional interference and antisense transcription:

Here, we implement the subtraction operation in Fig. S5 using two biological models:

(1) Transcriptional interference (Fig. S5A). We utilize two competitive promoters (P_{Forw} vs P_{Rev}) that located in opposite orientation to each other, where P_{Rev} impede the transcriptional activity of P_{Forw}. The P_{Rev}promoter is located upstream to the target gene. This special organization allows interference between the forward and reverse promoters to arise due to RNA polymerase (RNAP) collisions. The promoter under test (PUT) is used as P_{Forw}.

(2) Antisense transcription (Fig. S5B). We utilize two competitive promoters ($\mathrm{P_{Forw}}$ vs $\mathrm{P_{Rev}}$) that located in opposite orientation to each other, where $\mathrm{P_{Rev}}$ produces antisense mRNAs to block the translation of mRNA transcribed from P_{Forw}. The $\mathrm{P_{Rev}}$ promoter is located downstream to the target gene. This special organization allows hybridization of complementary mRNAs from the forward and reverse promoters and thus down-regulation of promoter translational activities. The promoter under test (PUT) is used as $\mathrm{P_{Forw}}$.

Although a detailed biophysical model was developed (Brophy & Voigt, 2016) for modeling such complex structure of promoters, here we took a simpler approach. The purpose of our models is to capture the general behavior of systems and examine how they can be incorporated into the ICF and DNF circuits. Regarding the transcriptional interference in Fig. 5SA, because the two opposite promoters are located close to each other, we treat them as one system modeled using statistical thermodynamics (Fig. S6). The binding of RNA polymerases recruited by TFs to promoters is modeled according to the Shea-Ackers formalism (Ackers et al., 1982; Bintu et al., 2005). The model describes five different states: (1) Both promoters are unbound; (2) RNA polymerases (RNAP) without transcription factors are bound on both promoters and cause a basal level activity. (3) The complex activator (X)-RNAP is bound to the forward promoter, activating the output signal. (4) The complex activator (Y)-RNAP is bound to the reverse promoter, repressing the output signal. (5) The complex activator (X)-RNAP is bound to the forward promoter, and the complex activator (Y)-RNAP is bound to the reverse promoter. In our model we also assume that the collision interference is large and thus the probability that the forward and the reverse RNA polymerases can simultaneously bind to the DNA is very low ($\theta << 1$). The level of gene expression is proportional to the probability (P) that RNA polymerase is bound to the forward promoter at the equilibrium, and is given by:

$$P = \frac{\left(\frac{X}{K_{df}}\right)^{n_f} + \beta}{1 + \left(\frac{X}{K_{df}}\right)^{n_f} + \left(\frac{Y}{K_{dr}}\right)^{n_r} + \beta} \qquad \text{(S11)}$$

Where X and Y are the concentrations of transcription factors which bind to forward and reverse promoters, respectively. n_f and n_r are Hill-coefficients, K_{df} and K_{dr} are dissociation constants of transcription factors binding to forward and reverse promoters, respectively. Based on Eq. S11, the maximum activity of promoter is 1, however, the collision interference can also reduce the maximum expression level, and as a result, we can modify Eq. S11:

$$P = \frac{\left(\frac{X}{K_{df}}\right)^{n_f} + \beta}{1 + \left(\frac{X}{K_{df}}\right)^{n_f} + \rho_1 \cdot \left(\frac{Y}{K_{dr}}\right)^{n_r} + \beta} \cdot \left(\frac{1}{1 + \rho_2 \cdot \left(\frac{Y}{K_{dr}}\right)^{n_r}}\right) \qquad \text{(S12)}$$

where ρ_1 *and* ρ_2 are parameters representing the strength of two processes as Y level increases: (1) shift of switching threshold with coefficient ρ_1 and (2) repression in the expression level with coefficient ρ_2. The kinetics of gene expression based on the promoter activity in Eq. S12 is:

$$\frac{dZ}{zt} = \alpha \cdot \frac{\left(\frac{X}{K_{df}}\right)^{n_f} + \beta}{1 + \left(\frac{X}{K_{df}}\right)^{n_f} + \rho_1 \cdot \left(\frac{Y}{K_{dr}}\right)^{n_r} + \beta} \cdot \left(\frac{1}{1 + \rho_2 \cdot \left(\frac{Y}{K_{dr}}\right)^{n_r}}\right) - \frac{Z}{\tau_z} + P_{min} \qquad \text{(S13)}$$

At the steady state we get:

$$Z = Z_{max} \cdot \frac{\left(\frac{X}{K_{df}}\right)^{n_f} + \beta}{1+\left(\frac{X}{K_{df}}\right)^{n_f} + \rho_1 \cdot \left(\frac{Y}{K_{dr}}\right)^{n_r} + \beta} \cdot \left(\frac{1}{1+\rho_2 \cdot \left(\frac{Y}{K_{dr}}\right)^{n_r}}\right) + Z_{min} \quad \text{(S14)}$$

where Z_{max} is the maximum protein level, and Z_{min} is the minimum protein level. The Z_{min} level is different than the basal level. From Eq. S14, we can see that the basal level of the forward promoter is achieved when X=0 and is equal to ($\frac{\beta}{1+\rho_1 \cdot \left(\frac{Y}{K_{dr}}\right)^{n_r}}$) which is affected by β and Y, while the Z_{min} is not regulated. For example, Z_{min} can be a result of autofluorescence.

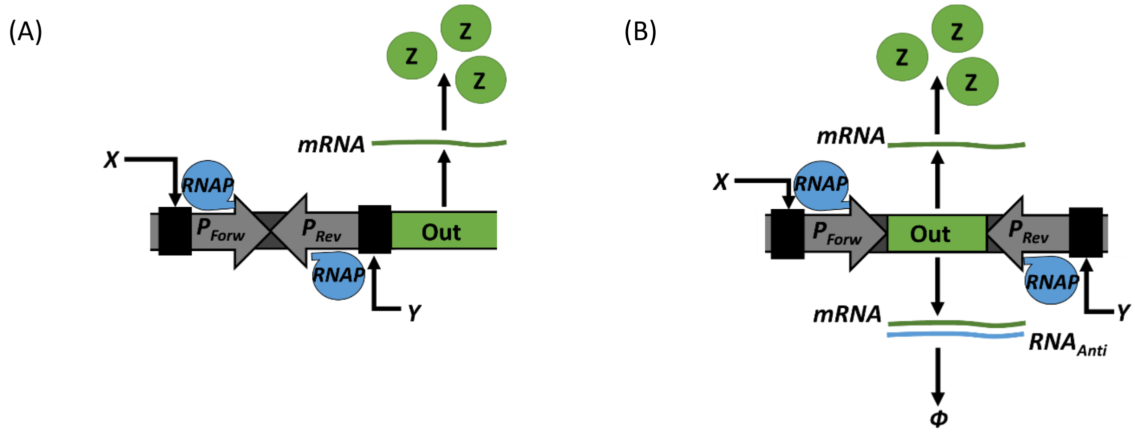

Fig. S5. Implementation of subtraction: (A) A schematics of genetic circuit based on transcriptional interference. (B)A schematics of genetic circuit based on antisense transcription.

Simulation results of the transcriptional interference model in Eq. S14 are shown in Fig. S7. The ρ_1 *and* ρ_2 parameters have a significant impact on the results. As explained previously, ρ_1 shifts the transfer function of Z relative to X rightward, increasing the effective dissociation constant (K_{df} is equal to the effective dissociation constant when $Y/K_{dr}<<1$). ρ_2 decreases the effective maximum Z level through the coefficient $\left(\frac{1}{1+\rho_2 \cdot \left(\frac{Y}{K_d}\right)^{n_r}}\right)$. Interestingly, our simulation results show that when ρ_1=0, $\rho_2=1$, the transcriptional interference behaves as a pure subtraction with respect to Y level (Fig. S7A and Fig. S7E have similar behaviors as in Fig. S2A). In this case, the FCA is either a constant without dependency on Y level for $Z_{min}=0$ (Fig. S8A) or decreases as Y increases for $Z_{min}=20$ (Fig. S8B). Here Z_{min} affects the FCA in the same way as in the linear model where all the negative values were set to zero, because biological signal cannot be negative. Another interesting case is when ρ_1=1, $\rho_2=0$ (Fig. S7F), specifically, FCA level increases even when the Z_{min} is high (Fig. S8B). This is because when $\rho_2=0$, there is no reduction to Z expression level even when the input is very high. An increase in Y can result in threshold shift and thereby enhance circuit FCA, but when it represses the gene expression level, a reduction of FCA can be observed. Consequently, an optimal level of FCA can be obtained at specific Y level (Fig. S8B). The main conclusions from our simulation results are as follows:

1. Transcriptional interference model can behave as a pure subtraction when there is only a repression component due to the enhanced activity of the reverse promoter (Y level), without any effect on the threshold of the complex promoter, that is, ρ_1=0, $\rho_2=1$ (Top curves in Fig. S8A and S8B).

2. The FCA of promoters based on transcriptional interference can be improved by enhancing the activity of reverse promoter (Y level), when the reverse promoter affects the threshold of the complex promoter, that is, $\rho_1 \neq 0$, $\rho_2=1$.

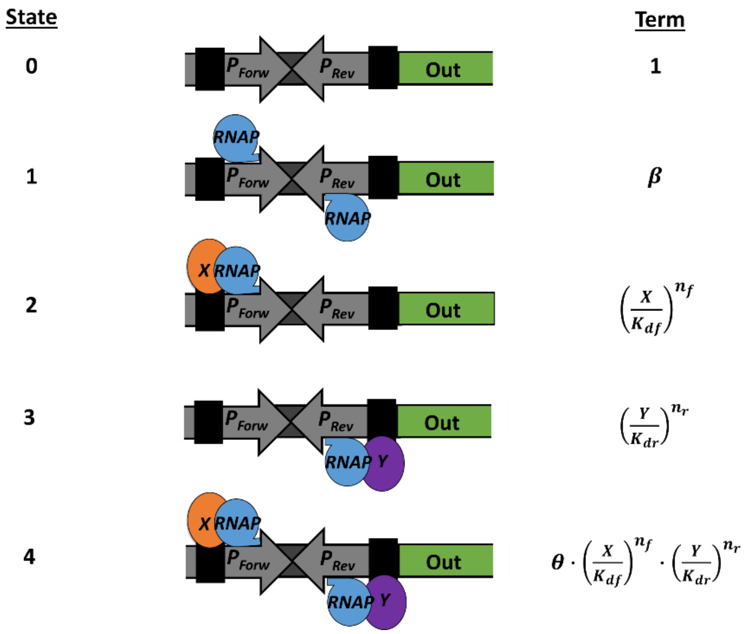

Fig. S6. The binding states of the forward promoter and the transcriptional interference by the reverse promoter.

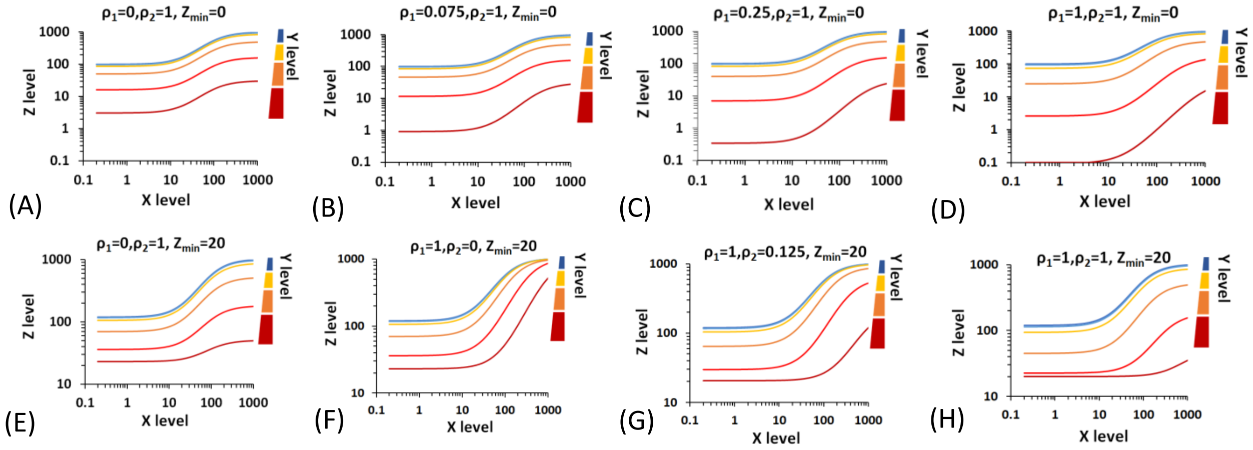

Fig. S7. Simulation results of transcriptional interference model (Eq. S14) as function of X levels (activity of forward promoter) and Y levels (activity of reverse promoter). Simulation parameters: $n_f=1.5$, $n_r=1.5$, $K_{df}=100$, $K_{dr}=100$, $\beta=0.1$, $Z_{max}=1000$.

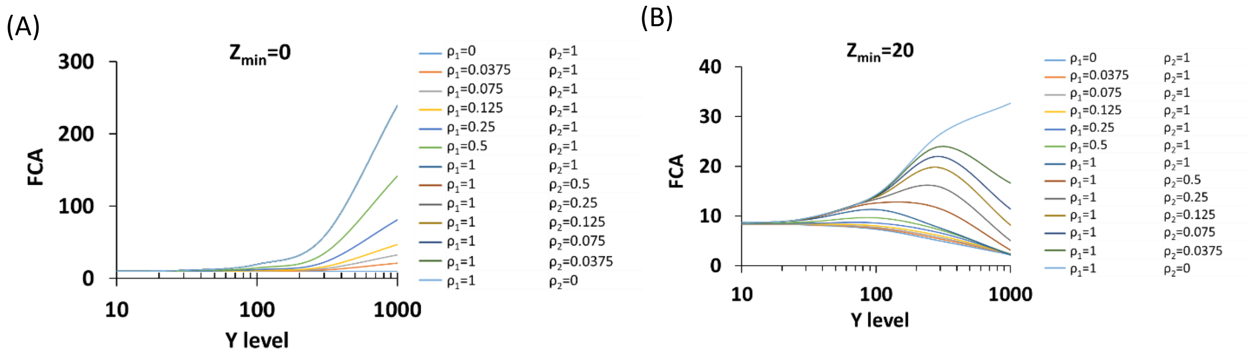

Fig. S8. Fold change activation (FCA) level of transcriptional interference model versus the activity of reverse promoter (*Y* level), based on results Fig. S7, (A) when $Z_{min}=0$, (B) when $Z_{min}=20$.

In this work, the biophysical model of antisense transcription is based on the biochemical binding reaction occurring between mRNA and antisense RNA (Fig. S5B), without considering the RNA polymerase (RNAP) collisions:

$$mRNA + RNA_{Anti} \overset{K_m}{\Leftrightarrow} Complex \quad \text{(S15)}$$

where mRNA is the level of mRNA produced from the forward promoter, and RNA_{Anti} is the level of antisense RNA produced from the reverse promoter, and K_m is the dissociation constant of the reaction. At steady state, we obtain:

$$Complex = \frac{mRNA \cdot RNA_{Anti}}{K_m} \quad \text{(S16.1)}$$

$$\frac{dmRNA}{dt} = -\frac{dComplex}{dt} \quad \rightarrow mRNA = mRNA_T - Complex \quad \text{(S16.2)}$$

$$\frac{dRNA_{Anti}}{dt} = -\frac{dComplex}{dt} \quad \rightarrow RNA_{Anti} = RNA_{Anti\_T} - Complex \quad \text{(S16.3)}$$

Where $mRNA_T$ and RNA_{Anti_T} are the total level of mRNA and antisense RNA. Substituting Eq. S16.2, Eq. S16.3 into Eq. S16.1:

$$Complex = \frac{(mRNA_T - Complex)\cdot(RNA_{Anti\_T} - Complex)}{K_m} \quad \text{(S17)}$$

With the solution of:

$$Complex = \frac{(mRNA_T + RNA_{Anti\_T} + K_m) - \sqrt{(mRNA_T + RNA_{Anti\_T} + K_m)^2 - 4\cdot mRNA_T \cdot RNA_{Anti\_T}}}{2} \quad \text{(S18)}$$

The expression level of the output Z is proportional to the free mRNA:

$$Z = \alpha_Z \cdot \tau_Z \cdot (mRNA_T - Complex) + Z_{min} \quad \text{(S19)}$$

α_Z is the translation rate and τ_Z is the protein half-life. Z_{min} is a minimum protein level, analogous to Z_{min} in Eq. S14. The concentrations of $mRNA_T$ and RNA_{Anti_T} are

$$mRNA_T = M_f \frac{\left(\frac{X}{K_{df}}\right)^{n_f} + \beta_f}{1 + \left(\frac{X}{K_{df}}\right)^{n_f}} \quad \text{(S20.1)}$$

$$RNA_{Anti\_T} = M_r \frac{\left(\frac{Y}{K_{dr}}\right)^{n_r} + \beta_r}{1 + \left(\frac{Y}{K_{dr}}\right)^{n_r}} \quad \text{(S20.2)}$$

Where M_f and M_r are the maximum mRNA and RNA_{Anti} levels and are produced by the forward and reverse promoters, respectively. Simulation results of the antisense transcription model based on Eqs. S18 to S20.2 are shown in Fig. S9. Interestingly, the results show that when K_m is slightly equal to M_r, the antisense transcription model behaves as a pure subtraction with respect to *Y* level (Fig. S9A has a similar behavior to Fig. S2A). In this case, the FCA based on mRNA level is constant without dependency on *Y* levels (Fig. S10A), and FCA based on protein levels decreases when *Y* is increased (Fig. S10B, Z_{min}>0). In case that the affinity of the binding reaction between mRNA and antisense RNA is strong, i.e., K_m is small, increasing the Y level can improve the FCA (Figs. S9B, S9C, S10A, 10B). This interesting dependency on K_m can be explained by Eq. S17, when mRNA- RNA_{Anti} complex level is small:

$$Complex \ll mRNA_T + RNA_{Anti\_T} + K_m$$

$$\Rightarrow \quad Complex = mRNA_T \cdot \frac{RNA_{Anti\_T}}{K_m + RNA_{Anti\_T} + mRNA_T} \quad \text{(S21)}$$

Case 1: $K_m \gg mRNA_T + RNA_{Anti\_T}$, when K_m is larger than M_f *and* M_r: We can assume that the concentration of the complex is smaller than the concentrations of $mRNA_T$ and RNA_{Anti_T} and therefore, the complex concentration can be written as:

$$Complex = \frac{mRNA_T \cdot RNA_{Anti\_T}}{K_m} \quad \text{(S22.1)}$$

$$mRNA = \frac{mRNA_T}{K_m} \cdot (K_m - RNA_{Anti\_T}) \quad \text{(S22.2)}$$

$$mRNA = \frac{M_f}{K_m} \cdot \frac{\left(\frac{X}{K_{df}}\right)^{n_f} + \beta_f}{1 + \left(\frac{X}{K_{df}}\right)^{n_f}} \cdot \left(K_m - M_r \cdot \frac{\left(\frac{Y}{K_{dr}}\right)^{n_r} + \beta_r}{1 + \left(\frac{Y}{K_{dr}}\right)^{n_r}} \right) \quad \text{(S22.3)}$$

$$mRNA = \frac{M_f}{K_m} \cdot \frac{\left(\frac{X}{K_{df}}\right)^{n_f} + \beta_f}{1 + \left(\frac{X}{K_{df}}\right)^{n_f}} \cdot \frac{1}{1 + \left(\frac{Y}{K_{dr}}\right)^{n_r}} \left(K_m - M_r \cdot \beta_r + (K_m - M_r) \cdot \left(\frac{Y}{K_{dr}}\right)^{n_r} \right) \quad \text{(S22.4)}$$

We are interested in the case when $K_m \approx M_r$: Increasing the *Y* level causes to decrease the mRNA level only, without shifting the threshold (As shown in Eq. S22.4). Thus, in such conditions, *Y* acts as a pure subtraction.

Case 2: $K_m + RNA_{Anti\_T} \gg mRNA_T$

For the case that $M_r >> K_m$, and M_f is equal to K_m, the Eq. S22.1 is not valid and instead, we developed a new equation based on Eq. S21.:

$$\Rightarrow \quad Complex = mRNA_T \cdot \frac{RNA_{Anti\_T}}{K_m + RNA_{Anti\_T}} \quad \text{(S23.1)}$$

$$\Rightarrow \quad mRNA = mRNA_T - mRNA_T \cdot \frac{RNA_{Anti\_T}}{K_m + RNA_{Anti\_T}}$$

$$\Rightarrow \quad mRNA = mRNA_T \cdot \frac{K_m}{K_m + RNA_{Anti\_T}} \quad \text{(S23.2)}$$

$$\Rightarrow \quad mRNA = M_f \cdot \frac{\left(\frac{X}{K_{df}}\right)^{n_f} + \beta_f}{1 + \left(\frac{X}{K_{df}}\right)^{n_f}} \cdot \frac{1}{1 + \frac{M_r}{K_m} \cdot \frac{\left(\frac{Y}{K_{dr}}\right)^{n_r} + \beta_r}{1 + \left(\frac{Y}{K_{dr}}\right)^{n_r}}} \qquad \text{(S23.3)}$$

Here (Eq. S23.3), we can see that when $M_r/K_m>>1$: Increasing the Y level causes to decrease in the mRNA level, without shifting the threshold. Thus, in such conditions Y acts as a pure subtraction.

<u>Case 3:</u> $K_m < mRNA_T + RNA_{Anti\_T}$

In this case, K_m is small but is not negligible compared to the mRNA levels. In this case, we directly used Eq. S21, and substituted it in Eq. S16.1

$$\Rightarrow \quad mRNA = mRNA_T \cdot \frac{K_m + mRNA_T}{K_m + RNA_{Anti\_T} + mRNA_T} \qquad \text{(S24.1)}$$

$$\Rightarrow \quad mRNA = mRNA_T \cdot \frac{1 + \frac{mRNA_T}{K_m}}{1 + \frac{RNA_{Anti\_T}}{K_m} + \frac{mRNA_T}{K_m}} \qquad \text{(S24.2)}$$

$$\Rightarrow \quad mRNA = M_f \cdot \frac{\left(\frac{X}{K_{df}}\right)^{n_f} + \beta_f}{1 + \left(\frac{X}{K_{df}}\right)^{n_f}} \cdot \frac{1 + \frac{M_f}{K_m} \cdot \frac{\left(\frac{X}{K_{df}}\right)^{n_f} + \beta_f}{1 + \left(\frac{X}{K_{df}}\right)^{n_f}}}{1 + \frac{M_r}{K_m} \cdot \frac{\left(\frac{Y}{K_{dr}}\right)^{n_r} + \beta_r}{1 + \left(\frac{Y}{K_{dr}}\right)^{n_r}} + \frac{M_f}{K_m} \cdot \frac{\left(\frac{X}{K_{df}}\right)^{n_f} + \beta_f}{1 + \left(\frac{X}{K_{df}}\right)^{n_f}}} \qquad \text{(S24.3)}$$

For $M_f/K_m>>1$, and $M_r/K_m>>1$:

$$mRNA = M_f \cdot \frac{\left(\frac{X}{K_{df}}\right)^{n_f} + \beta_f}{1 + \left(\frac{X}{K_{df}}\right)^{n_f}} \cdot \frac{\frac{\left(\frac{X}{K_{df}}\right)^{n_f} + \beta_f}{1 + \left(\frac{X}{K_{df}}\right)^{n_f}}}{\frac{M_r}{M_f} \cdot \frac{\left(\frac{Y}{K_{dr}}\right)^{n_r} + \beta_r}{1 + \left(\frac{Y}{K_{dr}}\right)^{n_r}} + \frac{\left(\frac{X}{K_{df}}\right)^{n_f} + \beta_f}{1 + \left(\frac{X}{K_{df}}\right)^{n_f}}} \qquad \text{(S25)}$$

According to Eq. S25, Y level only shifts the threshold and decreases the basal level, and thus, the FCA level increases when X increases.

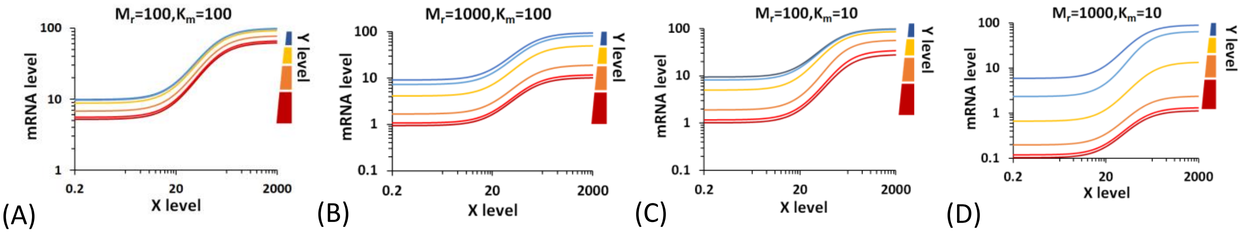

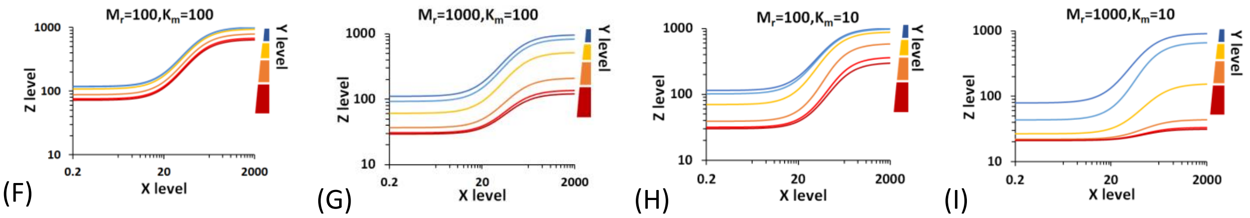

Fig. S9. Simulation results of antisense transcription model (Eqs. S18 to S20.2) as function of X levels (activity of forward promoter) and Y levels (activity of reverse promoter). Simulation parameters: n_f=1.5, n_r=1.5, K_{df}=100, K_{dr}=100, M_f=100, β_f=0.1, β_r=0.01, Z_{max}=1000, Zmin=20, $\alpha_Z \times \tau_Z$=10.

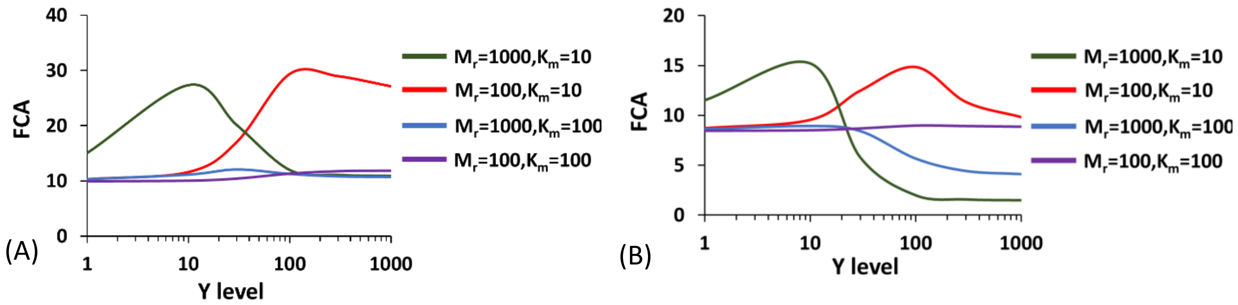

Fig. S10. Fold change activation (FCA) of the antisense transcription model as a function of the activity of reverse promoter (Y level), based on results Fig. S9, for (A) mRNA level, and (B) protein levels.

2.2. Models for ICF design: We developed two bio-models for the ICF design: (1) Molecular ICF using three nodes, which is based on biochemical reactions only, (2) Genetic ICF using four nodes, which is based on gene regulatory elements.

2.2.1. Molecular ICF three-nodes model: The next step was to develop a biomolecular model of the ICF circuit (Fig. S11) and test if this model is consistent with the models of the open loop circuits developed earlier in this work. The molecular ICF circuit comprise two branches (Fig. S11A): (1) the input molecule *X* activates the output molecule *Z*, and (2) the input molecule *X* inhibits the activity of molecule *Y*, which in turn inhibits the activity of *Z*. While both branches positively regulate the output *Z*, the first branch is a direct regulation of *Z*, and the second branch is an indirect regulation through cascading two inhibitors. The ICF circuit is also known as coherent feedforward type 4 (Mangan & Alon, 2003). The output signal of ICF is given by:

$$Z = Z_{max} \cdot \frac{\left(\frac{X}{K_{df}}\right)^{n_f} + \beta_f}{1+\left(\frac{X}{K_{df}}\right)^{n_f}} \cdot \frac{1}{1+\left(\frac{Y}{K_{dr}}\right)^{n_r}} \tag{S26.1}$$

$$Y = Y_{max} \cdot \frac{1}{1+\left(\frac{X}{K_{df}}\right)^{n_f}} \tag{S26.2}$$

Here the Y_{max} is the maximum molecular level achieved in the regulation of molecule *Y* by *X*, K_{dr} is the dissociation constant of binding *Y* and *Z*. The ratio Y_{max}/K_{dr} is the regulation strength of the second branch on the output *Z*. For simplicity, we assume that $n_r=1$, and by substituting the Eq. S26.2 into Eq. S26.1, we obtain:

$$Z = Z_{max} \cdot \frac{\left(\frac{X}{K_{df}}\right)^{n_f} + \beta_f}{1 + \left(\frac{X}{K_{df}}\right)^{n_f}} \cdot \frac{1 + \left(\frac{X}{K_{df}}\right)^{n_f}}{1 + \left(\frac{X}{K_{df}}\right)^{n_f} + \frac{Y_{max}}{K_{dr}}} + Z_{min} \qquad \text{(S27.1)}$$

$$Z = Z_{max} \cdot \frac{\left(\frac{X}{K_{df}}\right)^{n_f} + \beta_f}{1 + \left(\frac{X}{K_{df}}\right)^{n_f} + \frac{Y_{max}}{K_{dr}}} + Z_{min} \qquad \text{(S27.2)}$$

$$\Rightarrow \; FCA = \frac{Z|_{X \gg K_{df}}}{Z|_{X=0}} = \frac{1}{\beta_f} \cdot \left(1 + \frac{Y_{max}}{K_{dr}}\right) \qquad \text{(S27.3)}$$

The FCA given in Eq. S27.3 was calculated for $Z_{min}=0$, and it shows that increasing the Y_{max}/K_{dr} leads to increase in the FCA level. Eq. S27.1 is equivalent to Eq. S25 and Eq. S27.2 is equivalent to Eq. S14 when $\rho_1=1$ and $\rho_2=0$, which means that our models of antisense transcription and transcriptional interference under specific conditions has similar equations as ICF circuit. The simulation results of molecular ICF circuit (Eq. S27) are shown in Fig. S11B for $Z_{min}=0$ and Fig. S11C for $Z_{min}\neq 0$. Both indicate that FCA can be improved by increasing Y_{max}/K_{dr} level (Fig. S11D). We also presented the sensitivity in Fig. S11E for $Z_{min}=0$ and Fig. S11F for $Z_{min}\neq 0$ in order to evaluate the MDL. As we expected, improving the FCA by increasing the Y_{max}/K_{dr} level resulted in an increased MDL (Fig. S11G).

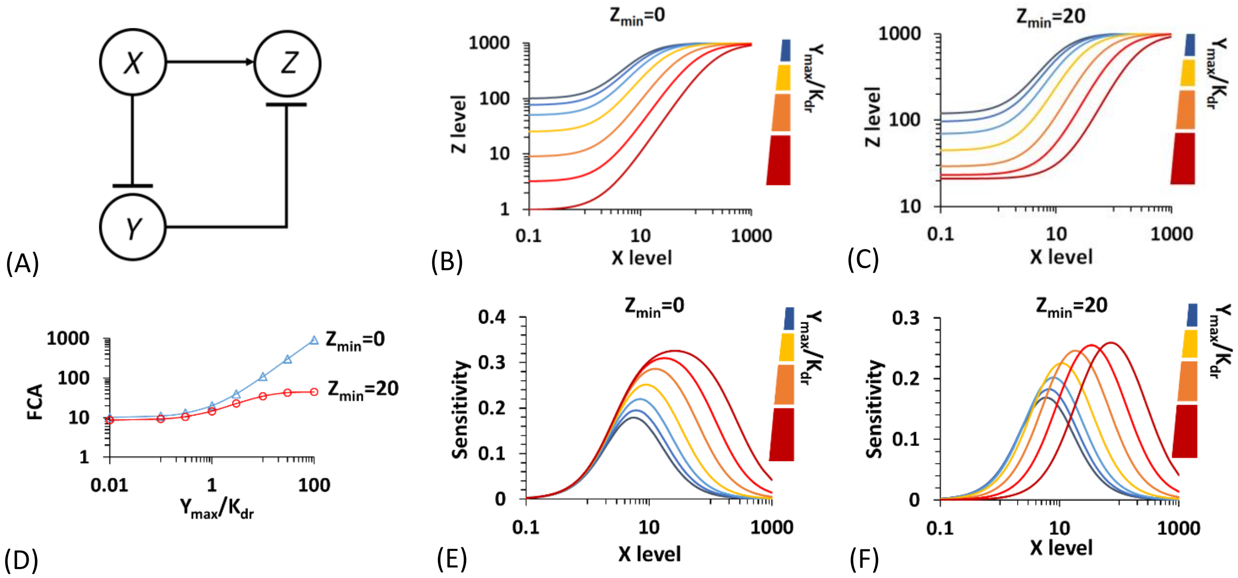

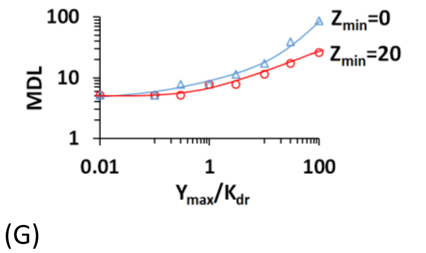

Fig. S11. (A) Schematic diagram of molecular indirect coherent feedforward (ICF), (B) and (C) Simulation results of molecular ICF circuit (Eq. S27) as a function of X levels and inhibition strength from the second branch (Y_{max}/K_{dr}) for $Z_{min}=0$ and $Z_{min}=20$, respectively. (D) Fold change activation (FCA) as a function of inhibition strength from the second branch (Y_{max}/K_{dr}). (E)-(F) Sensitivity results based on the simulation results for the ICF circuit, with $Z_{min}=0$ and $Z_{min}=20$, respectively. (G) Minimum detection level versus Y_{max}/K_{dr} level. Simulation parameters: $n_f=1.5$, $K_{df}=100$, $\beta_f=0.1$, $Z_{max}=1000$.

2.2.2. Genetic ICF four-nodes model: The implementation of ICF in living cells using gene regulation requires another node, because transcription factors often can either repress or activate promoters (some synthetic parts can have dual operations, however the efficiency of these parts is low). Therefore, a node *R* is added to receive the positive regulation from *X* that directly represses *Y* (Fig. S12A). Fig. S12B shows the implementation of ICF using gene regulation based on antisense transcription model, where the output *Z* is given by:

$$Z = \alpha_Z \cdot \tau_Z \cdot (mRNA_T - Complex) + Z_{min} \quad \text{(S19)}$$

$$Complex = \frac{(mRNA_T + mRNA_{Anti\_T} + K_m) - \sqrt{(mRNA_T + mRNA_{Anti\_T} + K_m)^2 - 4 \cdot mRNA_T \cdot mRNA_{Anti\_T}}}{2} \quad \text{(S18)}$$

$$mRNA_T = M_f \frac{\left(\frac{X}{K_{df}}\right)^{n_f} + \beta_f}{1 + \left(\frac{X}{K_{df}}\right)^{n_f}} \quad \text{(S20.1)}$$

$$mRNA_{Anti\_T} = M_r \frac{\left(\frac{Y}{K_{dr}}\right)^{n_r} + \beta_r}{1 + \left(\frac{Y}{K_{dr}}\right)^{n_r}} \quad \text{(S20.2)}$$

$$R = R_{max} \frac{\left(\frac{X}{K_{df}}\right)^{n_f} + \beta_f}{1 + \left(\frac{X}{K_{df}}\right)^{n_f}} \quad \text{(S28)}$$

$$Y = Y_{max} \frac{1}{1 + \left(\frac{R}{K_R}\right)^{n_R}} \quad \text{(S29)}$$

As we showed earlier, the binding affinity (K_m) between mRNA and antisense RNA can determine the functionality of antisense transcription genetic unit (Eq. S25, Fig. S9 and Fig. S10). For a strong binding affinity, the reverse promoter shifts the threshold and increases the FCA, similar to a comparator. For a weak binding affinity, the reverse promoter acts as subtraction, without increasing the FCA. Therefore, the implementation of ICF design using antisense transcription genetic unit can yield different FCA levels, based on the K_m value. In the circuit design, the forward promoter regulates the output signal and the transcription factor *R* (Fig. S12B). The repressor *R* regulates the activity of *Y*, which activates the antisense transcription unit from the reverse promoter P_{Rev}. The simulation results of ICF circuit using antisense transcription with a strong binding affinity (K_m is small), acting as a comparator, are shown in Fig. S12C for $Z_{min}=0$ and in Fig. S12D for $Z_{min}\neq 0$. With a weak binding affinity (K_m is large), the ICF circuit acts as a subtractor. The results are shown in Fig. S12E for $Z_{min}=0$ and in Fig. S12F for $Z_{min}\neq 0$. We also evaluated the FCA of this system, and in both cases for weak and strong binding affinities an improvement of the FCA was observed for a specific range K_R (Fig. S12G and H), where the K_R is the dissociation constant between repressor *R* and promoter P_R. Fig. S12I shows the simulation results for various molecules in the circuit as a function of X levels.

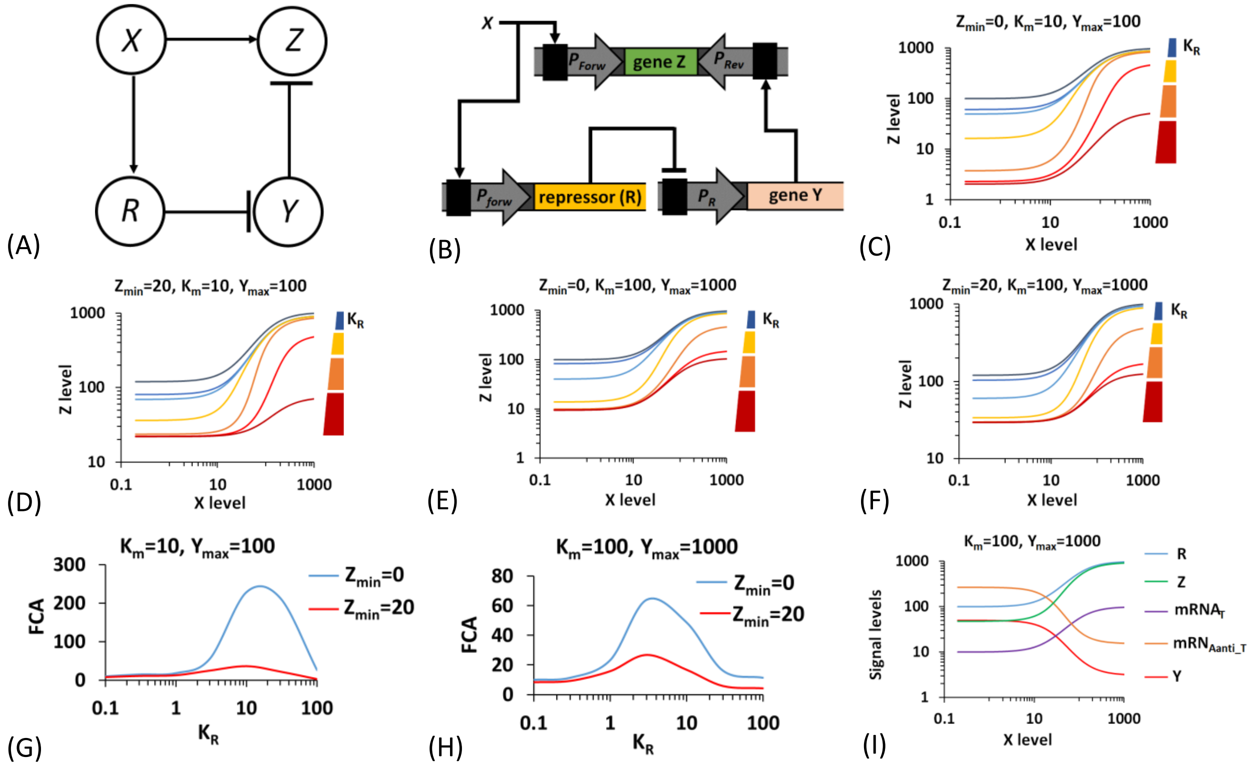

Fig. S12. (A) Schematic diagram of indirect coherent feedforward (ICF) in living cells using four nodes regulated by the input X. (B) The implementation of ICF design according to the diagram shown in A, based on antisense transcription. (C) and (D) Simulation results of output *Z* levels in ICF circuit (Eqs. S28) as a function of *X* levels. The circuit acts as a comparator. The dissociation constant between repressor *R* and promoter P_R (K_R) is varied across levels with $Z_{min}=0$ and $Z_{min}=20$, respectively. Simulation parameters are $n_f=1.5$, $n_r=1.5$, $K_{df}=100$, $K_{dr}=100$, $M_f=100$, $M_r=1000$, $\beta_f=0.1$, $\beta_r=0.01$, $Z_{max}=1000$, $Z_{min}=20$, $\alpha_Z \times \tau_Z=10$, $n_R=1.5$, $Y_{max}=100$, $K_m=10$. (E) and (F) Simulation results of output *Z* levels in ICF circuit (Eqs. S28) as a function of *X* levels. The circuit acts as a subtractor. The dissociation constant between repressor *R* and promoter P_R (K_R) is varied across levels with $Z_{min}=0$ and $Z_{min}=20$, respectively. Simulation parameters are $n_f=1.5$, $n_r=1.5$, $K_{df}=100$, $K_{dr}=100$, $M_f=100$, $M_r=1000$, $\beta_f=0.1$, $\beta_r=0.01$, $Z_{max}=1000$, $Z_{min}=20$, $\alpha_Z \times \tau_Z=10$, $n_R=1.5$, $Y_{max}=1000$, $K_m=100$. (G) FCA as a function of K_R, extracted from data that is shown in Fig. S12C and D. (H) FCA as a function of K_R, extracted from the data that is shown m Fig. S12E and F. (I) An example for all molecule levels in the ICF circuit based on antisense transcription.

Fig. S13A shows the implementation of ICF using gene regulation based on transcriptional interference model according to schematic diagram shown in Fig. S12A.The output Z is given by:

$$Z = Z_{max} \cdot \frac{\left(\frac{X}{K_{df}}\right)^{n_f} + \beta}{1 + \left(\frac{X}{K_{df}}\right)^{n_f} + \rho_1 \cdot \left(\frac{Y}{K_{dr}}\right)^{n_r} + \beta} \cdot \left(\frac{1}{1 + \rho_2 \cdot \left(\frac{Y}{K_{dr}}\right)^{n_r}}\right) + Z_{min} \qquad \text{(S14)}$$

$$R = R_{max} \frac{\left(\frac{X}{K_{df}}\right)^{n_f} + \beta_f}{1 + \left(\frac{X}{K_{df}}\right)^{n_f}} \qquad \text{(S28)}$$

$$Y = Y_{max} \frac{1}{1 + \left(\frac{R}{K_R}\right)^{n_R}} \qquad \text{(S29)}$$

As we showed that values of ρ_1 *and* ρ_2 parameters in Eq. S29.1 can determine the functionality of the genetic unit, either shifting the threshold acting as a comparator or repressing the expression level acting as a subtractor. The implementation of an ICF design using transcriptional interference genetic unit is shown in Fig. S13A. The forward promoter regulates the output signal as well as the transcription factor *R*, which represses gene Y. The Y protein activates the reverse promoter of the transcriptional interference unit. Here we only examine the transcriptional interference unit as a subtractor. The simulation results of ICF design using a transcriptional interference model with $\rho_1=0$, $\rho_2=1$, acting as a subtractor, are shown in Fig. S13B for $Z_{min}=0$ and in Fig. S13C for $Z_{min}\neq 0$. The estimated FCA level of this system shows an improvement when Y_{max} level increases (Fig. S13E). By comparing the FCA of antisense transcription (Fig. S12H) and transcriptional interference models (Fig. S13D), we can find two major differences:

(1) For $Z_{min}=0$, while the FCA level of antisense transcription has an optimum, the FCA level of transcriptional interference monotonically increases with respect to increasing strength activity of the reverse promoter.
(2) For $Z_{min}\neq 0$ and for the same reverse promoter activity, the FCA level of antisense transcription is higher than the FCA level of transcriptional interference.

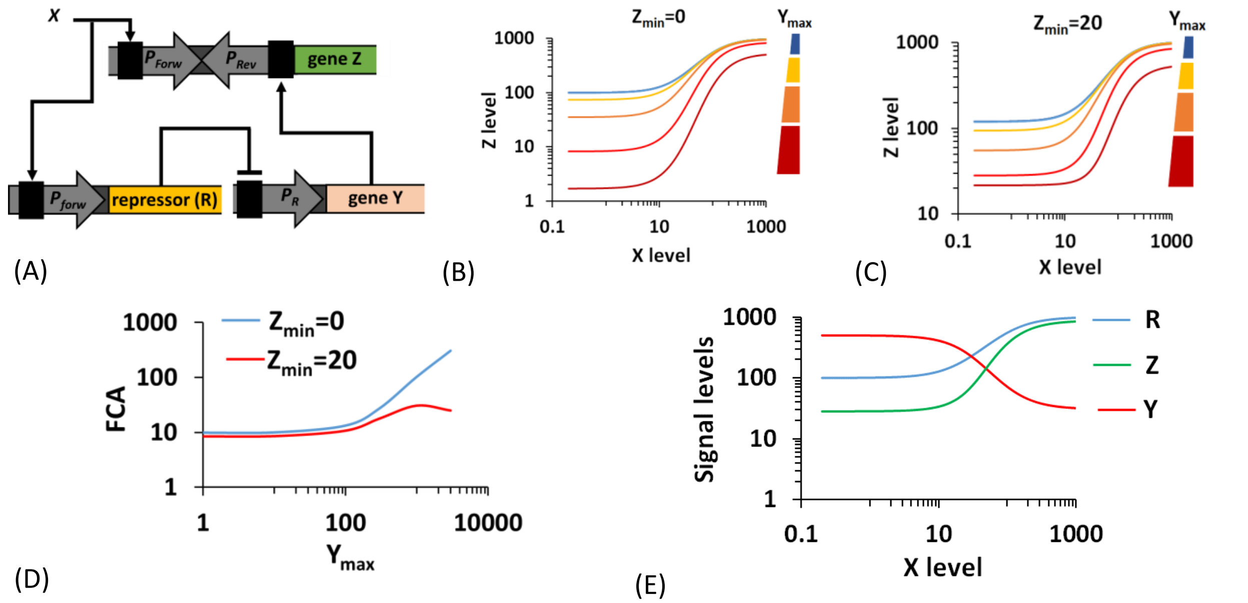

Fig. S13. (A) Implementation of ICF design according to the schematic diagram shown in Fig. S12A, using transcriptional interference model in living cells. (B) and (C) Simulation results of ICF circuit (Eqs. S29) acting as a subractor, as a function of *X* levels. The maximum protein level (Y_{max}) is varied by adjusting the repressor *R* for $Z_{min}=0$, *and* $Z_{min}=20$, respectively. Simulation parameters: $n_f=1.5$, $n_r=1.5$, $K_{df}=100$, $K_{dr}=100$, $\beta=0.1$, $Z_{max}=1000$, $n_R=1.5$, $K_R=100$, $\rho_1=0$, *and* $\rho_2=1$. (D) FCA level versus $\mathrm{Y_{max}}$, extracted from the data that is shown in Fig. S13B and C. (E) An example for all molecular signals in the ICF circuit based on transcriptional interference.

2.3. Models for DNF design: We developed two bio-models for the DNF design: (1) Molecular DNF using three nodes, which is based on biochemical reactions only, (2) Genetic DNF using four nodes, which is based on gene regulatory elements.

2.3.1. Molecular DNF three-nodes model: The molecular DNF circuit is shown in Fig. S14A. In the circuit, an input molecule *X* activates the output molecule *Z*. The output *Z* inhibits the activity of the

molecule *Y*, which in turn inhibits the activity of *Z*, leading to a mutual negative feedback between *Y* and *Z* molecules. The output signal of DNF is given by:

$$Z = Z_{max} \cdot \frac{\left(\frac{X}{K_{df}}\right)^{n_f} + \beta_f}{1+\left(\frac{X}{K_{df}}\right)^{n_f}} \cdot \frac{1}{1+\left(\frac{Y}{K_{dr}}\right)^{n_r}} + Z_{min} \tag{S30.1}$$

$$Y = Y_{max} \cdot \frac{1}{1+\left(\frac{Z}{K_{dz}}\right)^{n_z}} \tag{S30.2}$$

For simplicity we assume that $n_r=1$, and $n_z=1$, $Z_{min}=0$:

$$Z = Z_{max} \cdot f(x) \cdot \frac{1}{1+\frac{Y}{K_{dr}}}$$

$$\Rightarrow \quad Z + Z \cdot \frac{Y}{K_{dr}} = Z_{max} \cdot f(x) \tag{S31.1}$$

$$Y = Y_{max} \cdot \frac{1}{1+\frac{Z}{K_{dz}}} \tag{S31.2}$$

Substituting Eq. S31.2 into Eq. S31.1:

$$Z + Z \cdot \frac{Y_{max}}{K_{dr}} \cdot \frac{1}{1+\frac{Z}{K_{dz}}} = Z_{max} \cdot f(x) \tag{S32.1}$$

$$Z \cdot \left(1 + \frac{Z}{K_{dz}}\right) + Z \cdot \frac{Y_{max}}{K_{dr}} = Z_{max} \cdot f(x) \cdot \left(1 + \frac{Z}{K_{dz}}\right) \tag{S32.2}$$

$$Z^2 + Z \cdot K_{dz} \cdot \left(1 + \frac{Y_{max}}{K_{dr}} - \frac{Z_{max} \cdot f(x)}{K_{dz}}\right) = K_{dz} \cdot Z_{max} \cdot f(x) \tag{S32.3}$$

$$Z = K_{dz} \cdot \frac{-\left(1+\frac{Y_{max}}{K_{dr}}-\frac{Z_{max}\cdot f(x)}{K_{dz}}\right)+\sqrt{\left(1+\frac{Y_{max}}{K_{dr}}-\frac{Z_{max}\cdot f(x)}{K_{dz}}\right)^2+4\cdot\frac{Z_{max}}{K_{dz}}\cdot f(x)}}{2} \tag{S32.4}$$

We then calculated the lowest and highest levels for Z

$$Z_H|_{f(x \gg K_{df})=1} = K_{dz} \cdot \frac{-\left(1+\frac{Y_{max}}{K_{dr}}-\frac{Z_{max}}{K_{dz}}\right)+\sqrt{\left(1+\frac{Y_{max}}{K_{dr}}-\frac{Z_{max}}{K_{dz}}\right)^2+4\cdot\frac{Z_{max}}{K_{dz}}}}{2} \tag{S33.1}$$

$$Z_L|_{f(x \ll K_{df})=\beta} = K_{dz} \cdot \frac{-\left(1+\frac{Y_{max}}{K_{dr}}-\frac{Z_{max}\cdot\beta}{K_{dz}}\right)+\sqrt{\left(1+\frac{Y_{max}}{K_{dr}}-\frac{Z_{max}\cdot\beta}{K_{dz}}\right)^2+4\cdot\frac{Z_{max}}{K_{dz}}\cdot\beta}}{2} \tag{S33.2}$$

The FCA is defined as: $FCD = \frac{Z_H}{Z_L}$, and then, we obtain:

$$FCA\text{ד} = \frac{-\left(1+\frac{Y_{max}}{K_{dr}}-\frac{Z_{max}}{K_{dz}}\right)+\sqrt{\left(1+\frac{Y_{max}}{K_{dr}}-\frac{Z_{max}}{K_{dz}}\right)^2+4\cdot\frac{Z_{max}}{K_{dz}}}}{-\left(1+\frac{Y_{max}}{K_{dr}}-\frac{Z_{max}}{K_{dz}}\cdot\beta\right)+\sqrt{\left(1+\frac{Y_{max}}{K_{dr}}-\frac{Z_{max}}{K_{dz}}\cdot\beta\right)^2+4\cdot\frac{Z_{max}}{K_{dz}}\cdot\beta}} \tag{S34}$$

The simulation results of molecular DNF are shown in Fig. S14B for $Z_{min}=0$. We also analyzed the sensitivity (Fig. S14C) and evaluated the MDL. As we expected, increasing the Y_{max}/K_{dr} level leads to improvement of the FCA level and worsening the MDL (Fig. S14D). Interestingly, when $Z_{min}=0$, the FCA of molecular DNF has an optimum over a range of Y_{max} level (Fig. S14E), in contrast to molecular ICF design (Fig. S11).

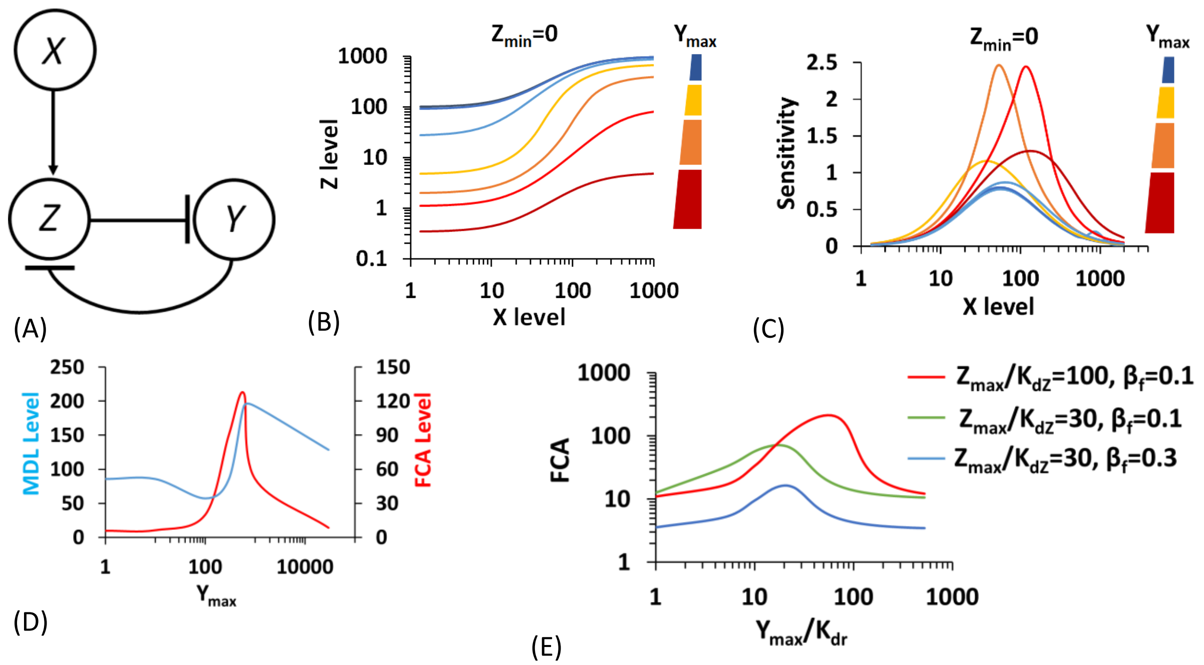

Fig. S14. (A) Schematic diagram of molecular double negative feedback (DNF) design, (B) Simulation results of molecular DNF circuit (Eq. S27) as a function of X levels for $Z_{min}=0$. The Y_{max}/K_{dr} represents the strength of negative feedback loop. (C) Sensitivity analysis based on the simulation results of molecular DNF circuit. (D) FCA and MDL levels as a function of feedback strength. (E) FCA levels for various circuit parameters. Simulation parameters are: $n_f=1.5$, $K_{df}=100$, $\beta_f=0.1$, $Z_{max}=1000$, $K_{dZ}=10$, $K_{dr}=10$.

2.3.2. Genetic DNF four-nodes model: Implementation of DNF in living cells using gene circuits requires another node as shown Fig. S15A. Here R and Z are both controlled by the same nodes (X, Y) and therefore have similar behavior. In our design, Z is used as a probe molecule (Z). Fig. S15B shows the implementation of DNF using gene regulation based on antisense transcription model, where the output Z is given by:

$$Z = \alpha_Z \cdot \tau_Z \cdot (mRNA_T - Complex) + Z_{min} \quad \text{(S19)}$$

$$R = R_{max} \cdot (mRNA_T - Complex) \quad \text{(S35)}$$

$$Complex = \frac{(mRNA_T + RNA_{Anti\_T} + K_m) - \sqrt{(mRNA_T + RNA_{Anti\_T} + K_m)^2 - 4 \cdot mRNA_T \cdot RNA_{Anti\_T}}}{2} \quad \text{(S18)}$$

$$mRNA_T = M_f \frac{\left(\frac{X}{K_{df}}\right)^{n_f} + \beta_f}{1 + \left(\frac{X}{K_{df}}\right)^{n_f}} \quad \text{(S20.1)}$$

$$RNA_{Anti\_T} = M_r \frac{\left(\frac{Y}{K_{dr}}\right)^{n_r} + \beta_r}{1 + \left(\frac{Y}{K_{dr}}\right)^{n_r}} \quad \text{(S20.2)}$$

$$Y = Y_{max}\frac{1}{1+\left(\frac{R}{K_R}\right)^{n_R}} \tag{S29}$$

The implementation of DNF design using antisense transcription genetic is shown in Fig. S15B. The forward promoter regulates the output signal as well as the repressor *R*, which represses the expression of gene *Y*. The protein Y activates the reverse promoter of the antisense transcription units, which are located downstream to gene *Z* and repressor *R*. Thus, *R* and *Y* comprise a double negative feedback. Here, we assumed that the binding affinity of mRNA- antisense mRNA is weak (K_m is large), and therefore, the antisense transcription unit acts as a subtractor. The simulation results of such systems are shown in Fig. S15C for $Z_{min}=0$, in Fig. S15D for $Z_{min}\neq 0$, and in Fig. S15E for *Y* signal. We also evaluated the FCA level of this system. In both cases for $Z_{min}=0$ and $Z_{min}\neq 0$, an improvement of the FCA can be achieved for a range of K_R (Fig. S15.F). The K_R is the dissociation constant between repressor *R* and promoter P_{forw_R}.

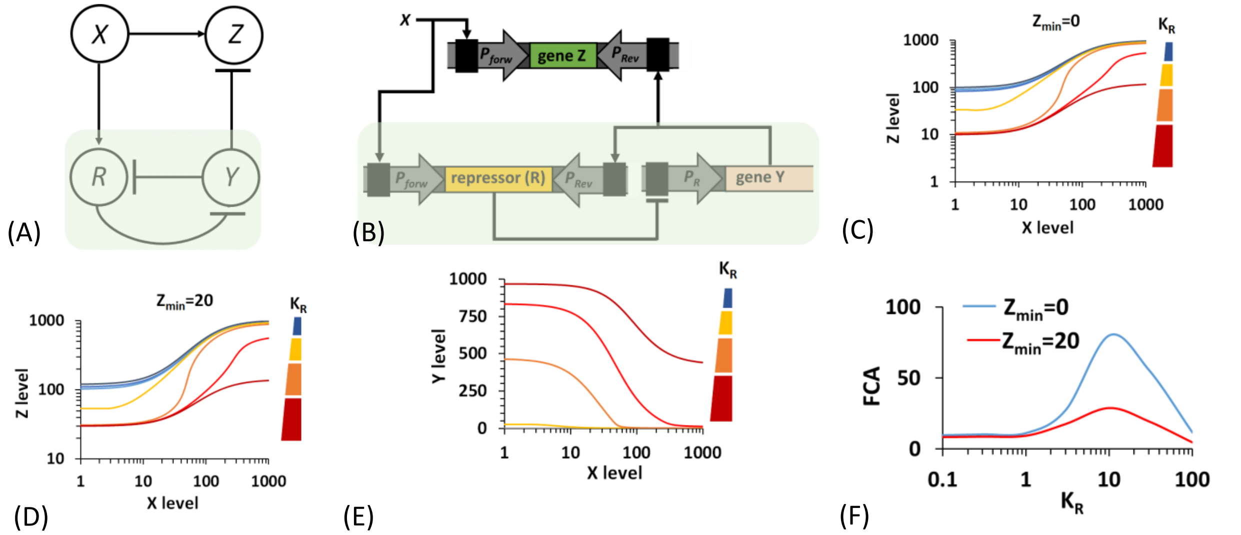

Fig. S15. (A) Schematic diagram of double negative feedback (DNF) design in living cells using four nodes. The area marked in green is the DNF loop. (B) The implementation of DNF design according to the diagram in A, using antisense transcription model in living cells. The area marked in green is the DNF loop. (C), (D) and (E) Simulation results of DNF circuit (Eqs. S35) as a function of X levels. The DNF circuit acts as a subtractor for various K_R values. K_R is the dissociation constant for the binding of repressor *R* to the promoter P_R.(C) *Z* level at $Z_{min}=0$. (D) *Z* level at $Z_{min}=20$. (E) Y level. Simulation parameters are: $n_f=1.5$, $n_r=1.5$, $K_{df}=100$, $K_{dr}=100$, $M_f=100$, $M_r=1000$, $\beta_f=0.1$, $\beta_r=0.01$, $Z_{max}=1000$, $Zmin=20$, $\alpha_Z\times\tau_Z=10$, $n_R=1$, $Y_{max}=1000$, $K_m=100$. (F) FCA as a function of K_R derived from the data from Fig. S15C and D.

Fig. S16A shows the implementation of DNF using gene regulation based on transcriptional interference model according to schematic diagram shown in Fig. S15A. The output Z is given by:

$$Z = Z_{max}\cdot\frac{\left(\frac{X}{K_{df}}\right)^{n_f}+\beta}{1+\left(\frac{X}{K_{df}}\right)^{n_f}+\rho_1\cdot\left(\frac{Y}{K_{dr}}\right)^{n_r}+\beta}\cdot\left(\frac{1}{1+\rho_2\cdot\left(\frac{Y}{K_{dr}}\right)^{n_r}}\right)+Z_{min} \tag{S14}$$

$$R = R_{max}\cdot\frac{\left(\frac{X}{K_{df}}\right)^{n_f}+\beta}{1+\left(\frac{X}{K_{df}}\right)^{n_f}+\rho_1\cdot\left(\frac{Y}{K_{dr}}\right)^{n_r}}\cdot\left(\frac{1}{1+\rho_2\cdot\left(\frac{Y}{K_{d}}\right)^{n_r}}\right) \tag{S36}$$

$$Y = Y_{max} \frac{1}{1+\left(\frac{R}{K_R}\right)^{n_R}} \quad \text{(S29)}$$

The forward promoter regulates the output signal and also the repressor R, which represses the expression of gene Y. The protein Y activates the reverse promoter of the transcriptional interference unit. The reverse promoter downregulates both the output signal (gene Z) and the repressor R. As a result, R and Y comprise a double negative feedback. DNF circuit that uses transcriptional interference model with $\rho_1=0$, $\rho_2=1$, acts as a comparator. The simulation results are shown in Fig. S16B for $Z_{min}=0$ and in Fig. S16C for $Z_{min}\neq 0$. We also evaluated the FCA level of this system (Fig. S16E), which can reach an optimal value at a specific Y_{max} level (Fig. S16F)

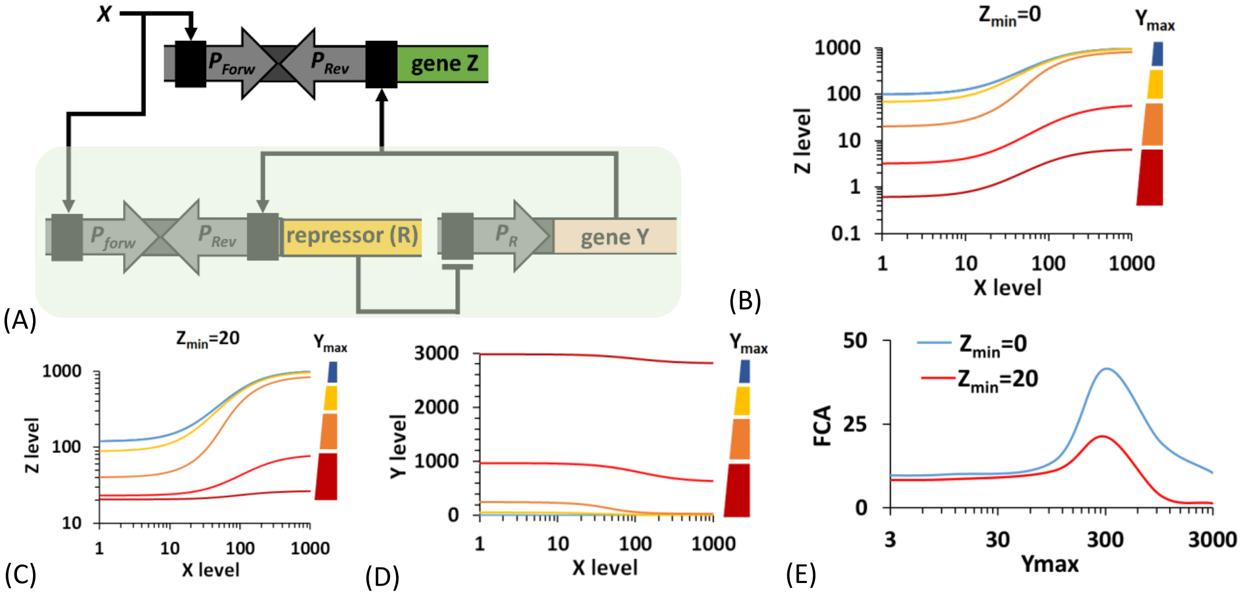

Fig. S16. (A) Implementation of DNF design according to the schematic diagram in Fig. S15A, using transcriptional interference model in living cells. The area marked in green is the DNF loop. (B), (C) and (D) Simulation results of DNF circuit (Eq. S36) as a function of X levels. The circuit acts as a subtractor and is regulated under various maximum levels of Y protein (Y_{max}). (B) Output Z level when $Z_{min}=0$. (C) Output Z level when $Z_{min}=20$. (D) Y signals. Simulation parameters are $n_f=1.5$, $n_r=1.5$, $K_{df}=100$, $K_{dr}=100$, $\beta=0.1$, $Z_{max}=1000$, $n_R=1.5$, $K_R=100$, $\rho_1=0$, and $\rho_2=1$. (E) FCA level as a function of Y_{max} derived from the data from Fig. S15B and C.

3. Experimental results:

3.1 The synthetic promoter P_{BADsyn}

The synthetic promoter P_{BADsyn} used in OL, ICF and DNF circuits contains only I_1 and I_2 binding sites, without O_2 DNA sites.2

Various synthetic circuits sensitive to arabinose were transformed into bacterial cells, where arabinose-inducible synthetic promoter P_{BADsyn} (w/o O_2 binding site for araC) was used as forward promoter and AHL-inducible promoter P_{lux} was used as a reverse promoter, located upstream to *gfp* gene. Arabinose and AHL

bind to their cognate transcriptional activators, araC and LuxR, generating complexes which bind to forward and reverse promoters, respectively. The transcriptional activator araC was constitutively expressed. LuxR was placed under the control of P_{tetO}, which functioned as a constitutive promoter when TetR wasn't included in the circuit .

Overnight grown bacterial cells containing and maintaining the circuit under test were diluted 50 times in the morning into fresh M9 minimal media and incubated for 2h before the induction with arabinose and additional inducers. Fluorescence was measured 3h after induction.

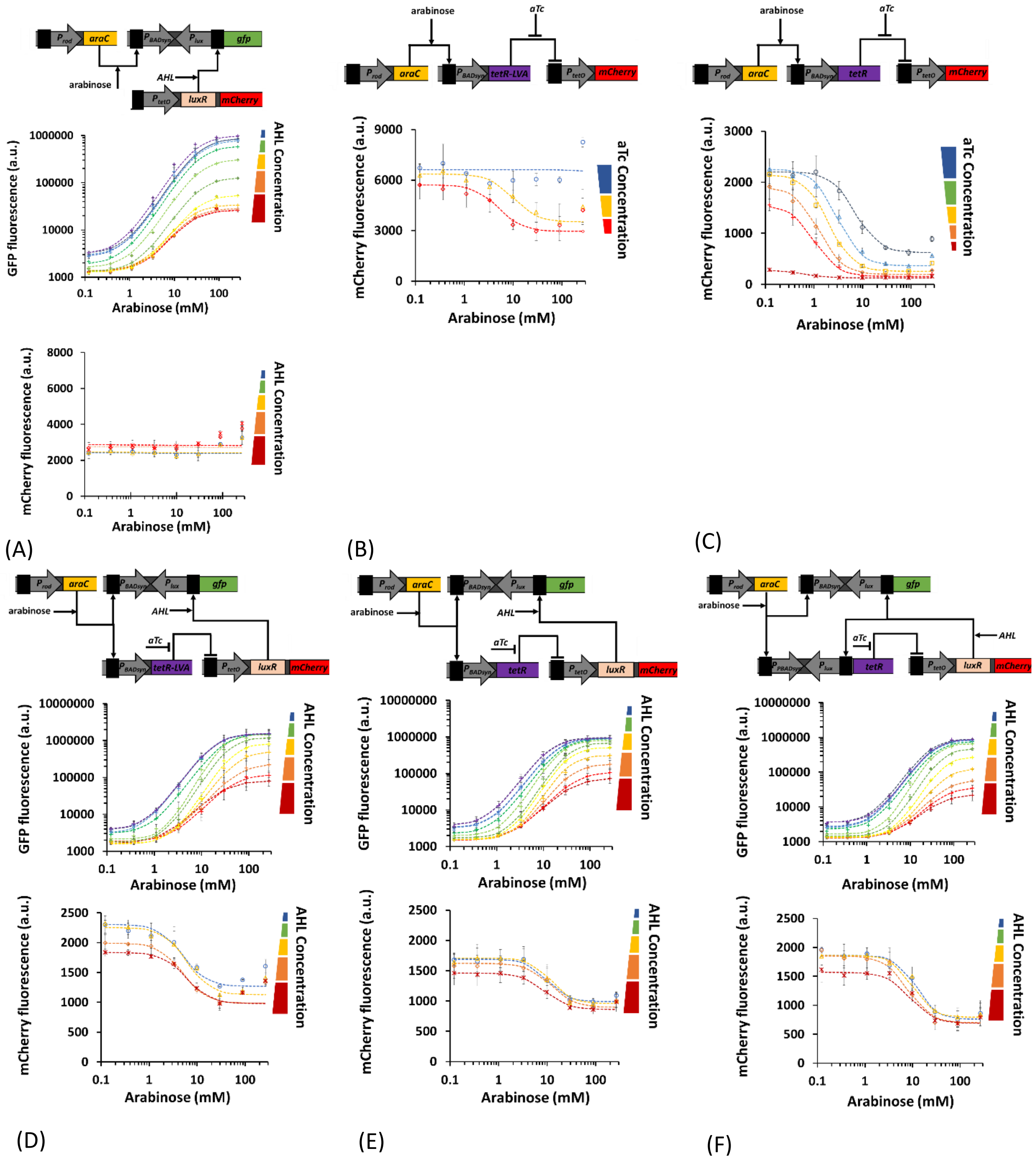

Fig. S17. P_{BADsyn} circuits in OL, ICF and DNF designs implemented using transcription interference. The upper panel contains the scheme for the synthetic circuit encompassing the promoter under test (P_{PUT}) and the lower panel shows the measured fluorescence for the tested circuit (Flow cytometry data for building this figure is described in Fig. S24-S33).

3.1.1 OL: At high AHL concentrations, the transcription of GFP by RNAPs from forward promoter is inhibited upon binding of AHL-LuxR complexes to the reverse promoter P_{lux}. With higher AHL concentrations, the transcriptional interference becomes stronger. The transcriptional interference originated from this RNAPs collision could be detected by the reduction of both minimum and maximum promoter activities of synthetic P_{BADsyn}. LuxR is constitutively expressed along entire range of arabinose concentrations and remains constant at various AHL concentrations, as can be seen by mCherry levels (Fig. S17A).

3.1.2 Arabinose-aTc inverting switch (TetR-LVA): The activity of P_{tetO} represented by mCherry levels in response to arabinose can be adjusted under various concentrations of aTc. At intermediate aTc concentrations, mCherry level is repressed as arabinose concentration increases from low to high. At high aTc concentrations, more aTc-TetR-LVA complexes are formed and are released from promoter $P_{tetO,}$ allowing transcription of mCherry. The LVA-degradation tag is responsible for reducing TetR (LVA) expression level and decreasing the ON/OFF ratio of P_{tetO} activity. An optimal concentration of anhydrotetracycline(aTc) was deduced to give the highest ON/OFF ratio to work with in the ICF and DNF circuits (explained below in 3.1.4, 3.1.5 and 3.1.6). If no significant differences in mCherry levels were observed between different concentrations of aTc then no aTc was added in the ICF and DNF circuits. In addition, the mCherry gene was later replaced by LuxR-mCherry operon to be integrated with the transcriptional interference in ICF and DNF circuits (Fig. S17B).

3.1.3 Arabinose-aTc inverting switch (TetR): The same as in 3.1.2 but with TetR instead of TetR-LVA (Fig. S17C).

3.1.4 ICF-TetR-LVA: The arabinose-inducible synthetic promoter P_{BADsyn} was regulated by adjusting between its minimum expression level (basal level) and its maximum expression level (the ON state). The basal level was controlled through the activity of P_{lux} induced by AHL-LuxR complex. Whereas the maximum expression level was controlled by TetR-LVA protein, which represses the expression of LuxR. To regain the maximum promoter activity level of synthetic P_{BADsyn} that was reduced in OL (see 3.1.1) we engineered transcriptional repressor TetR-LVA regulated by synthetic P_{BADsyn}. When arabinose concentration is high the TetR-LVA levels are also high, which in turn bind to P_{tetO} promoter and inhibit LuxR expression, thereby arresting the transcriptional interference formed by RNAPs transcribing from the reverse promoter P_{lux} and reducing the strength of feedforward loop. At low arabinose concentrations, TetR-LVA levels are not sufficient to bind P_{tetO} to repress LuxR expression, therefore -AHL- LuxR complexes bind to the reverse promoter $P_{lux,}$ inducing transcriptional interference and in turn increase the strength of the feedforward loop. This tuning is required to obtain low basal level at low arabinose concentration, and to reach maximum activity of promoter at high arabinose levels. The mCherry gene engineered downstream to LuxR under control of the same promoter P_{tetO}, acted as an indicator of P_{tetO} activity and LuxR levels. Thus, at low arabinose concentrations, LuxR and mCherry levels were high whereas at high arabinose concentrations, LuxR and mcherry levels were low. Overall, the combination of transcriptional interference with inverting switch improved the activity of P_{BADsyn} (Fig. S17D).

3.1.5 ICF-TetR: The same as in 3.1.4 but with TetR instead of TetR-LVA (Fig. S17E).

3.1.6 DNF-TetR: The same as in 3.1.4 but with TetR instead of TetR-LVA. AHL-inducible reverse promoter P_{lux} was also incorporated upstream to *tetR* gene. Thus, TetR levels were controlled not only by arabinose but also by AHL concentrations (Fig. S17F).

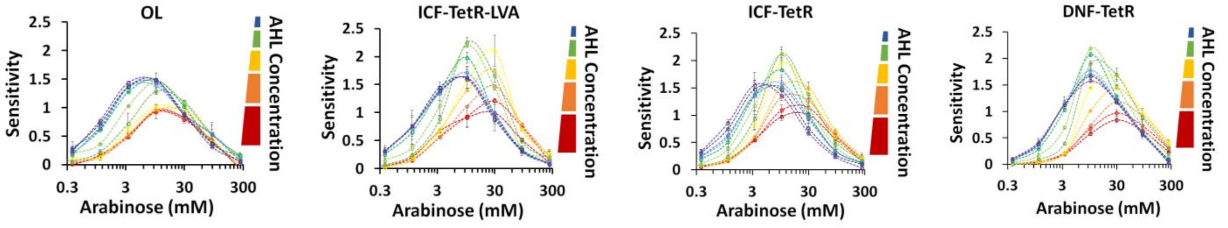

(A) (B) (C) (D)

Fig. S18. Sensitivity analysis for various P_{BADsyn} circuits. The sensitivity analysis of OL (A), ICF-TetR-LVA (B), ICF-TetR (C) and DNF-TetR (D) circuits with a diagrams described in Fig. S17A, Fig. S17D, S17E and S17F, respectively. The results were calculated based on equation S3.

3.2 The synthetic promoter P_{lacO}

IPTG is a commonly used inducer for P_{lacO} as a lactose analog. A synthetic circuit sensitive for IPTG was implemented based on transcriptional interference. P_{lacO} was used as forward promoter and AHL-inducible promoter P_{lux} was used as the reverse promoter. LacI was constitutively expressed. IPTG binds to transcriptional repressor LacI and releases the complex of lacI-IPTG from forward promoter P_{lacO}, allowing the expression of GFP. AHL binds to its transcriptional activators LuxR, creating AHL-LuxR complexes, which bind the reverse promoter $P_{lux,}$ thereby interfering with the transcription of P_{lacO} from the opposite direction. LuxR was placed under the control of P_{tetO}, which functioned as a constitutive promoter when TetR wasn't included in the circuit. An LVA-degradation tag was fused to LacI to increase LacI degradation.

Overnight grown bacterial cells containing and maintaining the circuit under test were diluted 100 times in the morning into fresh LB media and incubated for 0.5h before the induction with IPTG and additional inducers if required. Fluorescence was measured 4h after the induction.

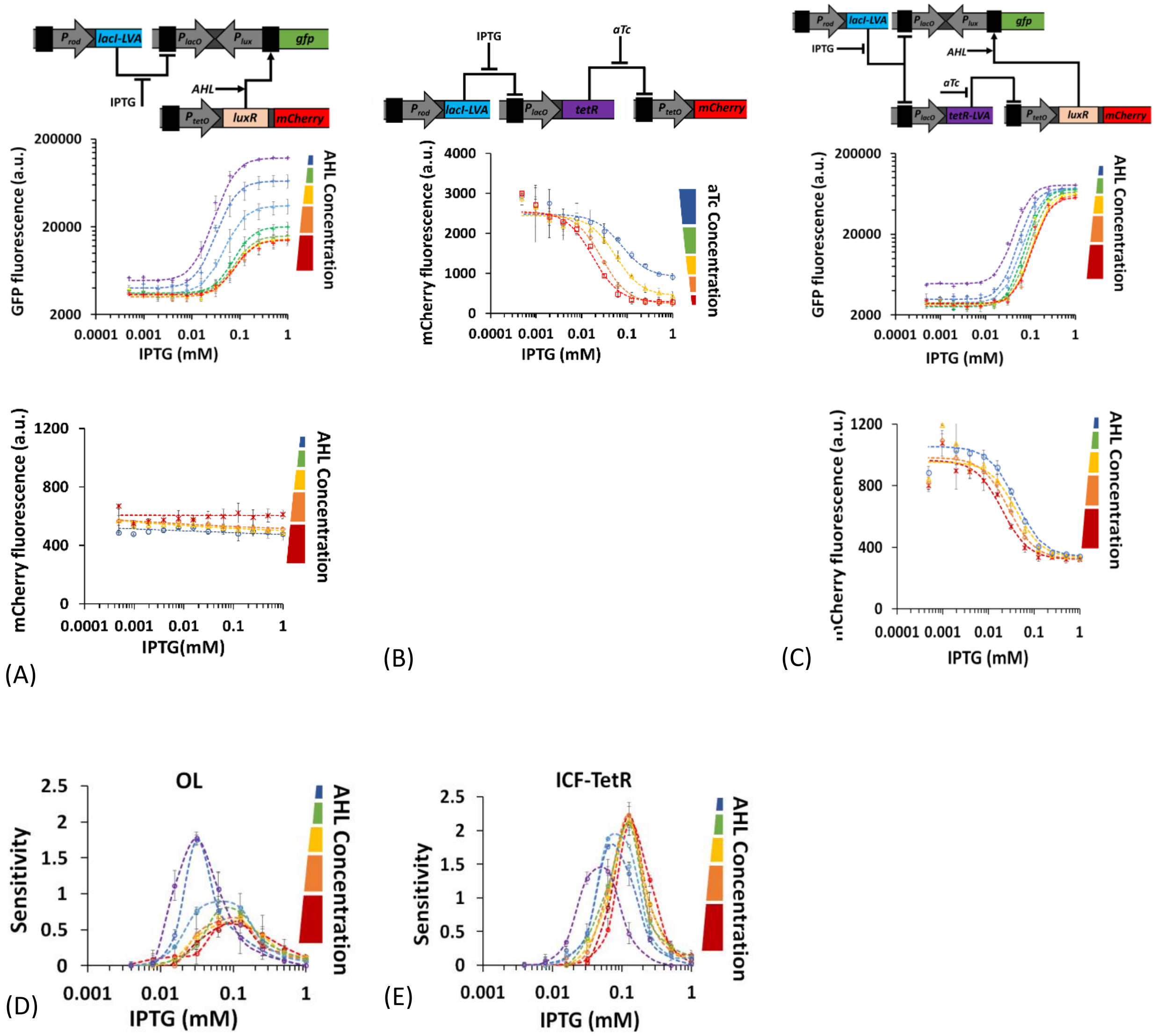

Fig. S19. Various P_{lacO} circuits implemented based on transcriptional interference. (A-C): The upper panel contains the scheme for the synthetic circuit encompassing the promoter under test P_{PUT} and the lower panel shows the measured fluorescence for the tested circuit. (D-E): The sensitivity analysis of OL and ICF-TetR-LVA circuits with schematic diagrams described in A and C, respectively. The results were calculated based on equation S3. (Flow cytometry data for building this figure is described in Fig. S34-S38).

3.2.1 OL: At high AHL concentrations, the transcription of GFP by RNAPs from forward promoter P_{lacO} is inhibited by RNAPs recruited by reverse promoter P_{lux} upon binding of AHL-LuxR complexes. With higher AHL concentrations, the transcriptional interference becomes stronger. The transcriptional interference could be detected by the reduction of both minimum and maximum promoter activity of P_{lacO}. LuxR is constitutively expressed through different concentrations of IPTG and can be seen by constant mCherry levels at different AHL concentrations (Fig. S19A).

3.2.2 IPTG-aTc inverting switch: The activity of P_{tetO} in response to IPTG can be adjusted using different concentrations of aTc, and is reported by mCherry level. At intermediate aTc concentrations, mCherry level is decreased as IPTG concentration increases from low to high. At high aTc concentrations, more aTc-TetR-LVA complexes are formed and are released from promoter $P_{lacO,}$

allowing transcription of mCherry. The LVA-degradation tag is responsible for reducing TetR-LVA expression. An optimal concentration of anhydrotetracycline(aTc) was deduced to give the highest ON/OFF ratio to work with in the ICF circuit (explained below in 3.2.3). If no significant differences in mCherry levels between the various concentrations of aTc were observed, then no aTc was added in the ICF circuit. The mCherry gene was later replaced by LuxR-mCherry operon to be integrated with the transcriptional interference in ICF circuit (Fig. S19B).

3.2.3 ICF-TetR-LVA: The IPTG-inducible promoter P_{lacO} was regulated by adjusting between its minimum expression level (basal level or the OFF state) and its maximum expression level (the ON state). The basal level was controlled through the activity of P_{lux} induced by AHL-LuxR complex. Whereas the maximum expression level was controlled by TetR-LVA protein, which represses the expression of LuxR. To regain the maximum promoter activity level of P_{lacO} that was reduced in OL (see 3.2.1) we engineered transcriptional repressor TetR-LVA regulated by P_{lacO}. When IPTG concentration is high, the level of TetR-LVA levels is high, allowing TetR-LVA to bind the promoter P_{tetO} and reduce LuxR expression, thereby arresting the transcriptional interference formed by RNAPs transcribing from the reverse promoter P_{lux}. Reducing the strength of feedforward loop results in restoring the maximum activity of P_{lacO} at high IPTG levels. At low IPTG concentrations, TetR-LVA levels are not sufficient to bind P_{tetO} to repress LuxR expression. Thus, AHL-LuxR complexes activate reverse promoter P_{lux} and induce transcriptional interference, which in turn increase the strength of the feedforward loop. The mCherry gene engineered downstream to LuxR under control of the same promoter P_{tetO}, acted as an indicator of P_{tetO} activity and subsequently as indicator of LuxR levels. Thus, at low IPTG concentrations, LuxR and mCherry levels were high whereas at high IPTG concentrations, LuxR and mcherry levels were low. Overall, the combination of transcriptional interference with inverting switch improved the activity of P_{lacO} (Fig. S19C).

3.3 The native specific promoter P_{LhrtO}

Hemin or heme-group containing molecule, is a type of molecules commonly found in blood. A synthetic circuit sensitive for heme was transformed into bacterial cells, where heme-inducible promoter P_{LhrtO} was used as forward promoter and AHL-inducible promoter P_{lux} was used as reverse promoter engineered downstream to gene *gfp*. Heme enters bacterial cells through outer membrane protein ChuA and binds with the transcriptional repressor HrtR to form HrtR-heme complex, which is released from heme-inducible promoter P_{LhrtO} allowing the expression of genes that are regulated (Mimee et al., 2018). AHL binds with transcriptional activators LuxR, forming AHL- LuxR complexes, which bind reverse promoter P_{lux}. The transcriptional repressor HrtR and outer membrane protein ChuA were constitutively expressed. LuxR was placed under the control of P_{tetO}, which functioned as a constitutive promoter when TetR wasn't included in the circuit. The reverse promoter regulates the forward promoter through antisense transcription, in which one transcriptional unit interferes with the second transcriptional unit.

Overnight grown bacterial cells containing and maintaining the circuit under test were diluted 100 times in the morning into fresh LB media and incubated for 0.5h before the induction with heme and additional inducers. Fluorescence was measured 4h after induction with heme.

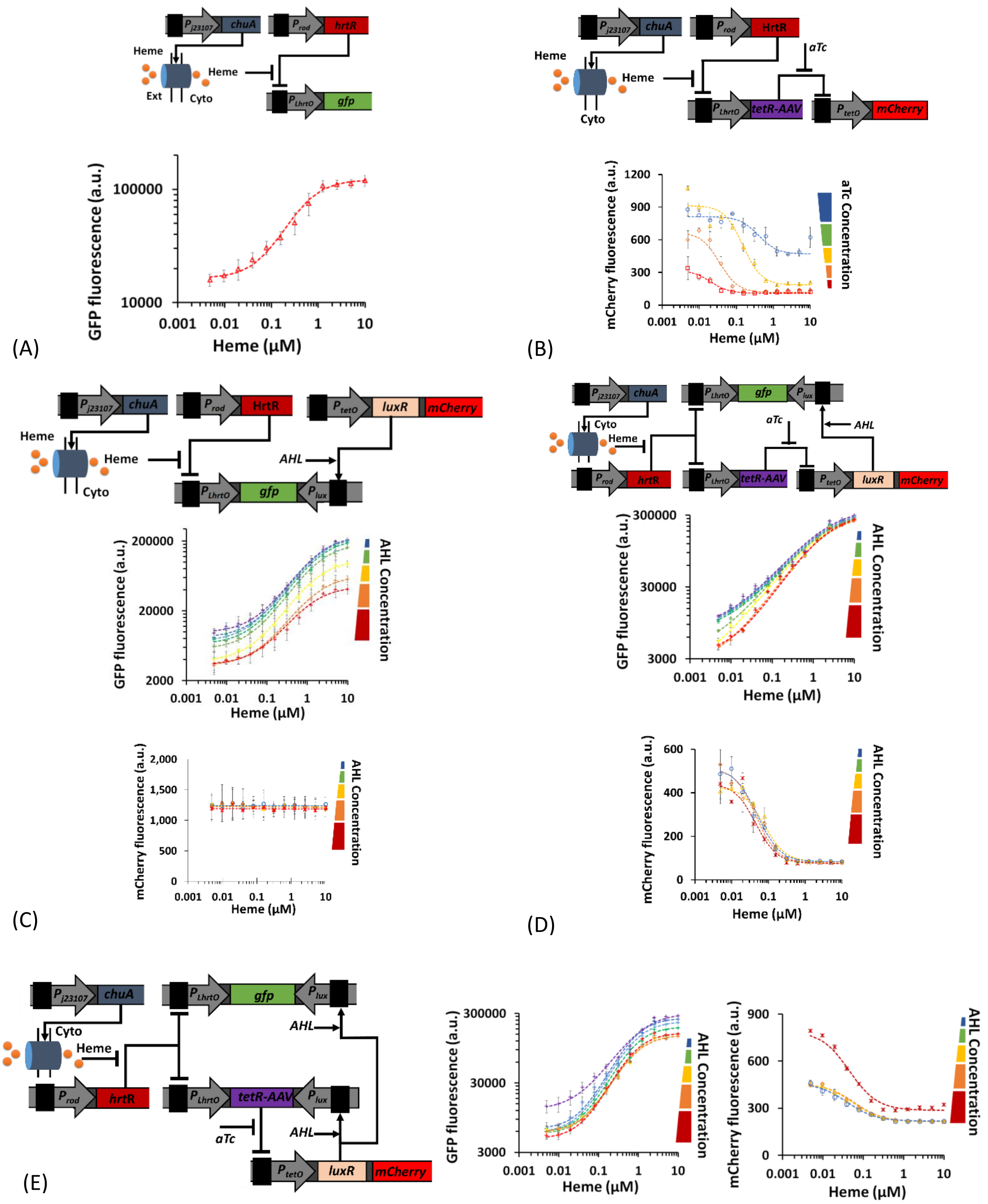
(A)
(B)
(C)
(D)
(E)
Heme
Ext
Cyto
chuA
hrtR
HrtR
gfp
tetR-AAV
mCherry
luxR
aTc
AHL
GFP fluorescence (a.u.)
mCherry fluorescence (a.u.)
Heme (µM)
aTc Concentration
AHL Concentration

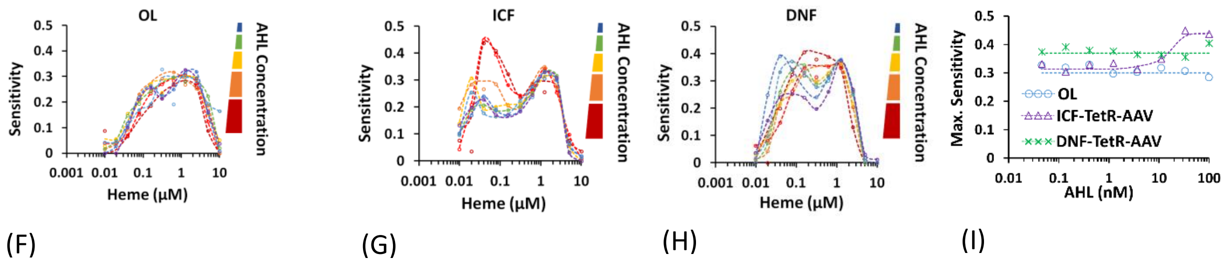

Fig. S20. Heme biosensor circuits implemented based on antisense transcription and sensitivity analyses for the circuits. (A)-(D): The upper panel contains the scheme for the synthetic circuit encompassing the promoter under test P_{PUT} and the lower panel shows the measured fluorescence for the tested circuit. (E): The left panel contains the scheme for the synthetic circuit encompassing the promoter under test P_{PUT} and the right panel shows the measured fluorescence for the tested circuit. (F-H): The sensitivity analysis of OL, ICF-TetR-AAV and DNF-TetR-AAV circuits with schematic diagrams described in C, D and E, respectively. (I) Maximum sensitivity for OL, ICF-TetR-AAV and DNF-TetR-AAV circuits. The results were calculated based on equation S3 (Flow cytometry data for building this figure is described in Fig. S39-S46).

3.3.1 Heme-sensing system. The expression of GFP is controlled by heme-inducible promoter P_{LhrtO} (Fig. S20A).

3.3.2 OL: The higher the AHL concentrations, the larger the inhibition from antisense transcription. The effect of antisense transcription due to this specific design could be detected by the reduction of both minimum and maximum promoter activities of P_{LhrtO}. LuxR is constitutively expressed and remained constant through different concentrations of heme at different concentrations of AHL as can be seen by mCherry levels (Fig. S20C).

3.3.3 Heme-aTc inverting switch (TetR-AAV): The activity of P_{tetO} represented by mCherry level in response to heme can be adjusted under various concentrations of aTc. At intermediate aTc concentrations, mCherry level is repressed as heme concentration increases from low to high (the yellow curve). At high aTc concentrations, more aTc-TetR-AAV complexes are formed, which are released from promoter P_{tetO} allowing transcription of mCherry. The AAV-degradation tag is responsible for reducing TetR expression levels. An optimal concentration of anhydrotetracycline (aTc) was deduced to give the highest ON/OFF ratio to work with in the ICF and DNF circuits (explained below in 3.3.4 and 3.3.5). If no significant differences in mCherry levels were observed between the various concentrations of aTc then no aTc was added in the ICF and DNF circuits. The mCherry gene was later replaced by LuxR-mCherry operon to be integrated in ICF and DNF circuits (Fig. S20B).

3.3.4 ICF-TetR-AAV: The heme-inducible promoter P_{LhrtO} was regulated by adjusting between its minimum expression level (basal level) and its maximum expression level (the ON state). The basal level was controlled through the activity of reverse promoter P_{lux} induced by AHL-LuxR complex. Whereas the maximum expression level was controlled by TetR-AAV protein, which represses the expression of LuxR. To regain the maximum promoter activity level of P_{LhrtO} that was reduced in OL (see 3.3.2) we engineered transcriptional repressor TetR-AAV under the control of P_{LhrtO} to obtain high TetR-AAV levels when heme concentrations are high. In this way, TetR-AAV bind promoter P_{tetO} and inhibit LuxR expression, thereby decreasing the antisense transcription formed by RNAPs from the reverse promoter P_{lux} and reducing the strength of feedforward loop. Thus, the maximum activity of P_{LhrtO} is restored at high heme levels. At low heme concentrations, TetR-AAV levels are

not sufficient to bind P_{tetO} to repress LuxR expression, therefore AHL-LuxR complexes bind to reverse promoter P_{lux} and activate antisense transcription, which in turn increase the strength of the feedforward loop. The mCherry gene engineered downstream to LuxR under control of the same promoter P_{tetO}, acted as an indicator of P_{tetO} activity and LuxR levels. Thus, at low heme concentrations LuxR and mCherry levels were high, whereas at high heme concentrations the LuxR and mcherry levels were low. Overall, the combination of transcriptional interference with inverting switch improved the activity of P_{LhrtO} (Fig. S20D).

3.3.5 DNF-TetR-AAV: The same as 3.3.4 but with reverse promoter P_{lux} incorporated downstream to TetR-AAV. In this way TetR-AAV was controlled not only by heme but also by AHL concentrations (Fig. S20E).

3.4 The native specific promoter P_{arsR}

A synthetic circuit sensitive to arsenic was introduced into bacterial cells. The circuit was implemented using antisense transcription, where the forward promoter was an arsenic-inducible promoter P_{arsR} and the reverse promoter was an AHL-inducible promoter $P_{lux,}$ engineered downstream to gene *gfp*. Transcriptional repressor arsR senses and binds with $AsNaO_2$ molecule, forming arsR-$AsNaO_2$ complex, which is then released from forward promoter P_{arsR} to allow the expression of genes that are regulated. (Wan et al., 2019). AHL binds with its transcriptional activators LuxR, creating AHL-LuxR complexes, which activate reverse promoter P_{lux} and initiate antisense transcription from P_{lux}. The transcriptional repressor arsR were constitutively expressed. LuxR was placed under the control of P_{tetO}, which functioned as a constitutive promoter when TetR wasn't included in the circuit. The reverse promoter P_{lux} downregulates the forward promoter through antisense transcription, in which one transcriptional unit interferes with the second transcriptional unit.

Overnight grown bacterial cells containing and maintaining the circuit under test were diluted 100 times in the morning into fresh LB media and supplemented with $AsNaO_2$ (Wan et al., 2019). The fluorescence was measured 5h after induction with $AsNaO_2$ and additional inducers.

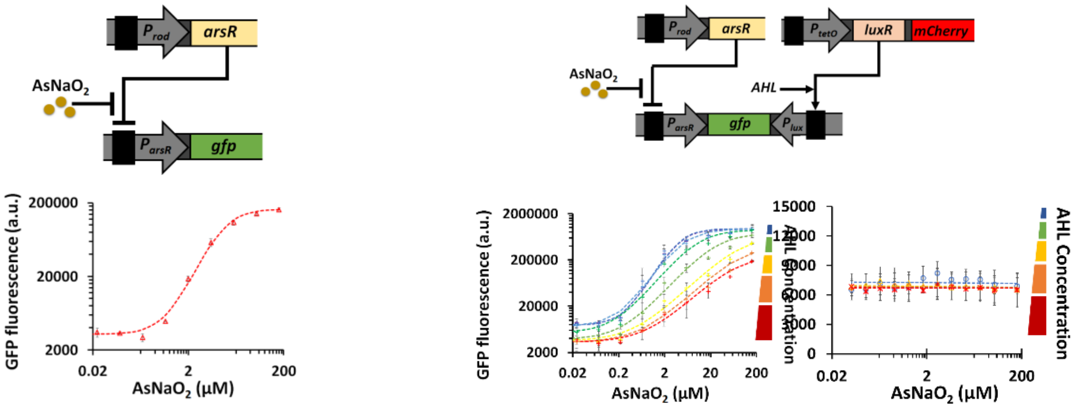

(A) (B)

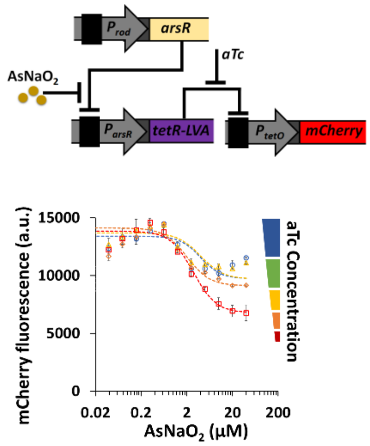
Prod arsR
aTc
AsNaO2
ParsR tetR-LVA
PtetO mCherry
mCherry fluorescence (a.u.)
15000
10000
5000
0
0.02 0.2 2 20 200
AsNaO2 (μM)
aTc Concentration

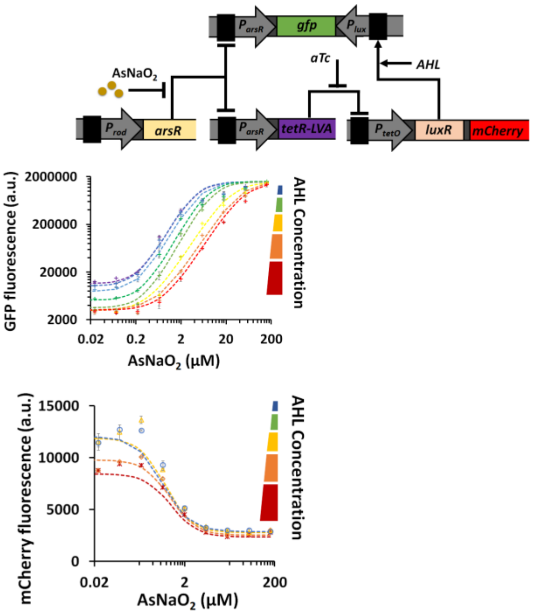
ParsR gfp Plux
aTc
AHL
AsNaO2
Prod arsR
ParsR tetR-LVA
PtetO luxR mCherry
GFP fluorescence (a.u.)
2000000
200000
20000
2000
0.02 0.2 2 20 200
AsNaO2 (μM)
AHL Concentration
mCherry fluorescence (a.u.)
15000
10000
5000
0
0.02 2 200
AsNaO2 (μM)
AHL Concentration

(C)

(D)

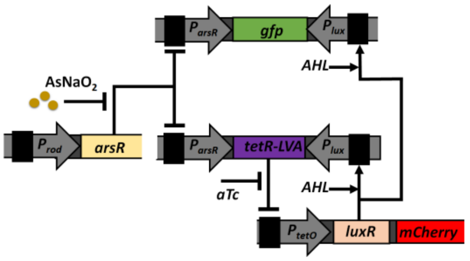
ParsR gfp Plux
AHL
AsNaO2
Prod arsR
ParsR tetR-LVA Plux
AHL
aTc
PtetO luxR mCherry

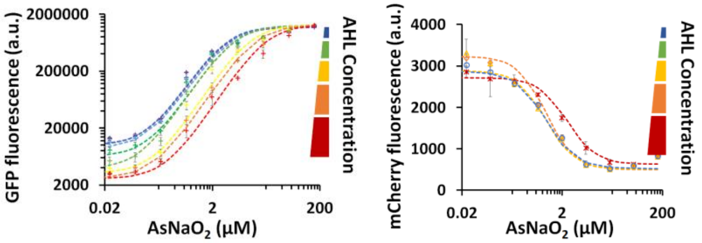
GFP fluorescence (a.u.)
2000000
200000
20000
2000
0.02 2 200
AsNaO2 (μM)
AHL Concentration
mCherry fluorescence (a.u.)
4000
3000
2000
1000
0
0.02 2 200
AsNaO2 (μM)
AHL Concentration

(E)

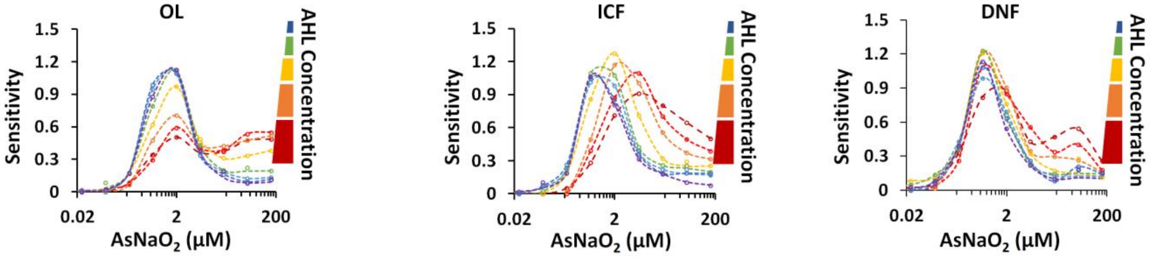
OL
ICF
DNF
Sensitivity
1.5
1.2
0.9
0.6
0.3
0
0.02 2 200
AsNaO2 (μM)
AHL Concentration

(F) (G) (H)

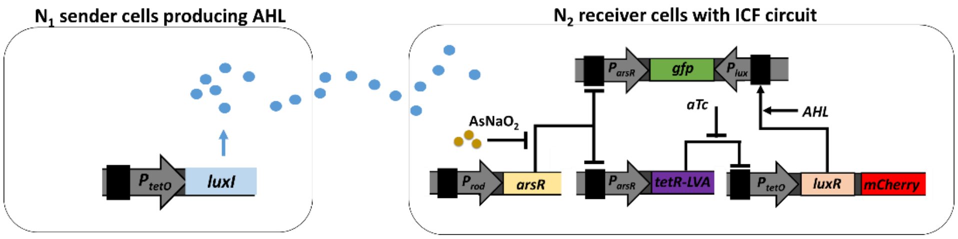
N1 sender cells producing AHL
PtetO luxI
N2 receiver cells with ICF circuit
ParsR gfp Plux
aTc
AHL
AsNaO2
Prod arsR
ParsR tetR-LVA
PtetO luxR mCherry

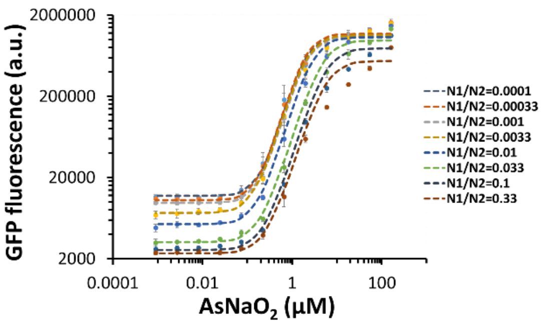

(I)

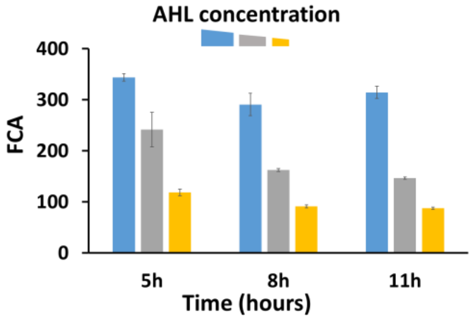

(J)

Fig. S21. Arsenic biosensing circuits and sensitivity analyses implemented based on antisense transcription. (A)-(D): The upper panel contains the scheme for the synthetic circuit encompassing the P_{arsR} as P_{PUT} and the lower panel shows the measured fluorescence for the tested circuit. (E): The left panel contains the scheme for the synthetic circuit encompassing the P_{arsR} as P_{PUT} and the right panel shows the measured fluorescence for the tested circuit. (F-H): The analysis sensitivity of OL, ICF-TetR-LVA and DNF-TetR-LVA circuits with schematic diagrams described in B, D and E, respectively. The results were calculated based on equation S3. (I) Cell-cell communication experiment between sender cells producing AHL and receiver cells containing the ICF circuit. The upper panel contains the scheme of the circuit and the lower panel shows the measured fluorescence for the communication circuit. The N_1 sender cells produce AHL quorum-sensing molecule by constitutively expressing *luxI* gene. N_1 strain contains two additional plasmids, we call them empty plasmids because they don't contain any part but used only to match the antibiotics used for N_2 strain. The N_2 strain contains the P_{arsR}-based ICF circuit. The various concentrations of AHL were obtained by mixing the strains N_1 and N_2 in different ratios. As the ratio N_1/N_2 increases the AHL concentration also is increased**.** This experiment demonstrates how ICF circuit can operate without the need of adding AHL externally. (J) Kinetics experiment made at several time points that demonstrates that ICF circuit can be optimized by AHL and operate for extended periods of time (Flow cytometry data for building this figure is described in Fig. S47-S53).

3.4.1 Arsenic-sensing system from *E. coli*. The expression of GFP is controlled by arsenic-inducible promoter P_{arsR} (Fig. S21A).

3.4.2 OL: The higher the AHL concentrations, the stronger is the strength of inhibition due to antisense transcription. The antisense transcription originated from this special orientation could be detected by the reduction of both minimum and maximum promoter activity of P_{arsR}. LuxR is constitutively expressed through different concentrations of $AsNaO_2$ and AHL which can be seen by constant mCherry levels at different AHL concentrations (Fig. S21B).

3.4.3 $AsNaO_2$-aTc inverting switch: At intermediate aTc concentrations, mCherry level becomes repressed as $AsNaO_2$ concentration increases from low to high. At high aTc concentrations, more aTc-TetR-LVA complexes are formed, which are released from promoter P_{tetO}, allowing transcription of mCherry. The LVA-degradation tag is responsible for reducing TetR-LVA expression levels. An optimal

concentration of anhydrotetracycline(aTc) was deduced to give the highest ON/OFF ratio to work with in the ICF and DNF circuits (explained below in 3.4.4 and 3.4.5). If no significant differences in mCherry levels were observed between the various concentrations of aTc then no aTc was added in the ICF and DNF circuits. The mCherry gene was later replaced by LuxR-mCherry operon to be integrated with the antisense transcription in ICF and DNF circuits (Fig. S21C).

3.4.4 ICF-TetR-LVA based on antisense transcription. The arsenic-inducible promoter P_{arsR} was regulated by adjusting between its minimum expression level (basal level) and its maximum expression level (the ON state). The basal level was controlled through the activity of P_{lux} induced by AHL-LuxR complex. Whereas the maximum expression level was controlled by TetR-LVA protein, which represses the expression of LuxR. To regain the maximum promoter activity level of P_{arsR} that was reduced in OL (see 3.4.2), we engineered transcriptional repressor TetR-LVA under the control of P_{arsR} to obtain high TetR-LVA levels when $AsNaO_2$ concentration is high. In this way, high levels of TetR-LVA binds promoter P_{tetO} and inhibit LuxR expression. The decreasing level of LuxR reduces the antisense transcription formed by RNAPs transcribing from the reverse promoter P_{lux}, as well as the strength of feedforward loop, thereby restoring the maximum activity of P_{arsR} at high $AsNaO_2$ levels. At low $AsNaO_2$ concentrations, TetR-LVA levels are not sufficient to bind P_{tetO} to repress LuxR expression, therefore AHL-LuxR complexes bind to reverse promoter P_{lux} to induce antisense transcription, which in turn increase the strength of the feedforward loop. The mCherry gene engineered downstream to LuxR under control of the same promoter P_{tetO}, acted as an indicator of P_{tetO} activity and LuxR levels. In this way, at low $AsNaO_2$ concentrations LuxR and mcherry levels were high, whereas at high $AsNaO_2$ concentrations the LuxR and mcherry levels were low. Overall, the combination of transcriptional interference with inverting switch improved the activity of P_{arsR} (Fig. S21D).

3.4.5 DNF-TetR-LVA based on antisense transcription. The same as in D but with reverse promoter P_{lux} incorporated downstream to TetR-LVA. In this way, TetR-LVA was controlled not only by $AsNaO_2$ but also by AHL concentrations (Fig. S21E).

3.5 The native stress promoter P_{katG}

Hydrogen peroxide, H_2O_2, cause oxidative stress to cells inflicting damage to proteins, nucleic acid, lipids and membranes. Upon exposure of the bacterial cells to H_2O_2 at least 30 antioxidant genes are induced when half of them being activated by transcriptional activator OxyR (Aslund et al., 2002; Belkin et al., 1996). A synthetic circuit sensitive to oxidative stress was inserted into bacterial cells. In this native stress circuit, the H_2O_2-inducible promoter P_{katG} was used as forward promoter and AHL-inducible promoter P_{lux} was used as the reverse promoter, comprising the transcriptional interference. H_2O_2 and AHL bind to their cognate transcriptional activators, OxyR and LuxR, generating complexes, which bind to the forward and reverse promoters, respectively. The transcriptional activator OxyR is constitutively expressed. LuxR was placed under the control of P_{tetO}, which functioned as a constitutive promoter where TetR wasn't included in the circuit .

Overnight grown bacterial cells containing and maintaining the circuit under test were diluted 50 times in the morning into fresh M9 minimal media and incubated for 3h before the induction with H_2O_2 and additional inducers if needed. H_2O_2 was added to the media every one hour for two times more, to enable 4 hours needed for response to AHL and aTc, since the H_2O_2 is degradated by a product of *KatG* gene

(Panek & Brian, 2004) and has a peak at about 50min followed by signal reduction (Barger et al., 2019; Belkin et al., 1996) . Fluorescence was measured 2.5h after the last induction.

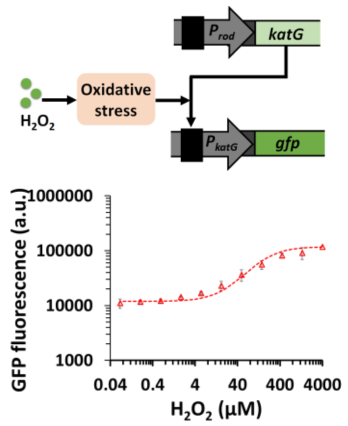

(A)

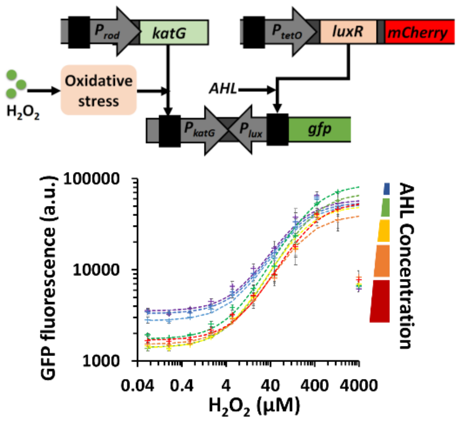

(B)

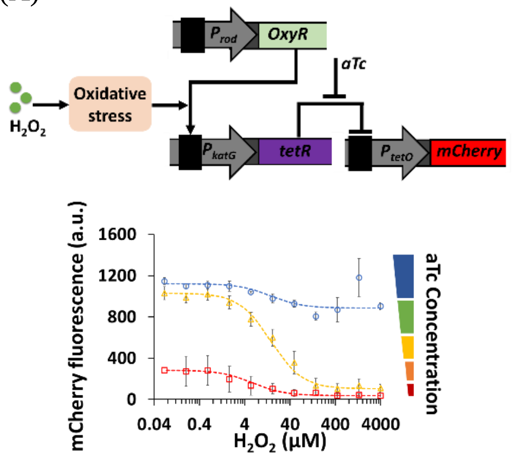

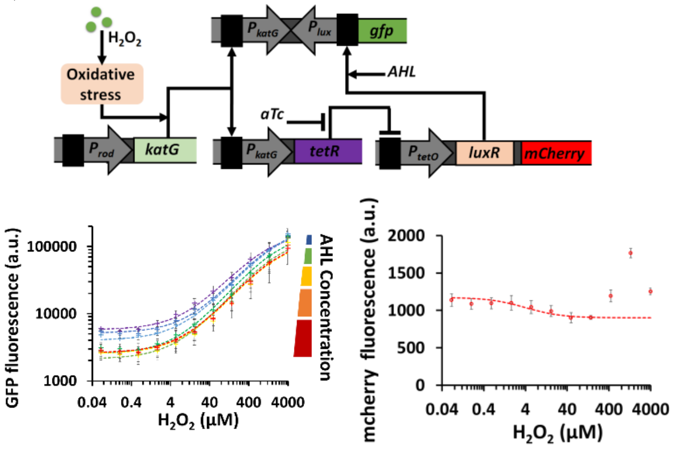

(D)

(C)

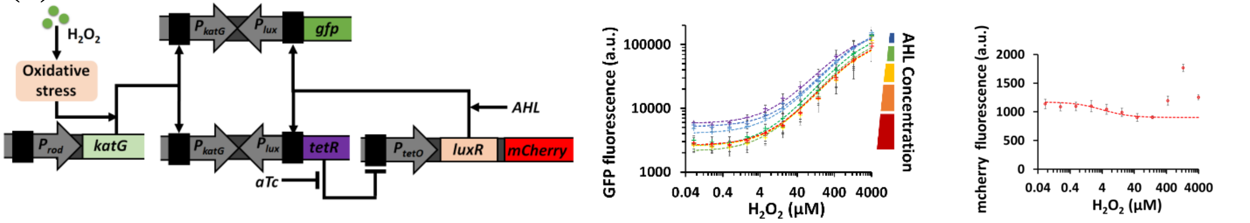

(E)

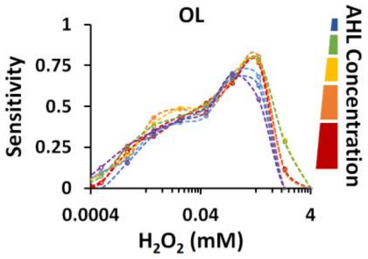

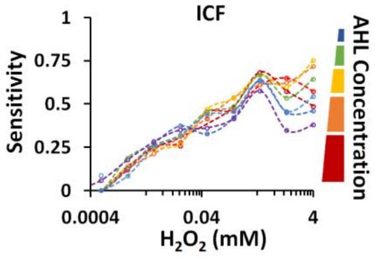

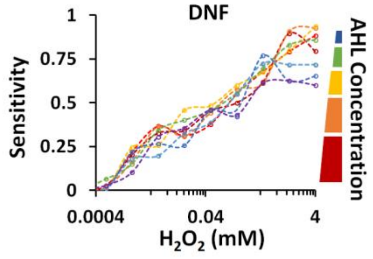

(F) (G) (H)

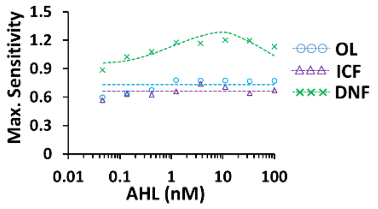

(I)

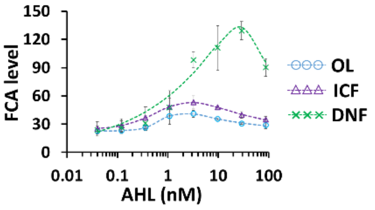

(J)

Fig. S22. KatG biosensing circuits based on transcriptional interference and corresponding sensitivity analyses. (A)-(D): The upper panel contains the scheme for the synthetic circuit encompassing the promoter under test P_{PUT} and the lower panel shows the measured fluorescence for the tested circuit. (E): The left panel contains the scheme for the synthetic circuit encompassing the promoter under test P_{PUT} and the right panel shows the measured fluorescence for the tested circuit. (F-H): The sensitivity analysis of OL, ICF and DNF circuits with schematic diagrams described in B, D and E. (I): Maximum sensitivity of OL, ICF and DNF circuits. The results were calculated based on equation S3. (J) FCA of OL, ICF and DNF circuits as function of F_S strengths, which are controlled by AHL levels (Flow cytometry data for building this figure is described in Fig. S54-S58).

3.5.1 H_2O_2-sensing system from *E. coli*. The expression of GFP is controlled by H_2O_2-inducible promoter P_{katG} (Fig. S22A).

3.5.2 OL: At high AHL concentrations, the transcription of GFP by RNAPs from forward promoter P_{katG} is inhibited by the reverse promoter P_{lux}, creating transcriptional interference that can be detected by the reduction of minimum and maximum promoter. The higher the AHL concentrations, the stronger is the strength of transcriptional inhibition (Fig. S22B).

3.5.3 H_2O_2-aTc inverting switch. At intermediate aTc concentrations, the mCherry level is repressed as concentration increases from low to high. At high aTc concentrations, more aTc-TetR complexes are formed, which are released from promoter $P_{tetO,}$ allowing transcription of mCherry. An optimal concentration of anhydrotetracycline (aTc) was deduced to give the highest ON/OFF ratio to work with in following circuits (explained below in 3.5.4 and 3.5.5). If no significant differences in mCherry levels were observed between the various concentrations of aTc then no aTc was added in the ICF and DNF circuits. The mCherry gene was later replaced by LuxR-mCherry operon to be integrated with the transcriptional interference in ICF and DNF circuits (Fig. S22C).

3.5.4 ICF-TetR. The H_2O_2-inducible promoter P_{katG} was regulated by adjusting between its minimum expression level (basal level) and its maximum expression level (the ON state). The basal level was controlled through the activity of P_{lux} induced by AHL-LuxR complex. Whereas the maximum expression level was controlled by TetR protein, which represses the expression of LuxR. To regain the maximum promoter activity level of P_{katG} that was reduced in OL (see 3.5.2), we designed the transcriptional repressor TetR to be under control of H_2O_2-inducible promoter $P_{katG,}$. At high H_2O_2 concentrations, the TetR levels are high, increasing the binding of TetR to P_{tetO} and reducing the LuxR levels, thereby inhibiting transcriptional interference from P_{lux}. At low H_2O_2 concentrations, TetR levels are not sufficient and therefore allow binding of AHL-LuxR complex with antisense promoter $P_{lux,}$ inducing transcriptional interference and inhibiting the transcription of P_{katG}. The mCherry gene engineered downstream to LuxR under control of the same promoter P_{tetO}, acted as an indicator of P_{tetO} activity and LuxR levels. In this way, at low H_2O_2 concentrations LuxR and mCherry levels were high, whereas at high H_2O_2 concentrations the LuxR and mCherry levels were low. LuxR levels exhibited high expression at high H_2O_2 concentration. The reason for this effect could be because the autofluorescence of the cells is enhanced when the bacterial cells are in stress (Surre et al., 2018) (Fig. S22D).

3.5.5 DNF-TetR: The same as in 3.5.4 with the addition of reverse promoter P_{lux} to control TetR levels. In this way TetR was controlled not only by H_2O_2 but also by AHL concentrations (Fig. S22E).

3.6 The native stress promoter P_{recA}

Nalidixic acid (NA) is an inhibitor of DNA gyrase enzyme required for DNA replication in living cells. Treatment with NA induces DNA damage through SOS response in which 30 genes are being de/activated upon autocleavage of the SOS-transcriptional repressor LexA by product of gene *recA* (Davidov et al., 2000; Vollmer et al., 1997). A synthetic circuit sensitive to SOS response was transformed into MG1655 *E. coli* cells since the strain 10-beta *E. coli* has mutations in *recA* gene. In this circuit, NA activates indirectly promoter $P_{recA,}$ which was used as a forward promoter. AHL-inducible promoter P_{lux} was used as reverse promoter engineered either upstream to *gfp* gene for transcriptional interference or downstream to *gfp* gene for antisense transcription. AHL binds to its transcriptional activators LuxR, creating AHL-LuxR complexes which bind to reverse promoter P_{lux}. A gene *lexA* was used as transcriptional repressor (Little et al., 1981). The transcriptional repressor LexA was constitutively expressed. LuxR was placed under the control of P_{tetO}, which functioned as a constitutive promoter when TetR wasn't included in the circuit.

Overnight grown bacterial containing and maintaining the circuit under test cells were diluted 50 times in the morning into fresh M9 minimal media and incubated for 3.5h before the induction with NA and additional inducers. Fluorescence was measured 3.5h after the induction.

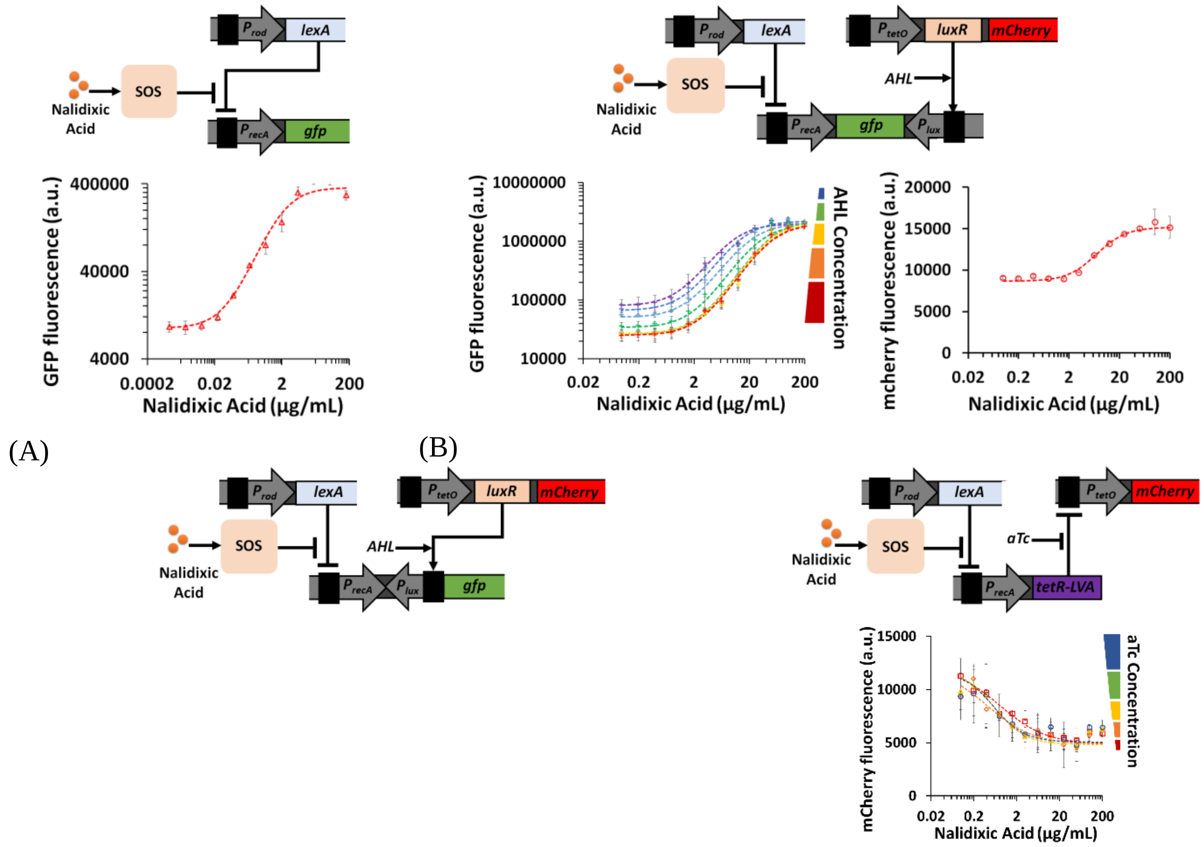

(D)

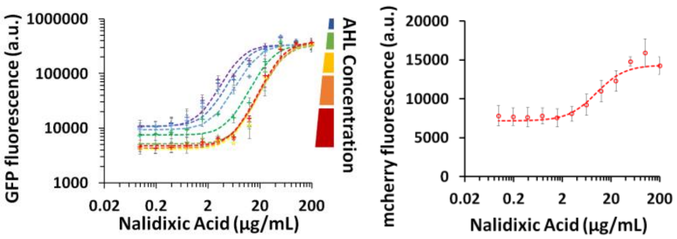

(C)

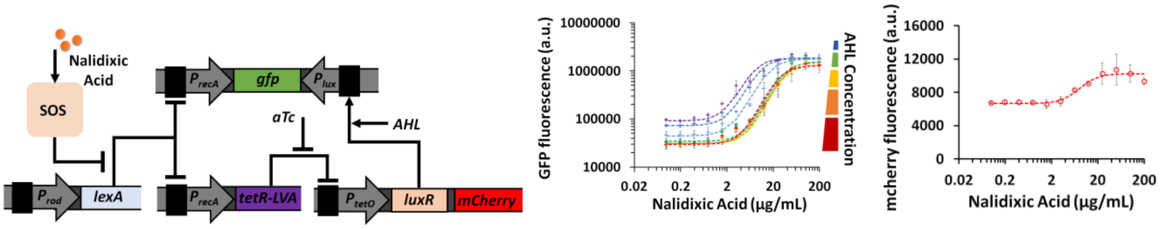

(E)

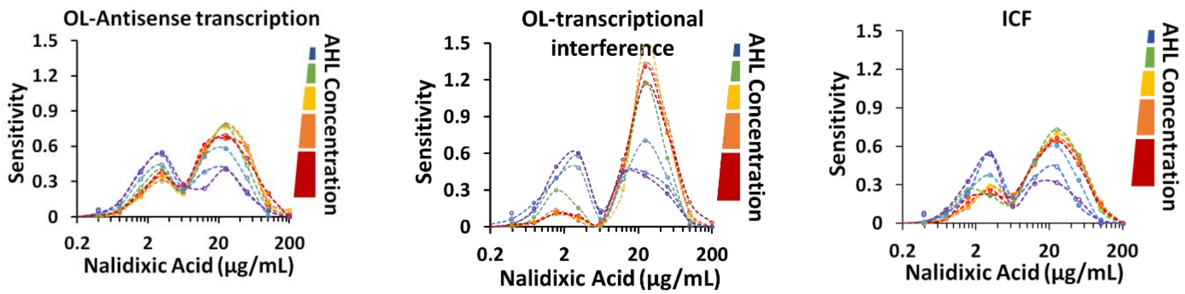

(F)

(G)

(H)

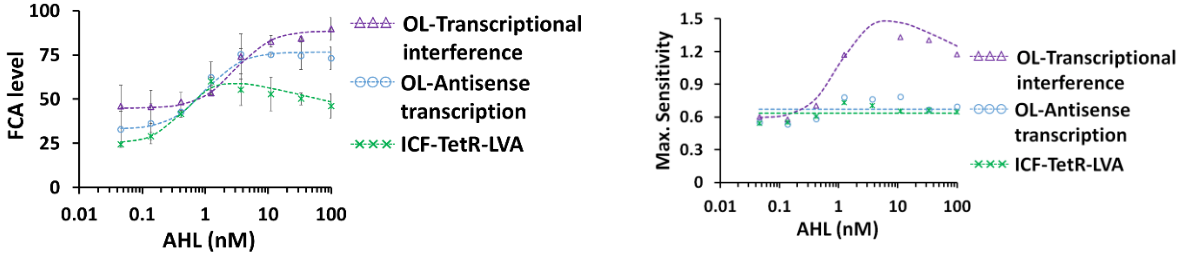

(I)

(J)

Fig. S23. P_{recA} biosensing circuits and corresponding sensitivity analyses. (A)-(D): The upper panel contains the scheme for the synthetic circuit encompassing the promoter under test P_{PUT} and the lower panel shows the measured fluorescence for the tested circuit. (E): The left panel contains the scheme for the synthetic circuit encompassing the promoter under test P_{PUT} and the right panel shows the measured fluorescence for the tested circuit. (F-H): The sensitivity analysis of antisense transcription based OL, transcriptional interference based OL and ICF-TetR-LVA circuits with schematic diagrams described in B, C and E, respectively. The results were calculated based on equation S3. (I) FCA for antisense transcription based OL circuit, transcriptional Interference based OL circuit, and ICF-TetR-LVA circuit as function of F_S strengths. (J) Maximum sensitivity for antisense transcription based OL circuit, transcriptional Interference based OL circuit, and ICF-TetR-LVA circuit were calculated based on equations S3 (Flow cytometry data for building this figure is described in Fig. S59-S63).

3.6.1 NA-sensing system from *E. coli*. The expression of GFP is controlled by NA-inducible promoter P_{recA} (Fig. S23A).

3.6.2 Antisense transcription based OL circuit. At high AHL concentrations, the transcription of P_{recA} promoter is inhibited by antisense RNA transcribed from $P_{lux,}$ which resulted in basal level reduction while keeping the maximum activity of the forward promoter P_{recA} intact (Fig. S23B).

3.6.3 Transcriptional interference based OL circuit. At high AHL concentrations, the transcription of P_{recA} promoter is inhibited by transcriptional interference from $P_{lux,}$ which resulted in basal level reduction while keeping the maximum activity of the forward promoter P_{recA} intact (Fig. S23C).

3.6.4 NA-aTc inverting switch. At low aTc concentrations, at low NA concentrations mcherry levels were high whereas at high NA concentrations the mcherry levels were low. The LVA-degradation tag is responsible for reducing TetR-LVA expression levels and for decreasing the ON/OFF ratio of P_{tetO}. An optimal concentration of anhydrotetracycline(aTc) was deduced to give the highest ON/OFF ratio to work with in following circuits (explained in 3.6.5). If no significant differences in mCherry levels were observed between the various concentrations of aTc then no aTc was added in the ICF circuit. The mCherry gene was later replaced by LuxR-mCherry operon to be integrated with the transcriptional interference in ICF circuit (Fig. S23D).

3.6.5 ICF-TetR-LVA. The NA-inducible promoter P_{recA} was regulated by adjusting between its minimum expression level (basal level) and its maximum expression level (the ON state). The basal level was controlled through P_{lux} activity inducible by AHL. Whereas the maximum expression level was controlled by TetR-LVA protein, which represses the expression of LuxR. The transcriptional repressor TetR-LVA was under the control of P_{recA}. The mCherry gene engineered downstream to LuxR under control of the same promoter P_{tetO}, acted as an indicator of P_{tetO} activity and LuxR levels. LuxR levels exhibited high expression at high NA concentration. The reason for this effect could be because the autofluorescence of the cells is enhanced when the bacterial cells are in stress (Surre et al., 2018) (Fig. S23E).

4. Experimental data from Flow cytometry

This section contains flow cytometry data for one representative experiment which was independently repeated for two more times.

4.1 Experimental data for synthetic promoter P_{BADsyn}

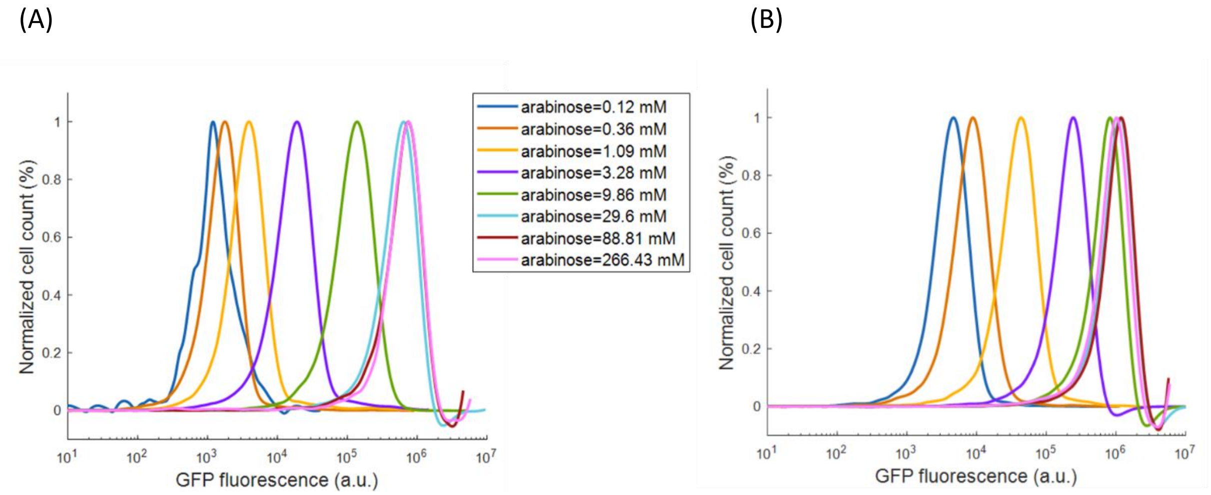

Fig. S24. Flow cytometry data for GFP over bacterial cells population. (A)containing the wild-type topology of P_{BAD}. (B)containing the wild-type topology of synthetic P_{BADsyn}. This experimental data was used for generating Fig. 4C.

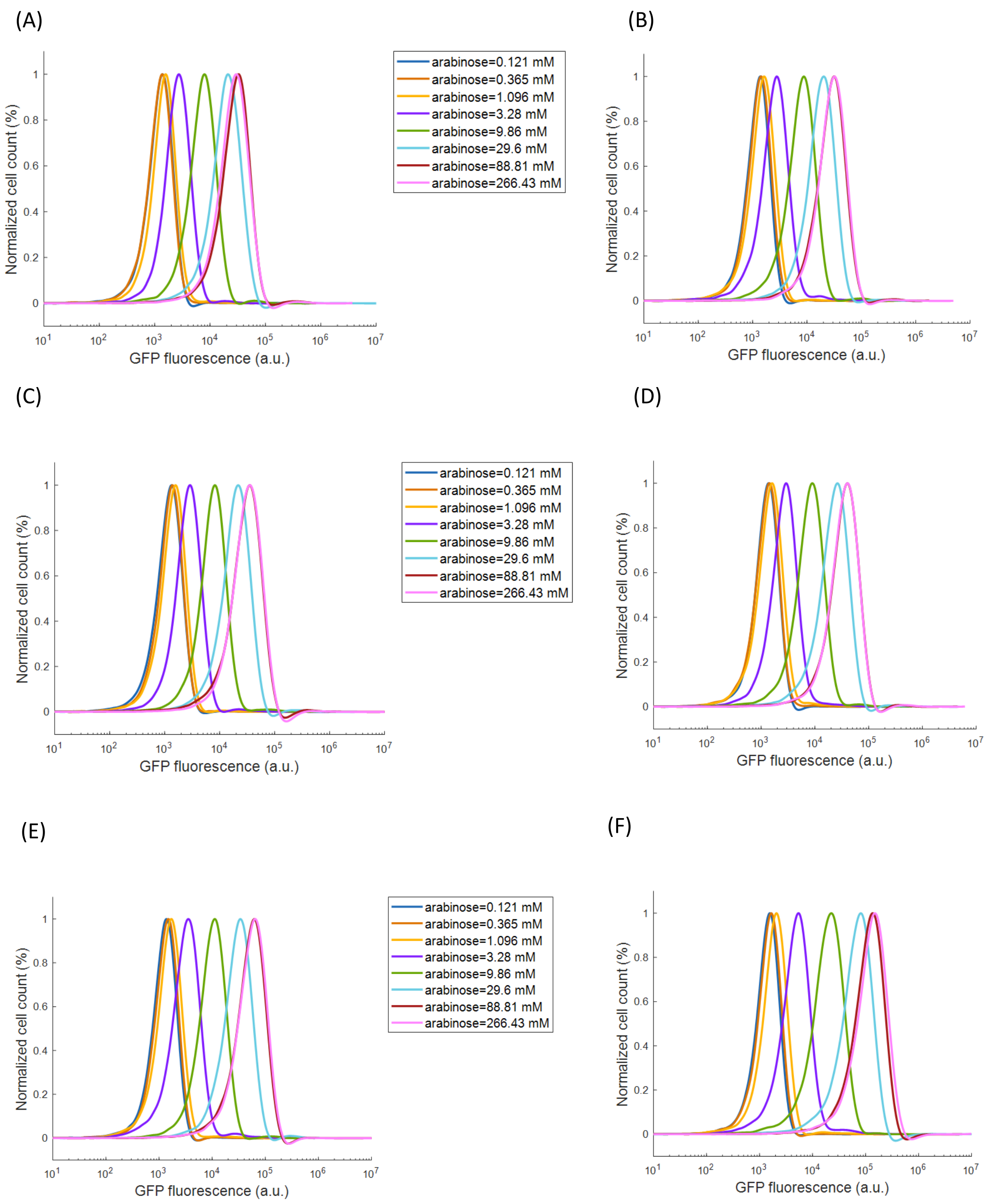

(G)

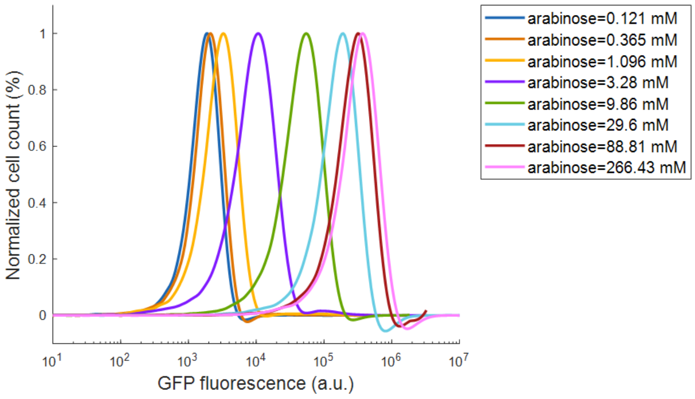
Normalized cell count (%)
GFP fluorescence (a.u.)
arabinose=0.121 mM
arabinose=0.365 mM
arabinose=1.096 mM
arabinose=3.28 mM
arabinose=9.86 mM
arabinose=29.6 mM
arabinose=88.81 mM
arabinose=266.43 mM

(H)

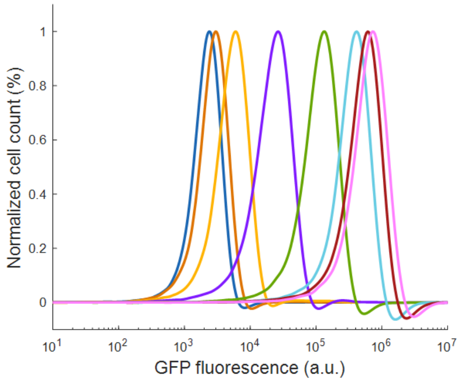
Normalized cell count (%)
GFP fluorescence (a.u.)

(I)

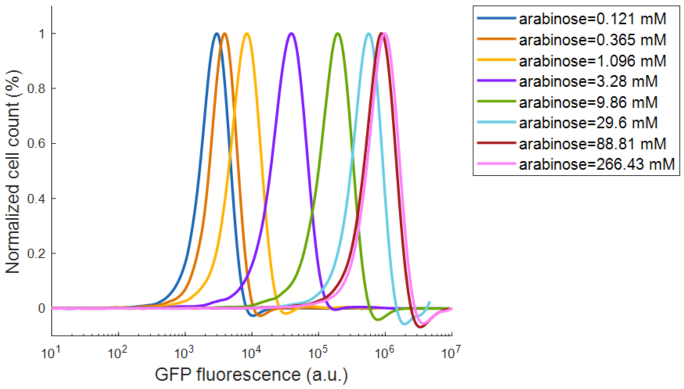
Normalized cell count (%)
GFP fluorescence (a.u.)
arabinose=0.121 mM
arabinose=0.365 mM
arabinose=1.096 mM
arabinose=3.28 mM
arabinose=9.86 mM
arabinose=29.6 mM
arabinose=88.81 mM
arabinose=266.43 mM

(J)

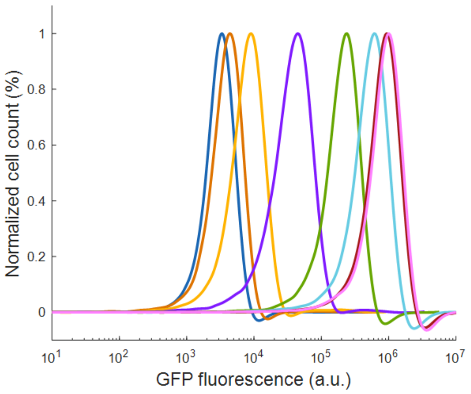
Normalized cell count (%)
GFP fluorescence (a.u.)

(K)

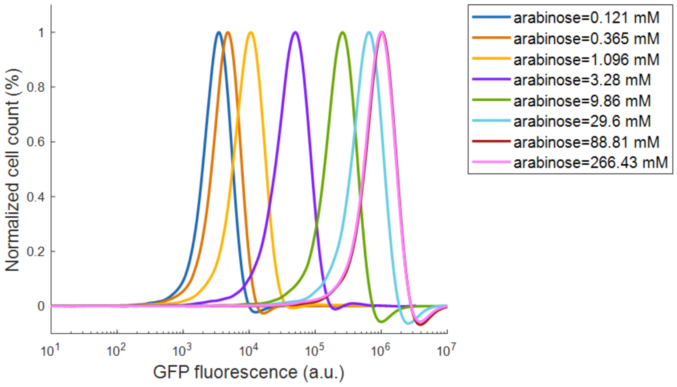
Normalized cell count (%)
GFP fluorescence (a.u.)
arabinose=0.121 mM
arabinose=0.365 mM
arabinose=1.096 mM
arabinose=3.28 mM
arabinose=9.86 mM
arabinose=29.6 mM
arabinose=88.81 mM
arabinose=266.43 mM

(L)

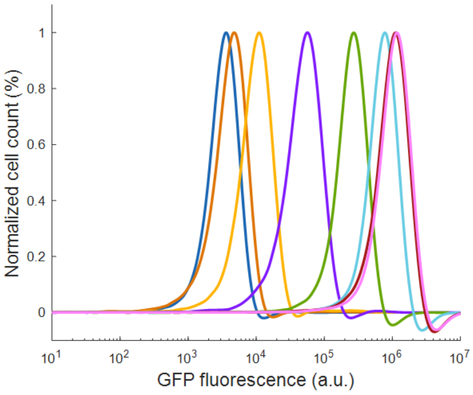
Normalized cell count (%)
GFP fluorescence (a.u.)

(M)

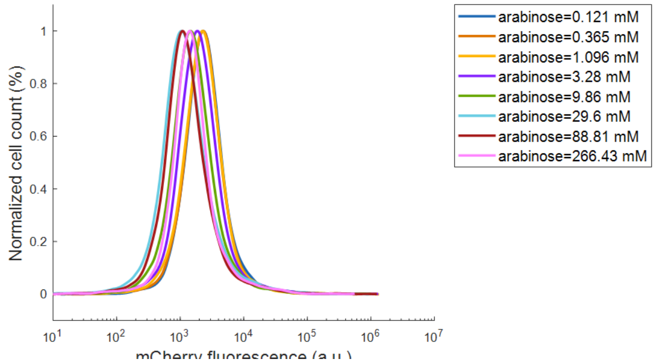

(N)

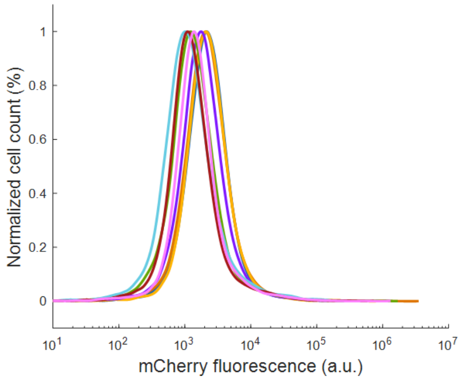

(O)

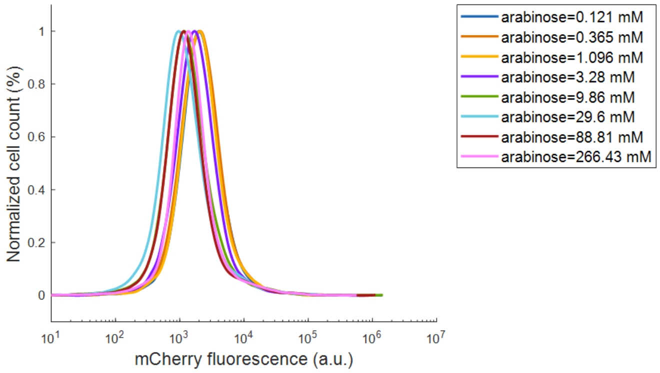

(P)

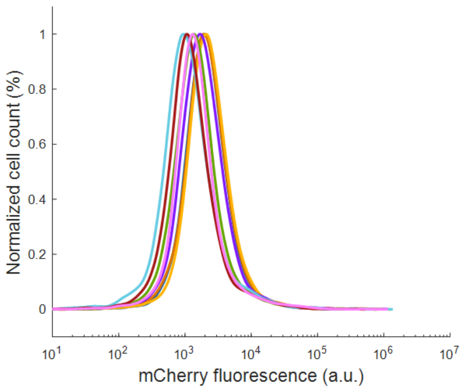

Fig. S25. Flow cytometry data for GFP over bacterial cells population containing OL design of P_{BADsyn}. (A) [AHL]=0.5 μM, (B)[AHL]=0.25 μM, (C)[AHL]=0.125 μM, (D)[AHL]=0.0625 μM, (E)[AHL]=0.0313 μM, (F)[AHL]=0.0156 μM, (G)[AHL]=0.0078 μM, (H)[AHL]=0.0039 μM, (I)[AHL]=0.002 μM, (J)[AHL]=0.001 μM, (K)[AHL]=0.0005 μM, (L)[AHL]=0.0002 μM. (M-P) Flow cytometry data for mCherry over bacterial cells population containing OL design. (M) [AHL]=0.0078 μM, (N) [AHL]=0.0039 μM, (O) [AHL]=0.00098 μM, (P) [AHL]=0.0005 μM. This experimental data was used for generating Fig. 6A and Fig.S17A.

(A)

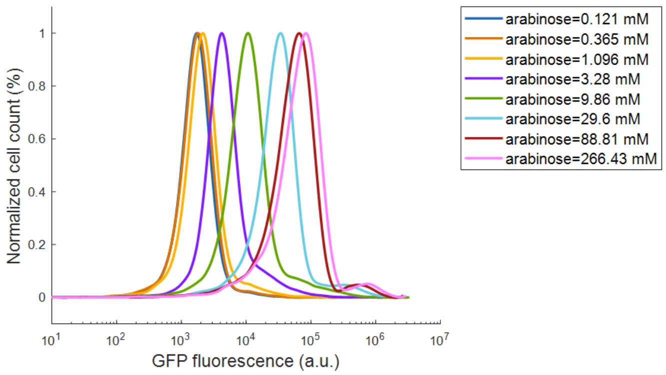

(B)

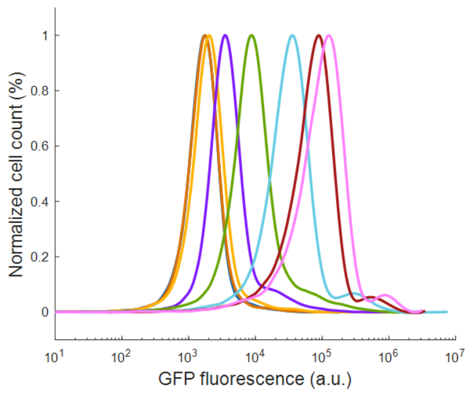

(C)

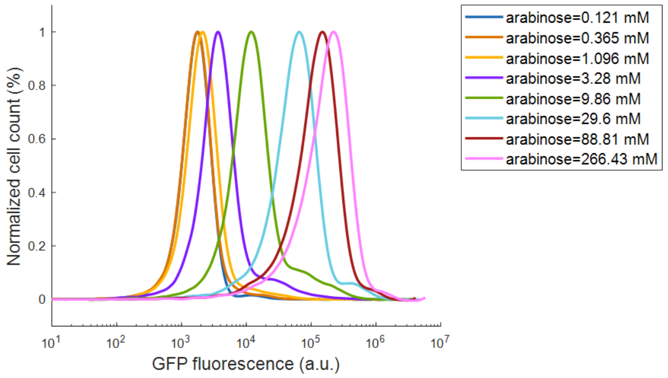
Normalized cell count (%)
GFP fluorescence (a.u.)
arabinose=0.121 mM
arabinose=0.365 mM
arabinose=1.096 mM
arabinose=3.28 mM
arabinose=9.86 mM
arabinose=29.6 mM
arabinose=88.81 mM
arabinose=266.43 mM

(D)

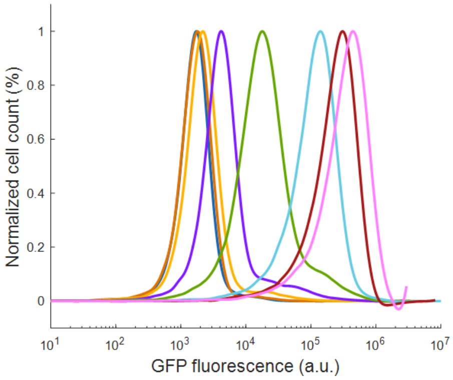
Normalized cell count (%)
GFP fluorescence (a.u.)

(E)

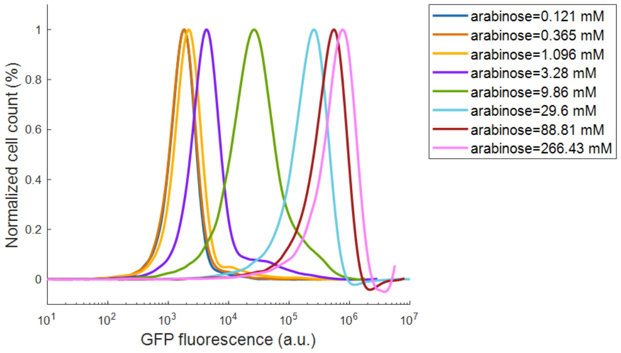
Normalized cell count (%)
GFP fluorescence (a.u.)
arabinose=0.121 mM
arabinose=0.365 mM
arabinose=1.096 mM
arabinose=3.28 mM
arabinose=9.86 mM
arabinose=29.6 mM
arabinose=88.81 mM
arabinose=266.43 mM

(F)

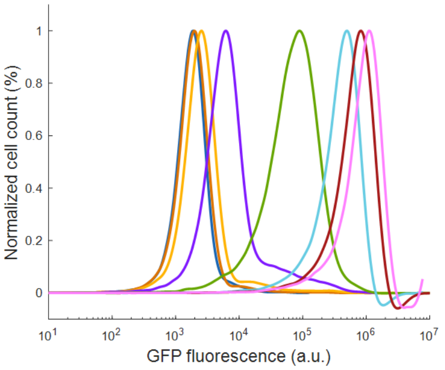
Normalized cell count (%)
GFP fluorescence (a.u.)

(G)

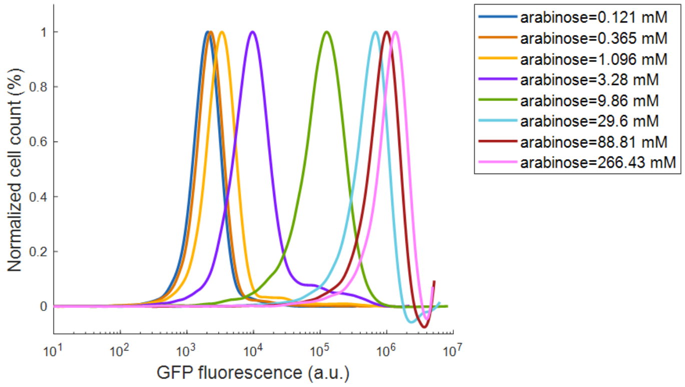
Normalized cell count (%)
GFP fluorescence (a.u.)
arabinose=0.121 mM
arabinose=0.365 mM
arabinose=1.096 mM
arabinose=3.28 mM
arabinose=9.86 mM
arabinose=29.6 mM
arabinose=88.81 mM
arabinose=266.43 mM

(H)

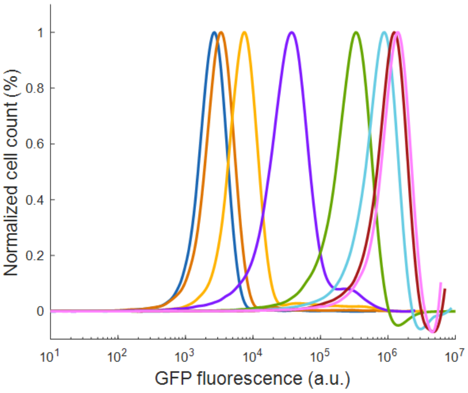
Normalized cell count (%)
GFP fluorescence (a.u.)

(I)

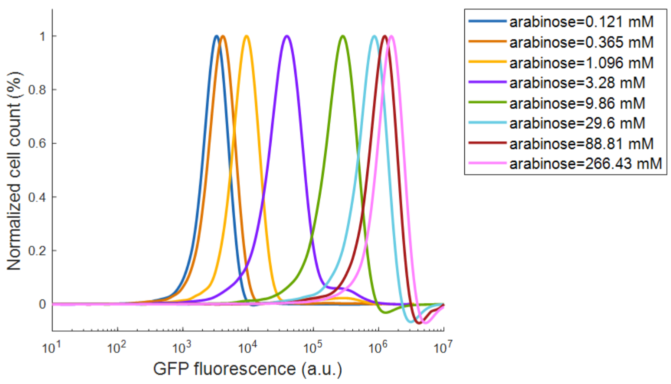

(J)

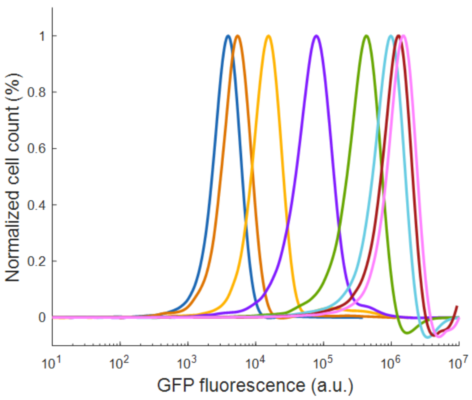

(K)

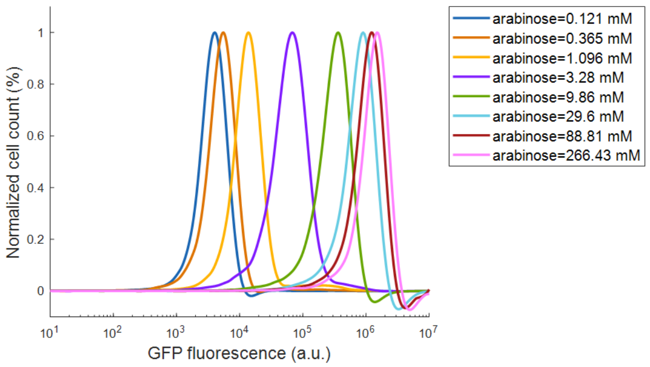

(L)

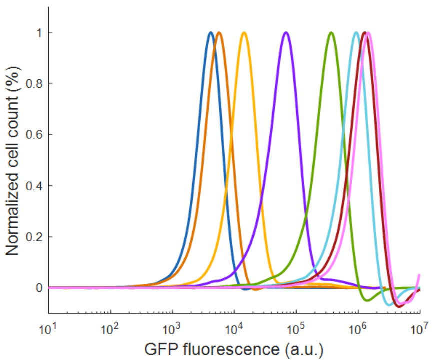

Fig. S26. Flow cytometry data for GFP over bacterial cells population containing ICF-TetR-LVA design of P_{BADsyn}. (A) [AHL]=0.5 μM, (B)[AHL]=0.25 μM, (C)[AHL]=0.125 μM, (D)[AHL]=0.625 μM, (E)[AHL]=0.0313 μM, (F)[AHL]=0.0156 μM, (G)[AHL]=0.0078 μM, (H)[AHL]=0.0039 μM, (I)[AHL]=0.002 μM, (J)[AHL]=0.001 μM, (K)[AHL]=0.0005 μM, (L)[AHL]=0.0002 μM. This experimental data was used for generating Fig. 6B and Fig.S17D.

(A)

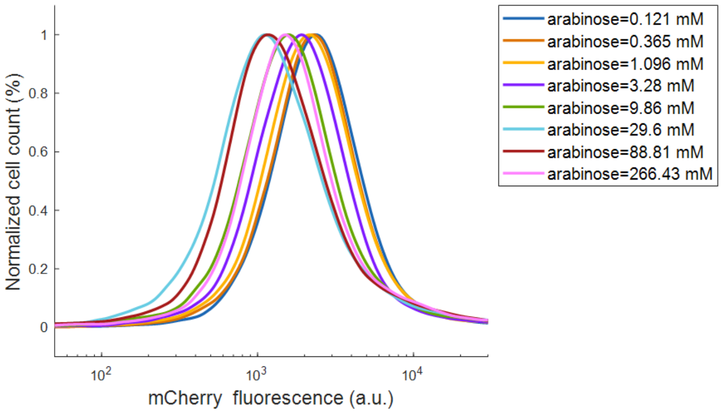

(B)

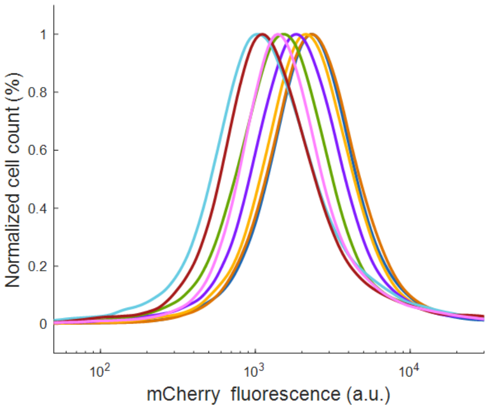

(C) (D)

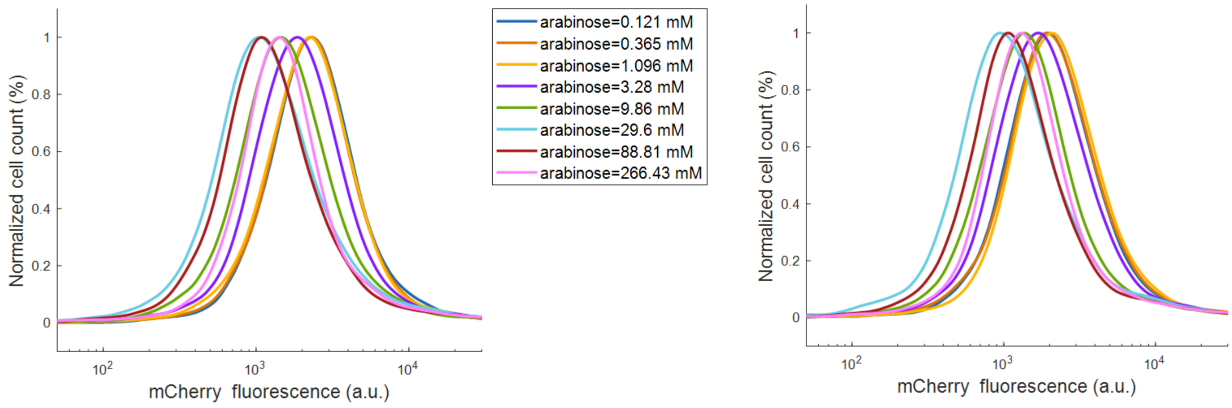

Fig. S27. Flow cytometry data for mCherry over bacterial cells population containing ICF-TetR-LVA design of P_{BADsyn}. (A) [AHL]=0.0313 μM, (B) [AHL]=0.0156 μM, (C) [AHL]=0.0078 μM, (D) [AHL]=0.0005 μM. This experimental data was used for generating Fig. 6B and Fig.S17D.

(A) (B)

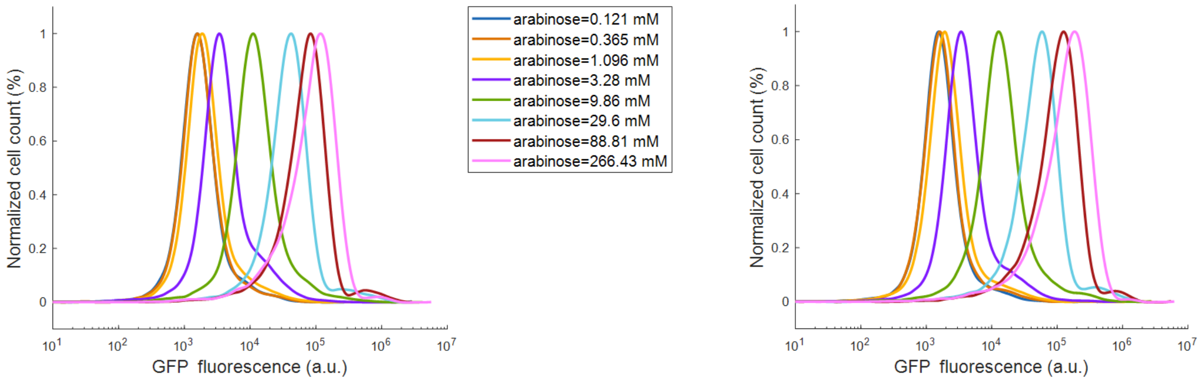

(C) (D)

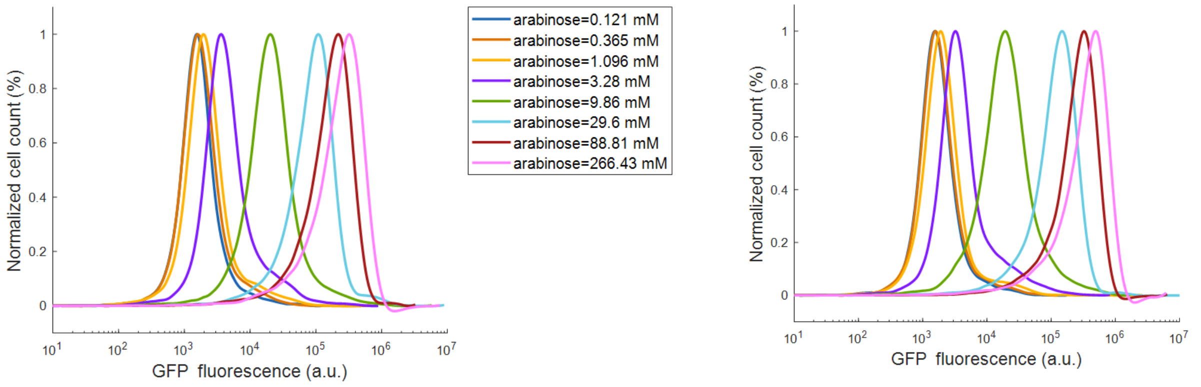

(E)

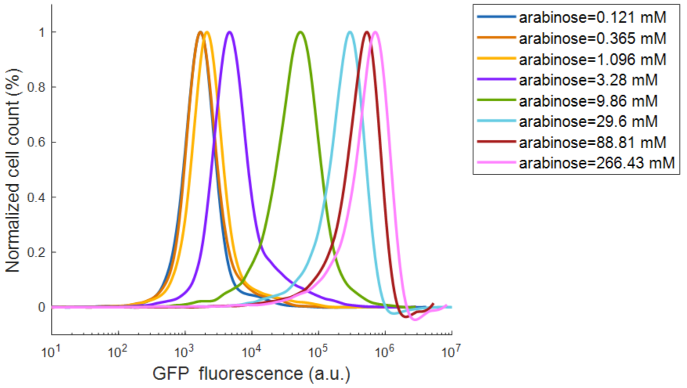
Normalized cell count (%)
GFP fluorescence (a.u.)
arabinose=0.121 mM
arabinose=0.365 mM
arabinose=1.096 mM
arabinose=3.28 mM
arabinose=9.86 mM
arabinose=29.6 mM
arabinose=88.81 mM
arabinose=266.43 mM

(F)

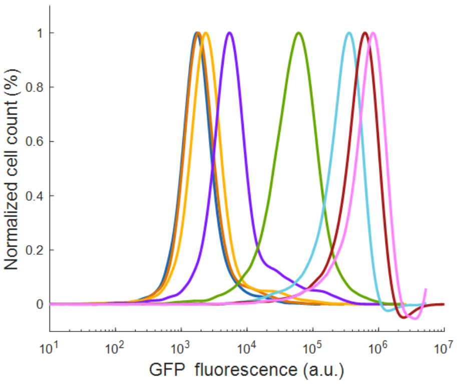
Normalized cell count (%)
GFP fluorescence (a.u.)

(G)

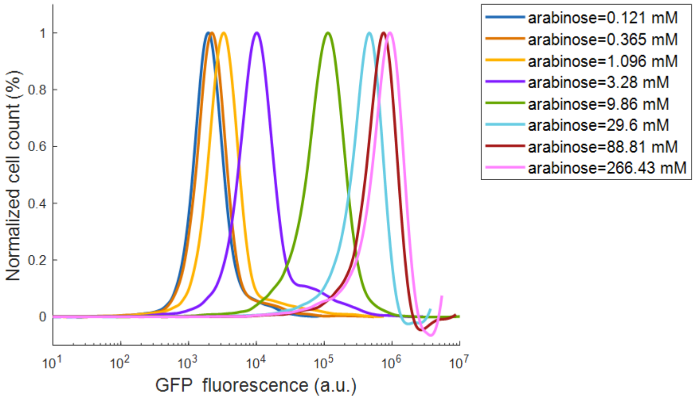
Normalized cell count (%)
GFP fluorescence (a.u.)
arabinose=0.121 mM
arabinose=0.365 mM
arabinose=1.096 mM
arabinose=3.28 mM
arabinose=9.86 mM
arabinose=29.6 mM
arabinose=88.81 mM
arabinose=266.43 mM

(H)

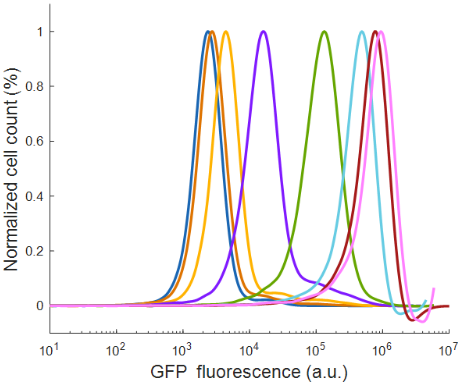
Normalized cell count (%)
GFP fluorescence (a.u.)

(I)

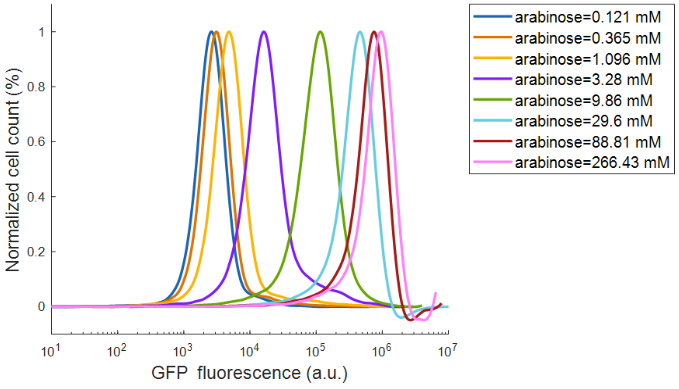
Normalized cell count (%)
GFP fluorescence (a.u.)
arabinose=0.121 mM
arabinose=0.365 mM
arabinose=1.096 mM
arabinose=3.28 mM
arabinose=9.86 mM
arabinose=29.6 mM
arabinose=88.81 mM
arabinose=266.43 mM

(J)

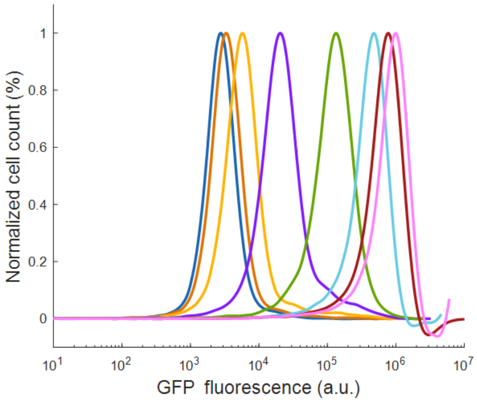
Normalized cell count (%)
GFP fluorescence (a.u.)

(K)

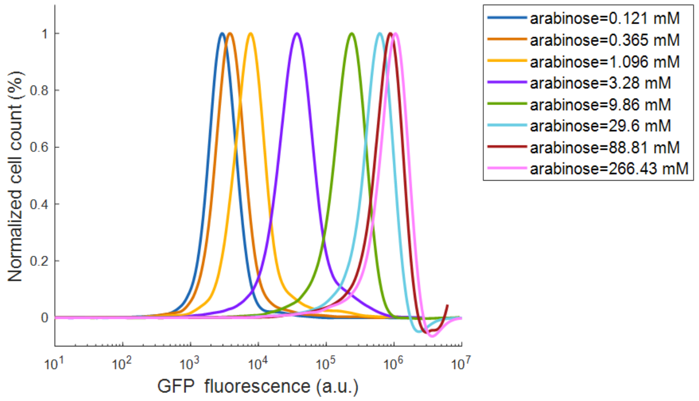

(L)

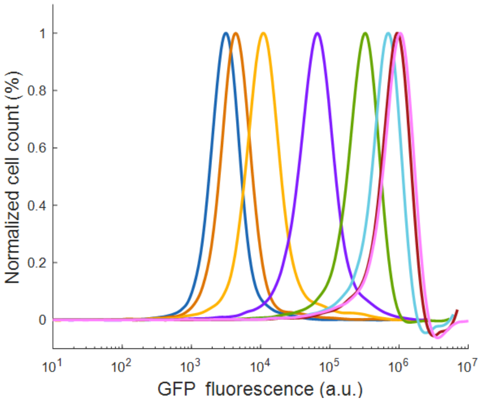

Fig. S28. Flow cytometry data for GFP over bacterial cells population containing ICF-TetR design of P_{BADsyn}. (A) [AHL]=0.5 μM, (B)[AHL]=0.25 μM, (C)[AHL]=0.125 μM, (D)[AHL]=0.625 μM, (E)[AHL]=0.0313 μM, (F)[AHL]=0.0156 μM, (G)[AHL]=0.0078 μM, (H)[AHL]=0.0039 μM, (I)[AHL]=0.002 μM, (J)[AHL]=0.001 μM, (K)[AHL]=0.0005 μM, (L)[AHL]=0.0002 μM. In all ICF-TetR experiments [aTc]=11.11ngr/ml. This experimental data was used for generating Fig.S17E.

(A)

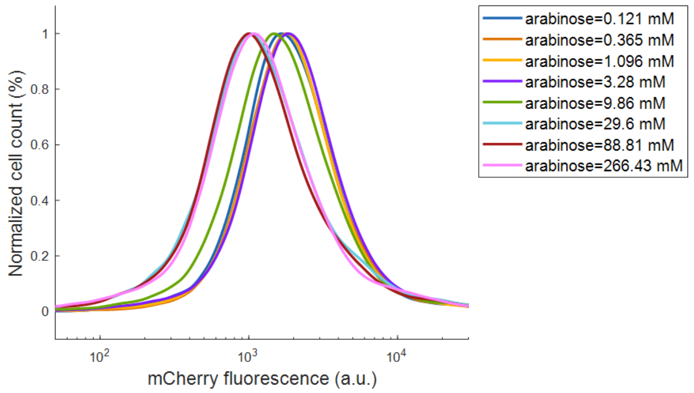

(B)

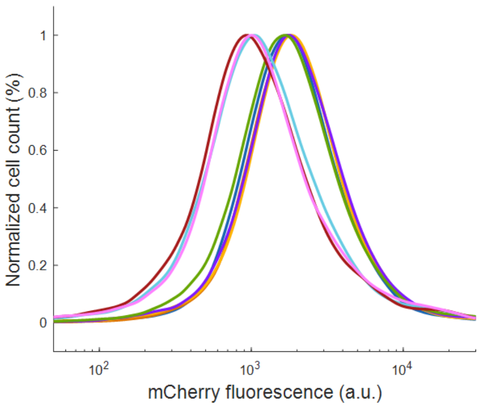

(C) (D)

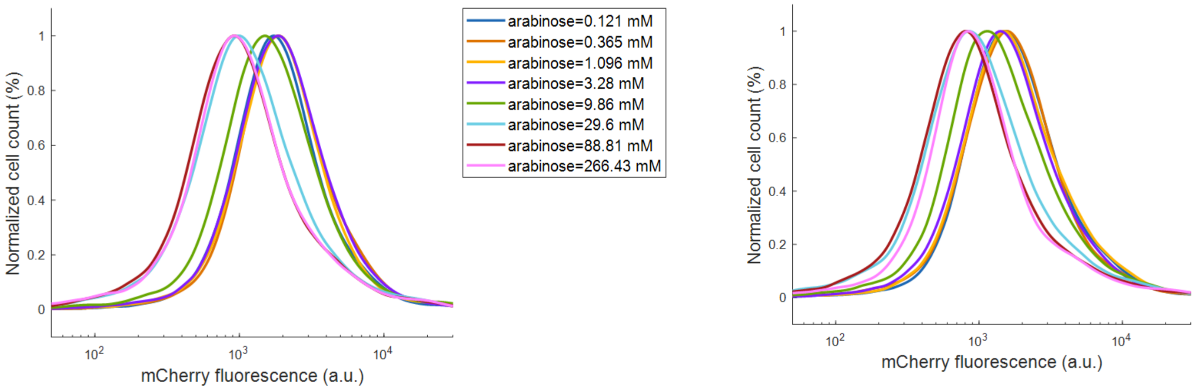

Fig. S29. Flow cytometry data for mCherry over bacterial cells population containing ICF-TetR design of P_{BADsyn}. (A) [AHL]=0.5 μM, (B) [AHL]=0.0625 μM, (C) [AHL]=0.0313 μM, (D) [AHL]=0.0002 μM. In all ICF-TetR experiments [aTc]=11.11ngr/ml. This experimental data was used for generating Fig.S17E.

(A) (B)

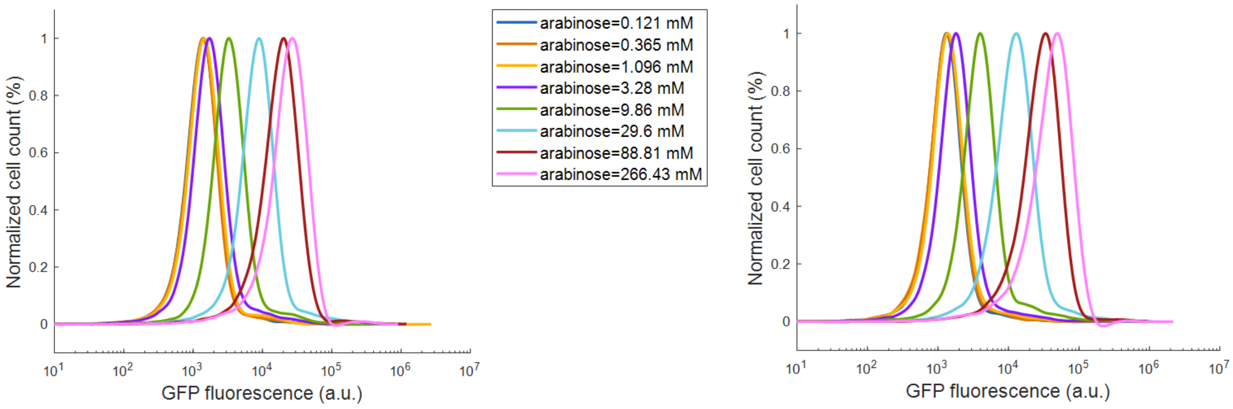

(C) (D)

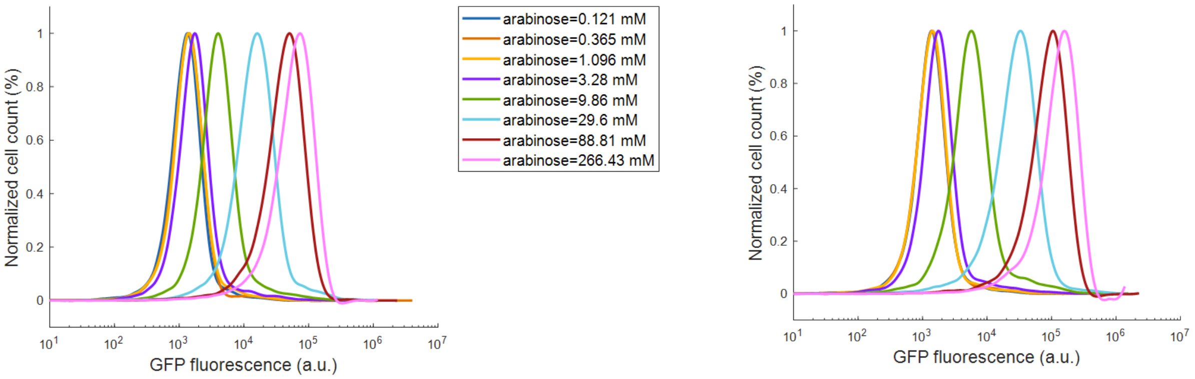

(E)

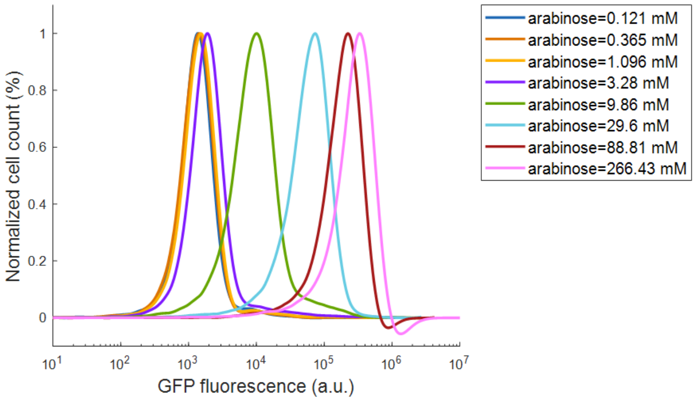
Normalized cell count (%)
GFP fluorescence (a.u.)
arabinose=0.121 mM
arabinose=0.365 mM
arabinose=1.096 mM
arabinose=3.28 mM
arabinose=9.86 mM
arabinose=29.6 mM
arabinose=88.81 mM
arabinose=266.43 mM

(F)

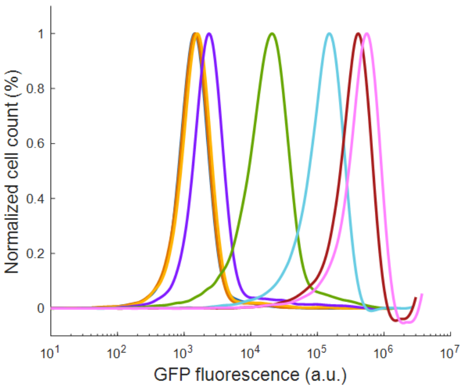
Normalized cell count (%)
GFP fluorescence (a.u.)

(G)

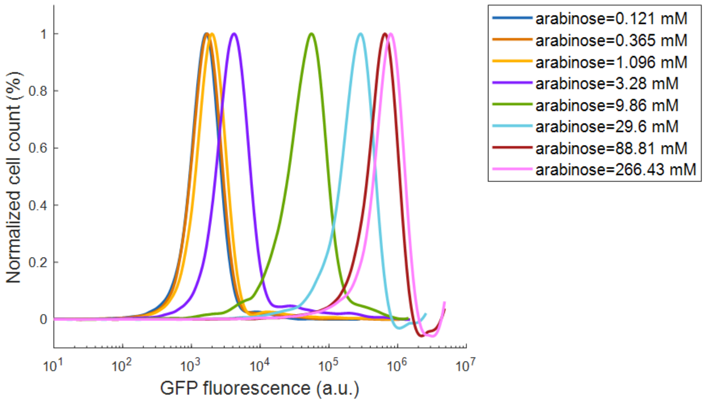
Normalized cell count (%)
GFP fluorescence (a.u.)
arabinose=0.121 mM
arabinose=0.365 mM
arabinose=1.096 mM
arabinose=3.28 mM
arabinose=9.86 mM
arabinose=29.6 mM
arabinose=88.81 mM
arabinose=266.43 mM

(H)

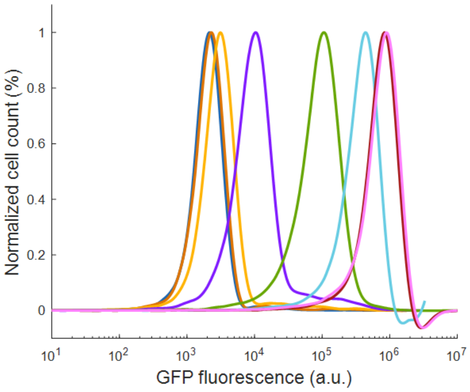
Normalized cell count (%)
GFP fluorescence (a.u.)

(I)

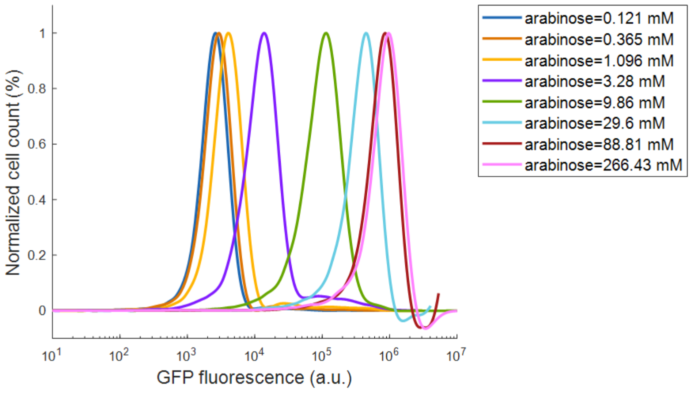
Normalized cell count (%)
GFP fluorescence (a.u.)
arabinose=0.121 mM
arabinose=0.365 mM
arabinose=1.096 mM
arabinose=3.28 mM
arabinose=9.86 mM
arabinose=29.6 mM
arabinose=88.81 mM
arabinose=266.43 mM

(J)

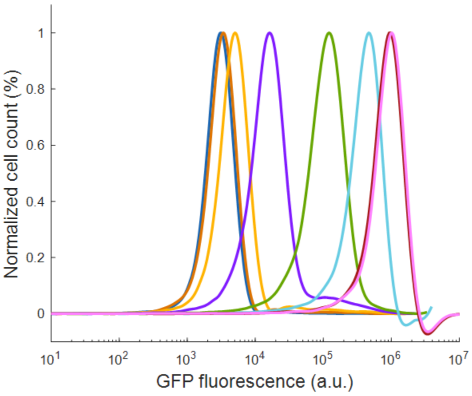
Normalized cell count (%)
GFP fluorescence (a.u.)

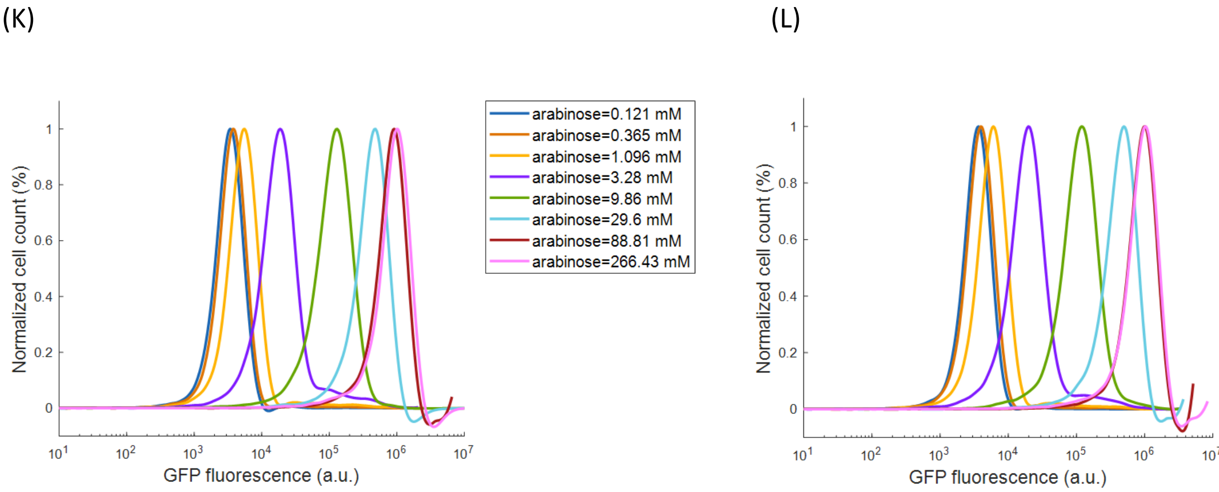

Fig. S30. Flow cytometry data for GFP over bacterial cells population containing DNF-TetR design of P_{BADsyn}. (A)[AHL]=0.5 µM, (B)[AHL]=0.25 µM, (C)[AHL]=0.125 µM, (D)[AHL]=0.625 µM, (E)[AHL]=0.0313 µM, (F)[AHL]=0.0156 µM, (G)[AHL]=0.0078 µM, (H)[AHL]=0.0039 µM, (I)[AHL]=0.002 µM, (J)[AHL]=0.001 µM, (K)[AHL]=0.0005 µM, (L)[AHL]=0.0002 µM. In all ICF-TetR experiments [aTc]=11.11ngr/ml. This experimental data was used for generating Fig.S17F.

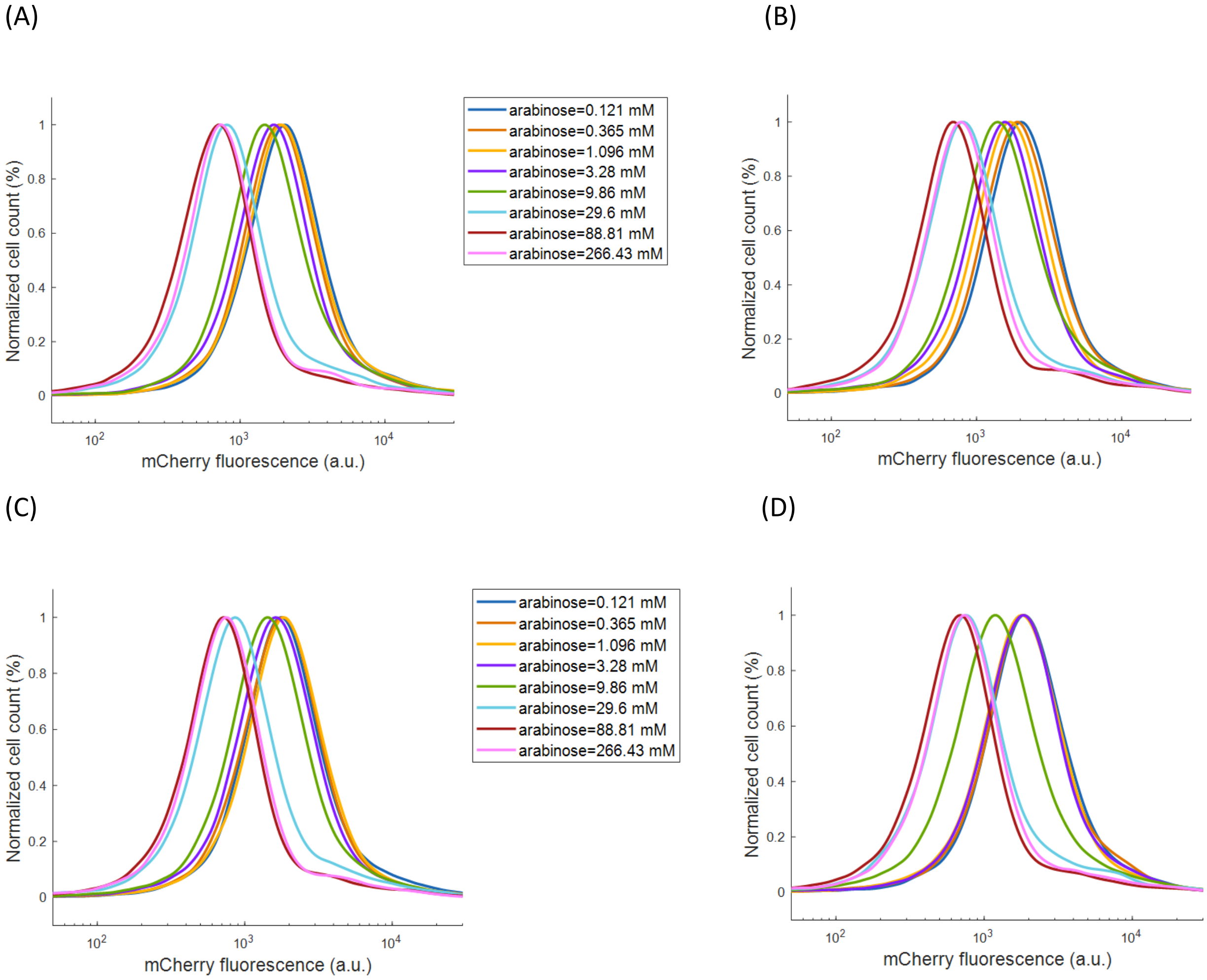

Fig. S31. Flow cytometry data for mCherry over bacterial cells population containing DNF-TetR design of P_{BADsyn}. (A) [AHL]=0.5 μM, (B) [AHL]=0.0625 μM, (C) [AHL]=0.0313 μM, (D) [AHL]=0.0078 μM. In all DNF-TetR experiments [aTc]=11.11ngr/ml. This experimental data was used for generating Fig.S17F.

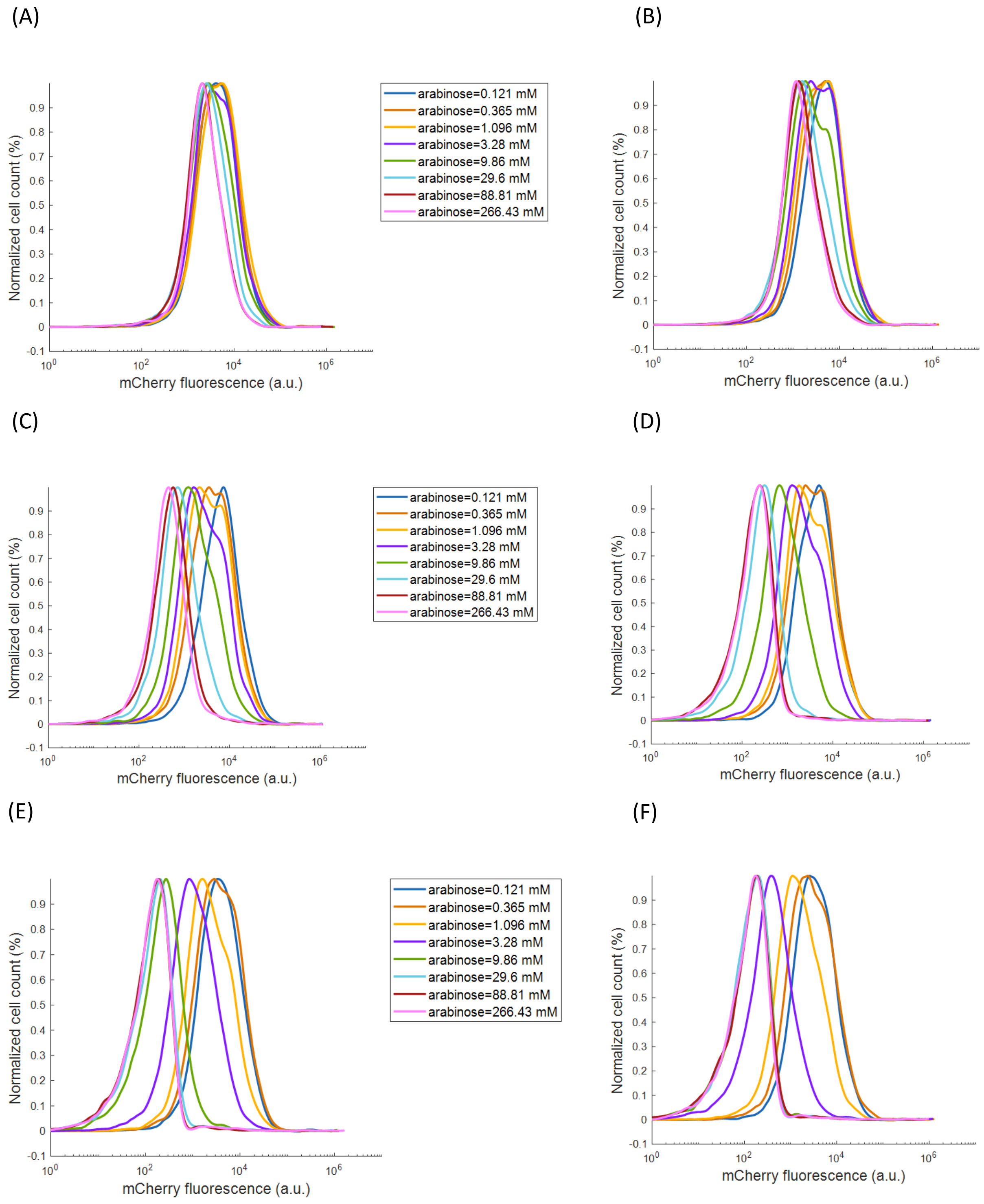

(G)

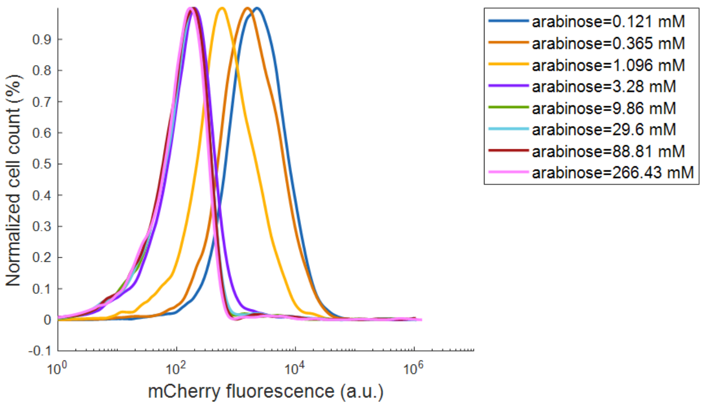
Normalized cell count (%)
mCherry fluorescence (a.u.)
arabinose=0.121 mM
arabinose=0.365 mM
arabinose=1.096 mM
arabinose=3.28 mM
arabinose=9.86 mM
arabinose=29.6 mM
arabinose=88.81 mM
arabinose=266.43 mM

(H)

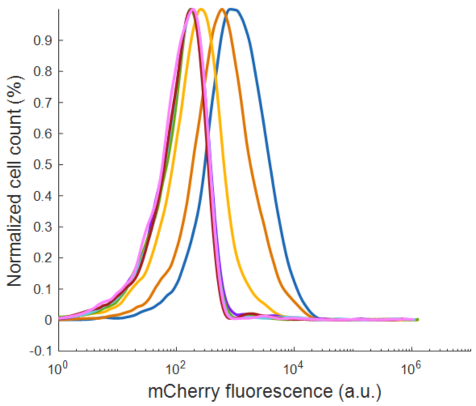
Normalized cell count (%)
mCherry fluorescence (a.u.)

(I)

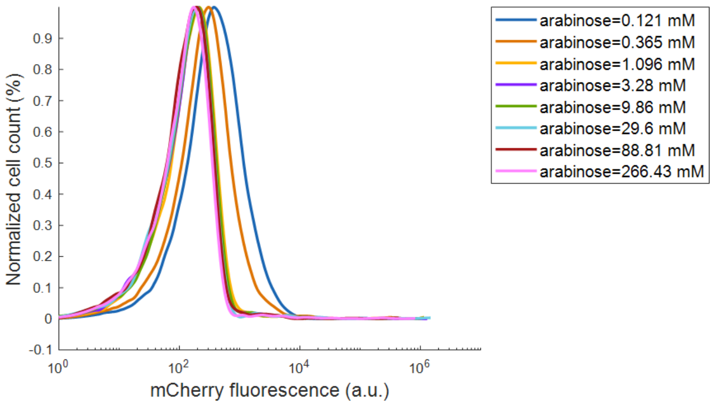
Normalized cell count (%)
mCherry fluorescence (a.u.)
arabinose=0.121 mM
arabinose=0.365 mM
arabinose=1.096 mM
arabinose=3.28 mM
arabinose=9.86 mM
arabinose=29.6 mM
arabinose=88.81 mM
arabinose=266.43 mM

(J)

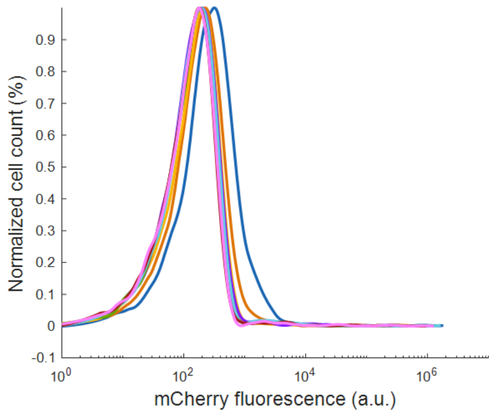
Normalized cell count (%)
mCherry fluorescence (a.u.)

(K)

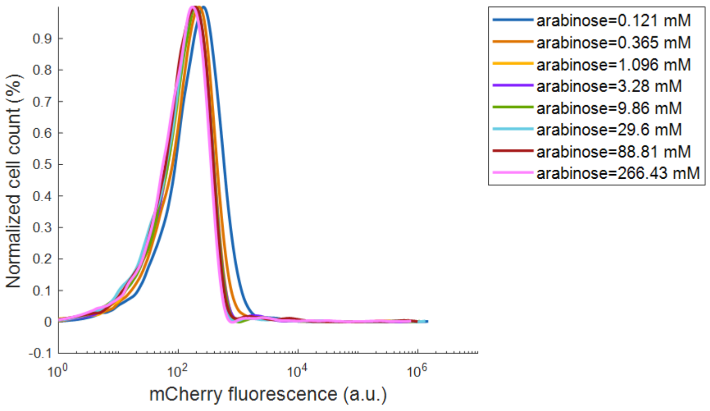
Normalized cell count (%)
mCherry fluorescence (a.u.)
arabinose=0.121 mM
arabinose=0.365 mM
arabinose=1.096 mM
arabinose=3.28 mM
arabinose=9.86 mM
arabinose=29.6 mM
arabinose=88.81 mM
arabinose=266.43 mM

(L)

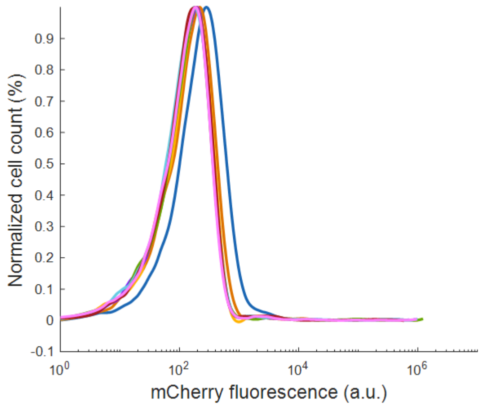
Normalized cell count (%)
mCherry fluorescence (a.u.)

(O)

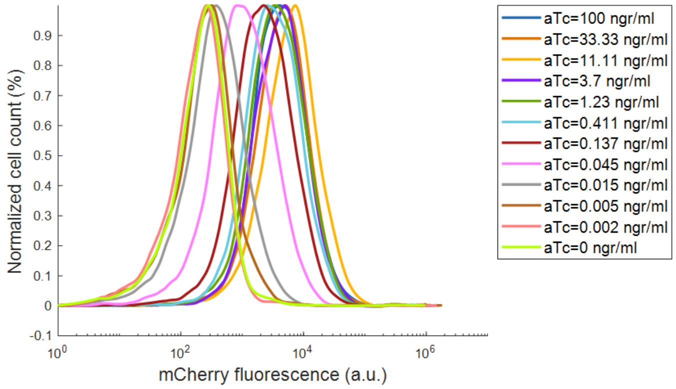

(P)

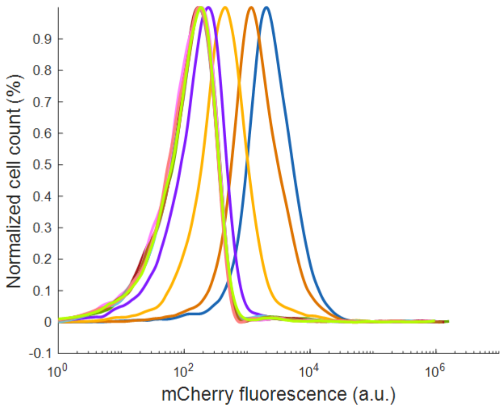

Fig. S32. Flow cytometry data for mCherry over bacterial cells population containing arabinose-aTc inverting switch (TetR) design of P_{BADsyn}. (A)[aTc]=100ngr/ml, (B)[aTc]=33.33ngr/ml, (C)[aTc]=11.11ngr/ml, (D) [aTc]=3.7ngr/ml, (E) [aTc]=1.23ngr/ml, (F) [aTc]=0.411ngr/ml, (G) [aTc]=0.137ngr/ml, (H) [aTc]=0.045ngr/ml, (I) [aTc]=0.015ngr/ml, (J) [aTc]=0.005ngr/ml, (K) [aTc]=0.002ngr/ml, (L) [aTc]=0ngr/ml. (O)[arabinose]=0.121mM, (P) [arabinose]=266.43mM. This experimental data was used for generating Fig.S17C.

(A)

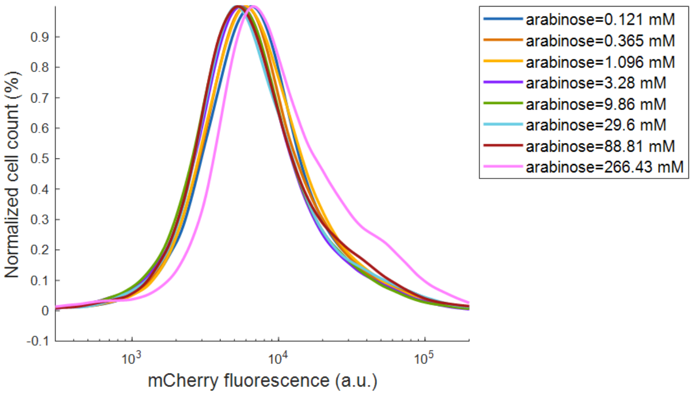

(B)

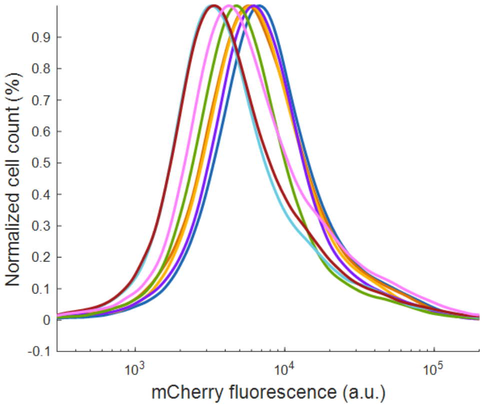

(C)

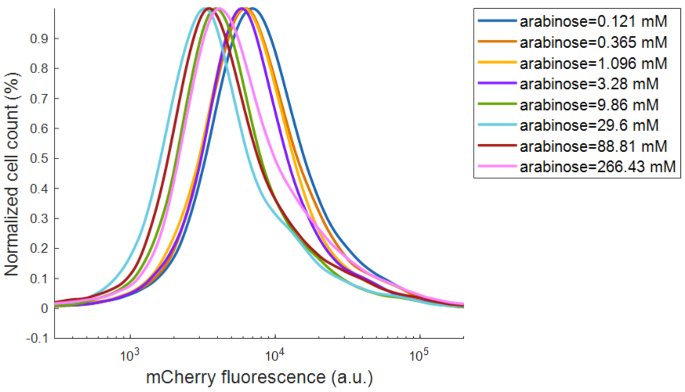

(D)

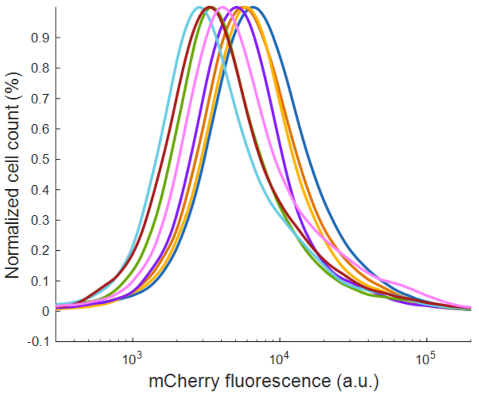

(E)

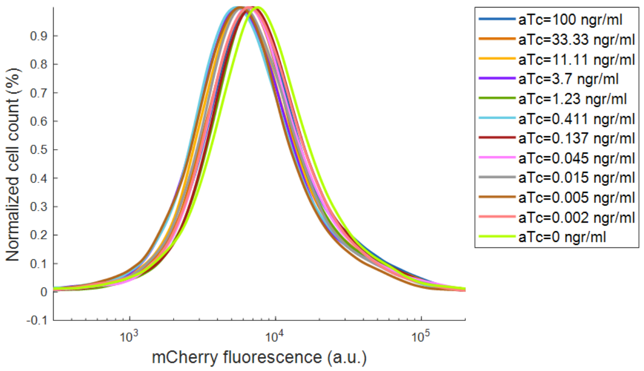

(F)

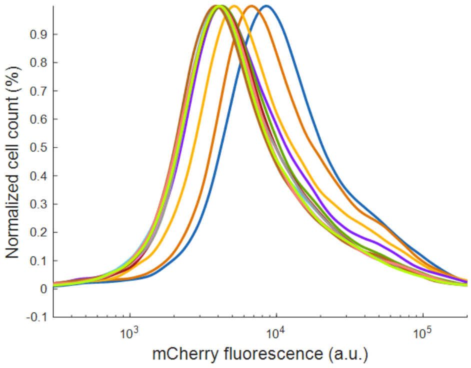

Fig. S33. Flow cytometry data for mCherry over bacterial cells population containing arabinose-aTc inverting switch (TetR-LVA) design of P_{BADsyn}. (A)[aTc]=33.33ngr/ml, (B)[aTc]=1.23ngr/ml, (C)[aTc]=0.137ngr/ml, (D) [aTc]=0.02ngr/ml, (E) [arabinose]=0.121mM, (F) [arabinose]=266.43mM. This experimental data was used for generating Fig.S17B.

4.2 Experimental data for synthetic promoter P_{lacO}

(A)

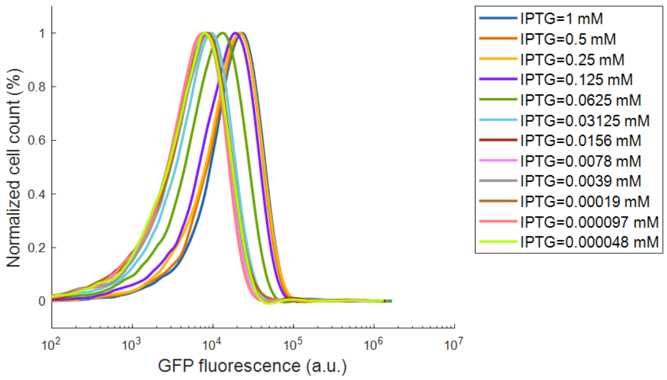

(B)

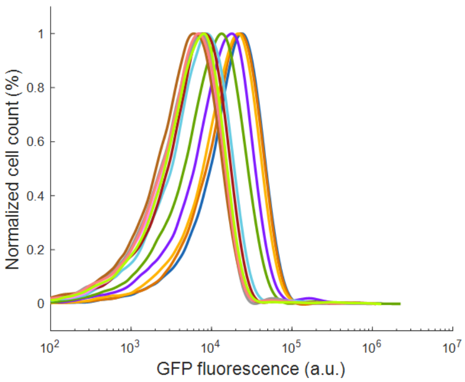

(C)

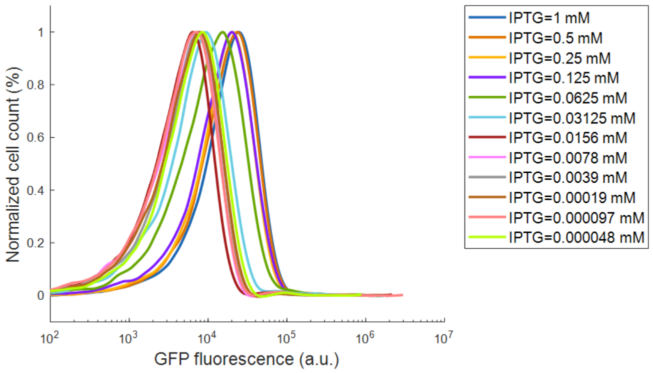

(D)

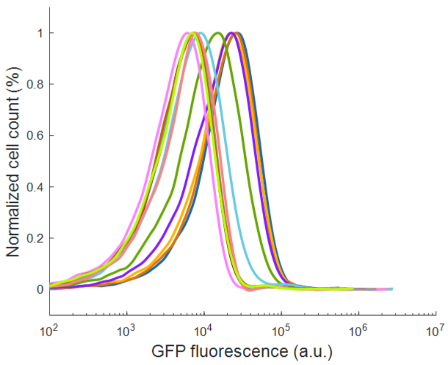

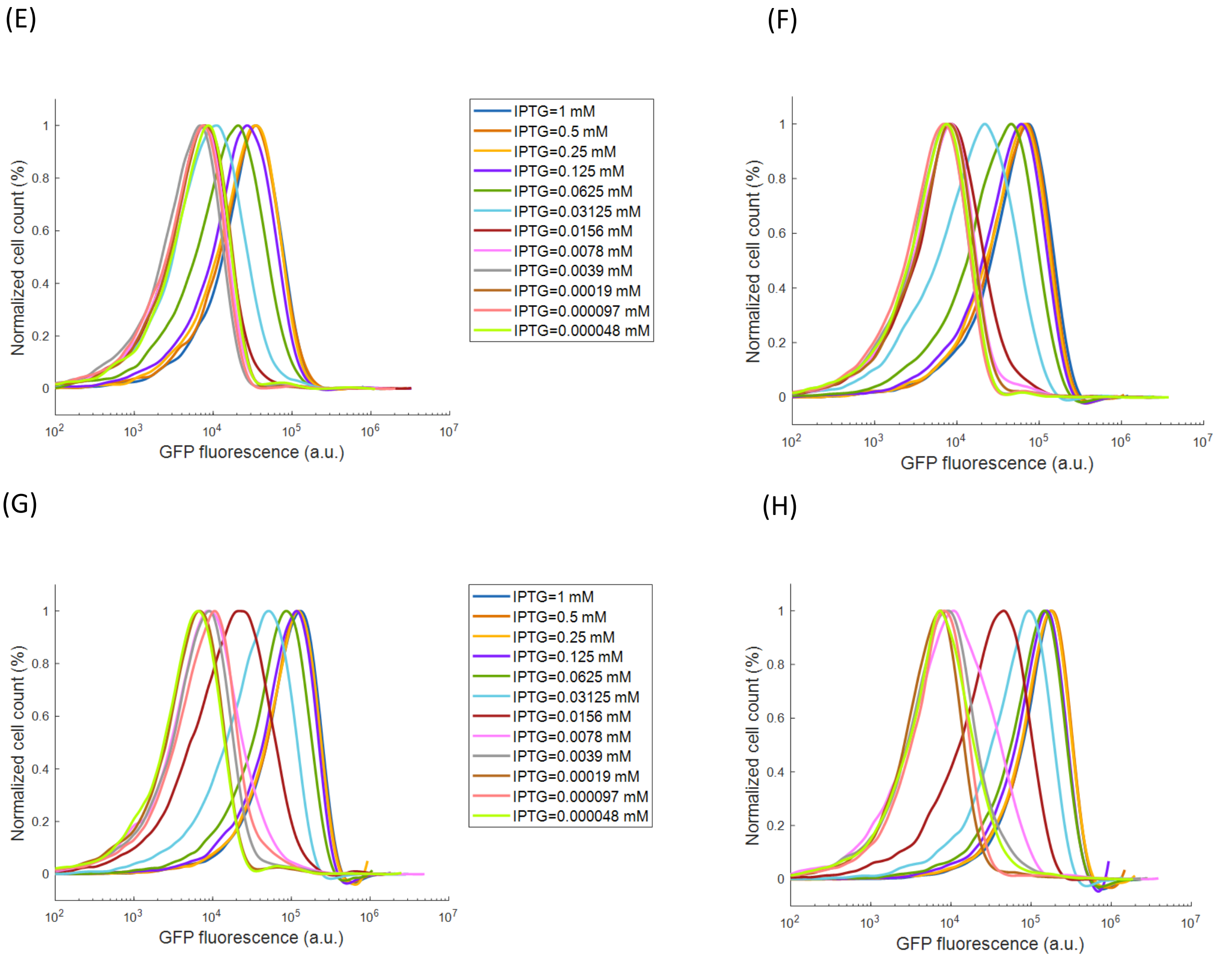

Fig. S34. Flow cytometry data for GFP over bacterial cells population containing OL design of P_{lacO}. (A) [AHL]=6.66 μM, (B) [AHL]=2.22 μM, (C) [AHL]=0.74 μM, (D) [AHL]=0.24 μM, (E) [AHL]=0.08 μM, (F) [AHL]=0.03 μM (G) [AHL]=0.009 μM, (H) [AHL]=0.00 μM. This experimental data was used for generating Fig.S19A.

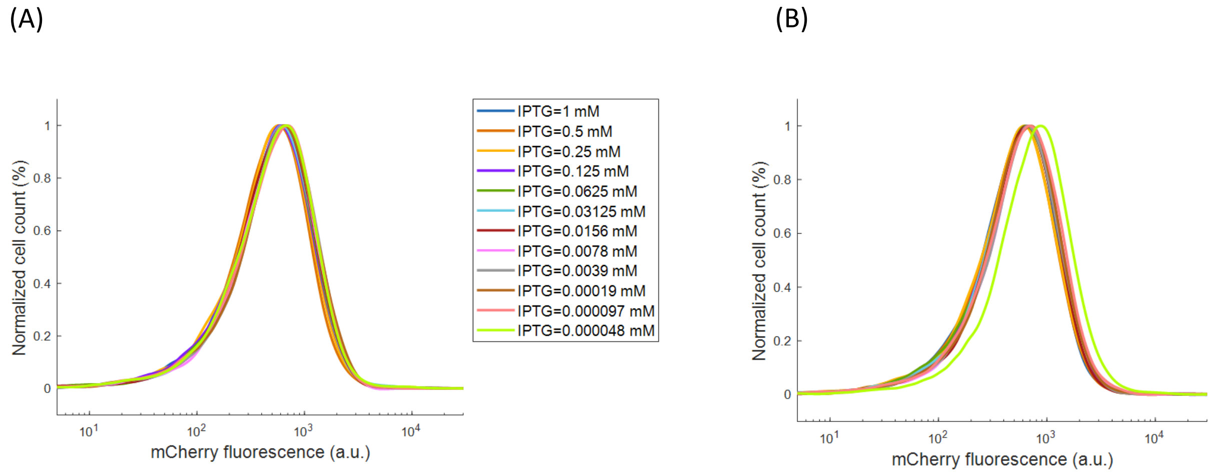

(C)

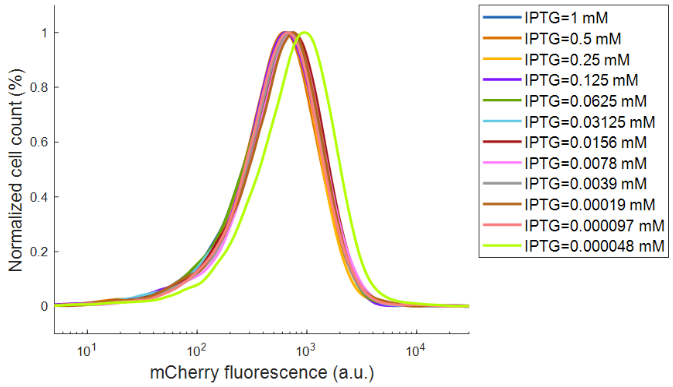

(D)

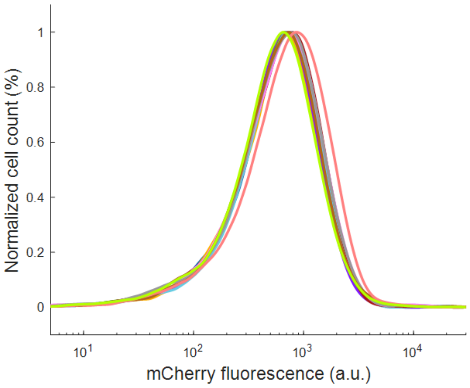

Fig. S35. Flow cytometry data for mCherry over bacterial cells population containing OL design of P_{lacO}. (A) [AHL]=6.66 μM M, (B) [AHL]=0.74 μM, (C) [AHL]=0.08 μM, (D) [AHL]=0.00 μM. This experimental data was used for generating Fig.S19A.

(A)

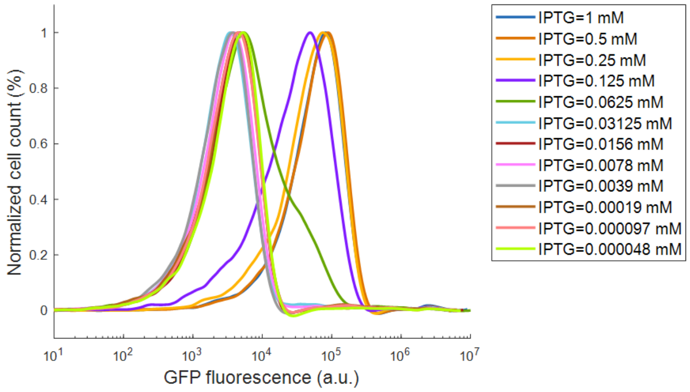

(B)

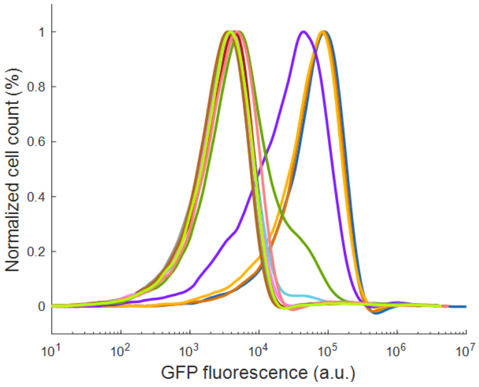

(C)

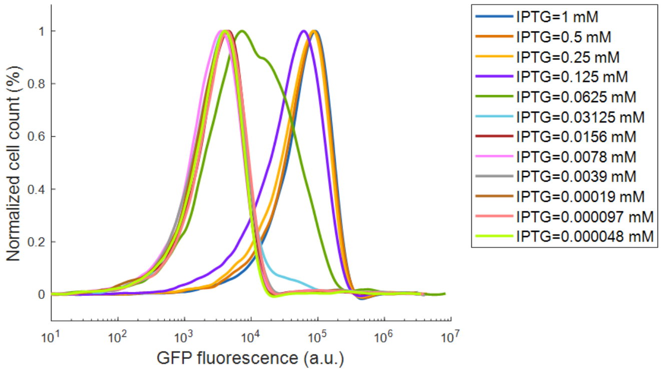

(D)

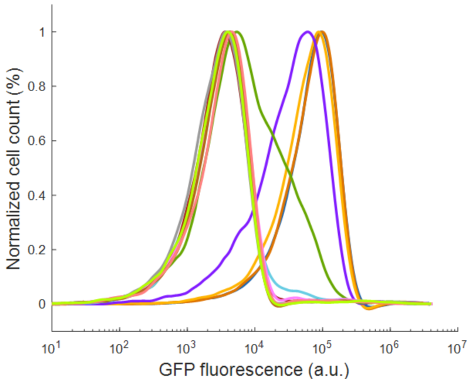

(E)

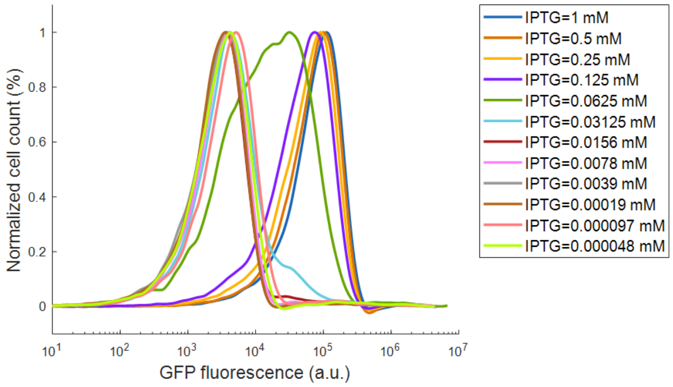

(F)

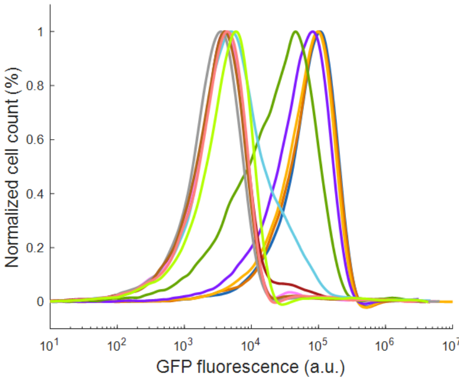

(G)

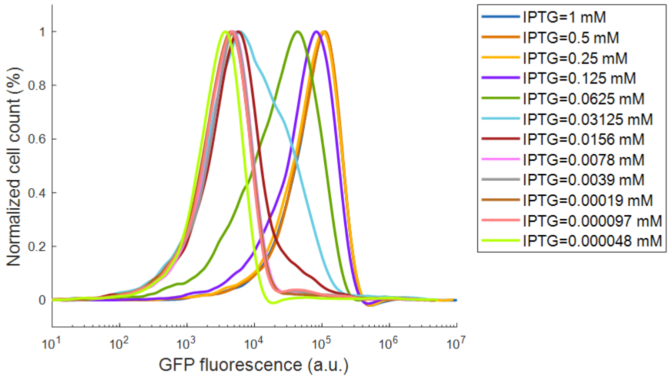

(H)

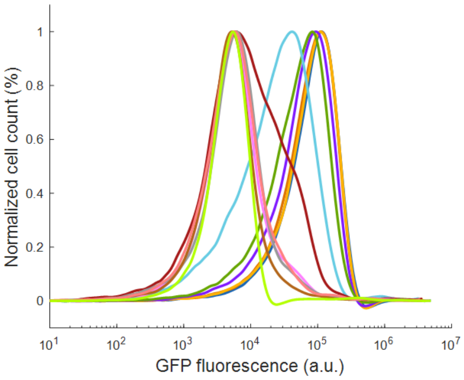

Fig. S36. Flow cytometry data for GFP over bacterial cells population containing ICF-TetR-LVA design of P_{lacO}. (A) [AHL]=6.66 μM, (B) [AHL]=2.22 μM, (C) [AHL]=0.74 μM, (D) [AHL]=0.24 μM, (E) [AHL]=0.08 μM, (F) [AHL]=0.03 μM (G) [AHL]=0.009 μM, (H) [AHL]=0.00 μM. This experimental data was used for generating Fig.S19C.

(A)

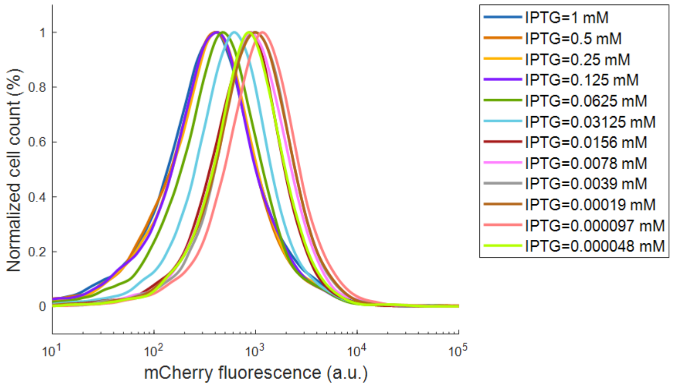

(B)

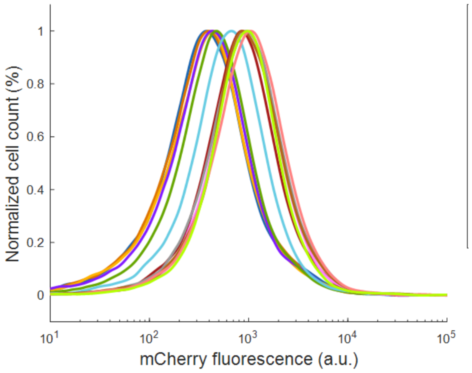

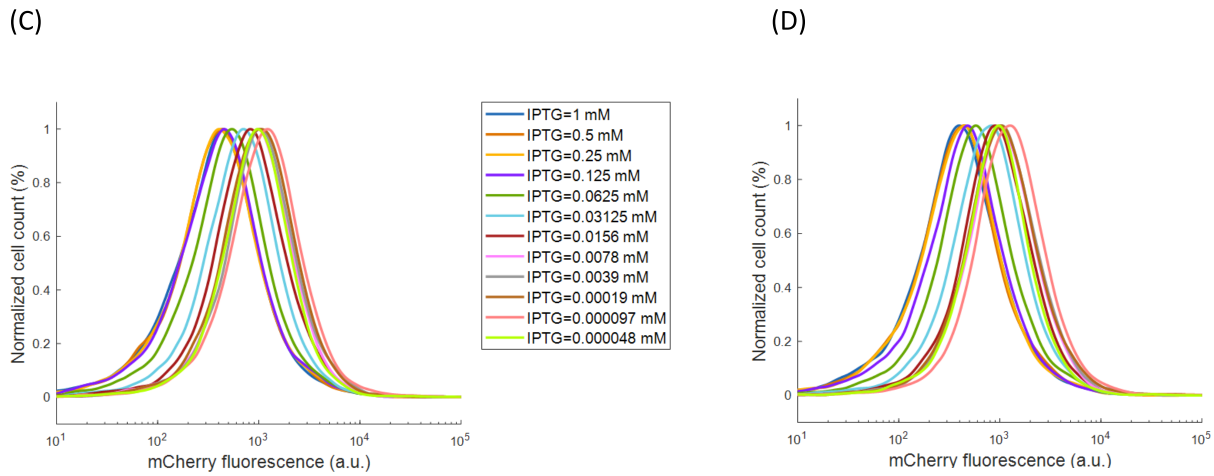

Fig. S37. Flow cytometry data for mCherry over bacterial cells population containing ICF-TetR-LVA design of P_{lacO}. (A) [AHL]=6.66 μM, (B) [AHL]=0.74 μM, (C) [AHL]=0.08 μM, (D) [AHL]=0.009 μM. This experimental data was used for generating Fig.S19C.

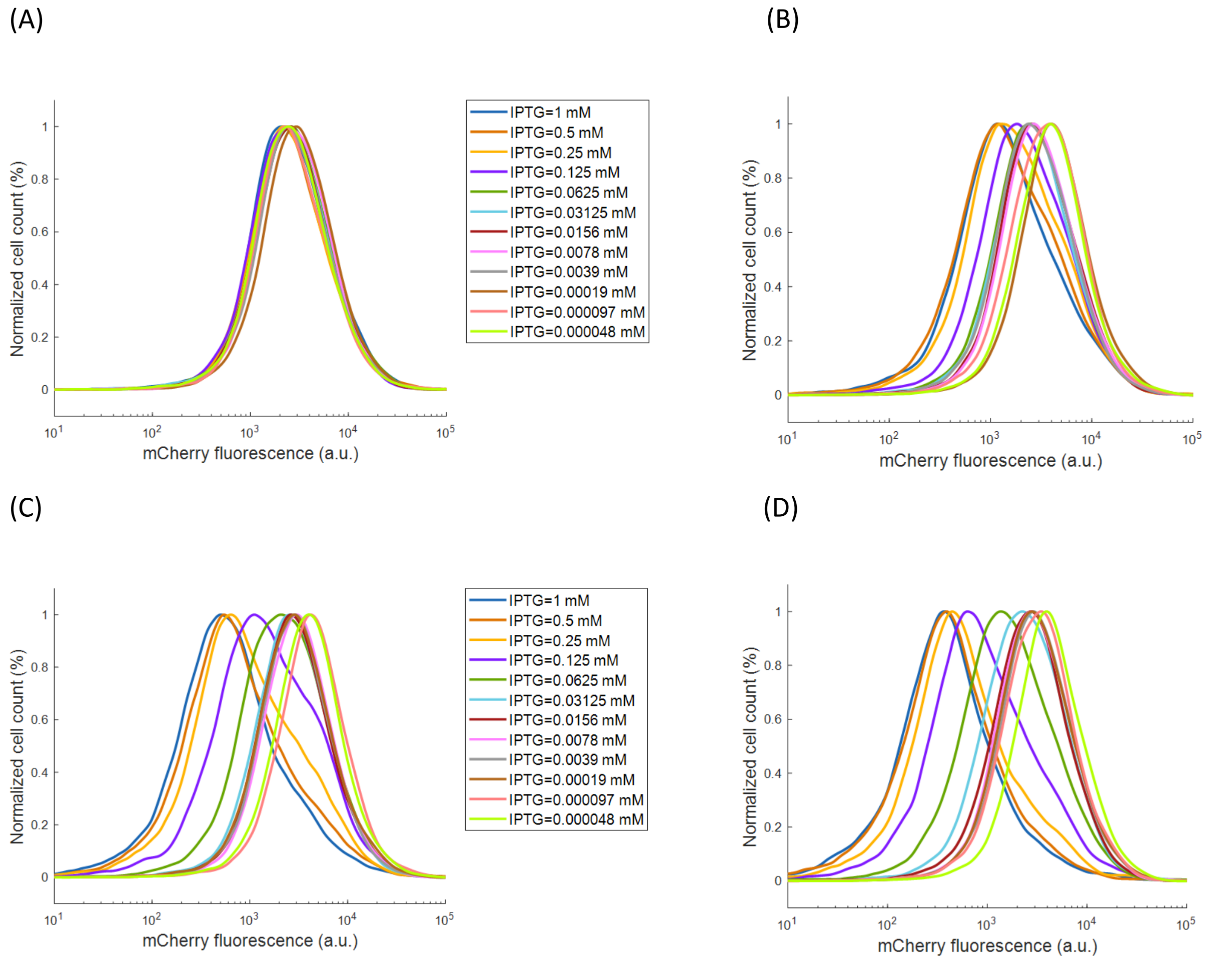

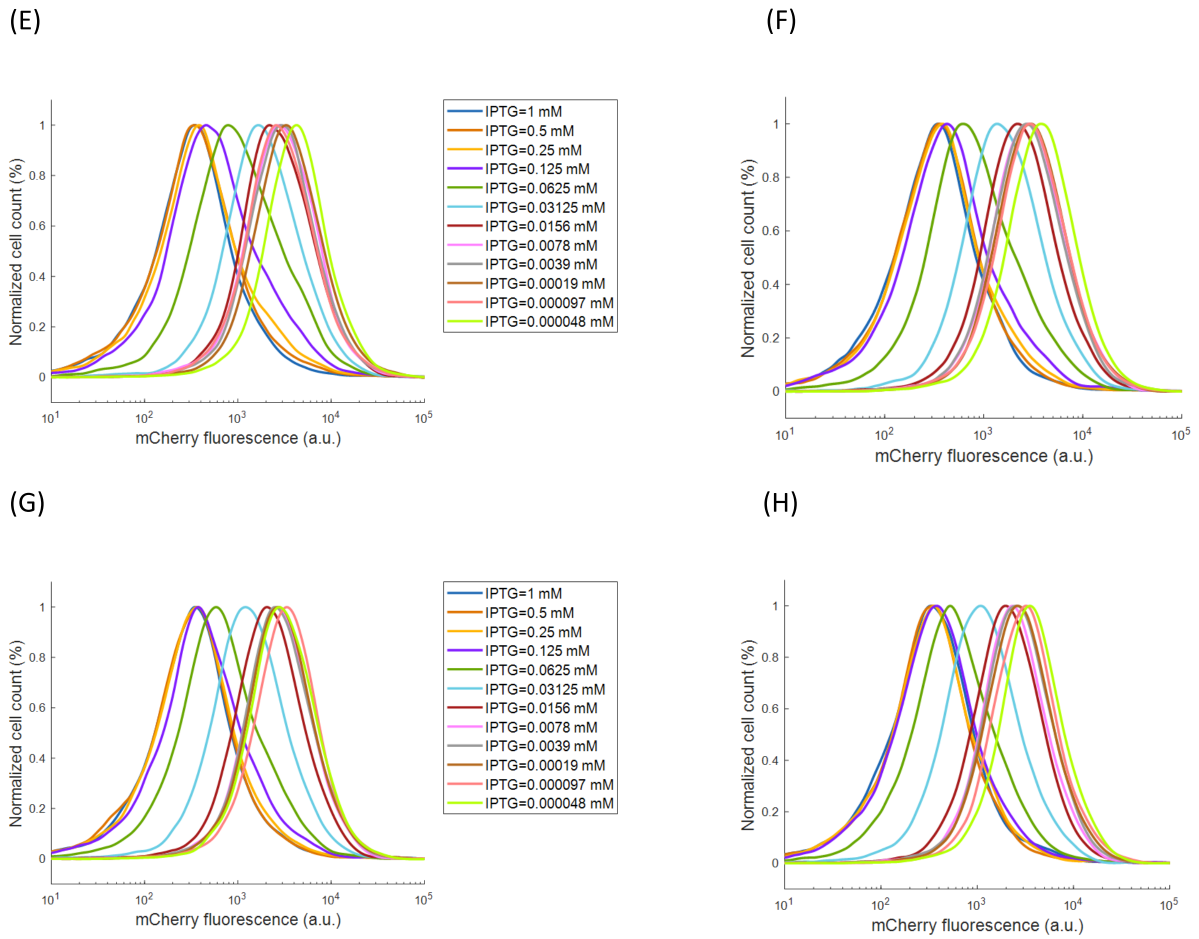

Fig. S38. Flow cytometry data for mCherry over bacterial cells population containing IPTG-aTc inverting switch design of P_{lacO}. (A)[aTc]=100ngr/ml, (B)[aTc]=33.33ngr/ml, (C)[aTc]=11.11ngr/ml, (D) [aTc]=3.7ngr/ml, (E) [aTc]=1.23ngr/ml, (F) [aTc]=0.411ngr/ml, (G) [aTc]=0.137ngr/ml, (H) [aTc]=0ngr/ml. This experimental data was used for generating Fig.S19B.

4.3 Experimental data for native specific promoter P_{LhrtO} .

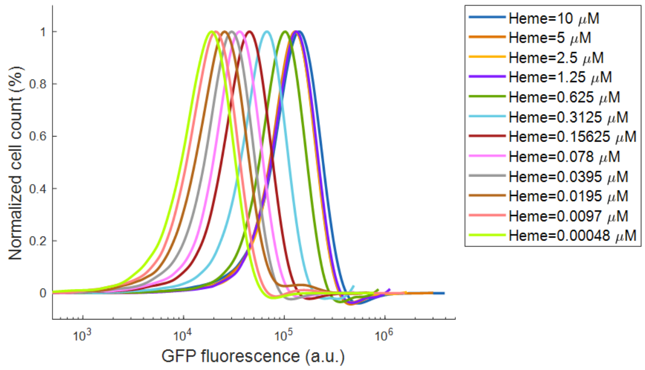

Fig. S39. Flow cytometry data for GFP over bacterial cells population containing wild-type design of P_{LhrtO}. This experimental data was used for generating Fig.7A and Fig.S20A.

(A)

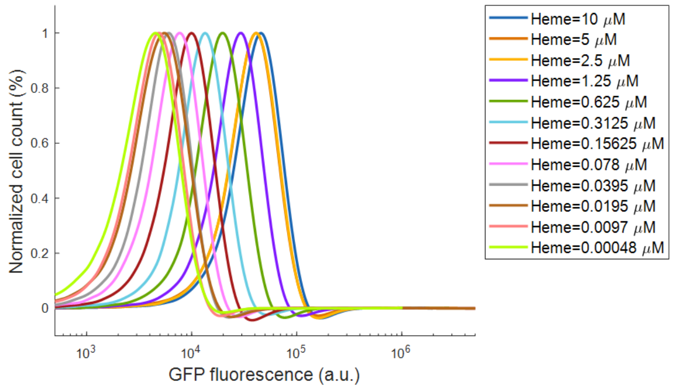
Normalized cell count (%)
GFP fluorescence (a.u.)
Heme=10 μM
Heme=5 μM
Heme=2.5 μM
Heme=1.25 μM
Heme=0.625 μM
Heme=0.3125 μM
Heme=0.15625 μM
Heme=0.078 μM
Heme=0.0395 μM
Heme=0.0195 μM
Heme=0.0097 μM
Heme=0.00048 μM

(B)

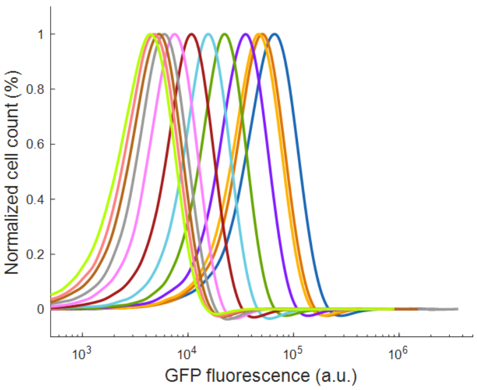
Normalized cell count (%)
GFP fluorescence (a.u.)

(C)

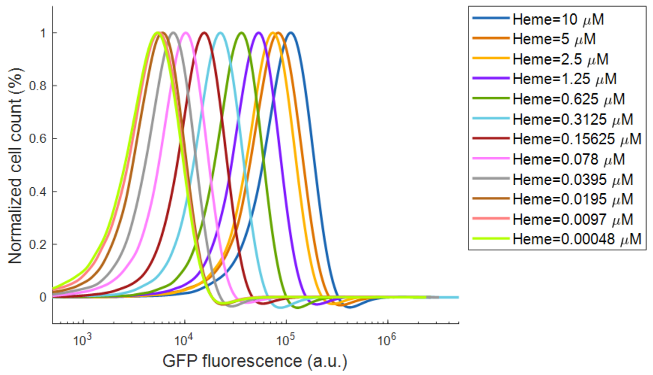
Normalized cell count (%)
GFP fluorescence (a.u.)
Heme=10 μM
Heme=5 μM
Heme=2.5 μM
Heme=1.25 μM
Heme=0.625 μM
Heme=0.3125 μM
Heme=0.15625 μM
Heme=0.078 μM
Heme=0.0395 μM
Heme=0.0195 μM
Heme=0.0097 μM
Heme=0.00048 μM

(D)

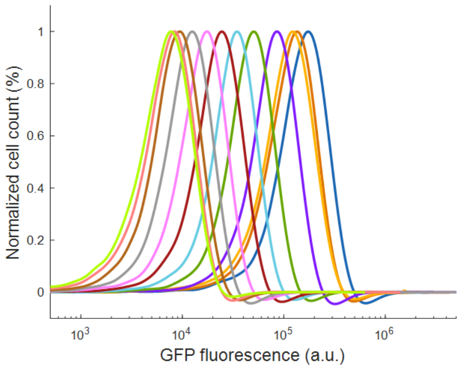
Normalized cell count (%)
GFP fluorescence (a.u.)

(E)

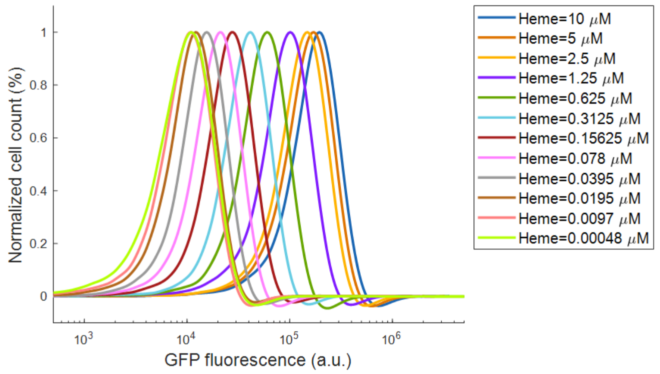
Normalized cell count (%)
GFP fluorescence (a.u.)
Heme=10 μM
Heme=5 μM
Heme=2.5 μM
Heme=1.25 μM
Heme=0.625 μM
Heme=0.3125 μM
Heme=0.15625 μM
Heme=0.078 μM
Heme=0.0395 μM
Heme=0.0195 μM
Heme=0.0097 μM
Heme=0.00048 μM

(F)

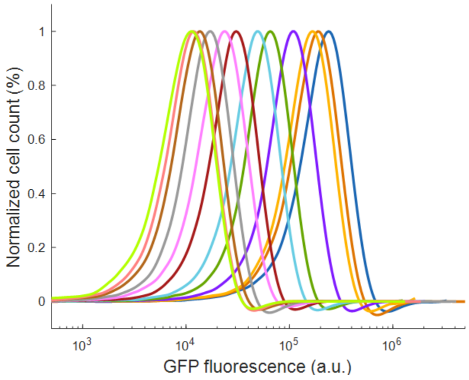
Normalized cell count (%)
GFP fluorescence (a.u.)

(G)

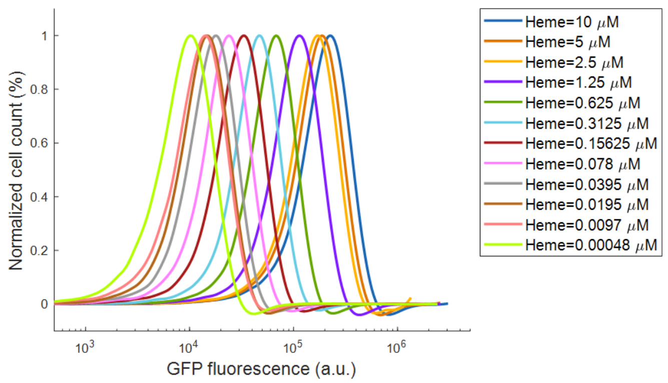

(H)

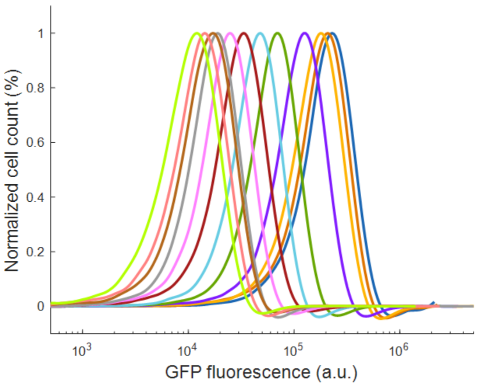

(I)

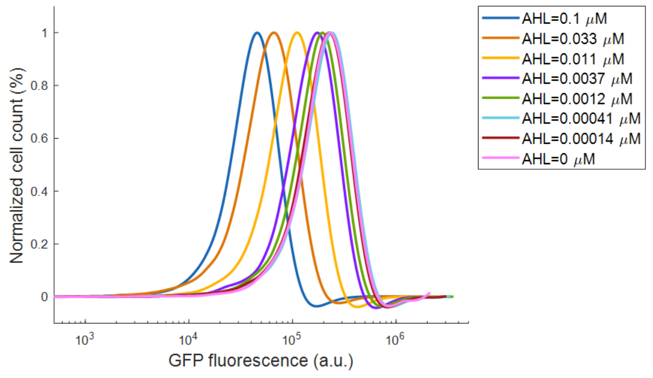

(J)

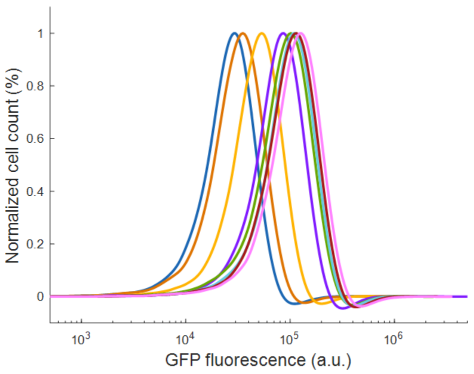

(K)

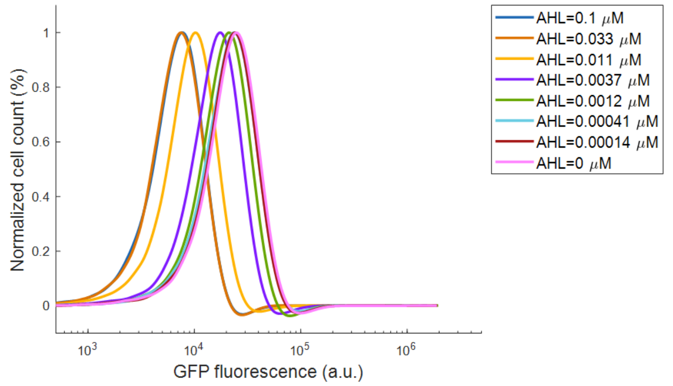

(L)

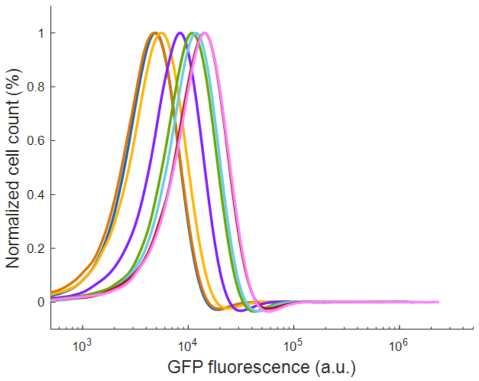

Fig. S40. Flow cytometry data for GFP over bacterial cells population containing OL design of P_{LhrtO}. (A) [AHL]=0.1 μM, (B) [AHL]=0.033 μM, (C) [AHL]=0.011 μM, (D) [AHL]=0.0037 μM, (E) [AHL]=0.0012 μM, (F) [AHL]=0.0004 μM, (G) [AHL]=0.0001 μM, (H) [AHL]=0 μM, (I) [Heme]=10 μM, (J) [Heme]=1.25 μM, (K) [Heme]=0.078 μM, (L) [Heme]=0.00977 μM. This experimental data was used for generating Fig.7B and Fig.S20B.

(A)

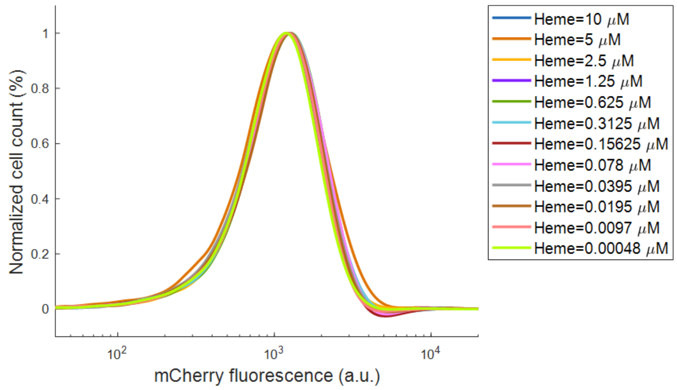

(B)

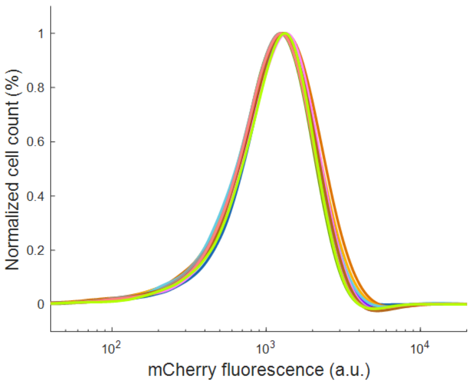

(C)

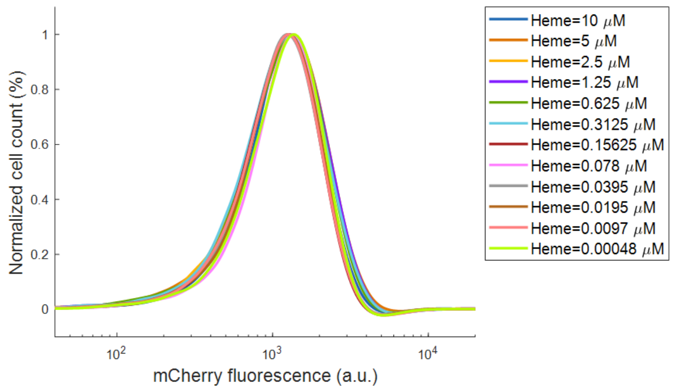

(D)

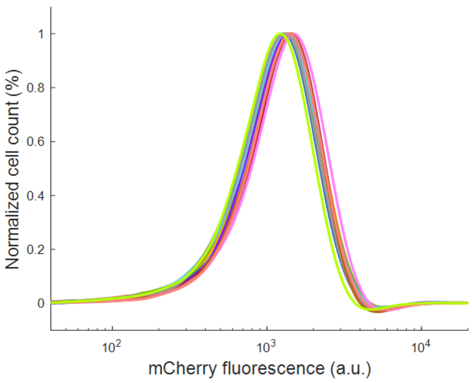

Fig. S41. Flow cytometry data for mCherry over bacterial cells population containing OL design of P_{LhrtO}. (A) [AHL]=0.1 μM, (B) [AHL]=0.011 μM, (C) [AHL]=0.0012 μM, (D) [AHL]=0.00014 μM. This experimental data was used for generating Fig.S20B.

(A)

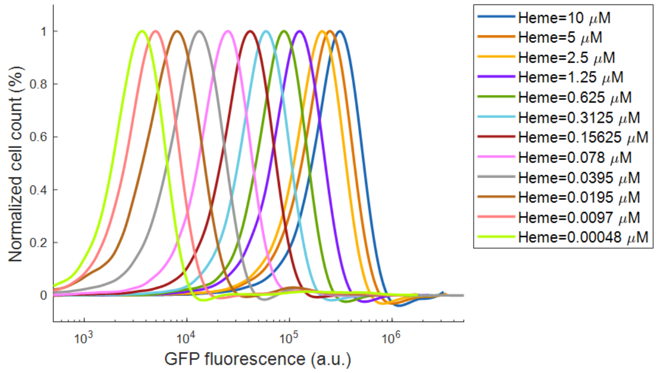

(B)

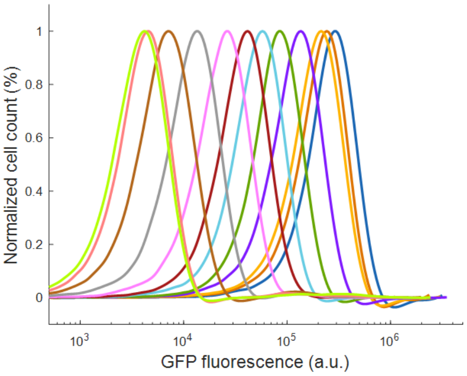

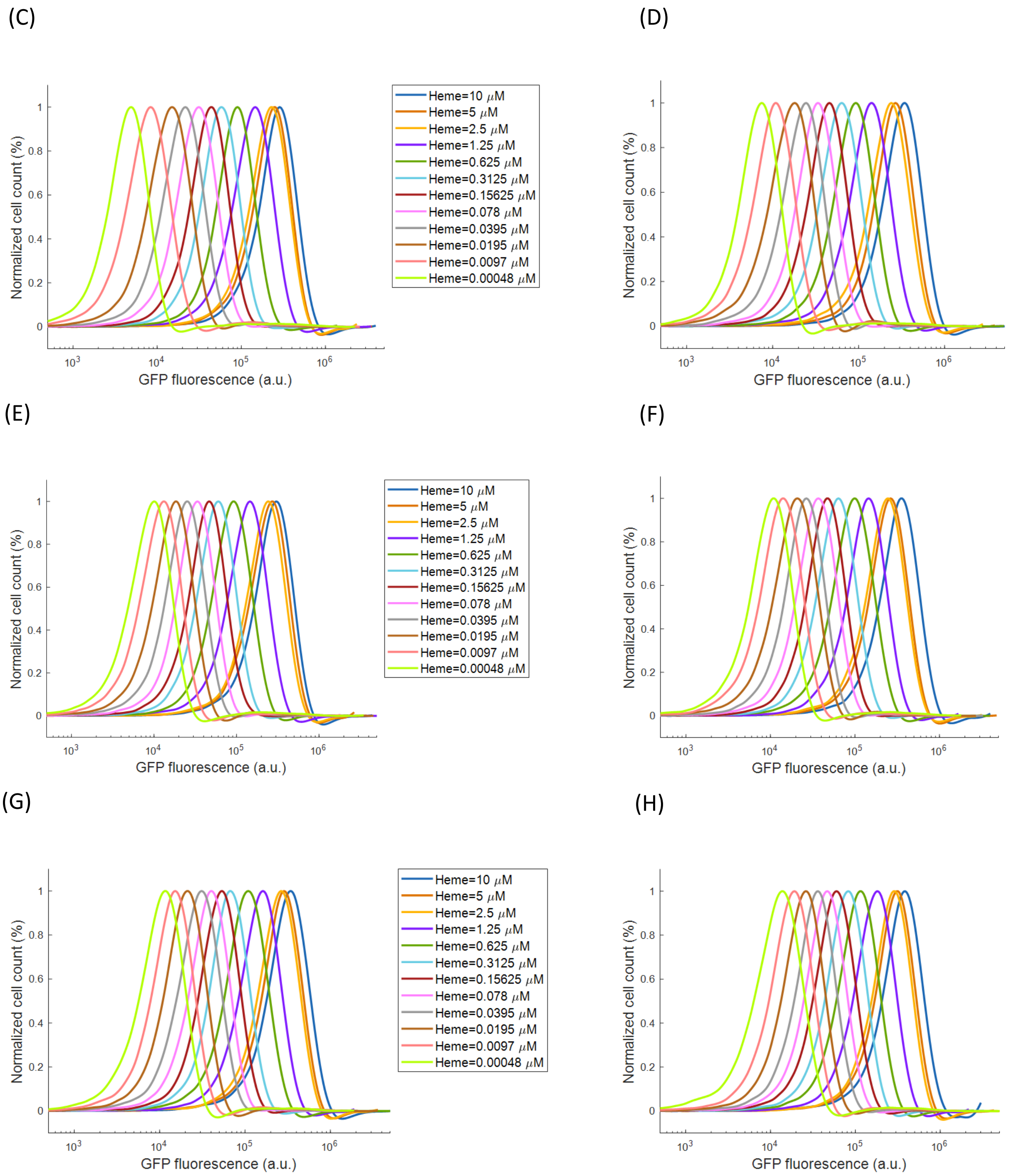

Fig. S42. Flow cytometry data for GFP over bacterial cells population containing ICF-TetR-AAV design of P_{LhrtO}. (A) [AHL]=0.1 μM, (B) [AHL]=0.033 μM, (C) [AHL]=0.011 μM, (D) [AHL]=0.0037 μM, (E) [AHL]=0.0012 μM, (F) [AHL]=0.0004 μM, (G) [AHL]=0.0001 μM, (H) [AHL]=0 μM. ([aTc]=11.11ngr/ml). This experimental data was used for generating Fig.7C and Fig.S20D.

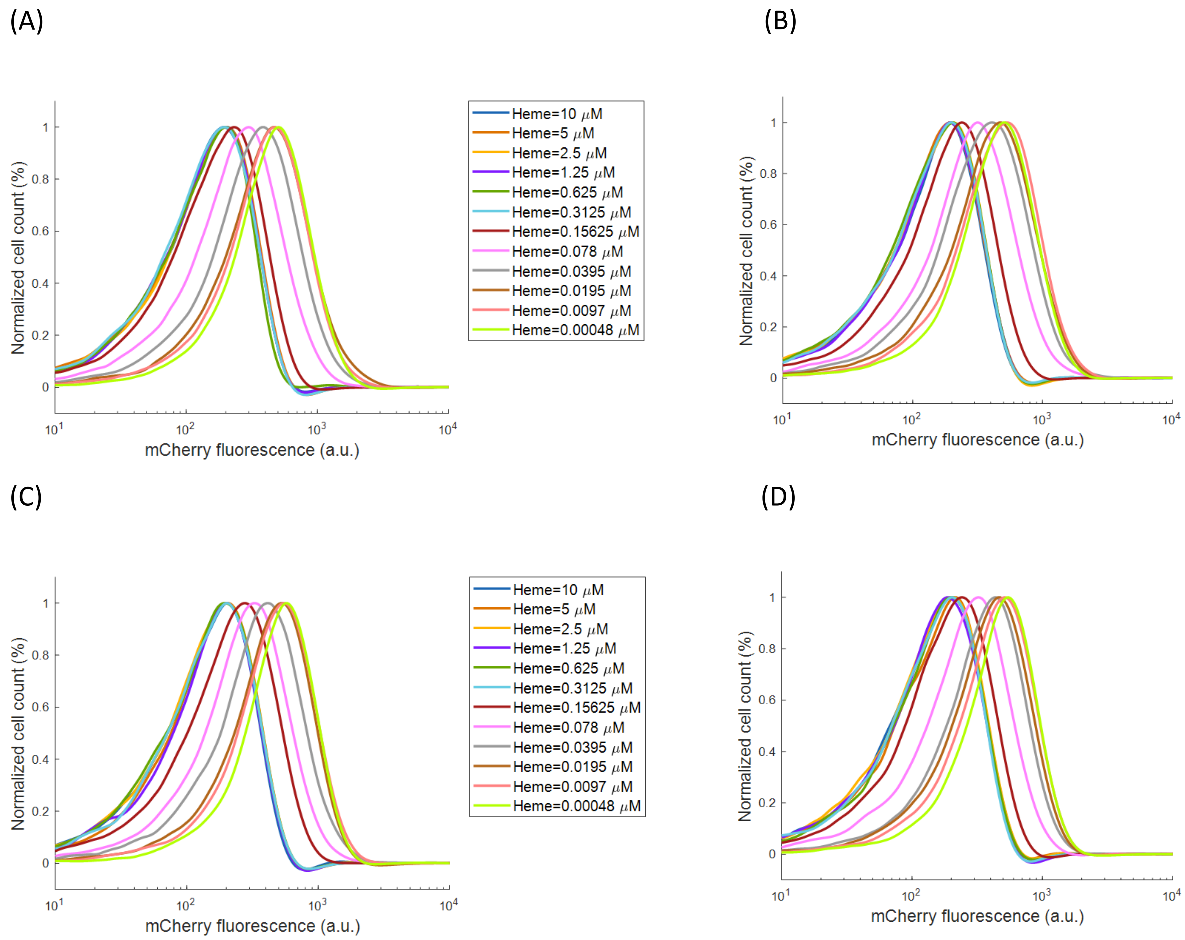

Fig. S43. Flow cytometry data for mCherry over bacterial cells population containing ICF-TetR-AAV design of P_{LhrtO}. (A) [AHL]=0.1 μM, (B) [AHL]= 0.011 μM, (C) [AHL]= 0.0012 μM, (D) [AHL]= 0.00014 μM. ([aTc]=11.11ngr/ml) This experimental data was used for generating Fig.S20D.

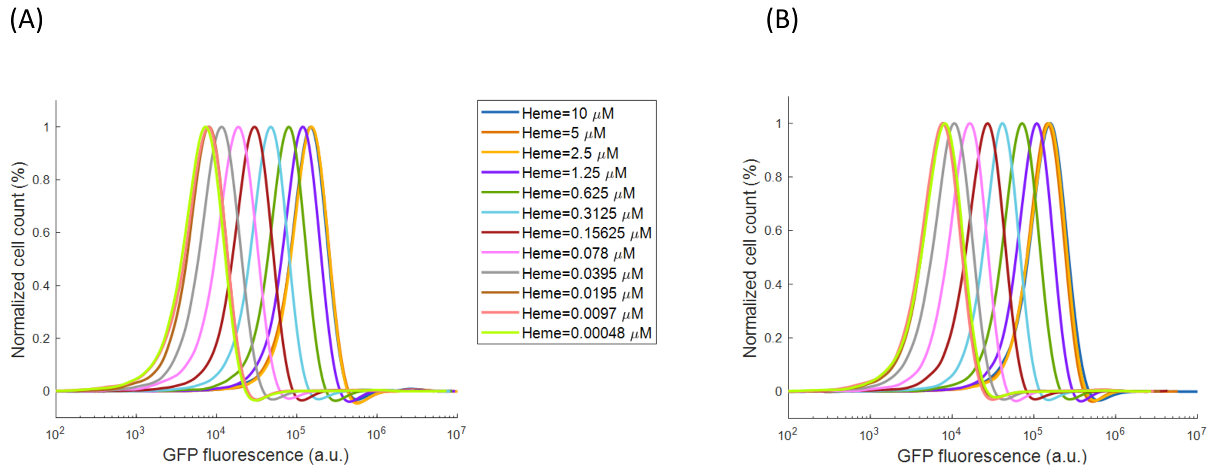

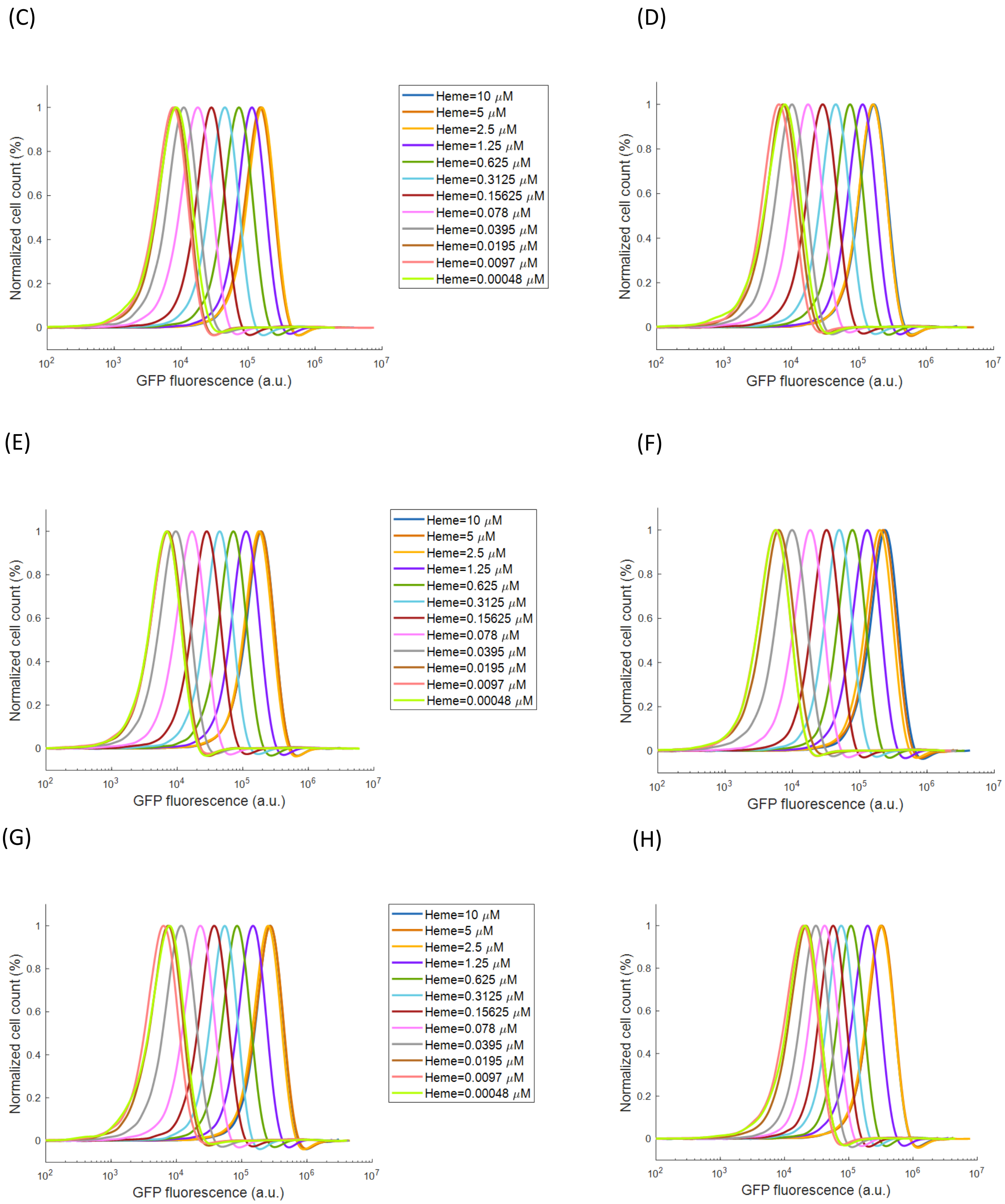

Fig. S44. Flow cytometry data for GFP over bacterial cells population containing DNF-TetR-AAV design of P_{LhrtO}. (A) [AHL]=0.1 μM, (B) [AHL]=0.033 μM, (C) [AHL]=0.011 μM, (D) [AHL]=0.0037 μM, (E) [AHL]=0.0012 μM, (F) [AHL]=0.0004 μM, (G) [AHL]=0.0001 μM, (H) [AHL]=0 μM. ([aTc]=3.7ngr/ml) This experimental data was used for generating Fig.S20E.

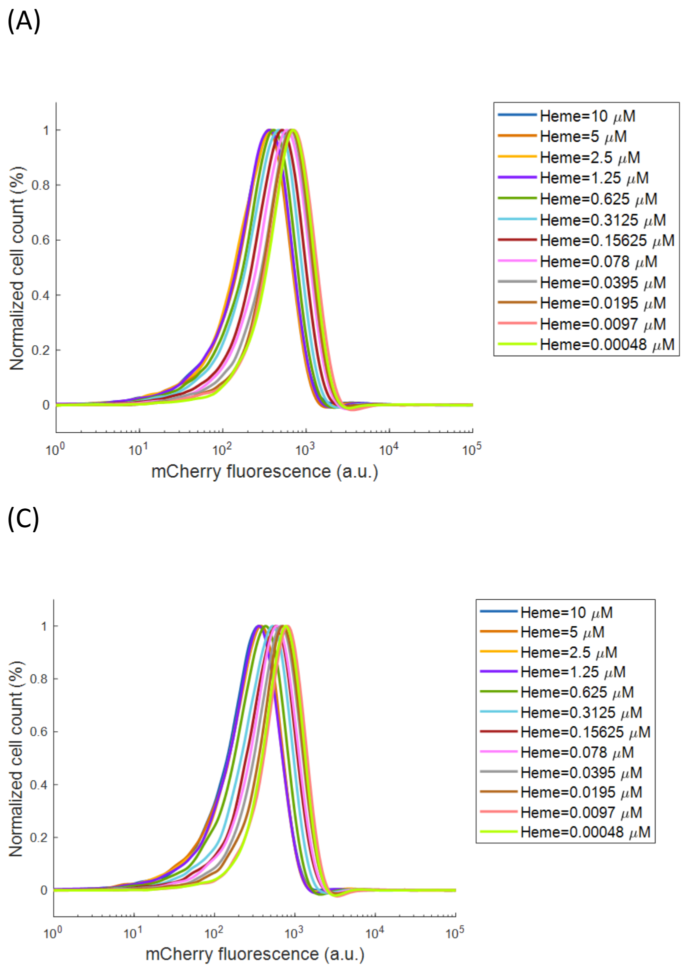

Fig. S45. Flow cytometry data for mCherry over bacterial cells population containing DNF-TetR-AAV design of P_{LhrtO}. (A) [AHL]=0.1 μM, (B) [AHL]= 0.011 μM, (C) [AHL]= 0.0012 μM, (D) [AHL]= 0.00014 μM. ([aTc]=3.7ngr/ml) This experimental data was used for generating Fig.S20E.

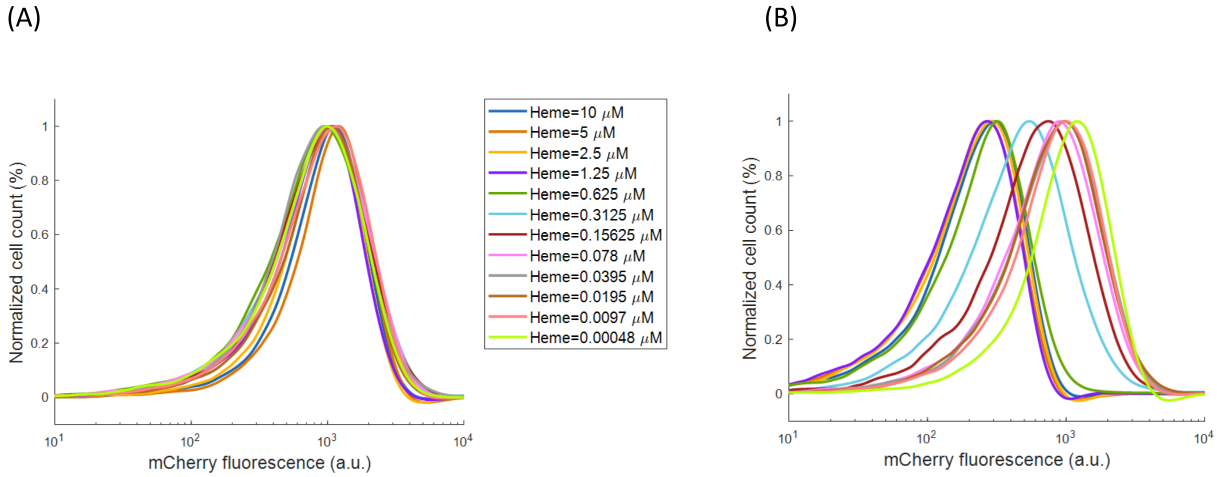

(C)

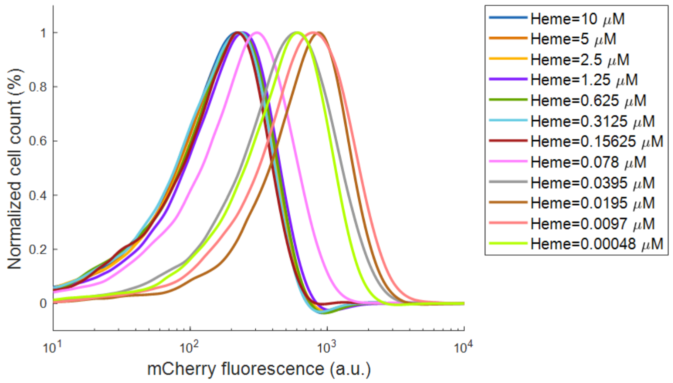

(D)

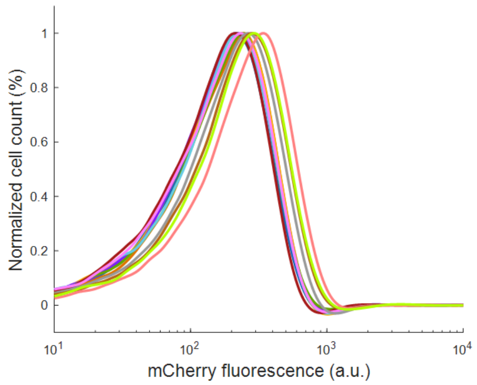

Fig. S46. Flow cytometry data for mCherry over bacterial cells population containing Heme-aTc inverting switch for ICF design of P_{LhrtO}. (A) [AHL]=0.1 μM, (B) [AHL]= 0.011 μM, (C) [AHL]= 0.0012 μM, (D) [AHL]= 0.00014 μM. This experimental data was used for generating Fig.S20C.

4.4 Experimental data for native specific promoter P_{arsR}

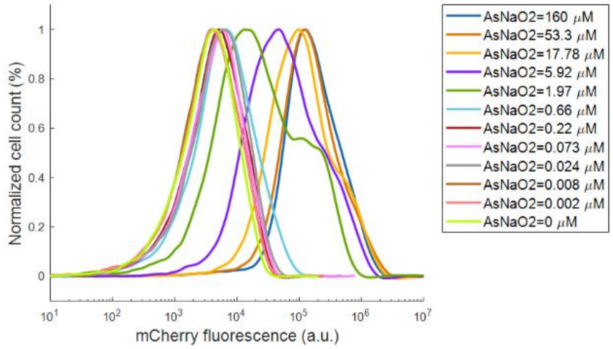

Fig. S47. Flow cytometry data for GFP over bacterial cells population containing P_{arsR} wild-type design. This experimental data was used for generating Fig.S21A.

(A)

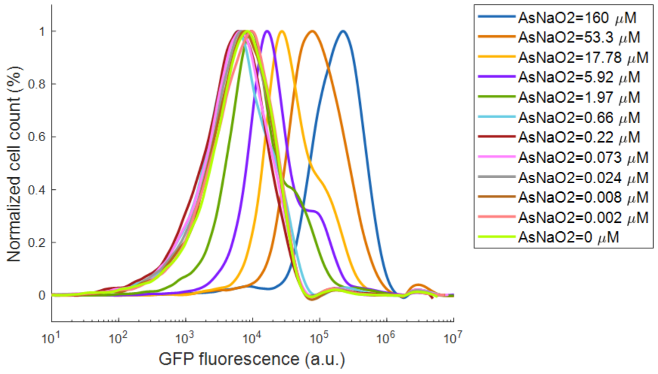

(B)

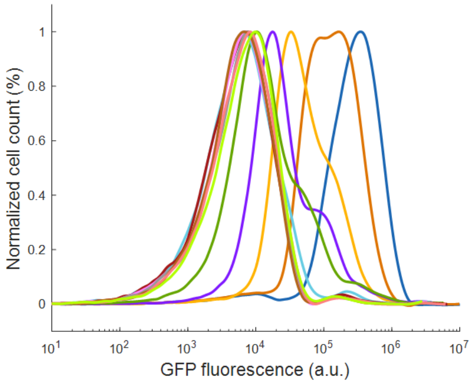

(C)

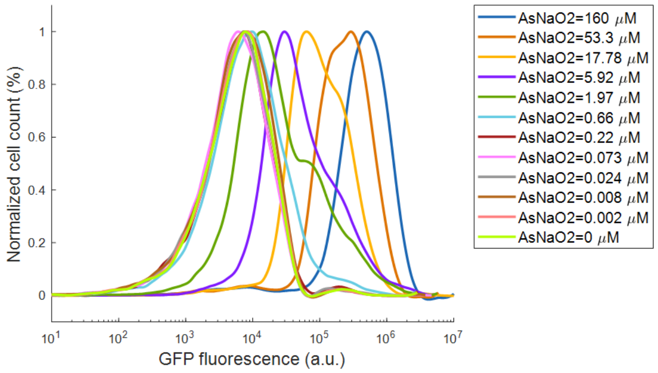
Normalized cell count (%)
0
0.2
0.4
0.6
0.8
1
10^{1}
10^{2}
10^{3}
10^{4}
10^{5}
10^{6}
10^{7}
GFP fluorescence (a.u.)
AsNaO2=160 μM
AsNaO2=53.3 μM
AsNaO2=17.78 μM
AsNaO2=5.92 μM
AsNaO2=1.97 μM
AsNaO2=0.66 μM
AsNaO2=0.22 μM
AsNaO2=0.073 μM
AsNaO2=0.024 μM
AsNaO2=0.008 μM
AsNaO2=0.002 μM
AsNaO2=0 μM

(D)

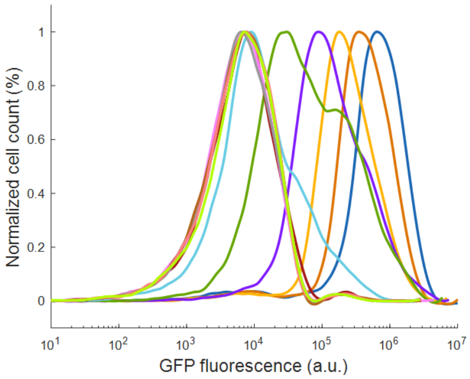
Normalized cell count (%)
0
0.2
0.4
0.6
0.8
1
10^{1}
10^{2}
10^{3}
10^{4}
10^{5}
10^{6}
10^{7}
GFP fluorescence (a.u.)

(E)

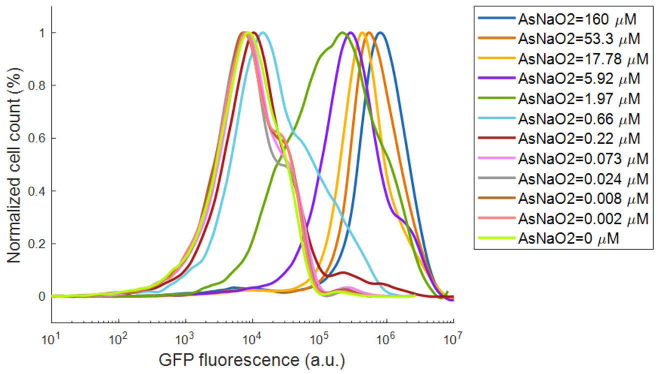
Normalized cell count (%)
0
0.2
0.4
0.6
0.8
1
10^{1}
10^{2}
10^{3}
10^{4}
10^{5}
10^{6}
10^{7}
GFP fluorescence (a.u.)
AsNaO2=160 μM
AsNaO2=53.3 μM
AsNaO2=17.78 μM
AsNaO2=5.92 μM
AsNaO2=1.97 μM
AsNaO2=0.66 μM
AsNaO2=0.22 μM
AsNaO2=0.073 μM
AsNaO2=0.024 μM
AsNaO2=0.008 μM
AsNaO2=0.002 μM
AsNaO2=0 μM

(F)

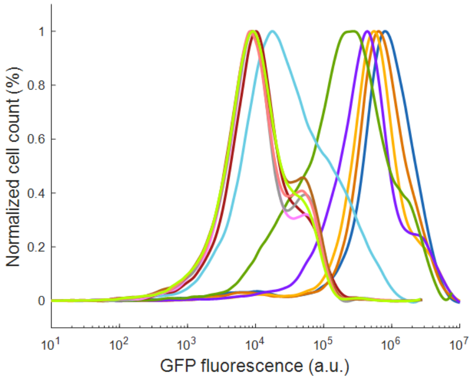
Normalized cell count (%)
0
0.2
0.4
0.6
0.8
1
10^{1}
10^{2}
10^{3}
10^{4}
10^{5}
10^{6}
10^{7}
GFP fluorescence (a.u.)

(G)

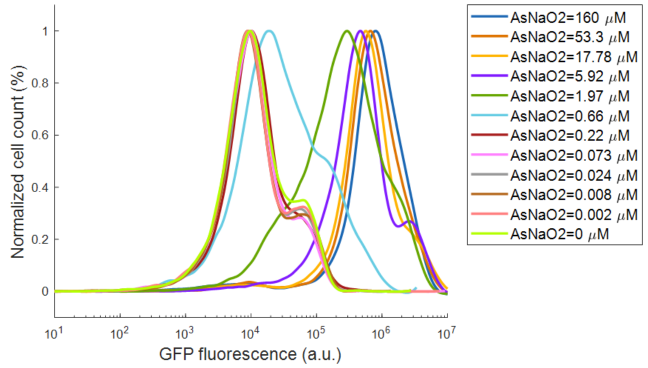
Normalized cell count (%)
0
0.2
0.4
0.6
0.8
1
10^{1}
10^{2}
10^{3}
10^{4}
10^{5}
10^{6}
10^{7}
GFP fluorescence (a.u.)
AsNaO2=160 μM
AsNaO2=53.3 μM
AsNaO2=17.78 μM
AsNaO2=5.92 μM
AsNaO2=1.97 μM
AsNaO2=0.66 μM
AsNaO2=0.22 μM
AsNaO2=0.073 μM
AsNaO2=0.024 μM
AsNaO2=0.008 μM
AsNaO2=0.002 μM
AsNaO2=0 μM

(H)

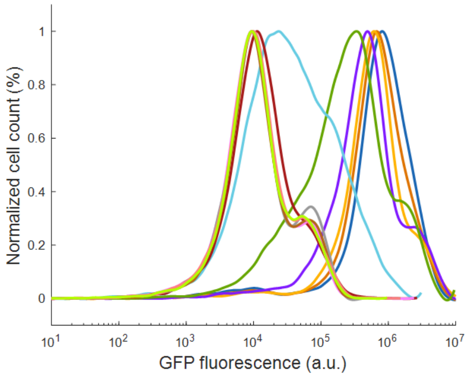
Normalized cell count (%)
0
0.2
0.4
0.6
0.8
1
10^{1}
10^{2}
10^{3}
10^{4}
10^{5}
10^{6}
10^{7}
GFP fluorescence (a.u.)

(I)

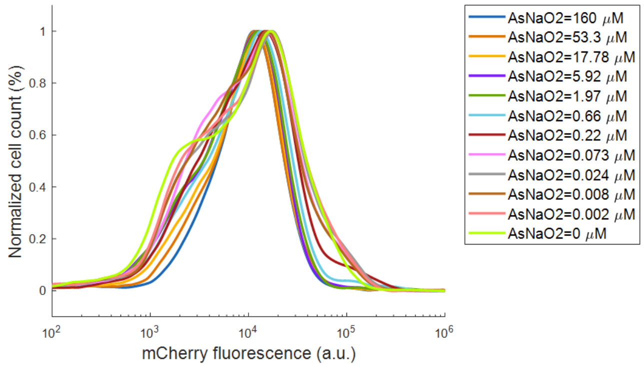

(J)

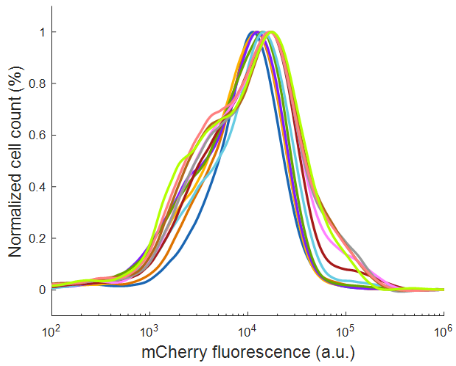

(K)

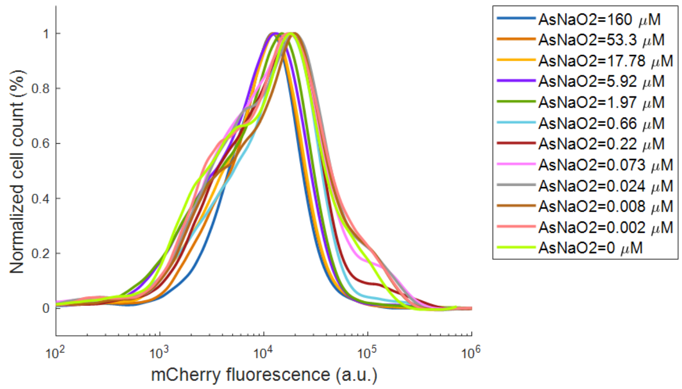

(L)

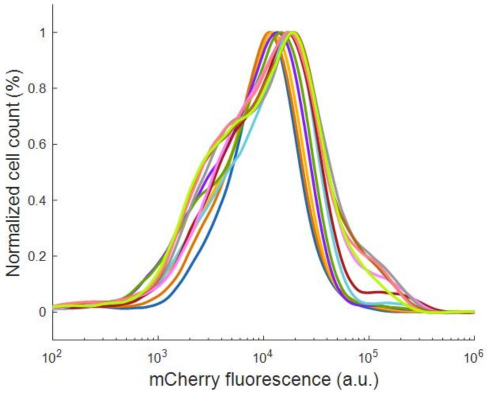

Fig. S48. Flow cytometry data for GFP over bacterial cells population containing OL design of P_{arsR}. (A) [AHL]=0.1371μM, (B) [AHL]=0.0457 μM, (C) [AHL]=0.0152 μM, (D) [AHL]=0.0051 μM, (E) [AHL]=0.0017 μM, (F) [AHL]=0.00056 μM, (G) [AHL]=0.00019 μM, (H) [AHL]=0 μM. (I-L) Flow cytometry data for mCherry over bacterial cells population containing OL design. (I) [AHL]=0.0457 μM, (J) [AHL]= 0.0051 μM, (K) [AHL]= 0.00056 μM, (L) [AHL]= 0 μM. This experimental data was used for generating Fig.7E and Fig.S21B.

(A)

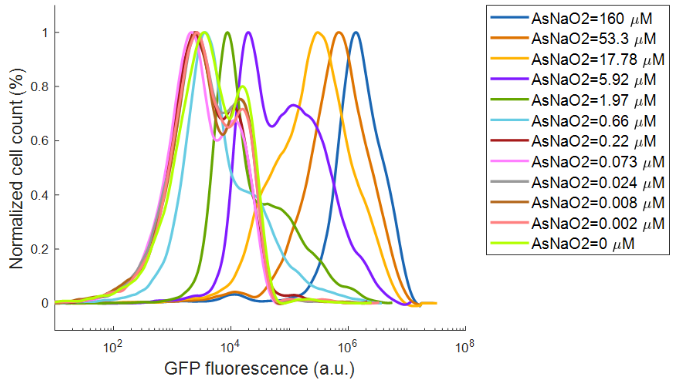

(B)

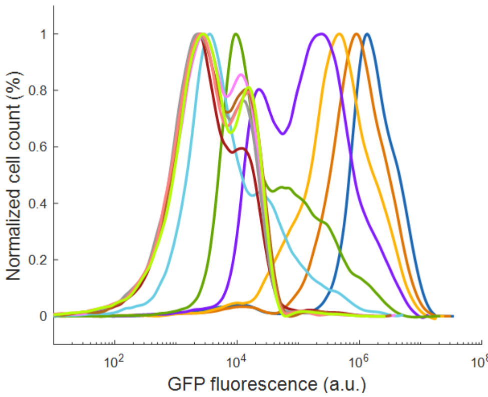

(C)

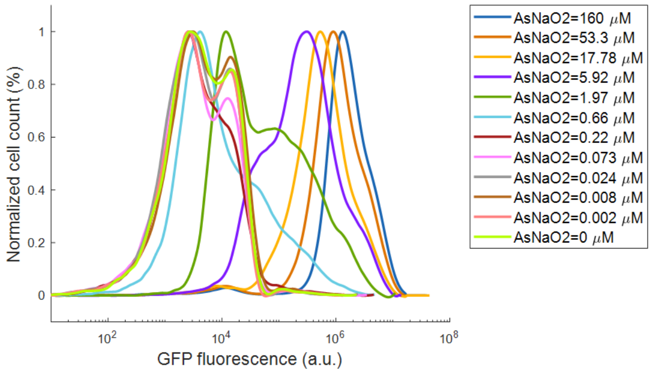

(D)

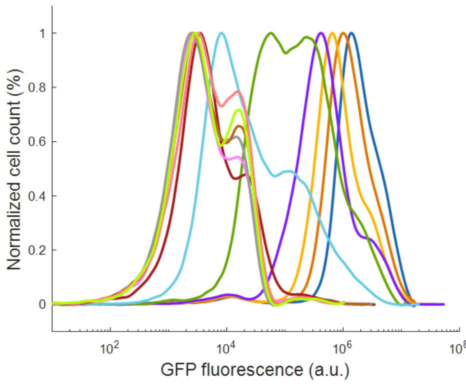

(E)

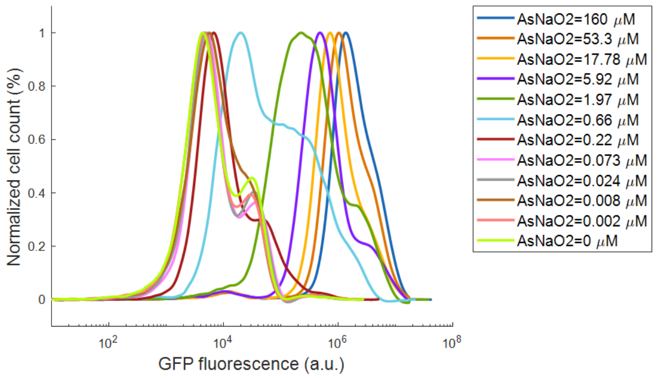

(F)

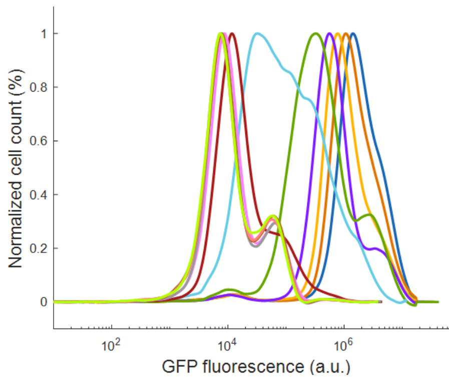

(G)

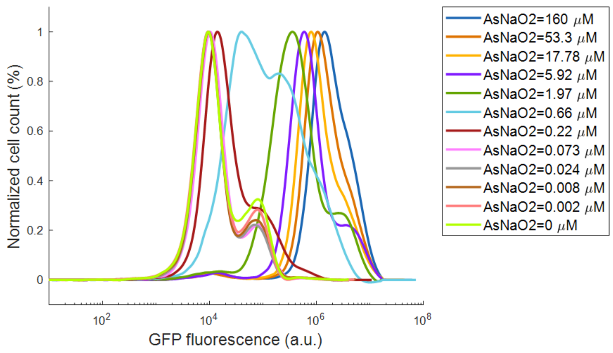

(H)

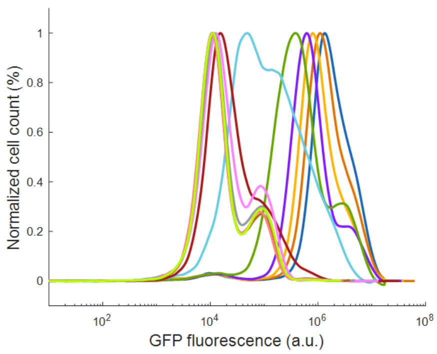

Fig. S49. Flow cytometry data for GFP over bacterial cells population containing ICF design of P_{arsR}. (A) [AHL]=0.1371 μM, (B) [AHL]=0.0457 μM, (C) [AHL]=0.0152 μM, (D) [AHL]=0.0051 μM, (E) [AHL]=0.0017 μM, (F) [AHL]=0.00056 μM, (G) [AHL]=0.00019 μM, (H) [AHL]=0 μM. This experimental data was used for generating Fig.7F and Fig.S21D.

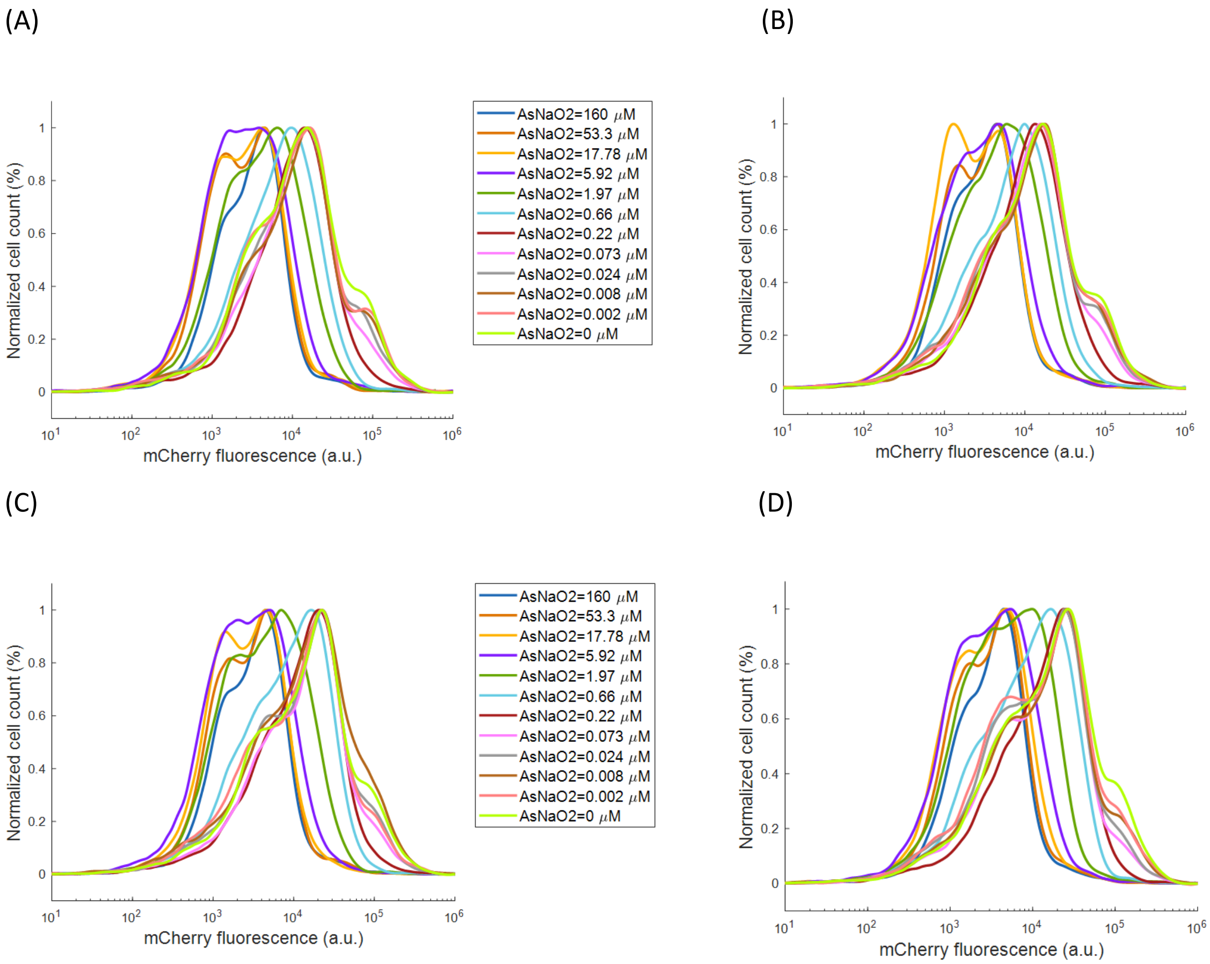

Fig. S50. Flow cytometry data for mCherry over bacterial cells population containing ICF design of P_{arsR}. (A) [AHL]=0.1371 μM, (B) [AHL]= 0.0152 μM, (C) [AHL]= 0.0017 μM, (D) [AHL]= 0.00019 μM. This experimental data was used for generating Fig.S21D.

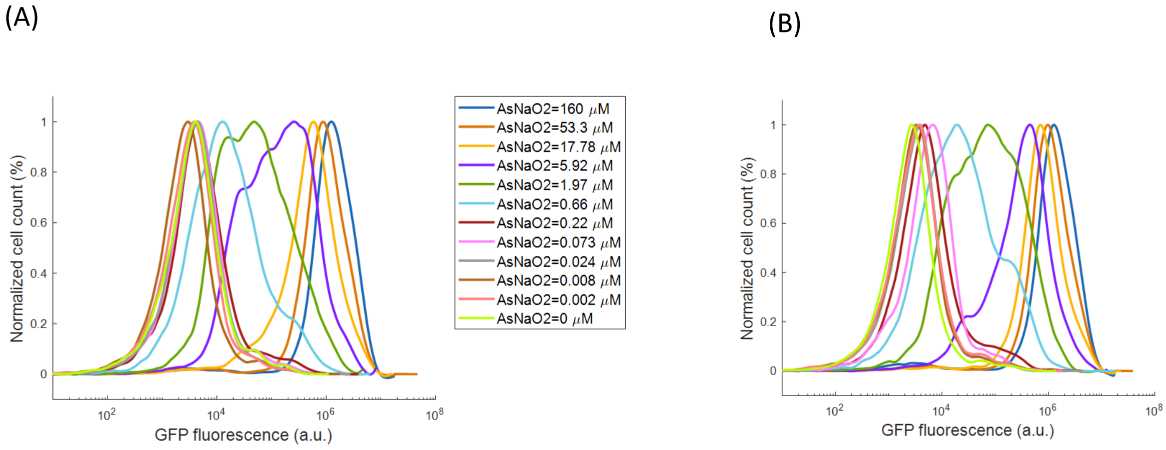

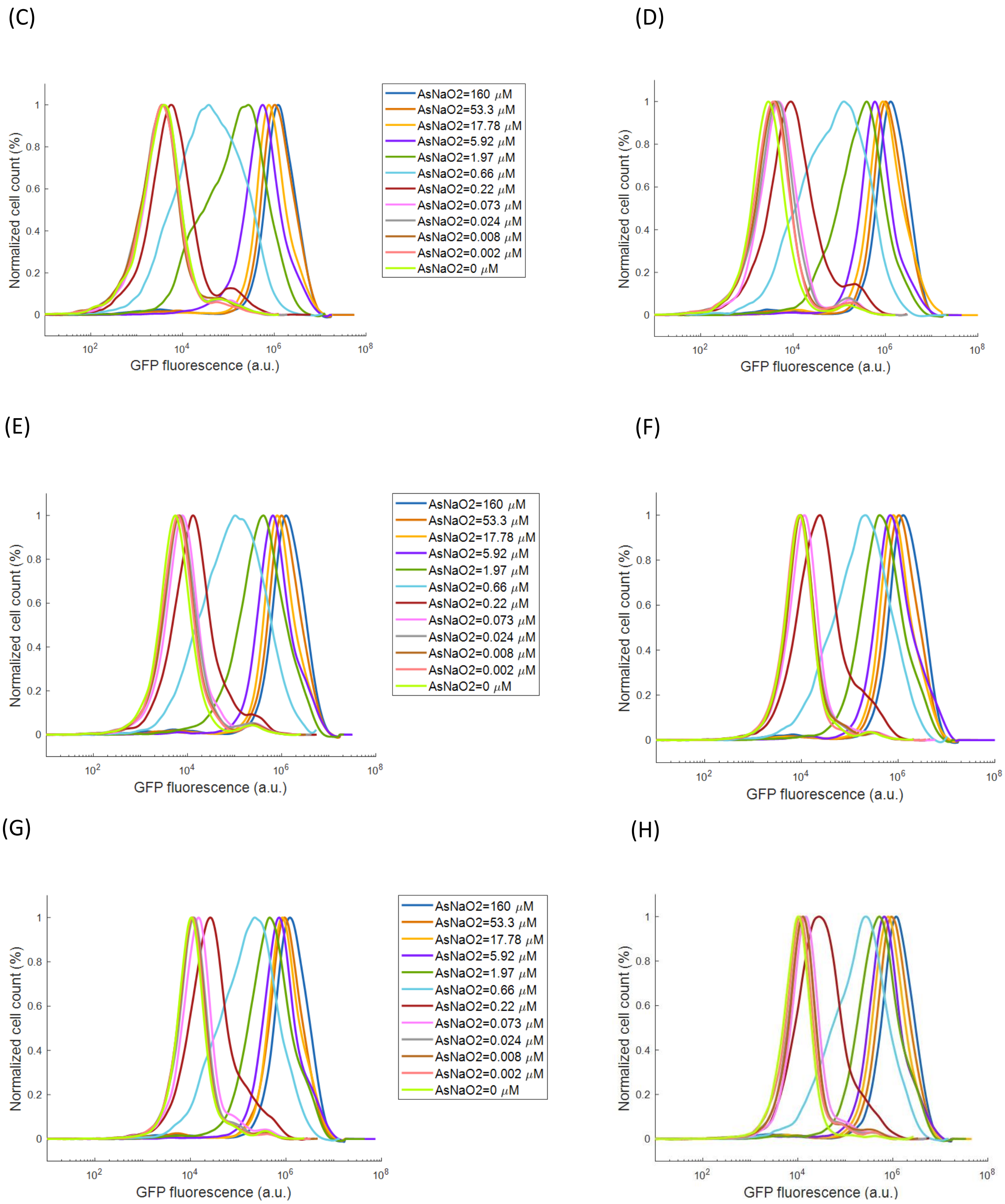

Fig. S51. Flow cytometry data for GFP over bacterial cells population containing DNF design of P_{arsR}. (A) [AHL]=0.1371 μM, (B) [AHL]=0.0457 μM, (C) [AHL]=0.0152 μM, (D) [AHL]=0.0051 μM, (E) [AHL]=0.0017 μM, (F) [AHL]=0.00056 μM, (G) [AHL]=0.00019 μM, (H) [AHL]=0 μM. This experimental data was used for generating Fig.S21E.

(A)

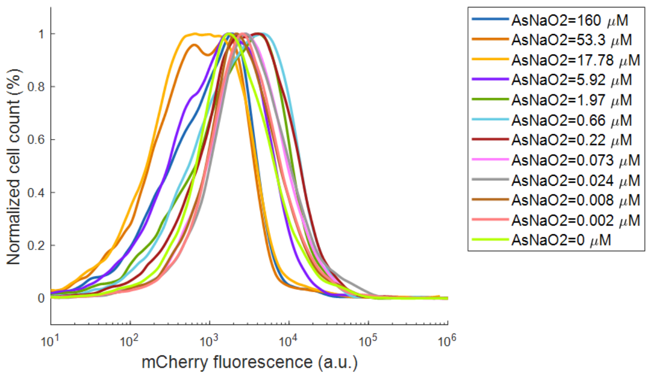

(B)

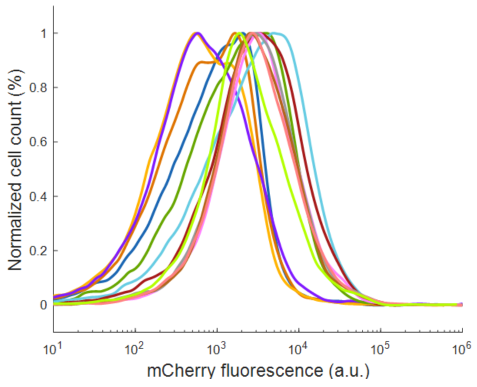

(C)

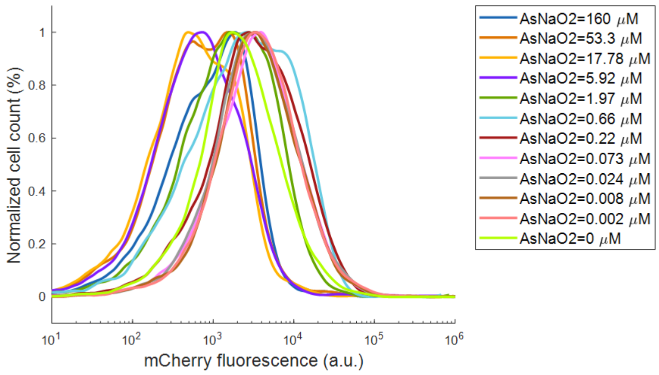

(D)

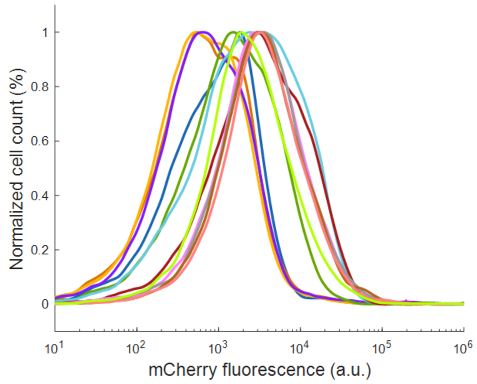

Fig. S52. Flow cytometry data for mCherry over bacterial cells population containing DNF design of P_{arsR}. (A) [AHL]=0.1371 μM, (B) [AHL]= 0.0152 μM, (C) [AHL]= 0.0017 μM, (D) [AHL]= 0.00019 μM. This experimental data was used for generating Fig.S21E.

(A)

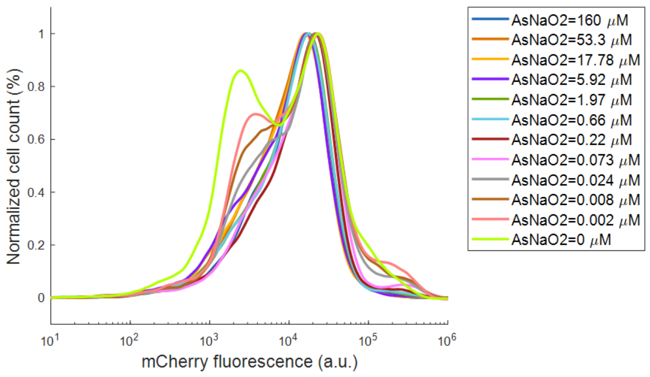

(B)

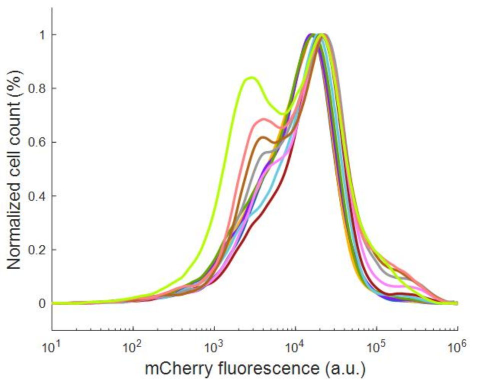

(C)

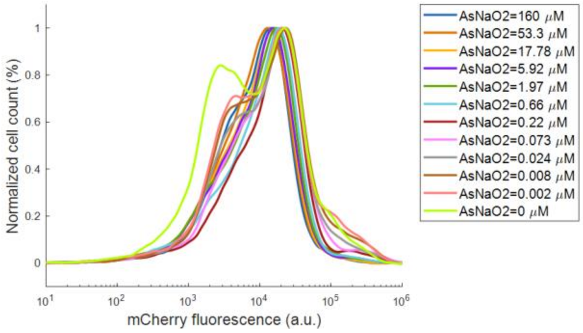

(D)

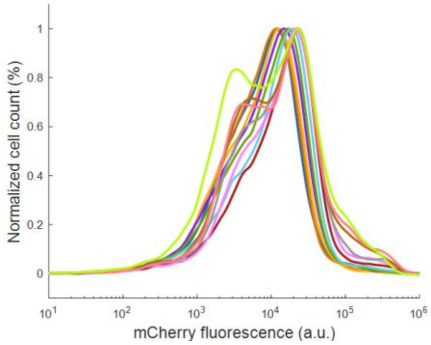

Fig. S53. Flow cytometry data for mCherry over bacterial cells population containing $AsNaO_2$ -aTc inverting switch design of P_{arsR}. (A) [aTc]=100ngr/ml, (B) [aTc]= 11.1ngr/ml, (C) [aTc]= 1.23ngr/ml, (D) [aTc]= 0.137 ngr/ml. This experimental data was used for generating Fig.S21C.

4.5 Experimental data for native stress promoter P_{katG}

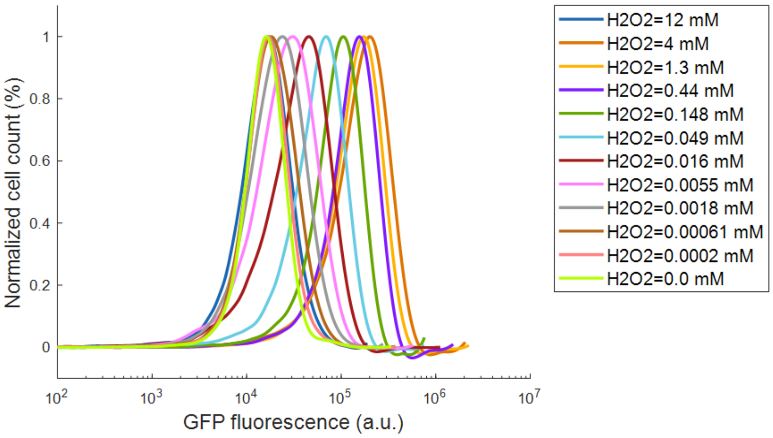

Fig. S54. Flow cytometry data for GFP over bacterial cells population containing wild-type design of P_{katG}. This experimental data was used for generating Fig.S22A.

(A)

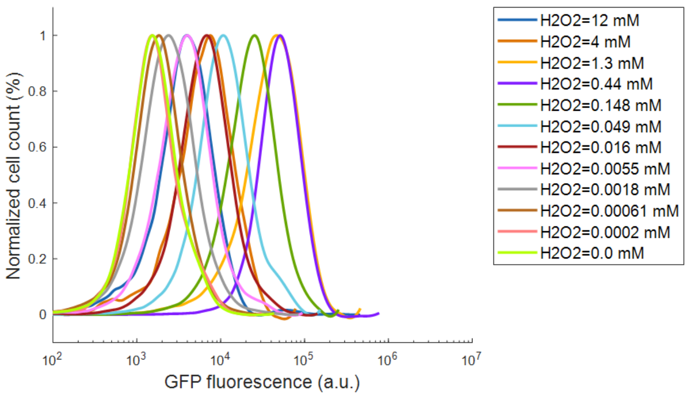

(B)

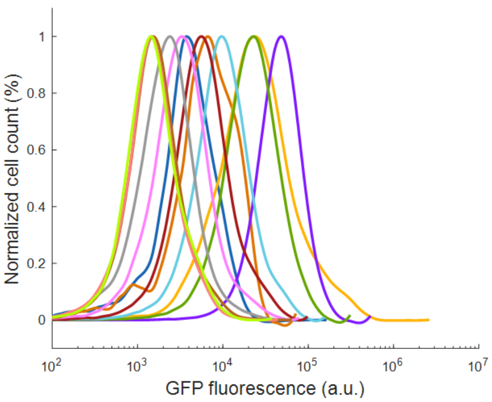

(C)

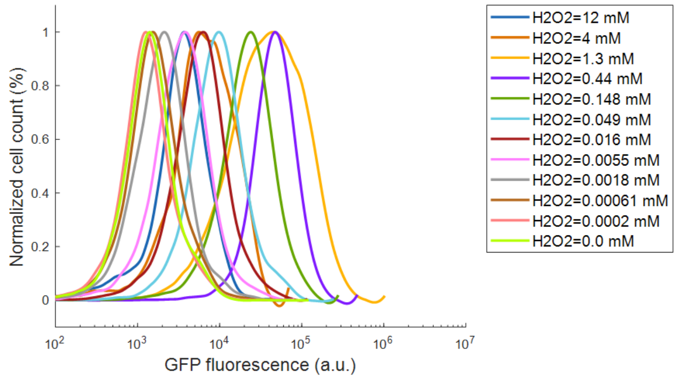

(D)

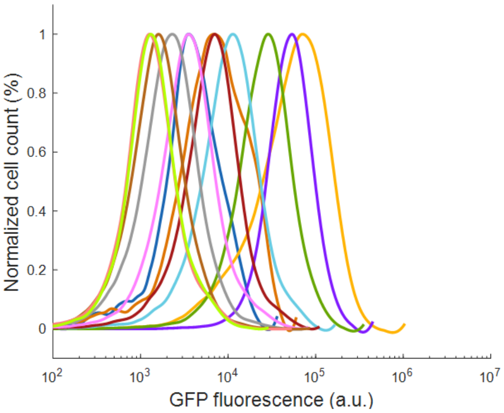

(E)

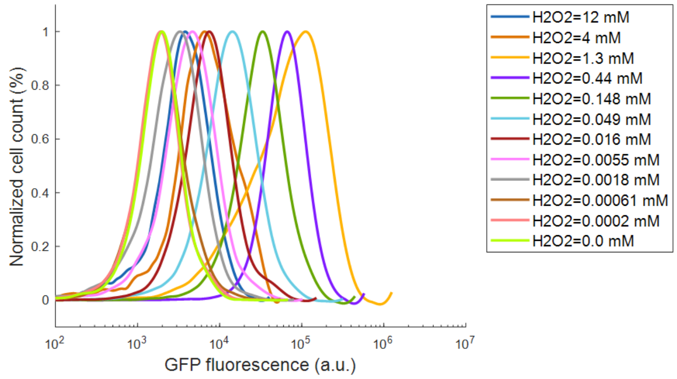

(F)

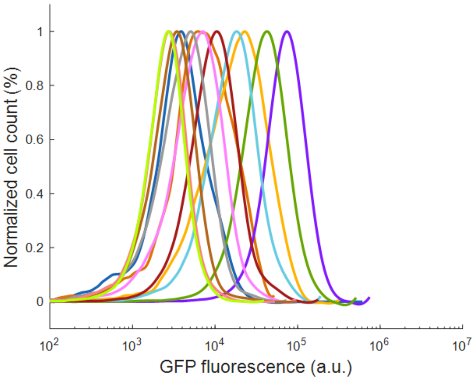

(G)

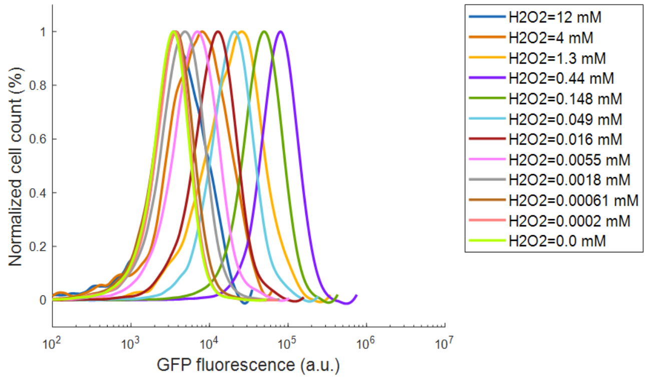

(H)

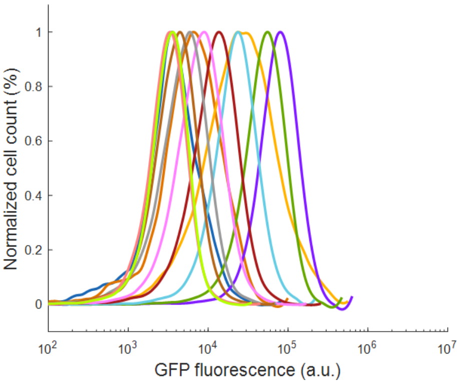

Fig. S55. Flow cytometry data for GFP over bacterial cells population containing OL design of P_{katG}. (A) [AHL]=0.4115 µM, (B) [AHL]=0.1371 µM, (C) [AHL]=0.0457 µM, (D) [AHL]=0.0152 µM, (E) [AHL]=0.0051 µM, (F) [AHL]=0.0017 µM, (G) [AHL]=0.00056 µM, (H) [AHL]=0 µM. This experimental data was used for generating Fif.8A and Fig.S22B.

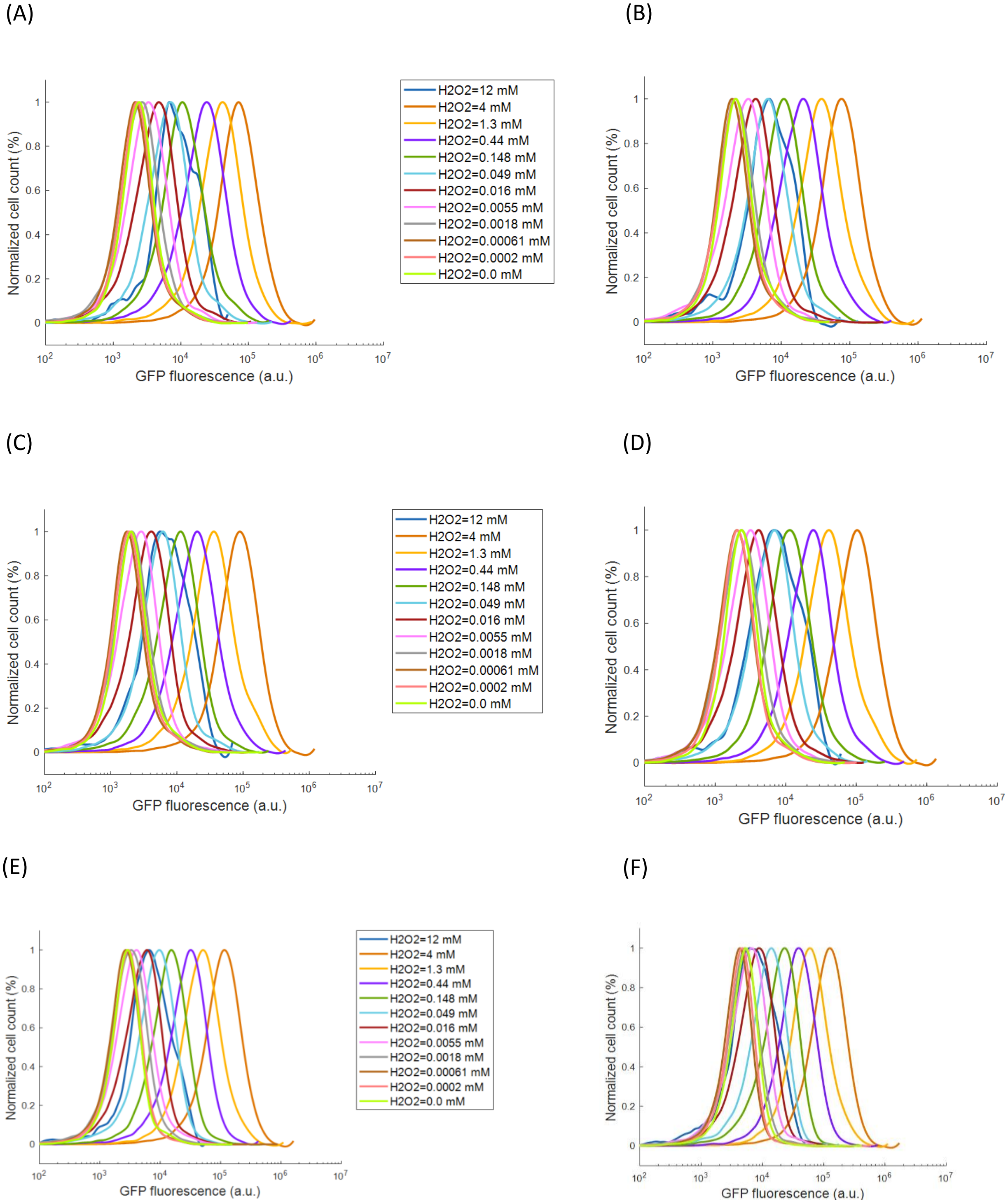
(A)
(B)
(C)
(D)
(E)
(F)
Normalized cell count (%)
GFP fluorescence (a.u.)
H2O2=12 mM
H2O2=4 mM
H2O2=1.3 mM
H2O2=0.44 mM
H2O2=0.148 mM
H2O2=0.049 mM
H2O2=0.016 mM
H2O2=0.0055 mM
H2O2=0.0018 mM
H2O2=0.00061 mM
H2O2=0.0002 mM
H2O2=0.0 mM

(G)

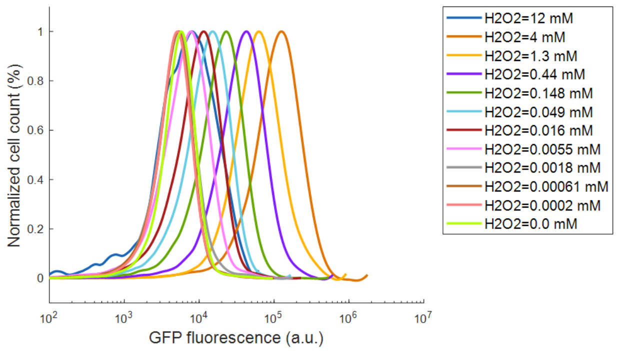

(H)

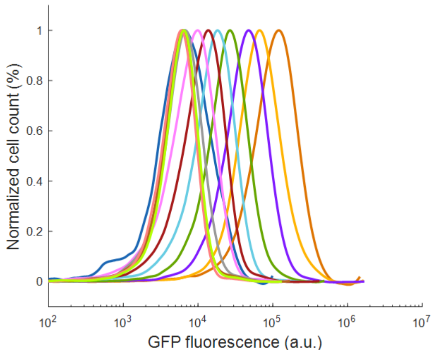

(I)

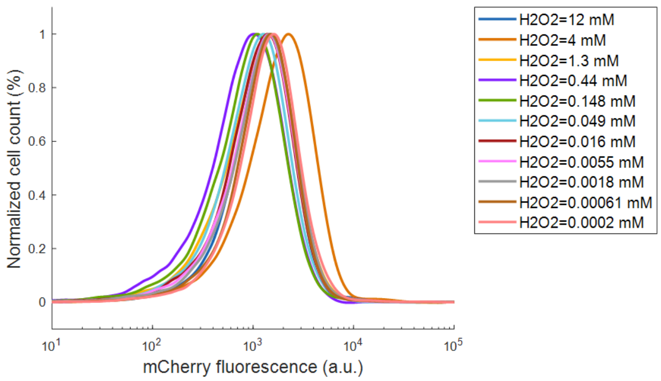

(J)

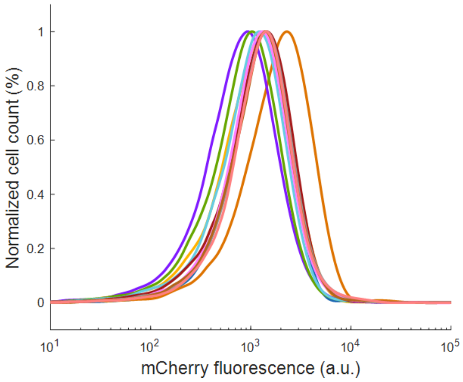

Fig. S56. Flow cytometry data for GFP over bacterial cells population containing ICF design of P_{katG}. (A) [AHL]=0.4115 µM, (B) [AHL]=0.1371 µM, (C) [AHL]=0.0457 µM, (D) [AHL]=0.0152 µM, (E) [AHL]=0.0051 µM, (F) [AHL]=0.0017 µM, (G) [AHL]=0.00056 µM, (H) [AHL]=0 µM. (I-J) Flow cytometry data for mCherry over bacterial cells population containing ICF design. (I) [AHL]=0.0017 µM, (J) [AHL]=0.0 µM. This experimental data was used for generating Fif.8C and Fig.S22D.

(A)

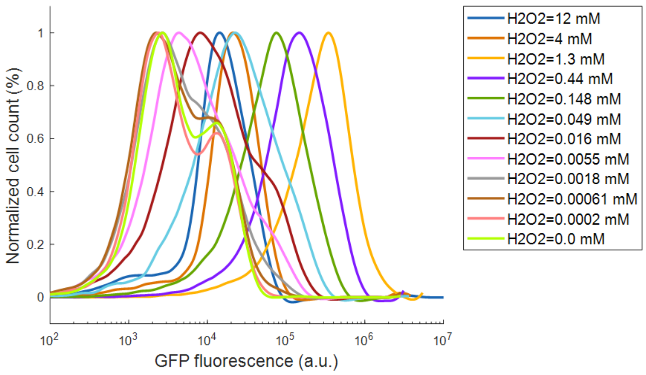

(B)

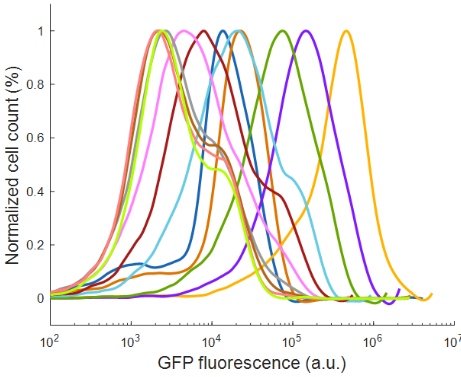

(C)

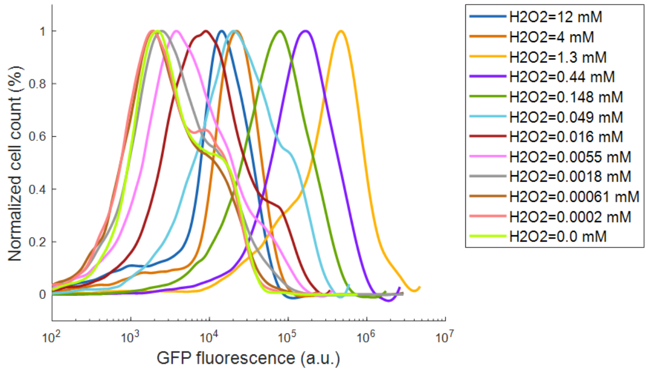
H2O2=12 mM
H2O2=4 mM
H2O2=1.3 mM
H2O2=0.44 mM
H2O2=0.148 mM
H2O2=0.049 mM
H2O2=0.016 mM
H2O2=0.0055 mM
H2O2=0.0018 mM
H2O2=0.00061 mM
H2O2=0.0002 mM
H2O2=0.0 mM
Normalized cell count (%)
GFP fluorescence (a.u.)

(D)

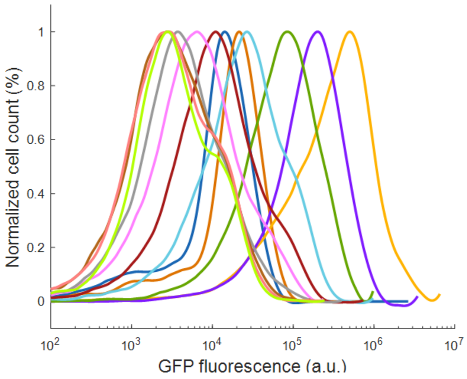
Normalized cell count (%)
GFP fluorescence (a.u.)

(E)

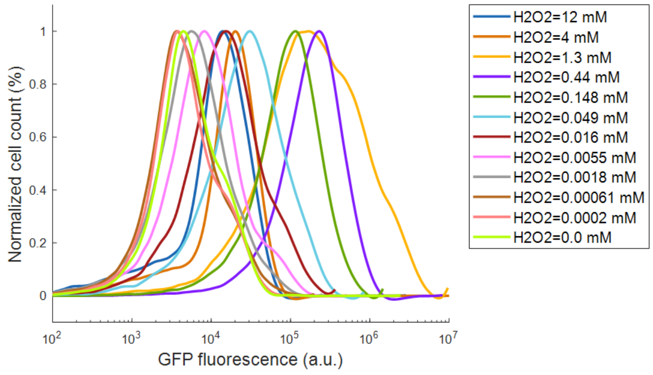
H2O2=12 mM
H2O2=4 mM
H2O2=1.3 mM
H2O2=0.44 mM
H2O2=0.148 mM
H2O2=0.049 mM
H2O2=0.016 mM
H2O2=0.0055 mM
H2O2=0.0018 mM
H2O2=0.00061 mM
H2O2=0.0002 mM
H2O2=0.0 mM
Normalized cell count (%)
GFP fluorescence (a.u.)

(F)

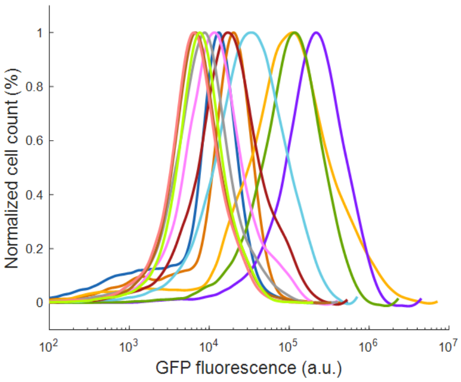
Normalized cell count (%)
GFP fluorescence (a.u.)

(G)

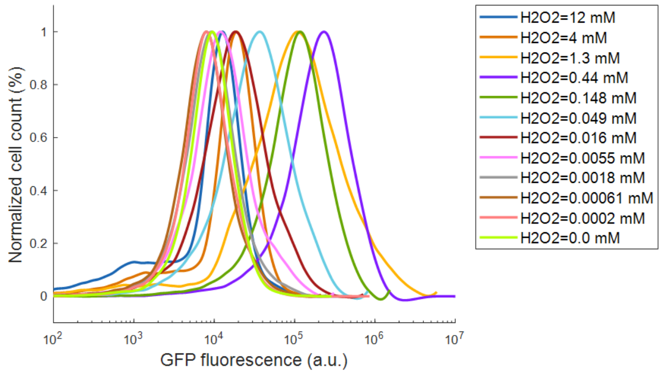
H2O2=12 mM
H2O2=4 mM
H2O2=1.3 mM
H2O2=0.44 mM
H2O2=0.148 mM
H2O2=0.049 mM
H2O2=0.016 mM
H2O2=0.0055 mM
H2O2=0.0018 mM
H2O2=0.00061 mM
H2O2=0.0002 mM
H2O2=0.0 mM
Normalized cell count (%)
GFP fluorescence (a.u.)

(H)

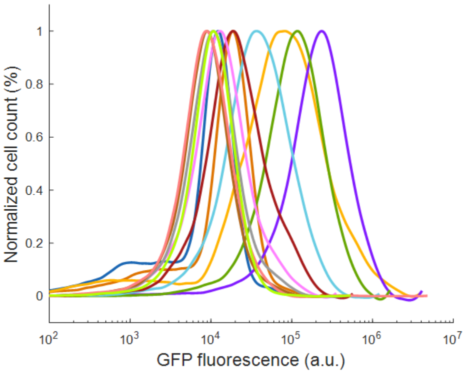
Normalized cell count (%)
GFP fluorescence (a.u.)

(I)

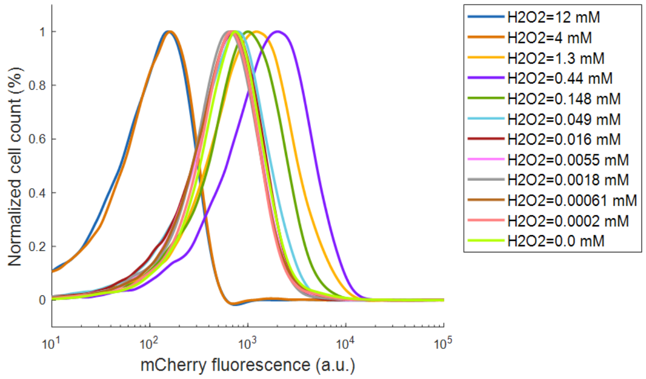

(J)

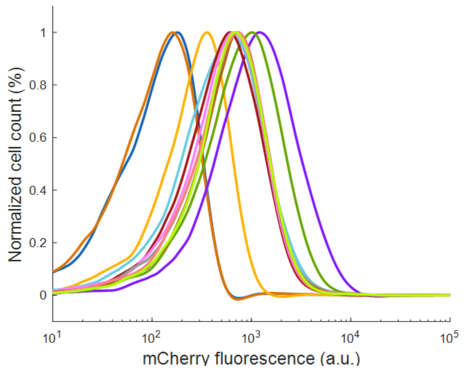

Fig. S57. Flow cytometry data for GFP over bacterial cells population containing DNF design of P_{katG}. (A) [AHL]=0.4115 µM, (B) [AHL]=0.1371 µM, (C) [AHL]=0.0457 µM, (D) [AHL]=0.0152 µM, (E) [AHL]=0.0051 µM, (F) [AHL]=0.0017 µM, (G) [AHL]=0.00056 µM, (H) [AHL]=0 µM. (I-J) Flow cytometry data for mCherry over bacterial cells population containing DNF design. (I) [AHL]=0.4115 µM, (J) [AHL]=0.0017 µM. This experimental data was used for generating Fig.S22E.

(A)

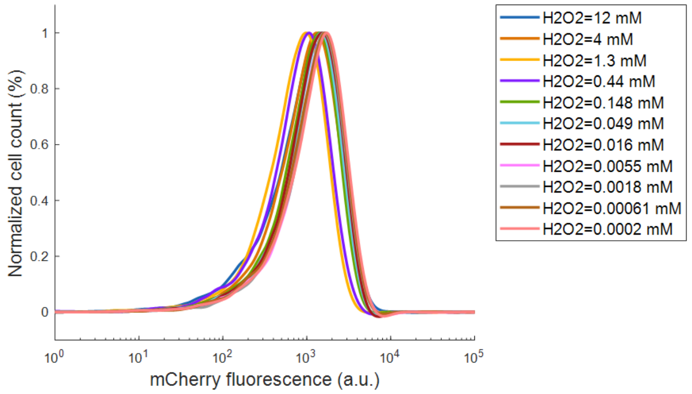

(B)

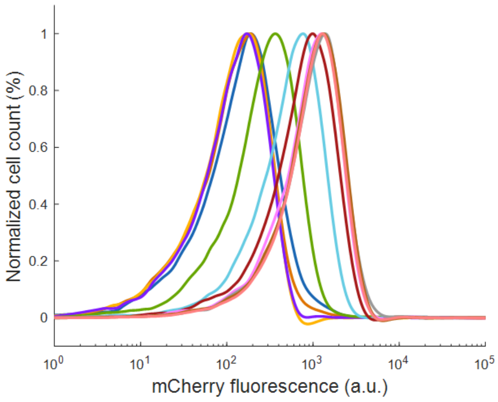

(C)

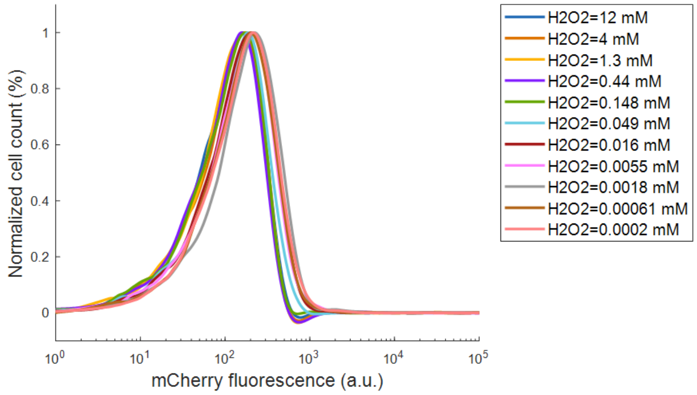

(D)

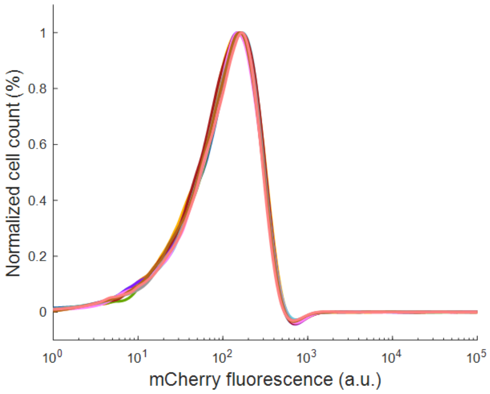

Fig. S58. Flow cytometry data for mCherry over bacterial cells population containing H_2O_2-aTc inverting switch design of P_{katG}. (A) [aTc]=33.33ngr/ml, (B) [aTc]=11.11ngr/ml, (C) [aTc]=3.7ngr/ml, (D) [aTc]=1.23ngr/ml. This experimental data was used for generating Fig.8B and Fig.S22C.

4.6 Experimental data for native stress promoter P_{recA}

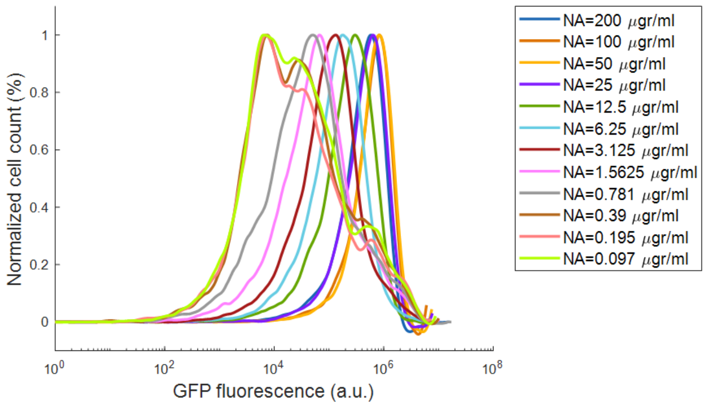

Fig. S59. Flow cytometry data for GFP over bacterial cells population containing P_{recA} wild-type design. This experimental data was used for generating Fig.S23A.

(A)

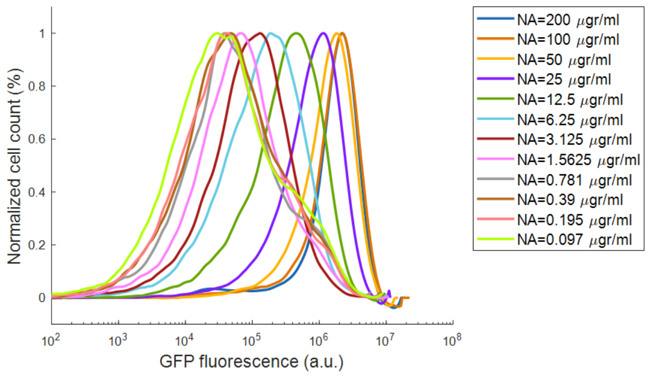

(B)

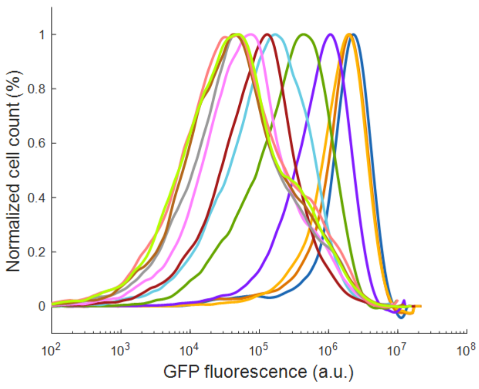

(C)

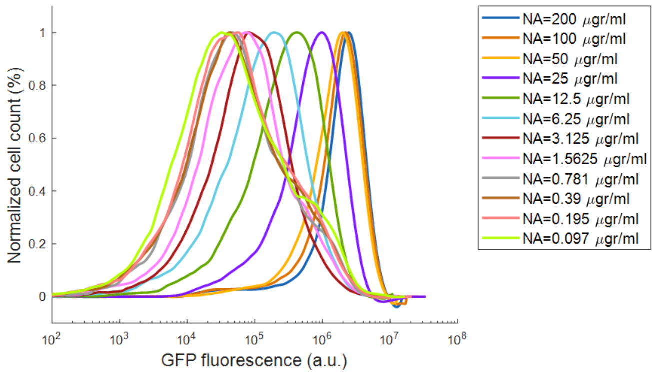

(D)

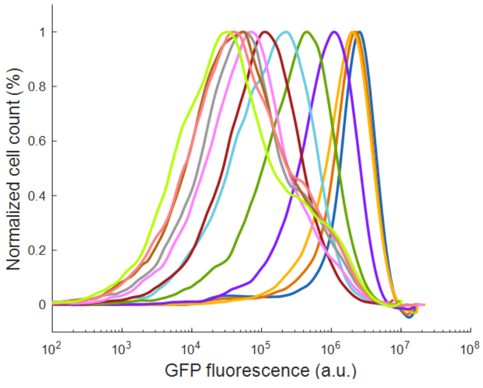

(E)

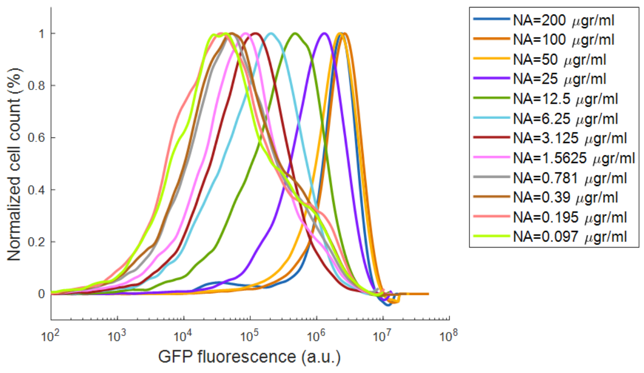

(F)

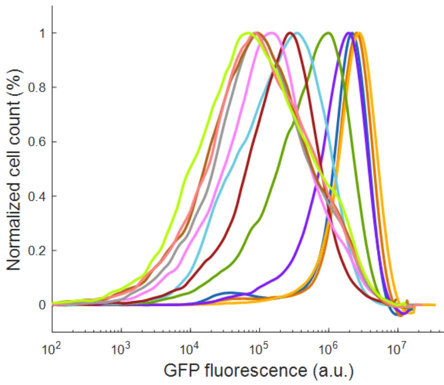

(G)

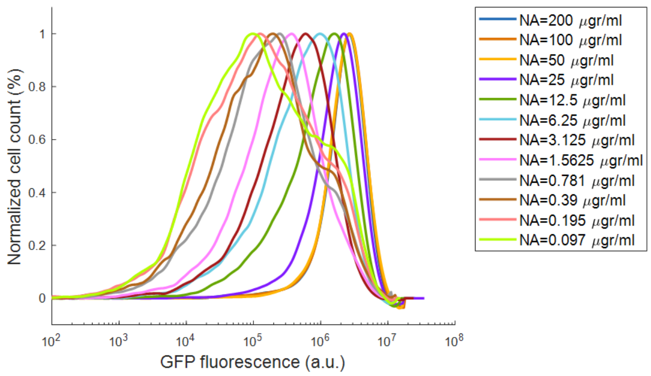

(H)

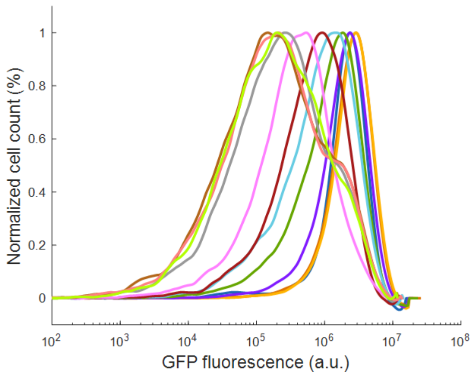

(I)

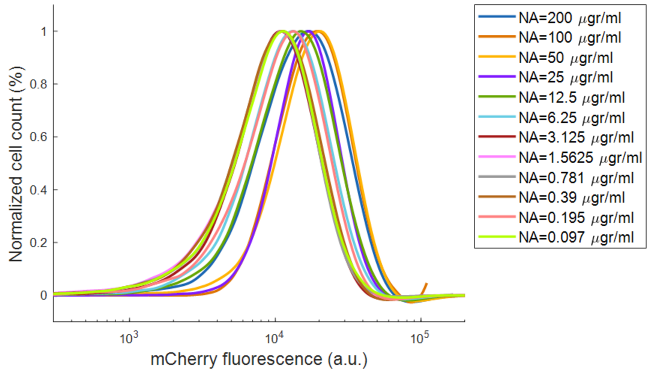

(J)

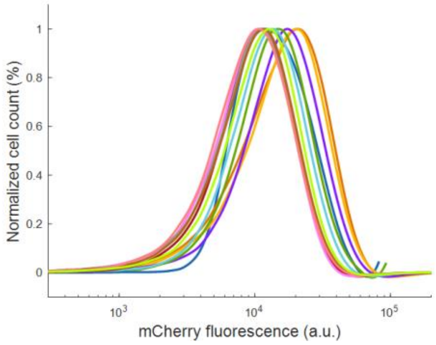

Fig. S60. Flow cytometry data for GFP over bacterial cells population containing AT-OL design of P_{recA}. (A) [AHL]=0.1371 μM, (B) [AHL]=0.0457 μM, (C) [AHL]=0.0152 μM, (D) [AHL]=0.0051 μM, (E) [AHL]=0.0017 μM, (F) [AHL]=0.00056 μM, (G) [AHL]=0.0002 μM, (H) [AHL]=0 μM. (I-J) Flow cytometry data for mCherry over bacterial cells population containing AT-OL design. (I) [AHL]=0.0152 μM, (J) [AHL]=0.0017 μM. This experimental data was used for generating Fig.S23B.

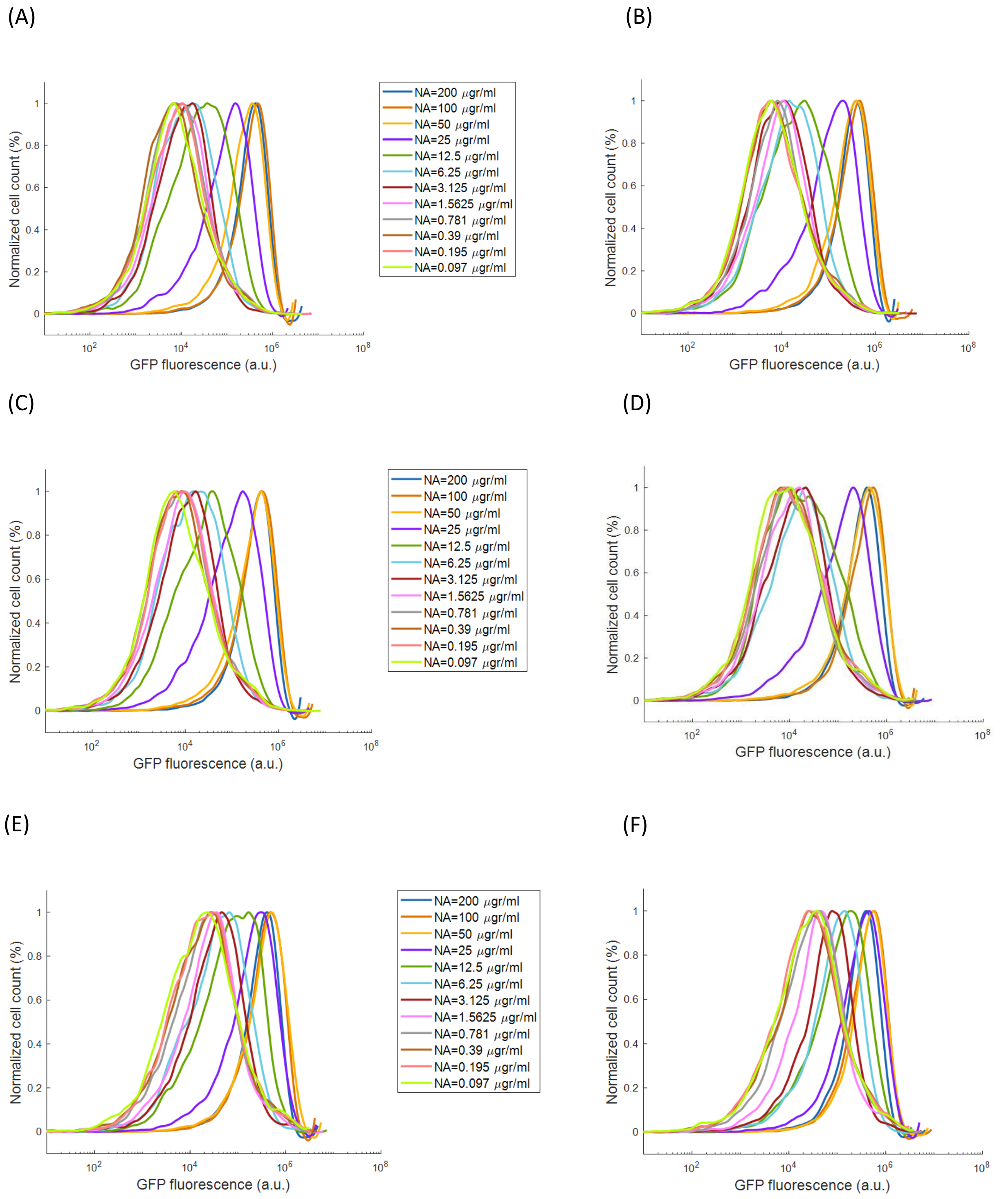
(A)
(B)
(C)
(D)
(E)
(F)
Normalized cell count (%)
GFP fluorescence (a.u.)
NA=200 μgr/ml
NA=100 μgr/ml
NA=50 μgr/ml
NA=25 μgr/ml
NA=12.5 μgr/ml
NA=6.25 μgr/ml
NA=3.125 μgr/ml
NA=1.5625 μgr/ml
NA=0.781 μgr/ml
NA=0.39 μgr/ml
NA=0.195 μgr/ml
NA=0.097 μgr/ml

(G)

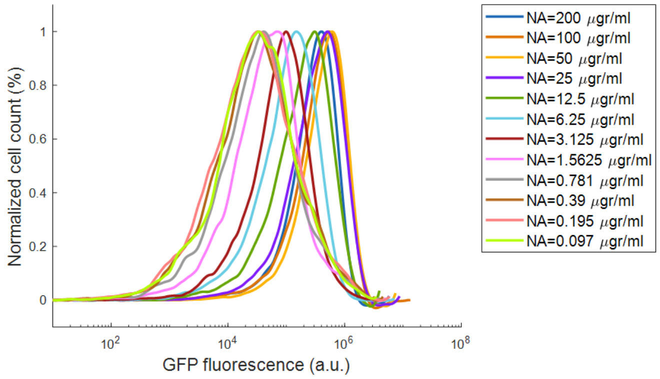

(H)

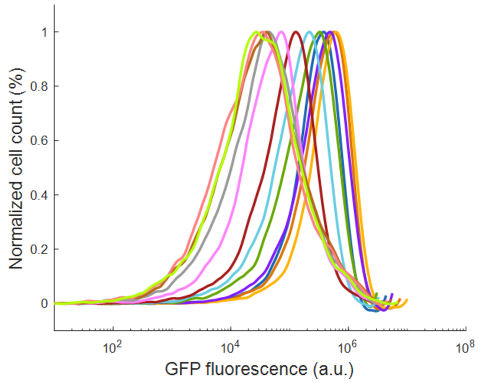

Fig. S61. Flow cytometry data for GFP over bacterial cells population containing TI-OL design of P_{recA}. (A) [AHL]=0.1371 μM, (B) [AHL]=0.0457 μM, (C) [AHL]=0.0152 μM, (D) [AHL]=0.0051 μM, (E) [AHL]=0.0017 μM, (F) [AHL]=0.00056 μM, (G) [AHL]=0.0002 μM, (H) [AHL]=0.0 μM. This experimental data was used for generating Fig.8E and Fig.S23C.

(A)

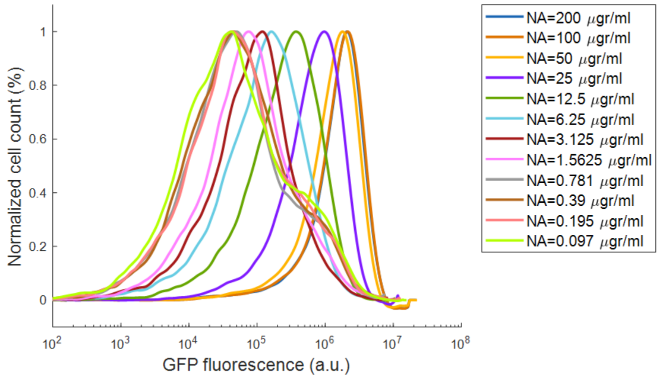

(B)

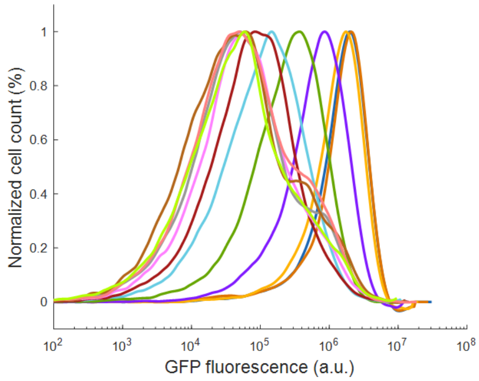

(C)

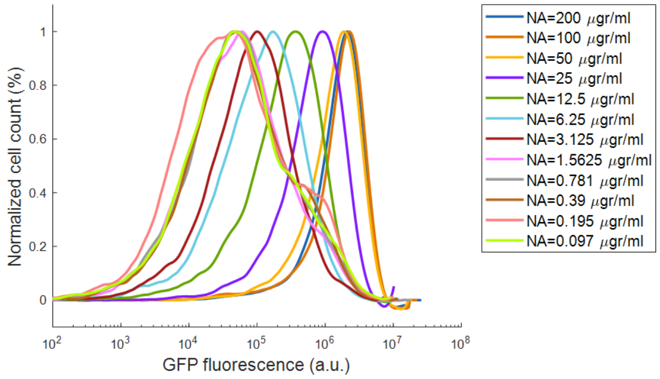

(D)

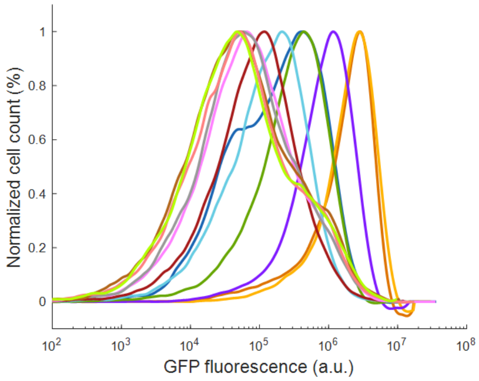

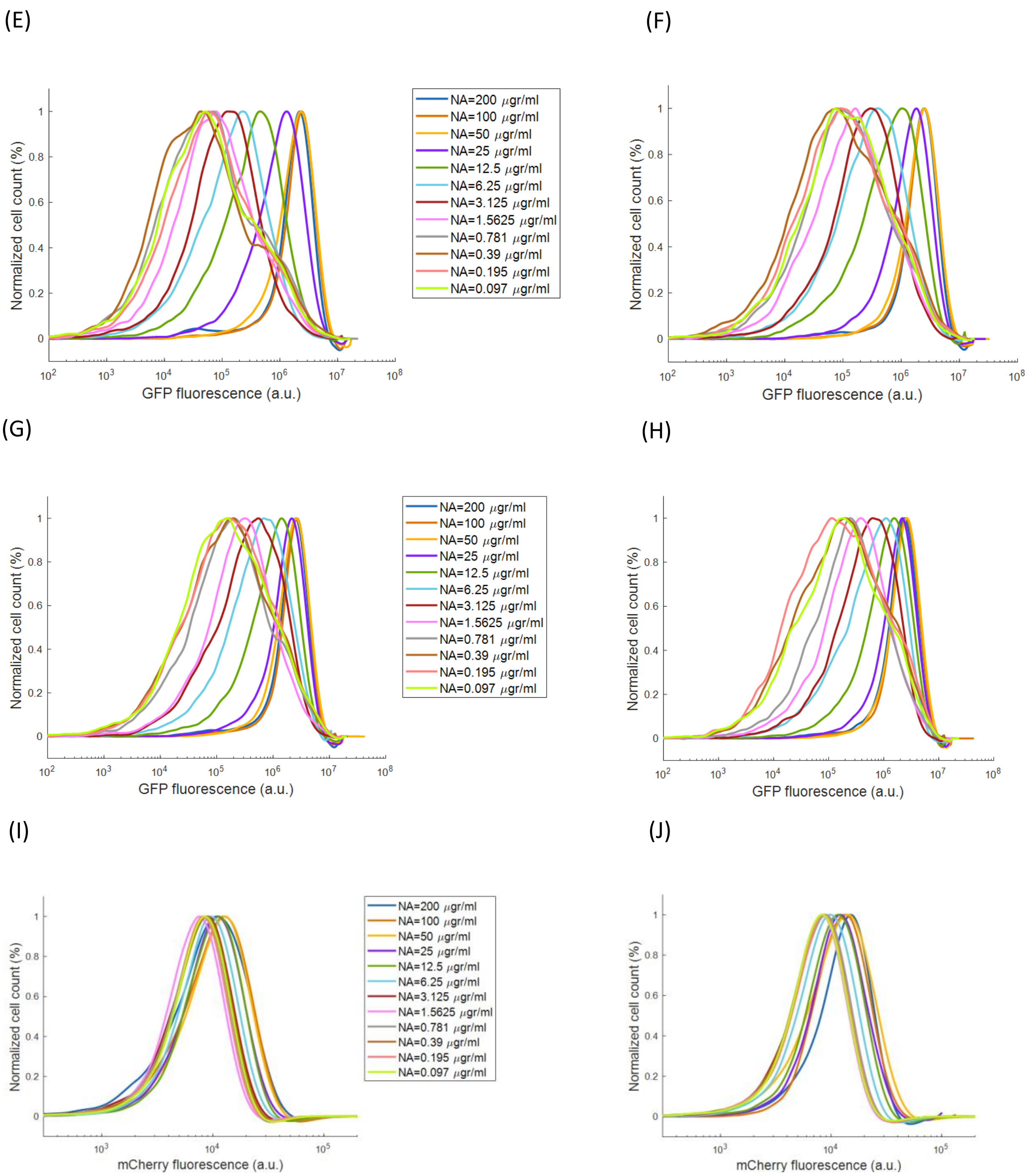

Fig. S62. Flow cytometry data for GFP over bacterial cells population containing ICF design of P_{recA}. (A) [AHL]=0.1371 μM, (B) [AHL]=0.0457 μM, (C) [AHL]=0.0152 μM, (D) [AHL]=0.0051 μM, (E) [AHL]=0.0017 μM, (F) [AHL]=0.00056 μM, (G) [AHL]=0.0002 μM, (H) [AHL]=0 μM. (I-J) Flow cytometry data for mCherry over bacterial cells population containing ICF design. (I) [AHL]=0.0152 μM, (J) [AHL]=0.0051 μM. This experimental data was used for generating Fig.8F and Fig.S23E.

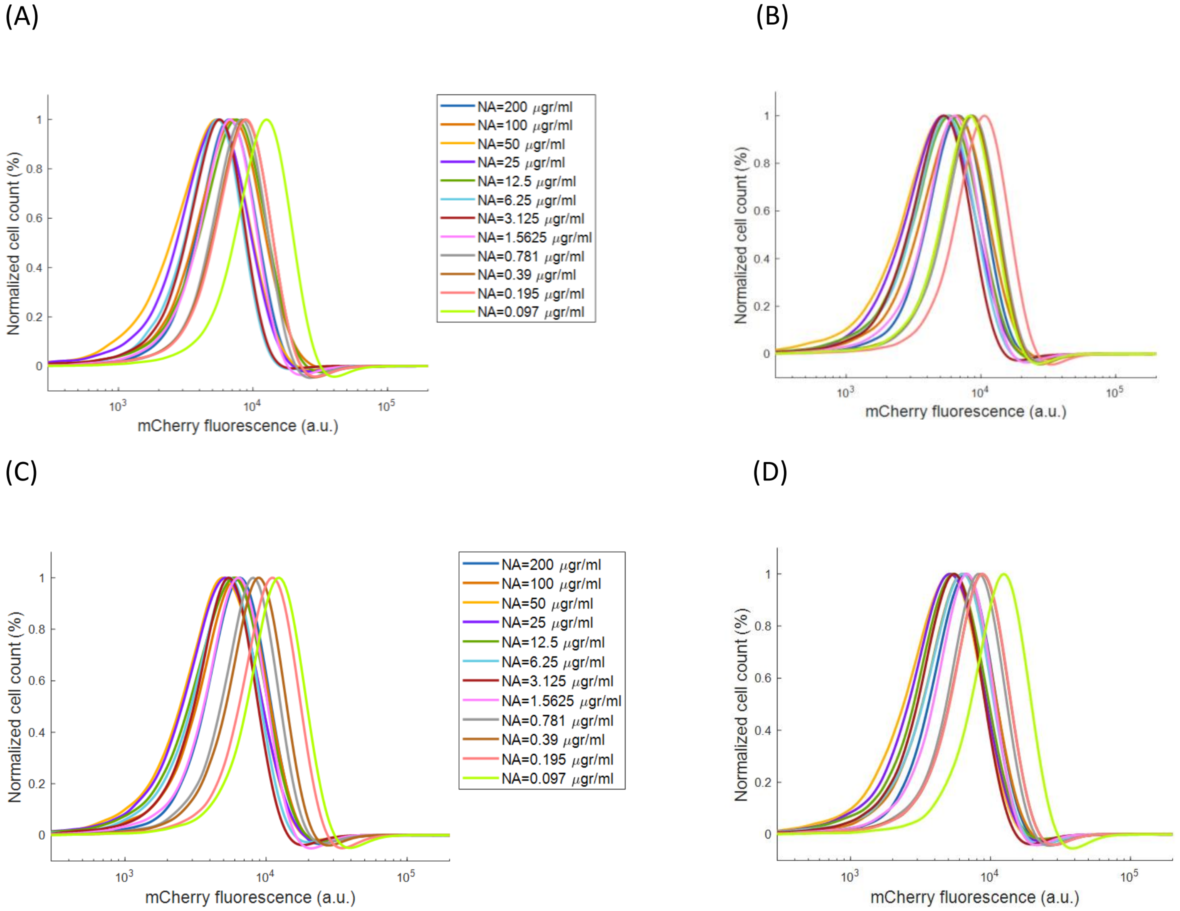

Fig. S63. Flow cytometry data for mCherry over bacterial cells population containing NA-aTc inverting switch design of P_{recA}. (A)[aTc]=33.33ngr/ml, (B)[aTc]=11.11ngr/ml, (C) [aTc]=1.23ngr/ml, (D) [aTc]=0.411ngr/ml. This experimental data was used for generating Fig.S23D.

5. Plasmid maps used in this work.

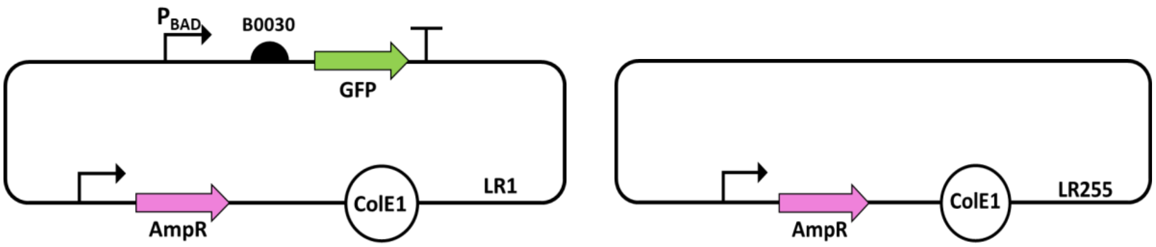

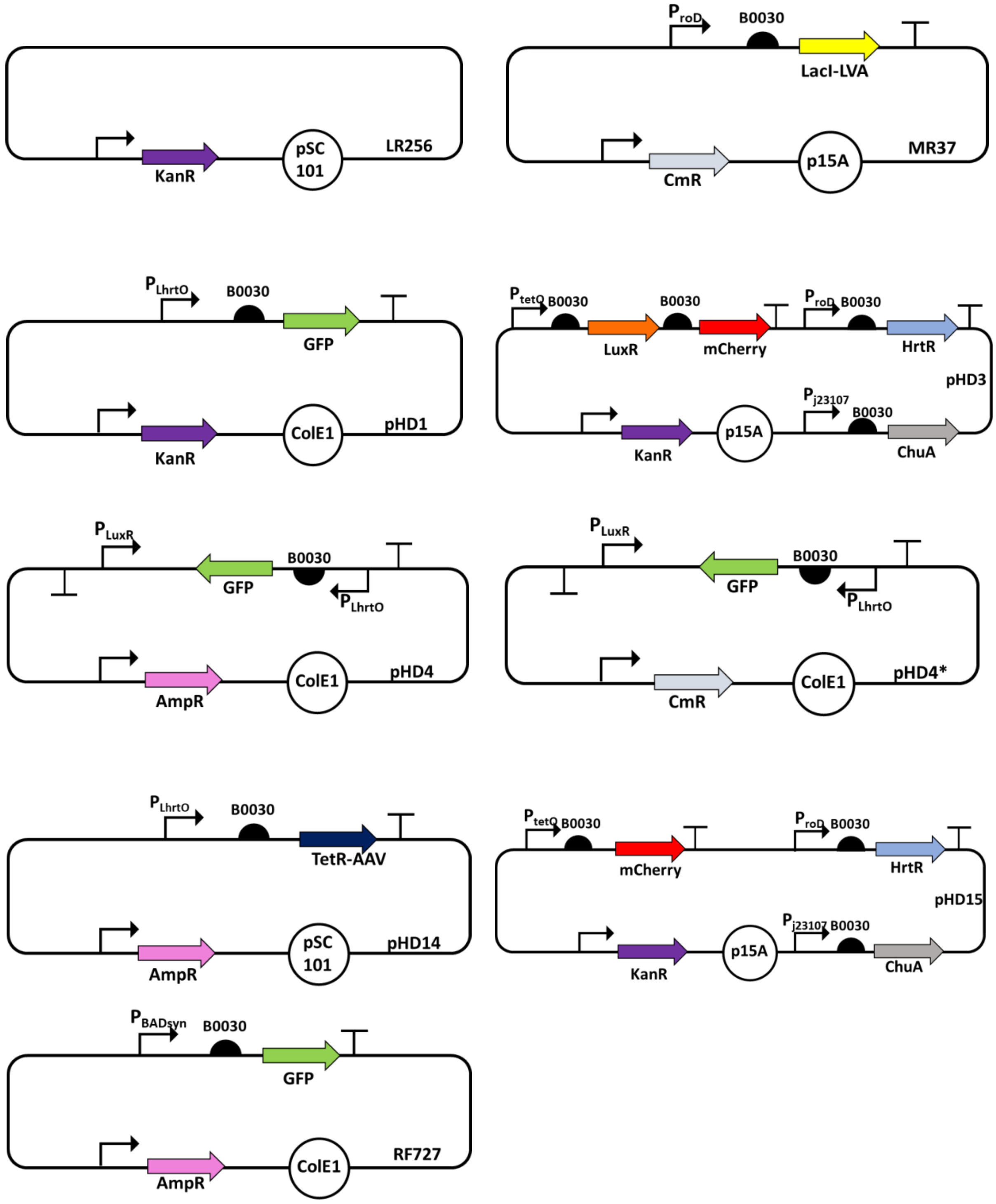
KanR
pSC 101
LR256
P roD
B0030
LacI-LVA
CmR
p15A
MR37
P LhrtO
B0030
GFP
KanR
ColE1
pHD1
P tetO
B0030
LuxR
B0030
mCherry
P roD
B0030
HrtR
pHD3
P j23107
B0030
KanR
p15A
ChuA
P LuxR
GFP
B0030
P LhrtO
AmpR
ColE1
pHD4
P LuxR
GFP
B0030
P LhrtO
CmR
ColE1
pHD4*
P LhrtO
B0030
TetR-AAV
AmpR
pSC 101
pHD14
P tetO
B0030
mCherry
P roD
B0030
HrtR
pHD15
P j23107
B0030
KanR
p15A
ChuA
P BADsyn
B0030
GFP
AmpR
ColE1
RF727

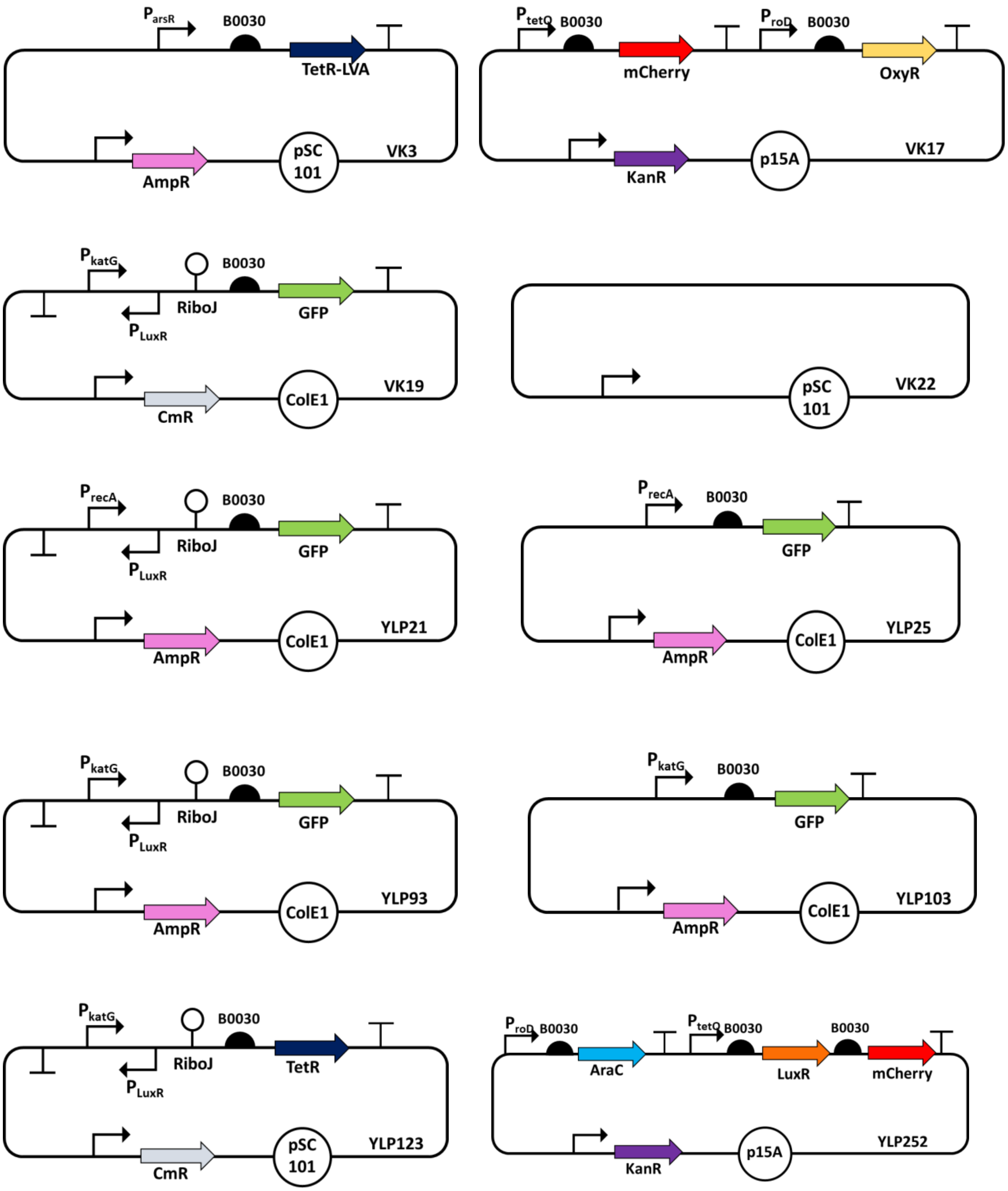
P_{arsR}
B0030
TetR-LVA
AmpR
pSC 101
VK3
P_{tetO}
B0030
mCherry
P_{roD}
B0030
OxyR
KanR
p15A
VK17
P_{katG}
RiboJ
B0030
GFP
P_{LuxR}
CmR
ColE1
VK19
pSC 101
VK22
P_{recA}
RiboJ
B0030
GFP
P_{LuxR}
AmpR
ColE1
YLP21
P_{recA}
B0030
GFP
AmpR
ColE1
YLP25
P_{katG}
RiboJ
B0030
GFP
P_{LuxR}
AmpR
ColE1
YLP93
P_{katG}
B0030
GFP
AmpR
ColE1
YLP103
P_{katG}
RiboJ
B0030
TetR
P_{LuxR}
CmR
pSC 101
YLP123
P_{roD}
B0030
AraC
P_{tetO}
B0030
LuxR
B0030
mCherry
KanR
p15A
YLP252

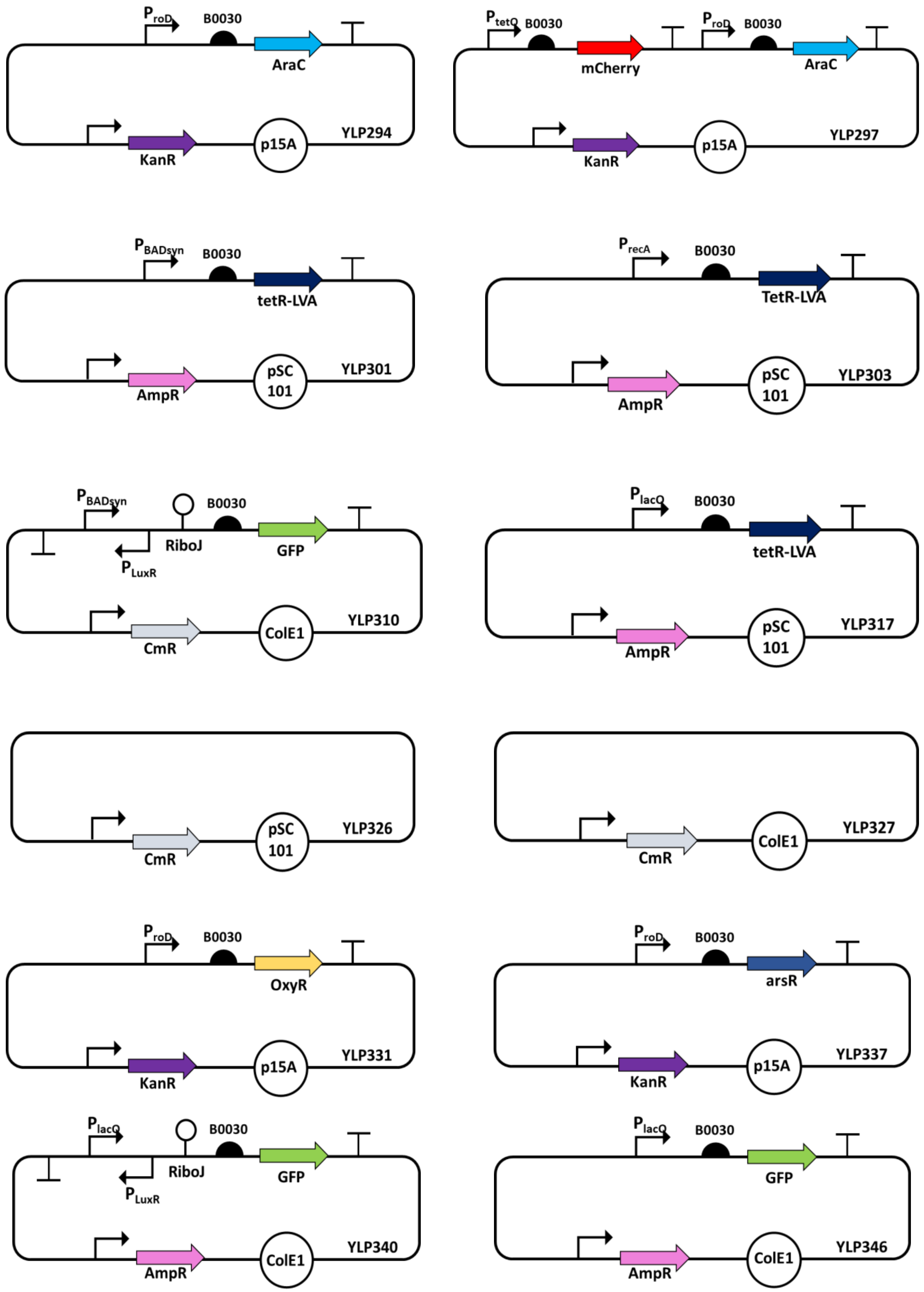
P roD
B0030
AraC
KanR
p15A
YLP294
P tetO
B0030
mCherry
P roD
B0030
AraC
KanR
p15A
YLP297
P BADsyn
B0030
tetR-LVA
AmpR
pSC 101
YLP301
P recA
B0030
TetR-LVA
AmpR
pSC 101
YLP303
P BADsyn
RiboJ
B0030
GFP
P LuxR
CmR
ColE1
YLP310
P lacO
B0030
tetR-LVA
AmpR
pSC 101
YLP317
CmR
pSC 101
YLP326
CmR
ColE1
YLP327
P roD
B0030
OxyR
KanR
p15A
YLP331
P roD
B0030
arsR
KanR
p15A
YLP337
P lacO
RiboJ
B0030
GFP
P LuxR
AmpR
ColE1
YLP340
P lacO
B0030
GFP
AmpR
ColE1
YLP346

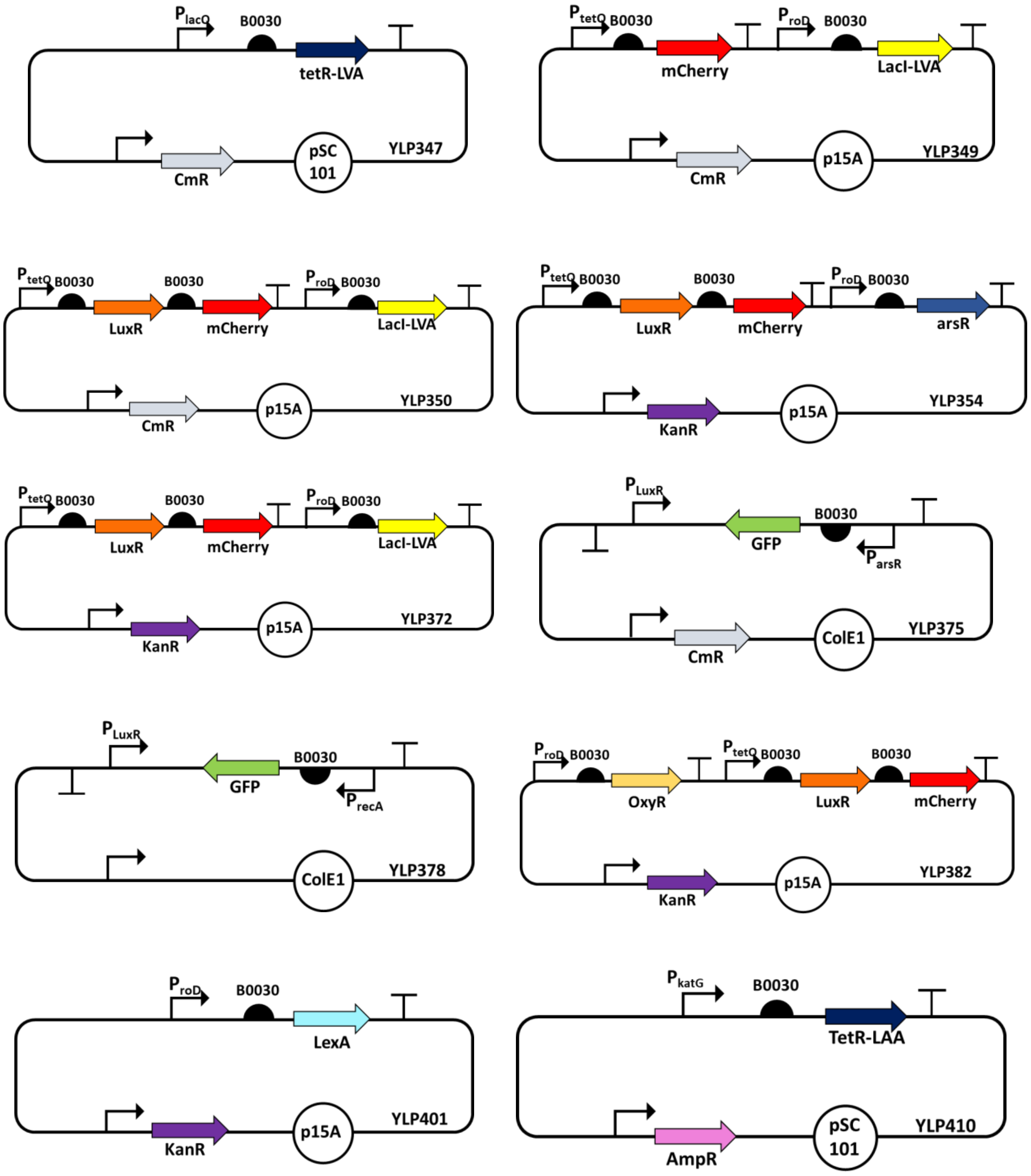
PlacO
B0030
tetR-LVA
CmR
pSC 101
YLP347
PtetO B0030
mCherry
ProD B0030
LacI-LVA
CmR
p15A
YLP349
PtetO B0030
B0030
LuxR
mCherry
ProD B0030
LacI-LVA
CmR
p15A
YLP350
PtetO B0030
B0030
LuxR
mCherry
ProD B0030
arsR
KanR
p15A
YLP354
PtetO B0030
B0030
LuxR
mCherry
ProD B0030
LacI-LVA
KanR
p15A
YLP372
PLuxR
GFP
B0030
ParsR
CmR
ColE1
YLP375
PLuxR
GFP
B0030
PrecA
ColE1
YLP378
ProD B0030
OxyR
PtetO B0030
LuxR
B0030
mCherry
KanR
p15A
YLP382
ProD
B0030
LexA
KanR
p15A
YLP401
PkatG
B0030
TetR-LAA
AmpR
pSC 101
YLP410

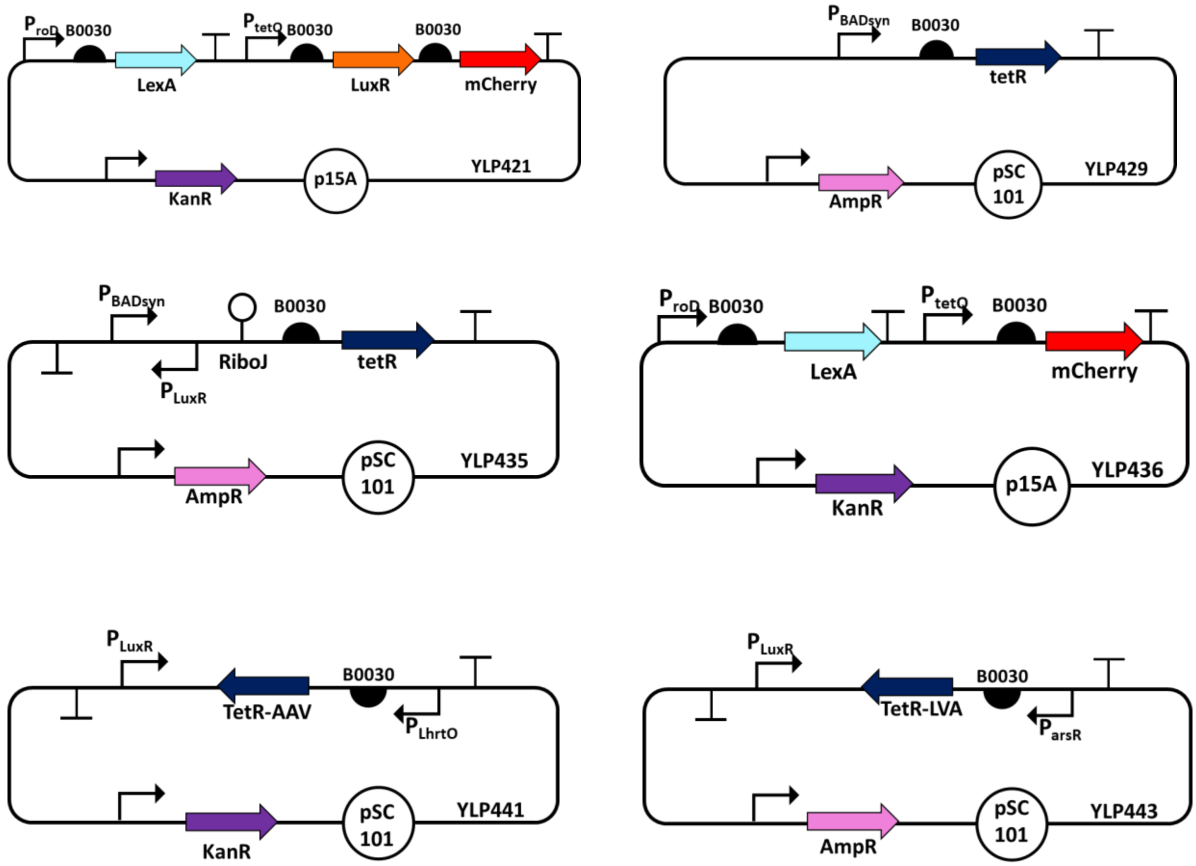

Promoter under test (PUT)	Circuit topology	Plasmids involved in circuit construction	Figures
P_{BAD}	wt	LR1+YLP294	Fig.4C
P_{BADsyn}	wt	RF727+YLP294	Fig.4C
	OL	YLP252+YLP310+VK22	Fig.6A, Fig.S17A
	ICF-TetR	YLP252+YLP310+YLP429	Fig.S17E
	ICF-TetR-LVA	YLP252+YLP310+YLP301	Fig.6B, Fig.S17D
	DNF-TetR	YLP252+YLP310+YLP435	Fig.S17F
	Arabinose-aTc inverting switch (TetR-LVA)	YLP297+YLP301	Fig.S17B
	Arabinose-aTc inverting switch (TetR)	YLP297+YLP429	Fig.S17C
P_{lacO}	OL	YLP340+YLP350+LR256	Fig.S19A
	ICF-TetR-LVA	YLP340+YLP372+YLP347	Fig.S19C
	IPTG-aTc inverting switch	YLP317+YLP349	Fig.S19B

P_{LhrtO}	wt	pHD1+pMM549	Fig.7A, Fig.S20A
	OL	pHD3+pHD4	Fig.7B, Fig.S20B
	ICF-TetR-AAV	pHD3+pHD14+pHD4*	Fig.7C, Fig.S20D
	DNF-TetR-AAV	pHD3+YLP441+pHD4*	Fig.S20E
	Heme-aTc inverting switch for ICF	pHD15+pHD14	Fig.S20C
P_{arsR}	wt	YLP322+YLP337+YLP326	Fig.S21A
	OL	VK9+YLP354+YLP326	Fig.7E, Fig.S21B
	ICF-TetR-LVA	YLP375+YLP354+VK3	Fig.7F, Fig.S21D
	DNF-TetR-LVA	YLP375+YLP354+YLP443	Fig.S21E
	$AsNaO_2$-aTc inverting switch for ICF	VK3+YLP354+YLP327	Fig.S21C
P_{katG}	wt	YLP103+YLP331+YLP326	Fig.S22A
	OL	YLP93+YLP382+YLP326	Fig.8A, Fig.S22B
	ICF	VK19+YLP382+YLP410	Fig.8C, Fig.S22D
	DNF	YLP93+YLP382+YLP123	Fig.S22E
	H_2O_2-aTc inverting switch for DNF	YLP123+VK17+LR255	Fig.8B, Fig.S22C
P_{recA}	wt	YLP25+YLP401+VK22	Fig.S23A
	AT-OL	YLP378+YLP421+VK22	Fig.S23B
	TI-OL	YLP21+YLP421+YLP326	Fig.8E, Fig.S23C
	ICF	YLP303+YLP421+YLP378	Fig.8F, Fig.S23E
	NA-aTc inverting switch	YLP303+YLP436	Fig.S23D

Table S1. List of plasmids used for synthetic genetic circuits in this work.

6. Synthetic parts used in this work

Part	Type	DNA sequence	Source
P_{lacO}	promoter	aattgtgagcggataacaattgacattgtgagcggataacaagatactgagcacatcagcaggacgcactgacc	(Lutz & Bujard, 1997)
P_{tetO}	promoter	tccctatcagtgatagagattgacatccctatcagtgatagagatactgagcacatcagcaggacgcactgacc	(*Part: BBa_R0040*, 2013)
P_{lux}	promoter	acctgtaggatcgtacaggtttacgcaagaaaatggtttgttatagtcgaataaa	(*Part:BBa R0062 - Parts.Igem. Org*, 2013)
P_{LhrtO}	promoter	ataaatgacacagtgtcatttgacaaaatgacacagtgtcatgatactgagcaca	(Mimee et al., 2018)
P_{roD}	Constitutive promoter	cacagctaacaccacgtcgtccctatctgctgccctaggtctatgagtggttgctggataactttacgggcatgcataaggctcgtataatatattcagggagaccacaacggtttccctctacaaataattttgtttaacttt	(Davis et al., 2011)
P_{j23107}	Constitutive promoter	tttacggctagctcagccctaggtattatgctagc	(Mimee et al., 2018)
P_{BAD}	Inducible promoter	aagaaaccaattgtccatattgcatcagacattgccgtcactgcgtcttttactggctcttctcgctaaccaaaccggtaaccccgcttattaaaagcattctgtaacaaagcgggaccaaagccatgacaaaaacgcgtaacaaaagtgtctataatcacggcagaaaagtccacattgattatttgcacggcgtcacactttgctatgccatagcatttttatccataagattagcggatcctacctgacgctttttatcgcaactctctactgtttctccat	(Lee et al., 1981)

P_{BADsyn}	Synthetic P_{BAD} promoter	atagcatttttatccataagattagcggatcctacctgacgctttttatcgcaactctctactgtttctccataccgtttttttgggctagc	This work
P_{katG}	H2O2-inducible promoter	cgaaatgagggcgggaaaataaggttatcagccttgttttctccctcattacttgaaggatatgaagctaaaacccttttttataaagcatttgtccgaattcggacataatcaaaaaagcttaattaagatcaatttgatctacatctctttaaccaacaatatgtaagatctcaactatcgcatccgtggattaattcaattataacttctctctaacgctgtgtatcgtaacggtaacactgta	Modified (Belkin et al., 1996)
P_{recA}	NA-inducible promoter	agagaagcctgtcggcaccgtctggtttgcttttgccactgcccgcggtgaaggcattacccggcgggatgcttcagcggcgaccgtgatgcggtgcgtcgtcaggctactgcgtatgcattgcagaccttgtggcaacaatttctacaaaacacttgatactgtatgagcatacagtataattgcttcaacagaacatattgactatccggtattacccggcatgac	Modified (Vollmer et al., 1997)
P_{arsR}	$AsNaO_2$-inducible promoter	ccaactcaaaattcacacctattaccttcctctgcacttacacattcgttaagtcatatatgtttttgacttatccgcttcgaagagagacactacctgcaa	(*Part:BBa_J33201 Parts.Igem.Org*, 2013)
GFP	CDS	atgcgtaaaggagaagaacttttcactggagttgtcccaattcttgttgaattagatggtgatgttaatgggcacaaattttctgtcagtggagagggtgaaggtgatgcaacatacggaaaacttacccttaaatttatttgcactactggaaaactacctgttccatggccaacacttgtcactactttcggttatggtgttcaatgctttgcgagatacccagatcatatgaaacagcatgactttttcaagagtgccatgcccgaaggttatgtacaggaaagaactatatttttcaaagatgacgggaactacaagacacgtgctgaagtcaagtttgaaggtgatacccttgttaatagaatcgagttaaaaggtattgattttaaagaagatggaaacattcttggacacaaattggaatacaactataactcacacaatgtatacatcatggcagacaaacaaaagaatggaatcaaagttaacttcaaaattagacacaacattgaagatggaagcgttcaactagcagaccattatcaacaaaatactccaattggcgatggccctgtccttttaccagacaaccattacctgtccacacaatctgccctttcgaaagatcccaacgaaaagagagaccacatggtccttcttgagtttgtaacagctgctgggattacacatggcatggatgaactatacaaataa	(Bongaerts et al., 2002)
mCherry	CDS	atggtgagcaagggcgaagaagataacatggccatcatcaaggagttcatgcgcttcaaggtgcacatggagggctccgtgaacggccacgagttcgagatcgagggcgagggcgagggccgcccctacgagggcacccagaccgccaagctgaaggtgaccaagggtggccccctgcccttcgcctgggacatcctgtcccctcagttcatgtacggctccaaggcctacgtgaagcaccccgccgacatccccgactacttgaagctgtccttccccgagggcttcaagtgggagcgcgtgatgaacttcgaggacggcggcgtggtgaccgtgacccaggactcctccctgcaggacggcgagttcatctacaaggtgaagctgcgcggcaccaacttcccctccgacggccccgtaatgcagaagaagaccatgggctgggaggcctcctccgagcggatgtaccccgaggacggcgccctgaagggcgagatcaagcagaggctgaagctgaaggacggcggccactacgacgctgaggtcaagaccacctacaaggccaagaagcccgtgcagctgcccggcgcctacaacgtcaacatcaagttggacatcacctcccacaacgaggactacaccatcgtggaacagtacgaacgcgccgagggccgccactccaccggcggcatggacgagctgtacaagtaa	(Shaner et al., 2004)
araC	CDS	atggctgaagcgcaaaatgatcccctgctgccgggatactcgtttaacgcccatctggtggcgggtttaacgccgattgaggccaacggttatctcgatttttttatcgaccgaccgctgggaatgaaaggttatattctcaatctcaccattcgcggtcagggggtggtgaaaaatcagggacgagaatttgtctgccgaccgggtgatattttgctgttcccgccaggagagattcatcactacggtcgtcatccggaggctcgcgaatggtatcaccagtgggtttactttcgtccgcgcgcctactggcatgaatggcttaactggccgtcaatatttgccaatacgggtttctttcgcccggatgaagcgcaccagccgcatttcagcgacctgtttgggcaaatcattaacgccgggcaaggggaagggcgctattcggagctgctggcgataaatctgcttgagcaattgttactgcggcgcatggaagcgattaacgagtcgctccatccaccgatggataatcgggtacgcgaggcttgtcagtacatcagcgatcacctggcagacagcaattttgatatcgccagcgtcgcacagcatgtttgcttgtcgccgtcgcgtctgt	(Lee et al., 1981)

		cacatcttttccgccagcagttagggattagcgtcttaagctggcgcgaggaccaacgcattagtcaggcgaagctgcttttgagcactacccggatgcctatcgccaccgtcggtcgcaatgttggttttgacgatcaactctatttctcgcgagtatttaaaaaatgcaccggggccagcccgagcgagtttcgtgccggttgtgaagaaaaagtgaatgatgtagccgtcaagttgtcataa	
LuxR	CDS	atgaaaaacataaatgccgacgacacatacagaataattaataaaattaaagcttgtagaagcaataatgatattaatcaatgcttatctgatatgactaaaatggtacattgtgaatattatttactcgcgatcatttatcctcattctatggttaaatctgatatttcaatcctagataattaccctaaaaaatggaggcaatattatgatgacgctaatttaataaaatatgatcctatagtagattattctaactccaatcattcaccaattaattggaatatatttgaaaacaatgctgtaaataaaaaatctccaaatgtaattaaagaagcgaaaacatcaggtcttatcactgggtttagtttccctattcatacggctaacaatggcttcggaatgcttagttttgcacattcagaaaaagacaactatatagatagtttatttttacatgcgtgtatgaacataccattaattgttccttctctagttgataattatcgaaaaataaatatagcaaataataaatcaaacaacgatttaaccaaaagagaaaaagaatgtttagcgtgggcatgcgaaggaaaaagctcttgggatatttcaaaaatattaggttgcagtgagcgtactgtcactttccatttaaccaatgcgcaaatgaaactcaatacaacaaaccgctgccaaagtatttctaaagcaattttaacaggagcaattgattgcccatactttaaaaattaa	(*Part: BBa_C0062 Http://Parts.Igem.Org/*, 2013)
LacI-LVA	CDS	gtgaaaccagtaacgttatacgatgtcgcagagtatgccggtgtctcttatcagaccgtttcccgcgtggtgaaccaggccagccacgtttctgcgaaaacgcgggaaaaagtggaagcggcgatggcggagctgaattacattcccaaccgcgtggcacaacaactggcgggcaaacagtcgttgctgattggcgttgccacctccagtctggccctgcacgcgccgtcgcaaattgtcgcggcgattaaatctcgcgccgatcaactgggtgccagcgtggtggtgtcgatggtagaacgaagcggcgtcgaagcctgtaaagcggcggtgcacaatcttctcgcgcaacgcgtcagtgggctgatcattaactatccgctggatgaccaggatgccattgctgtggaagctgcctgcactaatgttccggcgttatttcttgatgtctctgaccagacacccatcaacagtattattttctcccatgaagacggtacgcgactgggcgtggagcatctggtcgcattgggtcaccagcaaatcgcgctgttagcgggcccattaagttctgtctcggcgcgtctgcgtctggctggctggcataaatatctcactcgcaatcaaattcagccgatagcggaacgggaaggcgactggagtgccatgtccggttttcaacaaaccatgcaaatgctgaatgagggcatcgttcccactgcgatgctggttgccaacgatcagatggcgctgggcgcaatgcgcgccattaccgagtccgggctgcgcgttggtgcggatatctcggtagtgggatacgacgataccgaagacagctcatgttatatcccgccgttaaccaccatcaaacaggattttcgcctgctggggcaaaccagcgtggaccgcttgctgcaactctctcagggccaggcggtgaagggcaatcagctgttgcccgtctcactggtgaaaagaaaaaccaccctggcgcccaatacgcaaaccgcctctccccgcgcgttggccgattcattaatgcagctggcacgacaggtttcccgactggaaagcgggcagaggcctgctgcaaacgacgaaaactacgctttagtagcttaa	(Lutz & Bujard, 1997)
hrtR	CDS	atgccaaaatcaacctattttagtctttctgacgaaaaacgaaaacgtgtctatgatgcctgtttactagaatttcaaacgcactctttccatgaagctaaaatcatgcacatcgtaaaagcacttgatatcccaagaggaagtttttatcaatactttgaagatttgaaggattcatactattatatcttgtcacaggaaactgtcgagattcatgatttattttttaatttactaaaagaatatcctctagaagttgctcttaataaatacaagtatcttcttcttgaaaatttagtaaattcgccccaatataatctttataaatatcgattttttagattggacttatgaattagaaagagattggaagcctaaaggcgaggtaactgttcccgctcgtgaacttgataatcctatttcccaagtattaaaatcagtcattcacaatctagtttatcgcatgtttagtgaaaattgggatgaacaaaagtttattgaaacttacgataaagaaatcaaattgctcacagagggcttgcttaattatgttactgaaagcaaaaaatag	(Mimee et al., 2018)
ChuA	CDS	atgtcacgtccgcaatttacctcgttgcgtttgagtttattggccttagctgtttctgccaccttgccaacgtttgcttttgctactgaaaccatgaccgttacggcaacggggaatgcccgtagttccttcgaagcgcctatgatggtcagcgtcatcgacacttccgctcctgaaaatcaaacggctacttcagccaccgatctgctgcgtcatgttcctggaattactctggatggtaccggacgaaccaacggtcaggatgtaaatatgcgtggctatgatcatcgcggcgtgctggttcttgtcgatggtgttcgtcagggaacggataccggacacctgaatggcacttttctcgatccggcgctgatcaagcgtgttgagattgttcgtggaccttcagcattactgtatggcagtggcgcgctgggtggagtgatctcctac	(Mimee et al., 2018)

		gatacggtcgatgcaaaagatttattgcaggaaggacaaagcagtggttttcgtgtctttggta<br>ctggcggcacgggggaccatagcctgggattaggcgcgagcgcgtttgggcgaactgaaa<br>atctggatggtattgtggcctggtccagtcgcgatcggggtgatttacgccagagcaatggtg<br>aaaccgcgccgaatgacgagtccattaataacatgctggcgaaagggacctggcaaattgat<br>tcagcccagtctctgagcggtttagtgcgttactacaacaacgacgcgcgtgaaccaaaaaat<br>ccgcagaccgttggggcttctgaaagcagcaacccgatggttgatcgttcaacaattcaacgc<br>gatgcgcagctttcttataaactcgccccgcagggcaacgactggttaaatgcagatgcaaaa<br>atttattggtcggaagtccgtattaatgcgcaaaacacggggagttccggcgagtatcgcgaa<br>cagataacaaaaggagccaggctggagaaccgttccactctctttgccgacagtttcgcttctc<br>acttactgacatatggcggtgagtattatcgtcaggaacaacatccgggcggcgcgacgacg<br>ggcttcccgcaagcaaaaatcgattttagctccggctggctacaggatgagatcaccttacgc<br>gatctgccgattaccctgcttggcggaacccgctatgacagttatcgcggtagcagtgacggt<br>tacaaagacgttgatgccgacaaatggtcatctcgtgcggggatgactatcaatccgactaac<br>tggctgatgttatttggctcatatgcccaggcattccgcgccccgacgatgggcgaaatgtata<br>acgattctaagcacttctcgattggtcgcttctataccaactattgggtgccaaacccgaactta<br>cgtccggaaactaacgaaactcaggagtacggttttgggctgcgttttgatgacctgatgttgt<br>ccaatgatgctctggaatttaaagccagctactttgataccaaagcgaaggattacatctccac<br>gaccgtcgatttcgcggcggcgacgactatgtcgtataacgtcccgaacgccaaaatctggg<br>gctgggatgtgatgacgaaatataccactgatctgtttagccttgatgtggcctataaccgtacc<br>cgcggcaaagacaccgataccggcgaatacatctccagcattaacccggatactgttaccag<br>cactctgaatattccgatcgctcacagtggcttctctgttgggtgggttggtacgtttgccgatcg<br>ctcaacacatatcagcagcagttacagcaaacaaccaggctatggcgtgaatgatttctacgt<br>cagttatcaaggacaacaggcgctcaaaggtatgaccactactttggtgttgggtaacgctttc<br>gacaaagagtactggtcgccgcaaggcatcccacaggatggtcgtaacggaaaaattttcgt<br>gagttatcaatggtaa	
riboj	RNA	gctgtcaccggatgtgctttccggtctgatgagtccgtgaggacgaaacagc	(Khvorova et al., 2003)
OxyR	CDS	atgaatattcgtgatcttgagtacctggtggcattggctgaacaccgccattttcggcgtgcgg<br>cagattcctgccacgttagccagccgacgcttagcgggcaaattcgtaagctggaagatgag<br>ctgggcgtgatgttgctggagcggaccagccgtaaagtgttgttcacccaggcgggaatgct<br>gctggtggatcaggcgcgtaccgtgctgcgtgaggtgaaagtccttaaagagatggcaagc<br>cagcagggcgagacgatgtccggaccgctgcacattggtttgattcccacagttggaccgta<br>cctgctaccgcatattatccctatgctgcaccagacctttccaaagctggaaatgtatctgcatg<br>aagcacagacccaccagttactggcgcaactggacagcggcaaactcgattgcgtgatcctc<br>gcgctggtgaaagagagcgaagcattcattgaagtgccgttgtttgatgagccaatgttgctg<br>gctatctatgaagatcacccgtgggcgaaccgcgaatgcgtaccgatggccgatctggcag<br>gggaaaaactgctgatgctggaagatggtcactgtttgcgcgatcaggcaatgggtttctgtttt<br>gaagccggggcggatgaagatacacacttccgcgcgaccagcctggaaactctgcgcaac<br>atggtggcggcaggtagcgggatcactttactgccagcgctggctgtgccgccggagcgca<br>aacgcgatggggttgtttatctgccgtgcattaagccggaaccacgccgcactattggcctgg<br>tttatcgtcctggctcaccgctgcgcagccgctatgagcagctggcagaggccatccgcgca<br>agaatggatggccatttcgataaagttttaaaacaggcggtttaa	(Aslund et al., 2002)
arsR	CDS	atgtcatttctgttacccatccaattgttcaaaattcttgctgatgaaacccgtctgggcatcgtttt<br>actgctcagcgaactgggagagttatgcgtctgcgatctctgcactgctctcgaccagtcgca<br>gcccaagatctcccgccacctggcattgctgcgtgaaagcgggctattgctggaccgcaagc<br>aaggtaagtgggttcattaccgcttatcaccgcatattccagcatgggcggcgaaaattattga<br>tgaggcctggcgatgtgaacaggaaaaggttcaggcgattgtccgcaacctggctcgacaa<br>aactgttccggggacagtaagaacatttgcagttaa	(*Part: BBa_J33201 Parts.Igem. Org*, 2013)
LexA	CDS	atgaaagcgttaacggccaggcaacaagaggtgtttgatctcatccgtgatcacatcagccag<br>acaggtatgccgccgacgcgtgcggaaatcgcgcagcgtttggggttccgttccccaaacg	(Little et al., 1981)

		cggctgaagaacatctgaaggcgctggcacgcaaaggcgttattgaaattgtttccggcgcatcacgcgggattcgtctgttgcaggaagaggaagaagggttgccgctggtaggtcgtgtggctgccggtgaaccacttctggcgcaacagcatattgaaggtcattatcaggtcgatccttccttattcaagccgaatgctgatttcctgctgcgcgtcagcgggatgtcgatgaaagatatcggcattatggatggtgacttgctggcagtgcataaaactcaggatgtacgtaacggtcaggtcgttgtcgcacgtattgatgacgaagttaccgttaagcgcctgaaaaaacagggcaataaagtcgaactgttgccagaaaatagcgagtttaaaccaattgtcgttgaccttcgtcagcagagcttcaccattgaagggctggcggttggggttattcgcaacggcgactggctgtaa	
TetR	CDS	atgtccagattagataaaagtaaagtgattaacagcgcattagagctgcttaatgaggtcggaatcgaaggtttaacaacccgtaaactcgcccagaagctaggtgtagagcagcctacattgtattggcatgtaaaaaataagcgggctttgctcgacgccttagccattgagatgttagataggcaccatactcacttttgccctttagaaggggaaagctggcaagattttttacgtaataacgctaaaagttttagatgtgctttactaagtcatcgcgatggagcaaaagtacatttaggtacacggcctacagaaaaacagtatgaaactctcgaaaatcaattagcctttttatgccaacaaggtttttcactagagaatgcattatatgcactcagcgctgtggggcattttactttaggttgcgtattggaagatcaagagcatcaagtcgctaaagaagaaagggaaacacctactactgatagtatgccgccattattacgacaagctatcgaattatttgatcaccaaggtgcagagccagccttcttattcggccttgaattgatcatatgcggattagaaaaacaacttaaatgtgaaagtgggtcctaa	(*Part: BBa_C0040 Parts.Igem. Org*, 2013)
TetR-AAV	CDS	atgtccagattagataaaagtaaagtgattaacagcgcattagagctgcttaatgaggtcggaatcgaaggtttaacaacccgtaaactcgcccagaagctaggtgtagagcagcctacattgtattggcatgtaaaaaataagcgggctttgctcgacgccttagccattgagatgttagataggcaccatactcacttttgccctttagaaggggaaagctggcaagattttttacgtaataacgctaaaagttttagatgtgctttactaagtcatcgcgatggagcaaaagtacatttaggtacacggcctacagaaaaacagtatgaaactctcgaaaatcaattagcctttttatgccaacaaggtttttcactagagaatgcattatatgcactcagcgctgtggggcattttactttaggttgcgtattggaagatcaagagcatcaagtcgctaaagaagaaagggaaacacctactactgatagtatgccgccattattacgacaagctatcgaattatttgatcaccaaggtgcagagccagccttcttattcggccttgaattgatcatatgcggattagaaaaacaacttaaatgtgaaagtgggtccaggcctgctgcaaacgacgaaaactacgctgcagcagtttaa	(*Part: BBa_C0040 Parts.Igem. Org*, 2013)
TetR-LVA		atgtccagattagataaaagtaaagtgattaacagcgcattagagctgcttaatgaggtcggaatcgaaggtttaacaacccgtaaactcgcccagaagctaggtgtagagcagcctacattgtattggcatgtaaaaaataagcgggctttgctcgacgccttagccattgagatgttagataggcaccatactcacttttgccctttagaaggggaaagctggcaagattttttacgtaataacgctaaaagttttagatgtgctttactaagtcatcgcgatggagcaaaagtacatttaggtacacggcctacagaaaaacagtatgaaactctcgaaaatcaattagcctttttatgccaacaaggtttttcactagagaatgcattatatgcactcagcgctgtggggcattttactttaggttgcgtattggaagatcaagagcatcaagtcgctaaagaagaaagggaaacacctactactgatagtatgccgccattattacgacaagctatcgaattatttgatcaccaaggtgcagagccagccttcttattcggccttgaattgatcatatgcggattagaaaaacaacttaaatgtgaaagtgggtccaggcctgctgcaaacgacgaaaactacgctttagtagcttaa	(*Part: BBa_C0040 Parts.Igem. Org*, 2013)S20

Table S2. List of the synthetic parts, their types and sequences.

Primer	Description	Sequence
AatII-recA-f	P_{recA} promoter, amplified from MG6515 genome	ttaagacgtcagagaagcctgtcggcac

EcoRI-recA-r	P_{recA} promoter	tagtatgaattcgtcatgccgggtaataccg
AatII-P_{katG}-f	P_{katG} promoter	tagacgtccgaaatgagggcgggaaa
KpnI-PkatG-B0030-r	P_{katG} promoter	taatatggtacctttctcctctttaatacagtgttaccgttacgatacac
Aat-P_{arsR}-f	P_{arsR} promoter	taactagacgtcccaactcaaaattcacacctattac
EcoRI-P_{arsR}-r	P_{arsR} promoter	taagttgaattcttgcaggtagtgtctctcttcg
KpnI-LexA-f	*LexA* gene	tacaaatagtaggtaccatgaaagcgttaacggccaggc
BamHI-LexA-r	*LexA* gene	tacaatggatccttaCAGCCAGTCGCCGTTGC
KpnI-arsR-f	*arsR* gene	taatcaggtaccatgtcatttctgttacccatccaattg
BamHI-arsR-r	*arsR* gene	tactatggatccttaactgcaaatgttcttactgtccc
KpnI-OxyR-f	*OxyR* gene	atgcggtaccatgaatattcgtgatcttgagtacc
BamHI-XmaI-OxyR-r	*OxyR* gene	gcatggatcccccgggttaaaccgcctgttttaaaactttatc

Table S3. Primers used for part amplification in this work.

Symbol	Description
OL	Open loop
ICF	Indirect coherent feedforward
DNF	Double negative feedback
FCA	Fold change activation
PUT	Part under test
In	Inducer level
Out	Output level e.g., GFP, mCherry
β	Basal level of promoter
S	Sensitivity of part under test
S_f	Sensitivity of the function *f(In)*
$f(In)$	Non-linear monotonic function that describes the circuit behavior
F_S	Strength of the feedforward/feedback loops
x	Concentration of inducer
x_0	Initial concentration of inducer
MDL	Minimum detection level
θ	Probability of binding of RNAPs to forward and reverse promoters simultaneously
K_{df}	Dissociation constant of transcription factors X binding to forward promoter
K_{dr}	Dissociation constant of transcription factors Y binding to reverse promoter
K_{dz}	Dissociation constant of transcription factors Z binding to its promoter
K_R	Dissociation constant of repressor R binding to promoter P_{forw_R}
K_m	Dissociation constant of the binding reaction between mRNA and antisense RNA
X	Concentration of transcription factors which bind to forward promoter
Y	Concentration of transcription factors which bind to reverse promoter
Y_{max}	Maximum concentration of transcription factors Y which bind to reverse promoter P_{Rev}
Z	Expression level of output
Z_{max}	Maximum protein level
Z_{min}	Minimum protein level
n_f	Hill-coefficient of transcription factors X binding to forward promoter
n_r	Hill-coefficient of transcription factors Y binding to reverse promoter
n_z	Hill-coefficient of transcription factors Z binding to their promoter
n_R	Hill-coefficient of repressor R binding to promoter P_{forw_R}
P	Probability that RNA polymerase is bound to the forward promoter at the equilibrium

ρ_1	Shift coefficient in switching threshold
ρ_2	Repression coefficient in the expression level
α	Translation rate of Z through transcriptional interference
α_Z	Translation rate of Z through antisense transcription
$mRNA$	Level of mRNA transcribed from forward promoter
$mRNA_T$	Total level of mRNA transcribed from forward promoter
M_f	Maximum mRNA level
RNA_{Anti}	Level of antisense RNA transcribed from reverse promoter located downstream to Z
RNA_{Anti_T}	Total level of antisense RNA
M_r	Maximum RNA_{Anti} level
τ_Z	Protein Z half-life
β_f	Basal level of forward promoter (w/o the reverse promoter)
β_r	Basal level of forward promoter (w/ the reverse promoter)
R	Concentration of repressor R which binds to promoter P_{forw_R}
R_{max}	Maximum concentration of repressor R
P_{forw_R}	Promoter that drives the expression of gene Y and is repressed by repressor R
P_{forw}	Forward promoter (sense) that drives the transcription of the *out*
P_{Rev}	Reverse promoter (antisense) that interferes with the transcription of the *out*
TF	Transcription factor
AHL	N-(β-Ketocaproyl)-L-homoserine lactone 3OC6HSL
IPTG	Isopropyl-β-D-1-thiogalactopyranoside
aTc	Anhydrotetracyclin
P_{lux}	Promoter regulated by LuxR, sensitive for AHL
P_{BAD}	Promoter regulated by AraC and sensitive for arabinose
P_{BADsyn}	Synthetic AraC promoter, regulated by AraC and sensitive for arabinose
P_{lacO}	Promoter regulated by LacI and sensitive for IPTG
P_{LhrtO}	Promoter regulated by HrtR and sensitive for heme group
P_{arsR}	Promoter regulated by ArsR and sensitive for arsenic
P_{katG}	Promoter for a *katG* gene, regulated by OxyR and sensitive for H_2O_2
P_{recA}	Promoter for a *recA* gene, regulated by LexA and sensitive for Nalidixic acid (NA)

Table S4. List of parameters and abbreviations used in this work.

References

Ackers, G. K., Johnsontt, A. D., & Shea, M. A. (1982). Quantitative model for gene regulation by A phage repressor. *Proc. NatL Acad. Sci. USA*, *79*, 1129–1133.

Aslund, F., Zheng, M., Beckwith, J., & Storz, G. (2002). Regulation of the OxyR transcription factor by hydrogen peroxide and the cellular thiol--disulfide status. *Proceedings of the National Academy of Sciences*, *96*(11), 6161–6165.

Barger, N., Litovco, P., Li, X., Habib, M., & Daniel, R. (2019). Synthetic metabolic computation in a bioluminescence-sensing system. *Nucleic Acids Research*, *47*(19), 10464–10474.

Belkin, S., Smulski, D. R., Vollmer, A. C., Van Dyk, T. K., & Larossa, R. A. (1996). Oxidative stress

detection with Escherichia coli harboring a katG'::lux fusion. *Applied and Environmental Microbiology*, *62*(7), 2252–2256.

Bintu, L., Buchler, N. E., Garcia, H. G., Gerland, U., Hwa, T., Kondev, J., & Phillips, R. (2005). Transcriptional regulation by the numbers: models. *Curr Opin Genet Dev*, *15*(2), 116–124.

Bongaerts, R. J. M., Hautefort, I., Sidebotham, J. M., & Hinton, J. C. D. (2002). Green fluorescent protein as a marker for conditional gene expression in bacterial cells. *Methods in Enzymology*, *358*(February), 43–66.

Brophy, J. A. N., & Voigt, C. A. (2016). Antisense transcription as a tool to tune gene expression. *Molecular Systems Biology*, 0–14.

Davidov, Y., Rozen, R., Smulski, D. R., Van Dyk, T. K., Vollmer, A. C., Elsemore, D. A., LaRossa, R. A., & Belkin, S. (2000). Improved bacterial SOS promoter::lux fusions for genotoxicity detection. *Genetic Toxicology and Environmental Mutagenesis*, *466*(Mutation Research), 97–107.

Davis, J. H., Rubin, A. J., & Sauer, R. T. (2011). Design, construction and characterization of a set of insulated bacterial promoters. *Nucleic Acids Research*, *39*(3), 1131–1141.

Khvorova, A., Lescoute, A., Westhof, E., & Jayasena, S. D. (2003). Sequence elements outside the hammerhead ribozyme catalytic core enable intracellular activity. *Nature Structural Biology*, *10*(9), 708–712.

Lee, N. L., Gielow, W. O., & Wallace, R. G. (1981). Mechanism of araC autoregulation and the domains of two overlapping promoters, P(c) and P(bad), in the l-arabinose regulatory region of Escherichia coli. *Proceedings of the National Academy of Sciences of the United States of America*, *78*(2 II), 752–756.

Little, J. W., Mount, D. W., & Yanisch-Perron, C. R. (1981). Purified lexA protein is a repressor of the recA and lexA genes. *Proceedings of the National Academy of Sciences of the United States of America*, *78*(7), 4199–4203.

Lutz, R., & Bujard, H. (1997). Independent and tight regulation of transcriptional units in Escherichia coli via the LacR/O, the TetR/O and AraC/I 1-I 2 regulatory elements. *Nucl*, *25:6*, 1203–1210.

Mangan, S., & Alon, U. (2003). Structure and function of the feed-forward loop network motif. *Proceedings of the National Academy of Sciences of the United States of America*, *100*(21), 11980–11985.

Mimee, M., Nadeau, P., Hayward, A., Carim, S., Flanagan, S., Jerger, L., Collins, J., Mcdonnell, S., Swartwout, R., Citorik, R. J., Bulovi, V., Langer, R., Traverso, G., Chandrakasan, A. P., & Lu, T. K. (2018). An ingestible bacterial-electronic system to monitor gastrointestinal health. *Science*, *918*(May), 915–918.

Panek, H. R., & Brian, M. R. O. (2004). *KatG Is the Primary Detoxifier of Hydrogen Peroxide Produced by Aerobic Metabolism in Bradyrhizobium japonicum*. *186*(23), 7874–7880.

Part: BBa_C0040 parts.igem.org. (2013). http://parts.igem.org/Part:BBa_C0040

Part: BBa_C0062 http://parts.igem.org/. (2013). http://parts.igem.org/Part:BBa_C0062

Part: BBa_J33201 parts.igem.org. (2013). http://parts.igem.org/Part:BBa_J33201

Part: BBa_R0040. (2013). http://parts.igem.org/Part:BBa_R0040

Part:BBa R0062 - parts.igem.org. (2013). http://parts.igem.org/Part:BBa_R0062

Shaner, N. C., Campbell, R. E., Steinbach, P. A., Giepmans, B. N. G., Palmer, A. E., & Tsien, R. Y. (2004). Improved monomeric red, orange and yellow fluorescent proteins derived from Discosoma sp. red fluorescent protein. *Nature Biotechnology*, *22*(12), 1567–1572.

Surre, J., Saint-ruf, C., Collin, V., Orenga, S., Ramjeet, M., & Matic, I. (2018). *Strong increase in the autofluorescence of cells signals struggle for survival*. 1–14.

Vollmer, A. C., Belkin, S., Smulski, D. R., Van Dyk, T. K., & Larossa, R. A. (1997). Detection of DNA damage by use of Escherichia coli carrying recA'::lux, uvrA'::lux, or alkA'::lux reporter plasmids. *Applied and Environmental Microbiology*, *63*(7), 2566–2571.

Wan, X., Volpetti, F., Petrova, E., French, C., Maerkl, S. J., & Wang, B. (2019). Cascaded amplifying circuits enable ultrasensitive cellular sensors for toxic metals. *Nature Chemical Biology*, *15*(5), 540–548.
